# An Efficient Integrated Strategy for Comprehensive Metabolite Profiling of Sakurasosaponin from Aegiceras corniculatum in Rats

**DOI:** 10.2174/0113892002299923240801092101

**Published:** 2024-08-06

**Authors:** Xiangying Wang, Xiao Yang, Erwei Hao, Jinling Xie, Zhengcai Du, Jiagang Deng, Xiaotao Hou, Wei Wei

**Affiliations:** 1 Faculty of Pharmacy, Guangxi University of Chinese Medicine, Nanning, Guangxi 530200, China;; 2 Guangxi Key Laboratory of Efficacy Study on Chinese Materia Medica, Guangxi University of Chinese Medicine, Nanning, Guangxi 530200, China

**Keywords:** *Aegiceras corniculatum*, sakurasosaponin, saponin, UHPLC-Q-TOF-MS, integrated strategy, metabolite profiling

## Abstract

**Objective:**

Sakurasosaponin, a primary bioactive saponin from Aegiceras corniculatum, shows potential as an anti-cancer agent. However, there is a lack of information on its *in vivo* metabolism. This study aims to profile the *in vivo* metabolites of sakurasosaponin in rat feces, urine, and plasma after oral administration. An efficient strategy using ultra-high-performance liquid chromatography/quadrupole time-of-flight mass spectrometry was developed, which combined metabolic prediction, multiple mass defects filtering, and high-resolution extracted ion chromatograms for rapid and systematic analysis.

**Methods:**

Firstly, a theoretical list of metabolites for sakurasosaponin was developed. This was done by considering the metabolic pathways of saponins. Next, the multiple mass defects filtering method was employed to identify potential metabolites in feces and urine, using the unique metabolites of sakurasosaponin as multiple mass defects filtering templates. Subsequently, a high-resolution extracted ion chromatogram was used to quickly determine the metabolites in rat plasma post-identification in feces and urine. Lastly, the analysis of accurate mass, typical neutral loss, and diagnostic ion of the candidate metabolites was carried out to confirm their structural elucidation, and metabolic pathways of sakurasosaponin *in vivo* were also proposed.

**Results:**

In total, 30 metabolites were provisionally identified in feces, urine, and plasma. Analysis of metabolic pathways revealed isomerization, deglycosylation, oxidation, hydroxylation, sulfate conjugation, glucuronide conjugation, and other related reactions as the primary biotransformation reactions of sakurasosaponin *in vivo*.

**Conclusion:**

The findings demonstrate that the designed research strategy effectively minimizes matrix interference, prevents the omission of low-concentration metabolites, and serves as a foundation for the discovery of active metabolites of sakurasosaponin.

## 1 INTRODUCTION

*Aegiceras corniculatum* (Linn.) Blanco, commonly known as the mangrove horned mangrove, belongs to the Aegicerataceae family. It is a significant shrub or small tree in mangrove ecosystems found in tropical and subtropical regions of Asia and Australia [1]. *A. corniculatum* is primarily distributed in the coastal provinces of China, including Guangdong, Guangxi, Fujian, and Hainan. It has been traditionally used for treating ailments such as cancer, arthritis, and inflammatory conditions [2]. Its extract has shown promising properties, including blood anti-coagulant, anti-plasmodial, and anti-inflammatory effects [3]. Studies on the phytochemistry of *A. corniculatum* have identified phenolic acids, flavonoids and their glycosides, saponins, alkaloids, triterpenoids, and various other compounds as their constituents [4, 5]. Modern pharmacological research has highlighted the extract and components of *A. corniculatum* for their anti-cancer, anti-oxidant, anti-bacterial, anti-diabetic, anti-inflammatory, analgesic, and cytotoxic activities [2, 6, 7].

In recent years, there has been considerable interest in the anti-tumor properties of *A. corniculatum* [7]. Bioactive com pounds such as alkylated benzoquinones [8], and triterpene saponins [9] have been discovered and characterized. Our previous research demon strated that 95% ethanol extract of *A. corniculatum* effectively hin dered the proliferation of DU145 prostate cancer cells, with the *n*-butanol fraction exhibiting particularly promising anti-cancer properties against prostate cancer [10, 11]. Further investigation into the underlying mechanism of action has revealed that *A. corniculatum* exerts potent anti-cancer effects on a wide range of tumor cell lines, primarily through the regulation of cell cycle progression and the induction of apoptosis [12]. These findings suggest that *A. corniculatum* has potential as an anti-cancer therapy. Apart from the pharmacological studies, the chemical analysis of the *n*-butanol extract identified 16 different compounds including flavonoid glycosides, benzoquinones, saponins, diphosphates, tetracyclic diterpenoids, and steroids. In our chemical analysis of the *n*-butanol extract of *A. corniculatum*, saponins emerged as the most abundant components. Notably, sakurasosaponin was identified as the primary bioactive compound, responsible for the pharmacological effects observed in the *n*-butanol fraction. This finding aligns with previous literature and our research, highlighting the significant role of sakurasosaponin in the anti-cancer effects of *A. corniculatum*.

In our ongoing study exploring the bioactive compounds found in this plant, we conducted compound isolation and identification of the *n*-butanol portion of *A. corniculatum*. The main bioactive constituent obtained was sakurasosaponin, a pentacyclic triterpenoid saponin. Studies have shown that sakurasosaponin isolated from *Jacquinia flammea* demonstrated antifungal and cytotoxic effects on cancer cells [13, 14]. Additionally, sakurasosaponin from *A. corniculatum* exhibited potent cytotoxicity against various cancer cell lines [9]. Research by Sung Wuk Jang *et al.* revealed that sakurasosaponin extracted from *Primula sieboldii* could trigger cell death through mitochondrial pathways. Furthermore, the antitumor property of sakurasosaponin was verified in a rat model [15]. Recent studies have suggested that sakurasosaponin inhibited the proliferation of lung cancer cells by inducing autophagy through AMPK activation [16]. These findings collectively strengthen the potential of sakurasosaponin as a promising candidate for cancer treatment.

Naturally occurring triterpenoid saponins are plant-derived secondary metabolites known for their valuable pharmacological properties, including immunomodulatory, anti-oxidative, anti-apoptotic, anti-diabetic, neuroprotective, and anti-cancer effects [17]. Recent research has focused on the metabolism of saponins *in vivo* due to their diverse pharmacological activities but limited bioavailability. Because of their physicochemical properties, saponins often exhibit low internal exposure when taken orally. Biotransformation reactions in the gastrointestinal tract produce metabolites such as secondary glycosides and aglycones, which typically have higher bioactivity than original compounds [18]. Therefore, studying the *in vivo* metabolism of saponins is crucial for identifying their active components and potentially discovering new leading compounds. There is currently a lack of literature on the *in vivo* metabolism of sakurasosaponin.

Studying metabolism is crucial for comprehending the safety and effectiveness of medications. Nevertheless, detecting and understanding the complex structures of metabolites *in vivo* poses challenges due to their low concentrations [19, 20]. Obtaining information from MS samples can be categorized into two primary methods: data-independent acquisition (DIA) mode [21] and data-dependent acquisition (DDA) mode [22]. Various MS data post-processing techniques, including mass defects filtering (MDF), multiple MDF (MMDF), neutral loss fragments filtering (NLF), and diagnostic ions filtering (DIF), have been created and utilized to enhance the efficiency of identifying metabolites in intricate biological samples [23].

Ultra-high-performance liquid chromatography/quadrupole time-of-flight mass spectrometry (UHPLC-Q-TOF-MS) has gained widespread utilization for the qualitative analysis of chemical substances *in vitro* and *in vivo*, owing to its exceptional precision, convenience, and structural identification capabilities [24]. This study aimed to rapidly and comprehensively characterize the metabolites *in vivo* post oral administration of sakurasosaponin in rats using an integrated method. Metabolites prediction (MP) was carried out based on potential metabolic pathways of saponins, resulting in the generation of a theoretical list of sakurasosaponin metabolites. Subsequently, the investigation of metabolites was conducted using a modified MMDF approach with sakurasosaponin special metabolites as templates. Identification of metabolites in rat feces and urine was followed by the application of an accurate and controllable high-resolution extracted ion chromatograms (HREIC) method to screen for potential major and trace candidate metabolites in rat plasma. The metabolites of sakurasosaponin were tentatively characterized by analyzing retention time, precise MS data, specific MS/MS fragmentation patterns such as NLs and DIs, and by referencing relevant works of literature. This research offers a more profound insight into the *in vivo* metabolic behavior of sakurasosaponin.

## 2 MATERIALS AND METHODS

### 2.1 Chemicals and Reagents

Water was obtained from a Milli-Q purification system (Millipore, Bedford, MA, USA). Acetonitrile, methanol, and formic acid of LC-MS grade were provided by Sigma-Aldrich (Sigma-Aldrich, MO, USA), while other reagents were of analytical grade. NMR spectroscopy data were recorded using a Varian INOVA 600 NMR spectrometer (^1^H 600 MHz, ^13^C 150 MHz). Mass spectrometric detection was carried out on the AB SCIEX X500R quadrupole-time of flight (QTOF) coupled with high-resolution mass spectrum (HRMS) (Applied Biosystems SCIEX, US). Column chromatography (CC) was conducted on silica gel (200-300 mesh, Qingdao Marine Chemical Ltd., Qingdao, China). Pre-HPLC was performed using a Thermo BETASIL C18 column (150 mm × 21.2 mm, 5 μm) on an RP-SP-HPLC system equipped with a 1525 binary HPLC pump and a 2489 UV detector (Waters, USA). The leaves of *A. corniculatum* were gathered from the seaside of Qinzhou city in Guangxi province, China. A voucher specimen (No. THS202111) was deposited in our lab. Sakurasosaponin was isolated, identified, and found to have a purity above 98% by HPLC and MS analysis.

### 2.2 Preparation of Sakurasosaponin

Air-dried leaves of *A. corniculatum* (2.5 kg) were powdered and subjected to extraction using 70% ethanol (8 L×4 h×5) under reflux conditions. The extraction process was carried out at a temperature of 60°C. After the removal of the organic solvent under reduced pressure, 0.75 kg of dry residue was obtained. This residue was then suspended in deionized water and sequentially extracted with petroleum ether, ethyl acetate, and *n*-butanol to obtain the respective extraction solutions. Subsequently, the organic solvents were evaporated at 45°C. The *n*-butanol fraction (103.2 g) was then chromatographed on an MCI gel column using a MeOH–H_2_O elution system (30:70, 50:50, 70:30, and 100:0, V/V) to yield sub-fractions (SFr.1-4). SFr3 underwent further purification through a SephadexLH-20 column using a CHCl_2_-MeOH elution system (1:1, V/V) followed by pre-HPLC using a MeOH-H_2_O elution system (70:30, v/v, 8 mL/min, 203 nm) to give sakurasosaponin (0.51 g). Structural characterization of sakurasosaponin was performed using advanced spectroscopic techniques such as HR-ESI-MS, ^1^H NMR, and ^13^C NMR.

### 2.3 LC-MS Analysis Condition

The study of sakurasosaponin and its breakdown products in biological samples was carried out using a quadrupole-time-of-flight (QTOF) mass spectrometer X500R (Applied Biosystems SCIEX, USA) in conjunction with ExionLC™ AD (Applied Biosystems SCIEX, US) ultra-fast and efficient liquid chromatography system, employing a Hypersil Gold column (2.1 mm × 100 mm, 1.9 µm, Thermo Scientific, USA). The LC-MS parameters are basically consistent with those reported in our previous literature [25]. The temperature of the column and the rate of flow were adjusted to 40°C and 0.4 mL/min, respectively, with an injection volume of 3 μL for both control and medicated biological samples. The mobile phase consisted of solvent A (water with 0.1% formic acid) and solvent B (acetonitrile), with the following optimized gradient elution program: 0-5 min, 12% B; 5-6 min, 12-24% B; 6-15 min, 24-57% B; 15-35 min, 57-72% B; and 35-40 min, 72-12% B. Analysis by mass spectrometry was carried out in a negative mode using full scan acquisition. The MS and MS/MS data of the compounds were obtained in information-dependent acquisition (IDA) mode, with electrospray ionization (ESI) as the ion source. The optimized settings for IDA were as follows: ion source gas 1 (GS1): 55 psi; ion source gas 2 (GS2): 55 psi; curtain gas: 35 psi; temperature: 600°C; and CAD gas: 7. For TOF MS: mass range, m/z 100–2000 Da; declustering potential (DP): ±80 V; collision energy (CE): ±35 V; and CE spread: 0 V. For TOF MS/MS: mass range of fragments, m/z 100-2000 Da; DP: ±80 V; CE: ±35 V; CE spread: 15 V; and accumulation time: 0.1 s. Data acquisition and analysis were managed using SCIEX OS software (Ver. 1.3.1, AB SCIEX Co.).

### 2.4 Animal Experiment

Six male SD rats weighing 220 ± 20 g were acquired from Hunan Silaikejingda Experimental Animal Co., Ltd. (ethical review certificate number: DW20211216-208), and placed in a controlled environment with a room temperature of 24 ± 2 °C and humidity at 70 ± 5%, following a 12 h light/12 h dark schedule. Following a one-week adjustment period, the rats were randomly assigned to the Drug Group (n = 3) to analyze plasma, urine, and feces, and the Control Group (n = 3) to analyze blank plasma, urine, and feces. Prior to the experiment, all rats underwent a 12-hour fasting period with unrestricted access to water. The suspension of Sakurasosaponin in distilled water was utilized for drug administration. All experiments were conducted in accordance with the Regulations of Experimental Animal Administration issued by the State Commission of Science and Technology of the People’s Republic of China. Experimental animal protocols were approved by the Animal Ethics Committee of the Guangxi University of Chinese Medicine, and all procedures followed the relevant regulations and guidelines.

### 2.5 Collection and Pretreatment of Biological Samples

The 100 mg/kg dosage of the drug was orally administered to each rat in the drug group, while rats in the control group received the same volume of distilled water. Blood samples (0.2 mL each) were collected from the suborbital venous plexus of rats at specified time points (0, 0.5, 1, 3, 5, 9, and 11 hours post-administration). Centrifugation was performed at 3500 rpm for 10 minutes for each sample. Urine and feces samples were gathered from the control group within the designated time frame (0–11 hours) post oral administration, contrasting with the drugged group that contained the drug in their samples. All biological samples from each group were combined into a single sample. Feces samples, both blank and drug-containing, were treated with 50% aqueous methanol and underwent ultrasonic treatment for 10 minutes. Subsequent centrifugation of the extractions allowed for the evaporation of the obtained supernatant liquid under reduced pressure. The remaining residues were dissolved in 1 mL of 50% aqueous acetonitrile. Following microporous membrane filtration, 5 μL of the solution was injected into the LC-MS system for UHPLC-Q-TOF-MS analysis.

### 2.6 Data Post-processing Procedures

The AB SCIEX EX X500R QTOF system was utilized for data acquisition. Utilizing SCIEX OS software (Ver. 1.3.1, AB SCIEX Co.), UHPLC-Q-TOF-MS and MS/MS data were acquired and processed. Parameters for the MS^1^ data included a 10 ppm Mass Tolerance, a Time Range aligned with UHPLC running time, and Mass Range *m/z* 100-2000 Da. Chemical peak formulas were identified using the “Mass calculators” feature of SCIEX OS software, with parameters set as C [0–90], H [0–100], O [0–90], and Ring Double Bond (RDB) equivalent value [0–15]. The chemical formulas of selected peaks were determined based on HRMS^1^ data. In Metabolitepilot^TM^ 2.2.4 software, parameters such as Charge state 1, Adduct [M-H]^-^, [M+HCOOH-H]^-^, and [M+Cl]^-^, with a minimum peak intensity of ≥100 cps for both MS and MS/MS peaks. Formula Prediction ranged from C_5_H_8_O_3_ to C_80_H_138_N_15_O_47_P_5_S_5_ for possible metabolites. The mass error for the MS/MS fragment was ±15 ppm, retention time window matching the UHPLC analysis running time. Isotope Pattern Tolerance set at 5 ppm for MS, and Isotope Peak Finding at 10 ppm for MS/MS *m/z* tolerance.

## 3 RESULTS AND DISCUSSIONS

### 3.1 Separation and Structural Elucidation of Sakurasosaponin (M0)

**Fig. (1):**
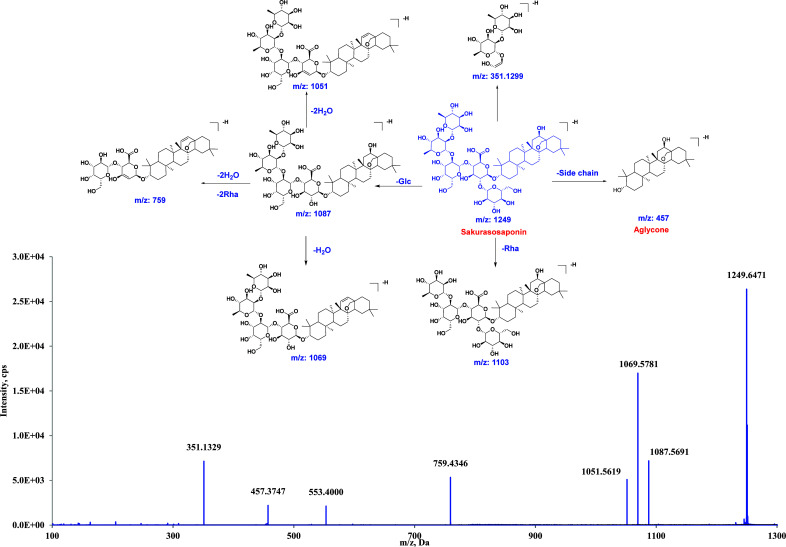
The chemical structure of sakurasosaponin isolated from *A. corniculatum* and its proposed MS/MS fragment pathways in negative ion mode.

After the separation and purification, the chemical structure of sakurasosaponin (parent drug, **M0**) was elucidated based on spectroscopic data analysis, including HR-ESI-MS, and NMR (Fig. **S1-3**). Sakurasosaponin was obtained as a withe amorphous solid. Its chemical formula was calculated to be C_60_H_98_O_27_ through negative HR-ESI-MS precursor ion at *m/z* 1249.6221 [M-H]^-^. The NMR data is shown below the (Fig. **S3**). On the basis of spectral evidence and comparison of its spectral data with those data reported in the literature, the parent drug (**M0**) was unambiguously identified as sakurasosaponin (Fig. **1**) [15].

### 3.2 MS/MS Fragment Patterns of Sakurasosaponin (M0)

Due to the structural similarity between the parent compound and its metabolites *in vivo*, it is essential to analyze the MS/MS fragment pathway of the parent drug to elucidate the structure of metabolites. Therefore, a thorough comprehension of the fragment pathway of sakurasosaponin is necessary. The LC-MS instrument was used to subject Sakurasosaponin (**M0**) to analysis, and its MS/MS fragment pathway was examined in negative ion mode under the specified analytical conditions. Negative ion mode was chosen for the analysis due to the higher ion intensity compared to positive ion mode. The base peak identified at a retention time of 24.59 min in the first-order MS spectrum was confirmed as sakurasosaponin based on the accurate MS data and MS/MS behavior. It exhibited a deprotonated ion at *m/z* 1249.6423 [M-H]^−^, indicating a molecular formula of C_60_H_98_O_27_. Moreover, in the MS/MS spectrum, a characteristic fragment ion at *m/z* 1069.5596 [M-H-H_2_O-Glc]^-^ was formed through the sequential loss of one H_2_O and one Glc group. The loss of Glc or Rha from the sugar side chain attached to the C-3 position resulted in the production of ions at *m/z* 1087.5607 [M-H-Glc]- and 1103.5648 [M-H-Rha]-, respectively. The fragment ion at m/z 759.4346 was generated by the loss of a Glc group, followed by 2H_2_O and 2Rha groups. The fragment ion at m/z 457.3699 was obtained by the removal of the glycoside chain connected to the aglycone of sakurasosaponin from the C-3 position. It can be inferred that the neutral losses (NLs) of H_2_O (18 Da), Glc (162 Da), and Rha (146 Da), or their combination were the characteristic NLs for sakurasosaponin. The corresponding product ions following the aforementioned NLs, such as *m/z* 351.1299, 457.3684, 1069.5615, and 1087.5707, were identified as the characteristic diagnostic ions (DIs) for the recognition of its metabolites *in vivo*. The primary NLs and DIs of sakurasosaponin are summarized in Table **S1**, while its chemical structure and MS/MS fragment pathways are illustrated in (Fig. **1**).

### 3.3 Establishment of the Integrated Strategy and Data Post Processing

#### 3.3.1 Metabolic Prediction (MP) of Sakurasosaponin (M0)

In contrast to other naturally occurring components, saponins have low bioavailability when taken orally, with gastrointestinal metabolites being the primary form absorbed into the blood. Following oral administration, saponins typically undergo various structural modifications, such as stepwise deglycination, oxidation, hydrogenation, dehydrogenation, and hydroxylation, among other composite reactions. Consequently, the potential metabolites of sakurasosapon in *in vivo* can be predicted. To streamline the elucidation of its *in vivo* metabolites, sakurasosaponin served as the foundational template to generate a range of potential metabolites, encompassing common and unique bio-transformations. According to existing literature, the proposed regular metabolic pathways of sakurasosaponin consist of decarboxylation, hydroxymethylene loss, bis-demethylation, CO loss, H_2_O loss, CO_2_ loss, demethylation and desaturation, demethylation, demethylation and hydrogenation, desaturation, glycine conjugation, oxidation, sulfate conjugation, glutamine conjugation, among others. A total of 117 conventional metabolic routes of sakurasosaponin and their associated mass shifts are tabulated in Table **S2**. Additionally, through scrutinizing the product ions of sakurasosaponin in its MS/MS spectrum, 44 potential distinct metabolites were identified, and their detailed MS information, including “Loss from Parent”, “Neutral Formula”, “*m/z* [M-H]^-^”, and “*m/z* [M+FA-H]^-^”, are documented in Table **S3**. Subsequently, all the aforementioned hypothetical metabolites were analyzed using the Metabolitepilot^TM^ 2.2.4 software to ascertain the metabolic candidate compounds.

#### 3.3.2 Establishments of MMDF and HREIC Methods

*In vivo* , the original drug and its metabolites typically display a similar core substructure but vary in their substituted components, suggesting comparable mass defects among these constituents. Over the last few decades, Mass Defect Filtering (MDF) techniques have been developed and utilized to swiftly identify the primary to minor metabolites present in diverse biological samples [26, 27]. Serving as a user-friendly approach to data analysis, MDF has demonstrated significant benefits in distinguishing metabolites from complex sample interference. To address the intricacies of biotransformation processes, a revised MMDF approach was devised in this investigation to effectively handle the High-Resolution Mass Spectrometry (HRMS) data of intricate metabolic byproducts. A critical aspect of implementing the MMDF method involves selecting suitable MMDF templates. By considering potential metabolic pathways of saponins, transformations such as glucuronidation, glutathione conjugation, bis-glucuronidation, as well as deglycosylation (*e.g.*, Glc, Xyl, Rha loss) and their combinations were used to construct MMDF templates for sakurasosapoin. This effort yielded a total of 49 potential sakurasosapoin metabolites, along with their corresponding mass spectrometry details, tabulated in Table **1** and employed for MMDF template generation. The Metabolitepilot^TM^ 2.2.4 software was employed for peak identification, with MMDF templates calibrated within ± 50 mDa of the mass defect and a mass window of ± 50 Da centered on the filter mass. The MMDF size cap was set at 113-1662 Da to encompass the metabolites.

Due to the poor bioavailability of saponins, the absorbed components of sakurasosaponin typically appear in low concentrations in plasma. Consequently, the peak signals produced by the corresponding metabolites may be obscured by potential interferences from endogenous substances. To swiftly detect sakurasosaponin metabolites in rat plasma, a precise and manageable HREIC method was utilized. The prototype and metabolites of the parent compound in rat plasma were promptly identified by entering the chemical formulas of the identified components in feces and urine into the SCIEX OS software's compound list. Then, the extraction of the target components was carried out in both blank and medicated plasma using an Extracted Ion Chromatogram (EIC). A mass tolerance of 10 ppm and a peak width of 0.02 mDa were implemented. The peaks exclusively present in the medicated plasma were identified as potential metabolites.

#### 3.3.3 Structural Identification of the Metabolites

The investigation of the MS/MS pathway patterns of sakurasosaponin (**M0**) in section **3.2** was crucial for the structural elucidation of the metabolites present in rat feces, urine, and plasma. The summary of the corresponding NLs and DIs of sakurasosaponin (**M0**) can be found in Table **S3**, serving as a reference for deducing the metabolic pathways of its metabolites. A detailed analysis of the precise MS and MS/MS data of the primary and trace metabolites of sakurasosaponin was carried out on all potential metabolites.

**Fig. (2):**
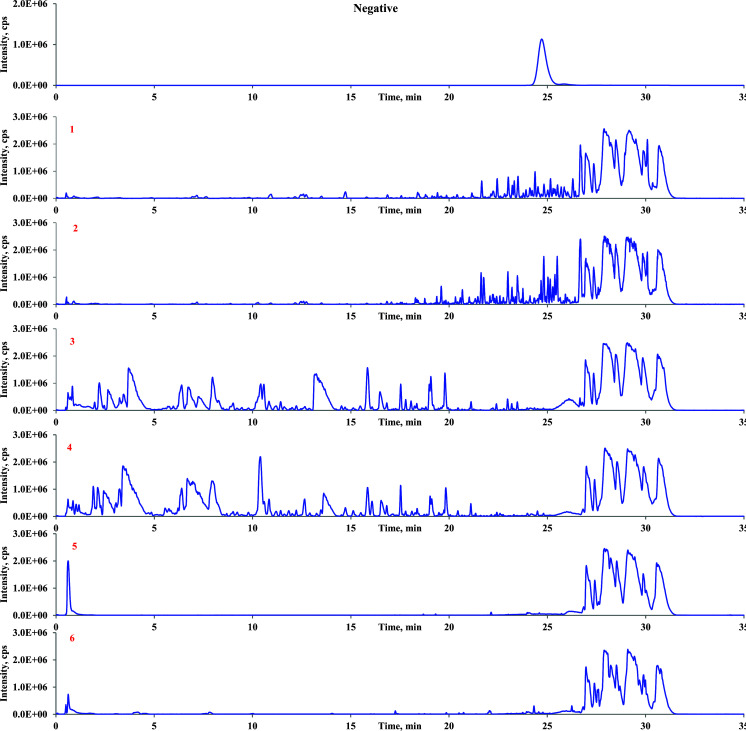
The BPCs of sakurasosaponin, rat plasma, feces, and urine. 0: Sakurasosaponin. 1: blank feces sample. 2: drug-containing feces sample. 3: blank urine sample. 4: drug-containing urine sample. 5: blank plasma sample. 6: drug-containing plasma sample.

**Fig. (3):**
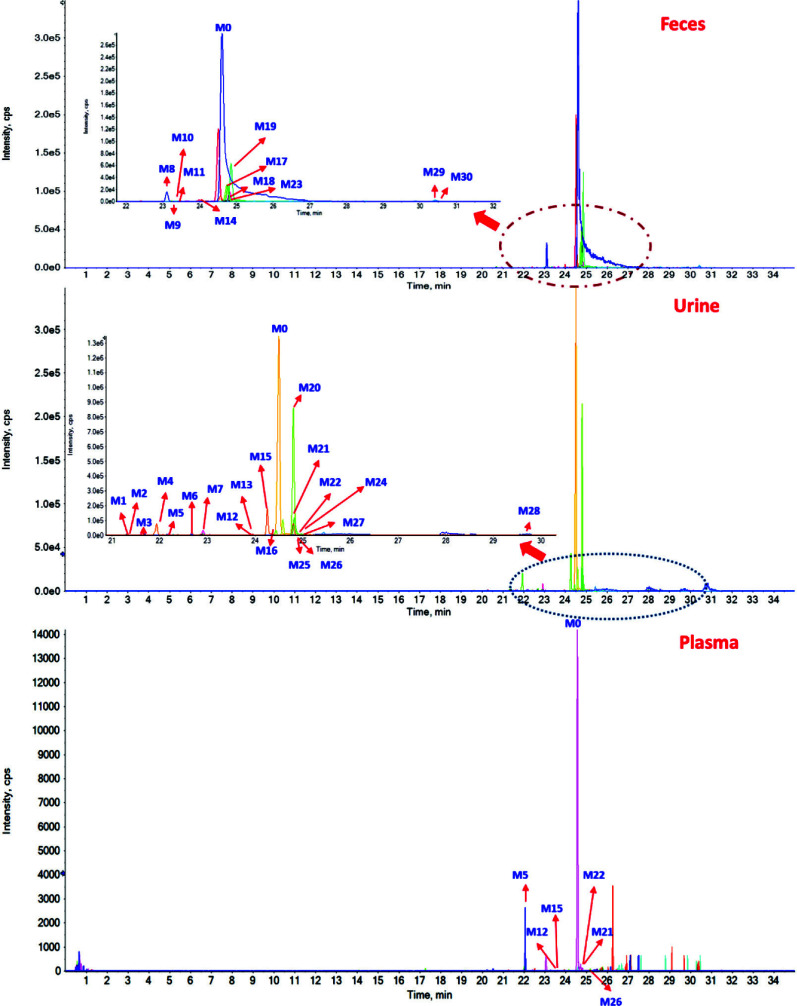
The HREICs of drugged rat plasma, feces, and urine in negative ion mode.

**Fig. (4):**
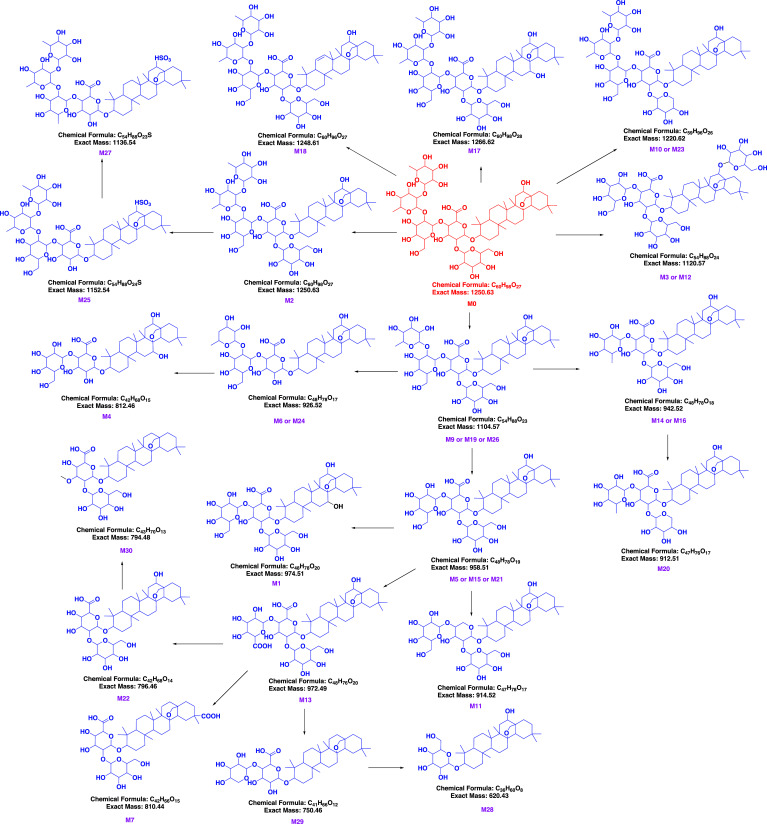
The proposed metabolic pathways of sakurasosaponin in rat feces, urine and plasma.

The gradual breakdown of glycosyl or glucuronosyl groups from saponins typically produces secondary glycosides, aglycone, or both [27]. Neutral losses (NLs) were extensively utilized for targeted screening of altered metabolites susceptible to neutral eliminations. Additionally, NLs exhibited exceptional selectivity and sensitivity in profiling and characterizing specific conjugated compounds in biological samples. Glc (162 Da) and Rha (146 Da), or their combination, was initially computed in the MS/MS spectra of the metabolites of sakurasosaponin for structural elucidation. The resulting diagnostic ions (DIs) from the detachment of sugar moieties were employed to confirm the metabolite structure. Upon thorough examination of the MS/MS fragmentation patterns of the parent compound, the signature DI of [A-H]^-^ was identified as distinctive for sakurasosaponin and was prioritized for primary structural determination of the metabolites. Subsequently, other DIs (Table **S1**) were also utilized as supplementary evidence for structural clarification. Comprehensive MS/MS data, encompassing chemical composition, characteristic product ions, key peaks for assignment, fragmentation behavior, structural nuances, and more, for all potential metabolites in feces, urine, and plasma, were documented on pages 11-227 of the supplementary information. The base peak chromatograms (BPCs) of sakurasosaponin, as well as rat plasma, feces, and urine, are illustrated in (Fig. **2**). Ultimately, a total of 30 metabolites (including **M0**) were provisionally characterized in rat feces, urine, and plasma under negative ion mode. The HREICs of the treated rat plasma, feces, and urine in negative ion mode are depicted in (Fig. **3**). Detailed MS and MS/MS data of the identified metabolites are summarized in Table **2**, with inferred metabolic pathways illustrated in (Fig. **4**). The major metabolic pathways were isomerization, deglycosylation, oxidation, hydroxylation, sulfate conjugation, glucuronide conjugation, and their composite reactions and so on. **M3** and **M14** were selected as the representative examples for the structural identification of the metabolites.

**M3** exhibited a dehydrogenation ion at *m/z* 1219.6234 [M-H]^-^, suggesting the chemical formula of C_59_H_96_O_26_. In the MS/MS spectrum, an *m/z* of 1101.5259 was observed after the loss of one H_2_O from the parent ion. The ions observed at *m/z* 1057.1336 and 1039.5613 indicated the successive losses of one Glc, one Glc, and one H_2_O group from the initial structure. As previously noted, [A-H]^-^ was a distinctive DI in the MS/MS fragment pathway of sakurasosaponin. The fragment ion at *m/z* 457.3703[M-H]^-^ suggested the detachment of the glycosyl side chain. Additionally, other unique DIs such as *m/z* 351.1425 and *m/z* 205.0774 were identified. Based on the aforementioned information, **M3** was provisionally identified as the metabolite formed by the elimination of one hydroxymethylene group from the core structure of sakurasosaponin. The fragmentation pathways and detailed MS data of **M3** are depicted in Fig. (**5**) and can be found in pages 18-21 of the supporting documents.

**Fig. (6):**
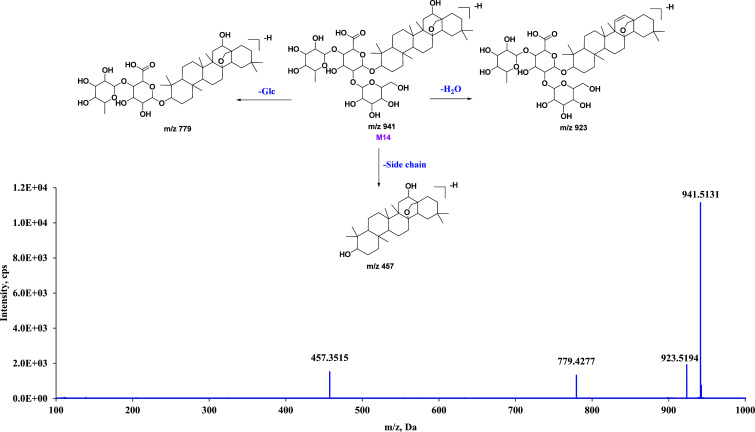
The proposed metabolic pathway of **M14** in negative ion mode.

**M14** provided an [M-H]^-^ ion with a mass-to-charge ratio of 941.5131, indicating a chemical formula of C_48_H_78_O_18_. Additionally, the analysis of the ESI-MS/MS spectrum revealed a loss of one H_2_O molecule, resulting in a product ion at *m/z* 923.5194. Subsequent removal of the Glc moiety from the original structure led to the detection of a product ion at *m/z* 779.4277. The distinctive DIs observed at *m/z* 457.3515 allowed for the inference that **M14** shared a similar core structure with sakurasosaponin. The chemical makeup and pathways of metabolism for **M14** are illustrated in (Fig. **6**) and detailed on pages 87-91 of the supporting information. Additional information can be found therein.

## CONCLUSION

It is common knowledge that drug efficacy and safety are closely linked to *in vivo* metabolism. Analyzing the structures of metabolites of active components is often seen as a challenging aspect of drug metabolism studies. Metabolites found in biological samples are typically present in low concentrations, which can hinder their detection as their signals may be obscured by background noise and interference from other molecules. In this research, a rapid and effective strategy was developed using UHPLC-Q-TOF-MS in combination with MP, MMDF, HREIC, NLs, and DIs to aid in identifying the metabolites of sakurasosaponin. The main metabolites were successfully identified in rat feces, urine, and plasma post-oral intake. A total of 30 metabolites were provisionally identified through precise mass measurements, fragment patterns, NLs, and DIs. Previous studies have suggested that saponins have a prolonged residence time in the intestines after ingestion, with their metabolites eventually being absorbed into the bloodstream. Results indicated that the primary compound found in rat feces and urine was sakurasosaponin, consistent with findings from existing literature [28]. After ingestion, sakurasosaponin proved challenging to metabolize, with the majority of the compound and its metabolites being excreted in feces and urine, and only trace amounts appearing in plasma. The major metabolic pathways for sakurasosaponin in rats are believed to involve deglycosylation, oxidation, desaturation, methylation, glucuronidation, *O*-sulphate conjugation, and deglycation. Among these, deglycosylation is the primary metabolic reaction. After oral administration of sakurasosaponin, glycosidic hydrolysis occurs first, and the resulting corresponding metabolites then undergo further metabolic reactions. Besides, there was a great difference among metabolites detected in urine and feces, which might be attributed to the different pathways *in vivo*.

The results presented demonstrate that the suggested approach proved to be effective in efficiently detecting and characterizing drug-related components of sakurasosaponin in rats, thus enhancing our comprehension of the pharmacological effects and potential for sakurasosaponin in new drug discovery.

## Figures and Tables

**Fig. (5):**
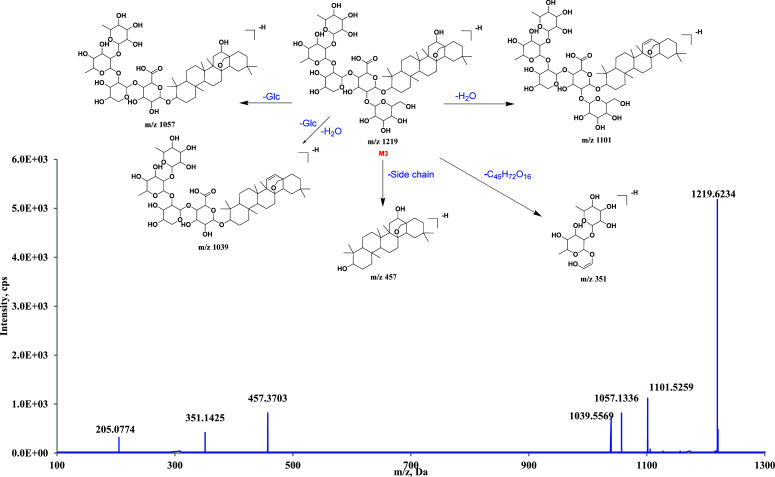
The proposed metabolic pathway of **M3** in negative ion mode.

**Table 1:** The possible compound special metabolites of sakurasosapoin that were used as MMDF templates.

**No.**	**Possible bio-reaction of sakurasosaponin (M0)**	**Formula of the possible metabolite**	**m/z**	**Defect**	**Mass Defect Window (mDa) - below**	**Mass Defect Window (mDa) - above**	**Mass Range - from m/z**	**Mass Range - to m/z**
1	Loss of C_48_H_76_O_19_ and C_6_H_10_O_5_	C_6_H_12_O_3_	131.0714	0.0714	30	20	113	191
2	Loss of C_54_H_86_O_23_	C_6_H_12_O_4_	147.0663	0.0663	30	20	129	207
3	Loss of C_54_H_86_O_22_	C_6_H_12_O_5_	163.0612	0.0612	30	20	145	223
4	Loss of C_54_H_86_O_21_	C_6_H_12_O_6_	179.0561	0.0561	30	20	161	239
5	Loss of C_48_H_76_O_19_ and O	C_12_H_22_O_7_	277.1293	0.1293	30	20	259	337
6	Loss of C_48_H_76_O_19_	C_12_H_22_O_8_	293.1242	0.1242	30	20	275	353
7	Loss of C_48_H_76_O_18_	C_12_H_22_O_9_	309.1191	0.1191	30	20	291	369
8	Loss of C_30_H_48_O_3_ and C_18_H_30_O_14_	C_12_H_20_O_10_	323.0984	0.0984	30	20	305	383
9	Loss of C_42_H_66_O_13_ and C_6_H_10_O_4_	C_12_H_22_O_10_	325.114	0.114	30	20	307	385
10	Loss of C_30_H_48_O_2_ and C_18_H_30_O_14_	C_12_H_20_O_11_	339.0933	0.0933	30	20	321	399
11	Loss of C_30_H_48_O_2_ and C_18_H_30_O_13_	C_12_H_20_O_12_	355.0882	0.0882	30	20	337	415
12	Loss of O and C_30_H_48_O_25_	C_30_H_50_O	425.3789	0.3789	30	20	407	485
13	Loss of C_42_H_66_O_14_ and O	C_18_H_32_O_12_	439.1821	0.1821	30	20	421	499
14	Loss of C_30_H_48_O_25_	C_30_H_50_O_2_	441.3738	0.3738	30	20	423	501
15	Loss of C_42_H_66_O_14_	C_18_H_32_O_13_	455.177	0.177	30	20	437	515
16	Loss of C_30_H_48_O_24_	C_30_H_50_O_3_	457.3687	0.3687	30	20	439	517
17	Loss of C_42_H_66_O_13_	C_18_H_32_O_14_	471.1719	0.1719	30	20	453	531
18	Loss of C_30_H_48_O_3_ and C_12_H_20_O_9_	C_18_H_30_O_15_	485.1512	0.1512	30	20	467	545
19	Loss of C_30_H_48_O_2_ and C_12_H_20_O_9_	C_18_H_30_O_16_	501.1461	0.1461	30	20	483	561
20	Loss of C_30_H_48_O_2_ and C_12_H_20_O_8_	C_18_H_30_O_17_	517.141	0.141	30	20	499	577
21	Loss of C_6_H_10_O_6_ and C_18_H_30_O_14_	C_36_H_58_O_7_	601.411	0.411	30	20	583	661
22	Loss of C_30_H_48_O_3_ and C_6_H_10_O_6_	C_24_H_40_O_18_	615.2142	0.2142	30	20	597	675
23	Loss of C_6_H_10_O_6_ and C_18_H_30_O_13_	C_36_H_58_O_8_	617.4059	0.4059	30	20	599	677
24	Loss of C_30_H_48_O_2_ and C_6_H_10_O_6_	C_24_H_40_O_19_	631.2091	0.2091	30	20	613	691
25	Loss of C_6_H_10_O_5_ and C_18_H_30_O_13_	C_36_H_58_O_9_	633.4008	0.4008	30	20	615	693
26	Loss of C_30_H_48_O_2_ and C_6_H_10_O_5_	C_24_H_40_O_20_	647.204	0.204	30	20	629	707
27	Loss of C_30_H_48_O_2_ and C_6_H_10_O_4_	C_24_H_40_O_21_	663.1989	0.1989	30	20	645	723
28	Loss of O and C_18_H_30_O_14_	C_42_H_68_O_12_	763.4638	0.4638	30	20	745	823
29	Loss of C_30_H_48_O_3_ and O	C_30_H_50_O_23_	777.267	0.267	30	20	759	837
30	Loss of C_18_H_30_O_14_	C_42_H_68_O_13_	779.4587	0.4587	30	20	761	839
31	Loss of C_30_H_48_O_3_	C_30_H_50_O_24_	793.2619	0.2619	30	20	775	853
32	Loss of C_18_H_30_O_13_	C_42_H_68_O_14_	795.4536	0.4536	30	20	777	855
33	Loss of C_30_H_48_O_2_	C_30_H_50_O_25_	809.2568	0.2568	30	20	791	869
34	Loss of C_6_H_10_O_6_ and C_6_H_10_O_5_	C_48_H_78_O_16_	909.5217	0.5217	30	20	892	970
35	Loss of O and C_12_H_20_O_9_	C_48_H_78_O_17_	925.5166	0.5166	30	20	908	986
36	Loss of C_12_H_20_O_9_	C_48_H_78_O_18_	941.5115	0.5115	30	20	924	1002
37	Loss of C_12_H_20_O_8_	C_48_H_78_O_19_	957.5065	0.5065	30	20	940	1018
38	Loss of O and C_6_H_10_O_6_	C_54_H_88_O_20_	1055.58	0.5796	30	20	1038	1116
39	Loss of C_6_H_10_O_6_	C_54_H_88_O_21_	1071.575	0.5745	30	20	1054	1132
40	Loss of C_6_H_10_O_5_	C_54_H_88_O_22_	1087.569	0.5694	30	20	1070	1148
41	Loss of C_6_H_10_O_4_	C_54_H_88_O_23_	1103.564	0.5644	30	20	1086	1164
42	Loss of O and O	C_60_H_98_O_25_	1217.632	0.6324	30	20	1200	1278
43	Loss of O	C_60_H_98_O_26_	1233.627	0.6274	30	20	1216	1294
44	Loss of H-_2_O	C_60_H_100_O_26_	1235.643	0.643	30	20	1218	1296
45	Parent	C_60_H_98_O_27_	1249.622	0.6223	30	20	1232	1310
46	Sulphate	C_60_H_98_O_30_S	1329.579	0.5791	30	20	1312	1390
47	Glucuronidation	C_66_H_106_O_33_	1425.654	0.6544	30	20	1408	1486
48	Glutathione	C_70_H_115_N_3_O_33_S	1556.706	0.7061	30	20	1539	1617
49	Bis-Glucuronidation	C_72_H_114_O_39_	1601.686	0.6864	30	20	1584	1662

**Table 2:** Summary of the sakurasosaponin metabolites in rat feces, urine, and plasma.

**Peak ID**	**Charge ([M-H]-)**	**RT (min)**	**Formula**	**Neutral Mass**	**Experimental mass *m/z***	**Error** **(ppm)**	**MS/MS Peaks selected for assignment (m/z)**	**Biotransformation**	**Source**
M1	-1	21.30	C_48_H_78_O_20_	974.52	973.5085	7.3	644.6539, 955.5069, 973.3659	Loss of C_12_H_20_O_8_+Oxidation [M-H]-	U
M2	-1	21.33	C_60_H_98_O_27_	1250.64	1249.6328	8.4	365.1182, 425.1299, 445.1563, 481.1906, 620.9673, 624.3121, 645.3482, 671.4936, 678.1662, 875.2764, 923.5005, 937.0390, 1187.6412, 1203.6421, 1211.7120, 1231.6314, 1249.6351	Isomer of Parent [M-H]- (Or isomer pf M1)	U
M3	-1	21.60	C_54_H_88_O_24_	1120.58	1119.5681	7.9	777.4948, 1119.4503	Loss of C_12_H_20_O_8_+Glucose Conjugation [M-H]-	U
M4	-1	22.18	C_42_H_68_O_15_	812.46	811.4548	7.7	309.9108, 400.9102, 441.9106, 447.1261, 473.3870, 475.0693, 513.2119, 541.4244, 631.3923, 811.4573	Loss of C_18_H_30_O_13_+Oxidation [M-H]-	U
M5	-1	22.38	C_48_H_78_O_19_	958.52	957.5124	6.2	493.9477, 647.7495, 652.2441, 912.1525, 957.5163	Loss of C_12_H_20_O_8_ [M-H]-	U, P
M6	-1	22.90	C_47_H_74_O_18_	926.49	925.4873	7.7	310.9522, 355.2198, 359.2704, 471.3640, 629.3727, 646.2919, 654.8217, 717.4314, 863.4913, 925.4900	Loss of O and C_12_H_20_O_9_+Demethylation and Methylene to Ketone [M-H]-	U
M7	-1	22.94	C_42_H_66_O_15_	810.45	809.4387	7.2	304.9150, 399.1974, 415.1412, 475.2714, 485.0434, 521.1603, 537.9054, 549.3722, 557.2578, 727.2027, 809.4423	Loss of C_18_H_30_O_14_+Demethylation to Carboxylic Acid [M-H]-	U
M8	-1	23.10	C_60_H_98_O_27_	1250.64	1249.6292	5.5	351.1341, 455.3531, 457.3736, 582.2263, 759.4383, 1044.8579, 1069.5758, 1087.5689, 1103.6178, 1199.5322, 1249.6314	Isomer of Parent [M-H]- (Isomer of M0)	F
M9	-1	23.24	C_54_H_88_O_23_	1104.58	1103.5775	11.9	469.2325, 923.5068, 1035.5142, 1085.5386, 1103.6418	Loss of C_6_H_10_O_4_ [M-H]-	F
M10	-1	23.35	C_59_H_96_O_26_	1220.63	1219.6223	8.6	351.1340, 788.5395, 1219.4378	Loss of Hydroxymethylene [M-H]-	F
M11	-1	23.66	C_47_H_78_O_17_	914.53	913.5246	8.7	339.2034, 407.2857, 459.7042, 471.2378, 493.2093, 505.3584, 586.0536, 605.9391, 661.5858, 751.4402, 825.4615, 867.4817, 913.5265	Loss of C_12_H_20_O_8_+Decarboxylation [M-H]-	F
M12	-1	23.82	C_54_H_88_O_24_	1120.58	1119.569	8.6	339.2028, 1073.5454, 1101.5588, 1119.5729	Loss of C_12_H_20_O_8_+Glucose Conjugation [M-H]- (Isomer of M3)	U, P
M13	-1	23.95	C_48_H_76_O_20_	972.5	971.4931	7.6	664.1911, 748.3449, 925.4744, 953.4725, 953.4961, 971.4975	Loss of C_12_H_20_O_9_+Demethylation to Carboxylic Acid [M-H]-	U
M14	-1	23.99	C_48_H_78_O_18_	942.53	941.5189	7.8	471.3140, 497.2960, 513.2837, 519.3451, 533.8852, 573.3781, 597.3693, 627.6491, 633.4079, 777.4566, 795.4616, 827.5398, 873.4570, 879.1636, 895.4068, 941.5218	Loss of C_12_H_20_O_9_ [M-H]-	F
M15	-1	24.26	C_48_H_78_O_19_	958.52	957.513	6.8	323.1051, 457.3733, 499.1387, 525.3966, 525.4116, 597.3881, 615.3983, 733.4627, 777.4518, 795.4735, 895.5160, 939.5086, 957.5151	Loss of C_12_H_20_O_8_ [M-H]- (Isomer of M5)	U, P
M16	-1	24.49	C_48_H_78_O_18_	942.52	941.517	5.8	307.1055, 325.1175, 457.3728, 571.4055, 597.3869, 615.3976, 633.4045, 733.4612, 751.4734, 795.4616, 879.5202, 897.5323, 923.5115, 941.5188	Loss of C_12_H_20_O_9_ [M-H]- (Isomer of M14)	U
M17	-1	24.56	C_60_H_98_O_28_	1266.63	1265.6252	6.3	367.1291, 457.3617, 759.4238, 847.3978, 1085.5661, 1152.9982, 1248.5979, 1265.1758	Oxidation [M-H]-	F
M0	-1	24.59	C_60_H_98_O_27_	1250.63	1249.626	3.0	291.1112, 301.0589, 309.1224, 351.1338, 457.3741, 553.3947, 733.4568, 759.4418, 1051.5615, 1069.5696, 1103.5783, 1231.6261, 1249.6404	Parent [M-H]-	F, U, P
M18	-1	24.75	C_60_H_96_O_27_	1248.62	1247.6144	6.2	351.1348, 757.4277, 859.1286, 1067.5239, 1247.6213	Desaturation [M-H]-	F
M19	-1	24.76	C_54_H_88_O_23_	1104.58	1103.5717	6.6	293.1826, 301.0612, 455.3568, 457.3737, 553.3931, 759.4420, 777.4732, 795.4589, 905.5056, 923.5116, 939.4972, 941.5058, 1099.8516, 1099.9372, 1103.4407, 1103.5732	Loss of C_6_H_10_O_4_ [M-H]- (Isomer of M9)	F
M20	-1	24.81	C_47_H_76_O_17_	912.51	911.5067	6.3	457.3738, 571.4067, 597.3862, 615.3628, 703.4509, 721.4603, 765.4521, 849.5109, 867.5199, 893.5009, 911.5077	Loss of C_12_H_20_O_9_+Loss of Hydroxymethylene [M-H]-	U
M21	-1	24.81	C_48_H_78_O_19_	958.52	957.5148	8.8	590.9887, 615.3929, 703.4552, 849.5078, 893.4914, 908.4195, 911.1536, 911.3822, 911.4195, 957.5508	Loss of C_12_H_20_O_8_ [M-H]- (Isomer of M5)	U, P
M22	-1	24.82	C_42_H_68_O_14_	796.47	795.4603	8.4	407.3407, 437.3434, 457.3737, 615.3962, 633.4034, 727.3869, 733.4586, 751.4742, 795.4602	Loss of C_18_H_30_O_13_ [M-H]-	U, P
M23	-1	24.86	C_59_H_96_O_26_	1220.63	1219.6185	5.5	301.0634, 309.1206, 351.1328, 423.1534, 455.3583, 457.3729, 525.4056, 553.3957, 759.4414, 805.5719, 1011.5580, 1039.5614, 1201.6138, 1219.6215	Loss of Hydroxymethylene [M-H]- (Isomer of M10)	F
M24	-1	25.09	C_48_H_78_O_17_	926.53	925.5238	7.8	369.2134, 669.3263, 857.6846, 907.5060, 925.5270	Loss of O and C_12_H_20_O_9_ [M-H]- (Isomer of M6)	U
M25	-1	25.19	C_54_H_88_O_24_S	1152.54	1151.5359	4.0	943.4498, 1083.4801, 1105.4432, 1105.6383, 1151.4019, 1151.5386	Loss of C_6_H_10_O_6_+Sulfate Conjugation [M-H]-	U
M26	-1	25.44	C_53_H_84_O_24_	1104.54	1103.5345	5.9	971.5122, 1039.5077, 1049.5785, 1057.6153, 1067.4511, 1085.5931, 1103.5303	Loss of C_6_H_10_O_4_+Demethylation and Methylene to Ketone [M-H]- (Isomer of M9, or M19)	U, P
M27	-1	25.44	C_54_H_88_O_23_S	1136.55	1135.5383	1.6	763.4120, 789.3445, 793.2904, 817.4260, 925.4514, 941.5129, 943.4786, 963.5201, 1005.5630, 1049.5658, 1067.4891, 1085.9071, 1135.5446	Loss of O and C_6_H_10_O_6_+Sulfate Conjugation [M-H]-	U
M28	-1	29.7	C_36_H_60_O_8_	620.43	619.4267	8.3	315.0284, 325.1903, 357.1568, 376.2242, 459.0020, 501.3310, 573.4261, 583.3969, 601.4234, 619.2320, 619.4430	Loss of C_30_H_48_O_24_+Glucose Conjugation [M-H]-	U
M29	-1	30.41	C_41_H_66_O_12_	750.45	749.4431	-6.8	311.1677, 325.1875, 327.1871, 339.2037, 341.2030, 371.1717, 387.1460, 407.1887, 657.2507, 665.3751, 703.3264, 731.4928, 749.3501, 749.4433	Loss of C_18_H_30_O_14_+Loss of Hydroxymethylene [M-H]-	F
M30	-1	30.43	C_43_H_70_O_13_	794.47	793.4673	-9.0	311.1699, 325.1872, 339.2049, 385.9776, 426.9271, 445.2952, 467.2791, 551.2357, 599.3213, 656.8920, 701.4508, 725.3688, 747.4990, 793.4691	Loss of C_18_H_30_O_14_+Methylation [M-H]-	F

**Note:** RT.: retention time. **M1-M30**: detected metabolites. F: feces, U: urine, P: plasma.

## Data Availability

The original contributions presented in the study are included in the article/figures/supplementary material; further inquiries can be directed to the corresponding author.

## LIST OFABBREVIATIONS

ESI-MS: Electrospray Ionization-Mass Spectrometer
LC-MS: Liquid Chromatography Paired with Mass Spectrometry
UHPLC-Q-TOF-MS: Ultra-high Performance Liquid Chromatography Quadrupole Time of Flight Mass Spectrometer
BPC: Base Peak Chromatograms
EIC: Extracted ion Chromatograms
Glc: Glucose
Rha: Rhamnose
DI: Diagnostic Ion
NL: Neutral Loss
A: Aglycone
BPC: Base Peak Chromatogram
EIC: Extracted Ion Chromatogram
ESI-MS: Electro-Spray Ionization-Mass Spectrometry
Fr: Fraction
Glc: Glucose
Rha: Rhamnose
IDA: Information-Dependent Acquirement
IR: Infrared
MP: Metabolite Prediction
MDF: Mass Defect Filtering
NLF: Neutral Loss Filtering
HREIC: High Resolution Extracted Ion Chromatograms
NMR: Nuclear Magnetic Resonance
