# Supplementary material for: An Efficient Integrated Strategy for Comprehensive Metabolite Profiling of Sakurasosaponin from Aegiceras corniculatum in Rats
Source: Curr Drug Metab. 2024 Aug 6;25(5):340–54. doi: 10.2174/0113892002299923240801092101 (PMC11774310; doi:10.2174/0113892002299923240801092101)
Supplement: Supplementary file 1 [file CDM-25-5-340_SD1.pdf]

Supplementary Material

An Efficient Integrated Strategy for Comprehensive Metabolite Profiling of Sakurasosaponin from Aegiceras corniculatum in Rats

Xiangying Wang[1,#], Xiao Yang[2,#], Erwei Hao[2], Jinling Xie[2], Zhengcai Du[2], Jiagang Deng[2], Xiaotao Hou[1,*] and Wei Wei[2,*]

[1]Faculty of Pharmacy, Guangxi University of Chinese Medicine, Nanning, Guangxi 530200, China; [2]Guangxi Key Laboratory of Efficacy Study on Chinese Materia Medica, Guangxi University of Chinese Medicine, Nanning, Guangxi 530200, China

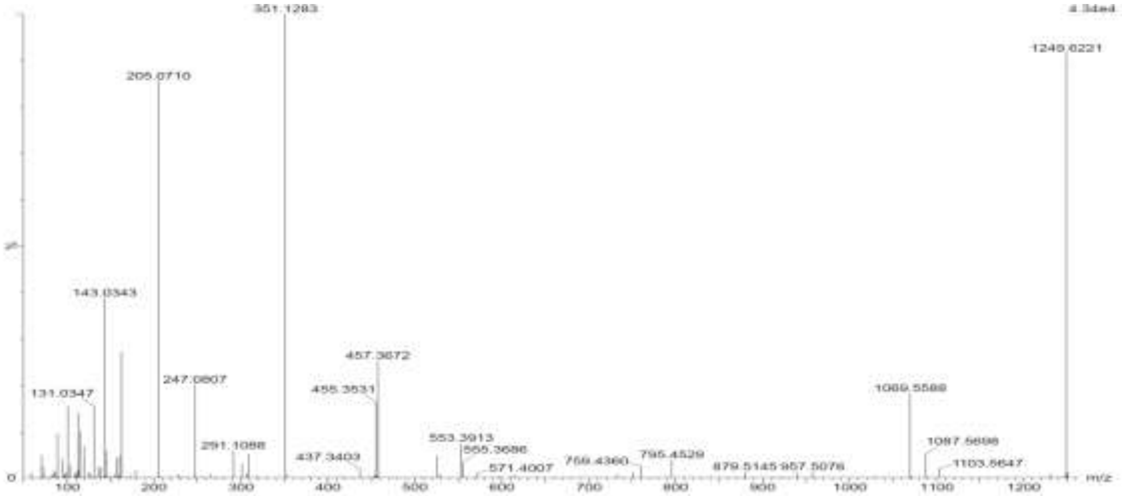

Fig.S1 The HR-ESI-MS spectrum of sakurasosaponin in negative ion mode.

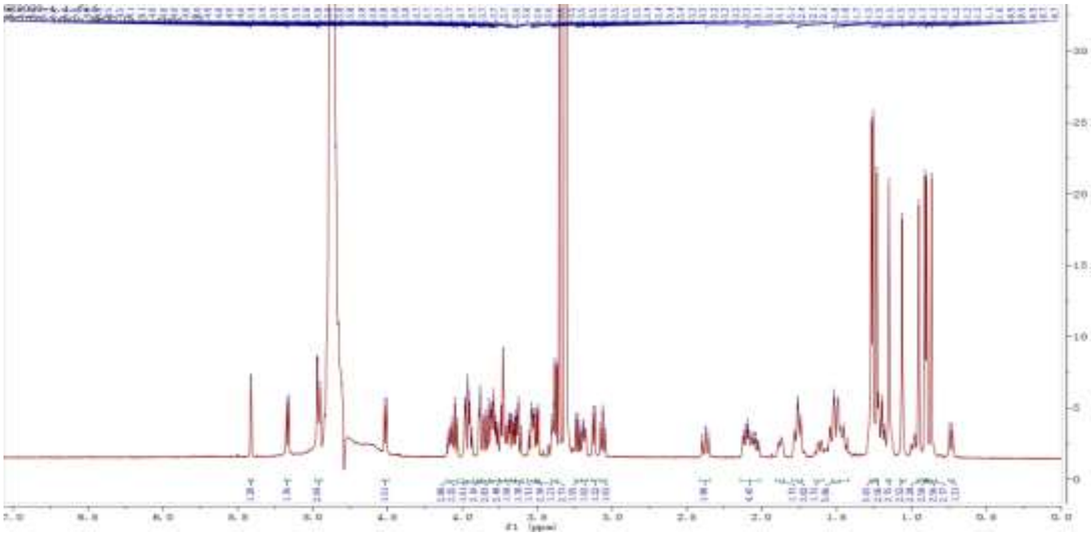

Fig.S2 The ^1H NMR spectrum of sakurasosaponin in MeOD (600 MHz).

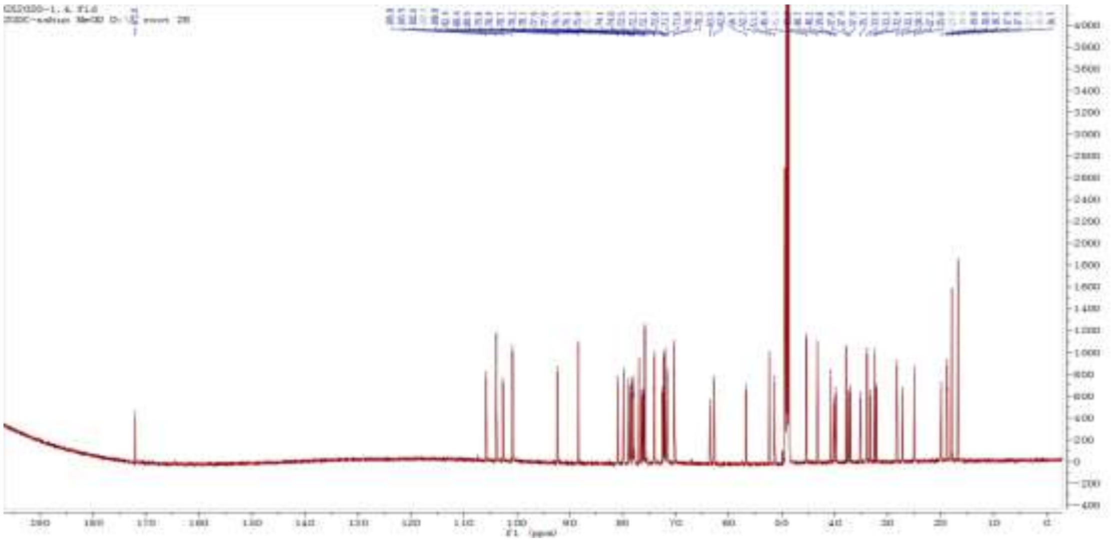

Fig.S3 The ^{13}C NMR spectrum of sakurasosaponin in MeOD (150 MHz).

Detail NMR data of sakurososaponin was as following. ^{1}H NMR (600 MHz, Methanol-d_4) δ 5.41 (d, *J* = 1.6 Hz, 1H), 5.17 (d, *J* = 7.6 Hz, 1H), 4.98 – 4.94 (m, 3H), 4.85 (s, 22H), 4.51 (d, *J* = 7.8 Hz, 1H), 4.12 – 4.02 (m, 2H), 3.98 (dd, *J* = 3.4, 1.7 Hz, 1H), 3.97 – 3.93 (m, 2H), 3.88 (t, *J* = 3.5 Hz, 2H), 3.86 – 3.81 (m, 2H), 3.81 – 3.76 (m, 2H), 3.71 (d, *J* = 3.2 Hz, 0H), 3.70 – 3.65 (m, 1H), 3.64 – 3.60 (m, 1H), 3.56 (s, 0H), 3.55 – 3.52 (m, 1H), 3.50 (d, *J* = 7.4 Hz, 1H), 3.39 (dd, *J* = 11.1, 7.6 Hz, 3H), 3.23 (dd, *J* = 9.4, 7.6 Hz, 1H), 3.21 – 3.17 (m, 1H), 3.12 (d, *J* = 7.5 Hz, 1H), 3.06 (t, *J* = 9.3 Hz, 1H), 2.38 (dd, *J* = 14.5, 11.9 Hz, 1H), 2.07 (ddt, *J* = 38.8, 13.8, 5.8 Hz, 3H), 1.76 (dd, *J* = 14.4, 10.7 Hz, 3H), 1.53 – 1.42 (m, 4H), 1.26 (d, *J* = 6.3 Hz, 7H), 1.23 (s, 3H), 1.19 (d, *J* = 8.5 Hz, 1H), 1.15 (s, 3H), 1.06 (s, 3H), 0.95 (s, 3H), 0.91 (s, 3H), 0.90 (s, 3H), 0.87 (s, 3H), 0.74 (d, *J*=11.9 Hz, 4H). ^{13}CNMR (151 MHz, MeOD): 40.2 (C-1), 27.2 (C-2), 92.4 (C-3), 40.70 (C-4), 56.7 (C-5), 18.7 (C-6), 35.1 (C-7), 43.3 (C-8), 51.3 (C-9), 37.8 (C-10), 19.9 (C-11), 32.2 (C-12), 88.4 (C-13), 45.3 (C-14), 37.0 (C-15), 78.1 (C-16), 45.4 (C-17), 52.4 (C-18), 39.8 (C-19), 32.4 (C-20), 37.4 (C-21), 33.3 (C-22), 28.3 (C-23), 16.8 (C-24), 16.7 (C-25), 18.8 (C-26), 19.9 (C-27), 78.7 (C-28), 33.9 (C-29), 25.0 (C-30), 172.0 (gluA-6), 105.8 (gluA-1), 103.9 (rha'-1), 102.6 (glu-1), 100.9 (rha-1), 100.8 (gal-1), 80.9 (gluA-2), 79.9 (rha-2), 78.9 (gluA-3), 78.2 (glu-3), 77.9 (glu-5), 77.0 (gal-5), 76.5 (gluA-5), 76.1 (glu-2), 75.8 (gal-2), 75.4 (gal-3), 74.1 (rha-4), 74.0 (rha-4'), 72.5 (gluA-4), 72.3 (glu-4), 72.1 (rha-3), 72.0 (rha'-2), 71.7 (rha'-3), 71.6 (gal-4), 70.3 (rha-5), 70.20 (rha'-5), 63.5 (glu-6), 62.8 (gal-6), 18.0 (rha-6), 17.90 (rha'-6). On the basis of above evidences and comparison its spectral data with those data reported in the literature, the parent drug (**M0**) was unambiguously identified as sakurasosaponin

Table S1 Characteristic NLs and corresponding DIs of sakurasosaponin in MS/MS spectrum.

DIs(m/z)	[M-H]	Formula	Error	NLs
101.0262	-1	C4H5O3	1.82	$C_{56}H_{92}O_{24}$
115.0408	-1	C5H7O3	0.69	$C_{55}H_{90}O_{24}$
131.0356	-1	C5H7O4	0.6	$C_{55}H_{90}O_{23}$
143.0358	-1	C6H7O4	0.86	$C_{54}H_{90}O_{23}$
163.063	-1	C6H11O5	1.82	$C_{54}H_{86}O_{22}$
205.0743	-1	C8H13O6	2.52	$C_{52}H_{84}O_{21}$
247.0852	-1	C10H15O7	2.88	$C_{50}H_{82}O_{20}$
309.1213	-1	C12H21O9	2.16	$C_{48}H_{76}O_{18}$
351.1342	-1	C14H23O10	4.54	$C_{46}H_{74}O_{17}$
455.358	-1	C30H47O3	4.96	$C_{30}H_{50}O_{24}$
457.3761	-1	C30H49O3	7.33	$C_{30}H_{48}O_{24}$
759.442	-1	C42H63O12	9.52	$C_{18}H_{34}O_{15}$
759.4595	-1	C39H67O14	5.83	$C_{21}H_{30}O_{13}$
1051.56	-1	C47H87O25	5.79	$C_{13}H_{10}O_{2}$
1069.5708	-1	C47H89O26	6.01	$C_{13}H_{8}O$

Table S2 Possible regular metabolic reactions for sakurasosaponin and the corresponding mass shift.

NO.	Name of the possible reaction	Mass shift
1	Hydrolysis of Nitrate Esters	-44.9851
2	Decarboxylation	-43.9898
3	Propyl Ketone to Acid	-40.0677
4	Loss of Hydroxymethylene	-30.0106
5	Nitro Reduction	-29.9742
6	Propyl Ether to Acid	-28.0677
7	Bis-Demethylation	-28.0313
8	Loss of CO	27 9949
9	Ethyl Ketone to Acid	-26.052
10	Loss of Water	-18.0106
11	Pyroglutamic acid formed from Gln	-17.0265
12	Demethylation and Desaturation	-16.0314
13	Sulfoxide to Thioether	-15.9949
14	Thioalcohol to Alcohol	-15.9772
15	Ethyl Ether to Acid	-14.052
16	Demethylation	-14.0157
17	Tert-Butyl to Acid	-12.0728
18	Ethyl to Alcohol	-12.0364
19	Demethylation and Hydrogenation	-12
20	Desaturation	-2.0157
21	Disulfide Bond	-2.0157
22	FomationDemethylation and ethylene to Ketone	0.0364
23	Parent	0
24	Oxidative Deamination to Alcohol	0.984
25	isopropyl to Acid	1.9429
26	Demethylation and Oixdation	1.9792
27	Hydrogenation	2.0157
28	Deethylation and Carbaxylic Acid Formation	3.9585
29	Ketone Formation	4.0313
30	Di.Hydrogenation	13.9793
31	Methylation	14.0157
32	Ethyl to Carboxylic Acid	15.9585
33	Oxidation	15.9949
34	Demethylation and Di.Oxidation	17.9742
35	internal Hydrolysis	18.0106
36	BiKetone Formation	27.9585
37	Formylation	27.9949
38	Amine to Carboxylic Acid	28.9789
39	Demethylation to Carbaxylic Acid	29.9742
40	Oxidation and Ketone Fo action	29.9742
41	Oxidation and Methylation	30.0106
42	Di.Oxidation	31.9898

43	Tri.Oxidation and Demethylation	33.9691
44	Oxidation and interna Hydrolysis	34.0055
45	N-Acetylation	42.0106
46	Acetylation	42.0106
47	Di.Oxidation and Ketone Formation	45.9691
48	Tri.Oxidation	47.9847
49	Tetra Oxidation and Demethylation	49.964
50	internal Hydrolysis and Di.Oxidation	50.0004
51	Glycine Conjugation	57.0215
52	Glycine (G)	57.0215
53	Tetra.Oxidation	63.9796
54	Alanine (A)	71.0371
55	Sulfate Conjugation	79.9568
56	Phosphorylation	79.9663
57	Di.Acetylation of Amines	84.0212
58	S-Cysteine Conjugation (Halide)	85.0431
59	Serine (S)	87.032
60	Oxidation and Sulfate Conjugation	95.9517
61	Proline (P)	97.0528
62	Valine (V)	99.0684
63	Threonine (T)	101.0477
64	Cysteine Conjugation	103.0092
65	Cysteine (C)	103.0092
66	Taurine Conjugation	107.0041
67	Isoleucine (I)	113.0841
68	Leucine (L)	113.0841
69	Isoleucine (I)	113.0841
70	Leucine (L)	113.0841
71	Asparagine (N)	114.0429
72	Aspartic Acid (D)	115.0269
73	Oxidation and Cysteine Conjugation	119.0041
74	S.Cysteine Conjugation	119.0041
75	Oxidation and Taurine Conjugation	122 9990
76	N-Acetylcysteine Conjugation (Hailide)	127.0536
77	Glutamine Conjugation	128.0586
78	Glutamine (O)	128.0586
79	Lysine (K)	128.095
80	Glutamic Acid (E)	129.0426
81	Methionine (M)	131.0405
82	Di.Oxidation and Cysteine Conjugation	134.999
83	Histidine (H)	137.0589
84	Di.Oxidation and Taurine Conjugation	138.9939
85	Oxidation and Glutamine Conjugation	144.0535

86	Phenylalanine (F)	147.0684
87	Arginine (R)	1,561,011
88	Bis-Sulfate Conjugation	159.9136
89	Bis-Phosphorylation	159.9327
90	N-Acetylcysteine Conjugation	161.0147
91	Demethylation and Glucuronide	162.0165
92	Glucose Conjugation	162.0528
93	Tyrosine (Y)	163.0633
94	Glucuronidation	176.0321
95	Tryptophan (W)	186.0793
96	Oxidation and Glucuronide Conjugation	192.027
97	Di.Oxidation and Glucuronide Conjugation	208.0219
98	Tri.Oxidation and Glucuronide	224.0168
99	Sulfate and Glucuronide Conjugation	255.9889
100	SGlutathione Conjugation (Halide)	271.1071
101	Oxidation and Sulfate and Glutathione Conjugation	271.9838
102	Glutathione Conjugation and Loss of H2S	273.0961
103	Glutathione Conjugation and Demethylation	289.0732
104	SGlutathione Conjugation and Demethylation	291.0525
105	Glutathione Conjugation and Demethylation	293.0682
106	Desaturation and SGlutathione	303.0525
107	SGlutathione Conjugation	305.0682
108	Glutathione Conjugation	307.0838
109	Oxidation and S.Glutathione Conjugation	321.0631
110	Glutathione Conjugation and Oxidation	323.0787
111	S.Glutathione Conjugation and Di-Oxidation	337.058
112	Methylation and Oxidation and S-Glutathione Conjugation	337.0944
113	Glutathione Conjugation and Di-Oxidation	339.0736
114	Bis.Glucuronide Conjugation	352.0642
115	Oxidation and Bis-glucuronide Conjugation	368.0591
116	Di.Oxidation and Bis.Glucuronidation	384.054
117	Tri.Oxidation and Bis.Glucuronidation	400.0489

Table S3 Compound special metabolites of sakurasosaponin that might generate *in vivo*.

No.	Loss from Parent	Neutral Formula	m/z [M-H]-	m/z [M+FA-H]-
1	$C_{48}H_{76}O_{19}$ and $C_6H_{10}O_5$	$C_6H_{12}O_3$	131.0714	177.0768
2	$C_{54}H_{86}O_{23}$	$C_6H_{12}O_4$	147.0663	193.0718
3	$C_{54}H_{86}O_{22}$	$C_6H_{12}O_5$	163.0612	209.0667
4	$C_{54}H_{86}O_{21}$	$C_6H_{12}O_6$	179.0561	225.0616
5	$C_{48}H_{76}O_{19}$ and O	$C_{12}H_{22}O_7$	277.1293	323.1348
6	$C_{48}H_{76}O_{19}$	$C_{12}H_{22}O_8$	293.1242	339.1297
7	$C_{48}H_{76}O_{18}$	$C_{12}H_{22}O_9$	309.1191	355.1246
8	$C_{30}H_{48}O_3$ and $C_{18}H_{30}O_{14}$	$C_{12}H_{20}O_{10}$	323.0984	369.1039
9	$C_{42}H_{66}O_{13}$ and $C_6H_{10}O_4$	$C_{12}H_{22}O_{10}$	325.114	371.1195

10	$C_{30}H_{48}O_2$ and $C_{18}H_{30}O_{14}$	$C_{12}H_{20}O_{11}$	339.0933	385.0988
11	$C_{30}H_{48}O_2$ and $C_{18}H_{30}O_{13}$	$C_{12}H_{20}O_{12}$	355.0882	401.0937
12	O and $C_{30}H_{48}O_{25}$	$C_{30}H_{50}O$	425.3789	471.3844
13	$C_{42}H_{66}O_{14}$ and O	$C_{18}H_{32}O_{12}$	439.1821	485.1876
14	$C_{30}H_{48}O_{25}$	$C_{30}H_{50}O_2$	441.3738	487.3793
15	$C_{42}H_{66}O_{14}$	$C_{18}H_{32}O_{13}$	455.177	501.1825
16	$C_{30}H_{48}O_{24}$	$C_{30}H_{50}O_3$	457.3687	503.3742
17	$C_{42}H_{66}O_{13}$	$C_{18}H_{32}O_{14}$	471.1719	517.1774
18	$C_{30}H_{48}O_3$ and $C_{12}H_{20}O_9$	$C_{18}H_{30}O_{15}$	485.1512	531.1567
19	$C_{30}H_{48}O_2$ and $C_{12}H_{20}O_9$	$C_{18}H_{30}O_{16}$	501.1461	547.1516
20	$C_{30}H_{48}O_2$ and $C_{12}H_{20}O_8$	$C_{18}H_{30}O_{17}$	517.141	563.1465
21	$C_6H_{10}O_6$ and $C_{18}H_{30}O_{14}$	$C_{36}H_{58}O_7$	601.411	647.4165
22	$C_{30}H_{48}O_3$ and $C_6H_{10}O_6$	$C_{24}H_{40}O_{18}$	615.2142	661.2197
23	$C_6H_{10}O_6$ and $C_{18}H_{30}O_{13}$	$C_{36}H_{58}O_8$	617.4059	663.4114
24	$C_{30}H_{48}O_2$ and $C_6H_{10}O_6$	$C_{24}H_{40}O_{19}$	631.2091	677.2146
25	$C_6H_{10}O_5$ and $C_{18}H_{30}O_{13}$	$C_{36}H_{58}O_9$	633.4008	679.4063
26	$C_{30}H_{48}O_2$ and $C_6H_{10}O_5$	$C_{24}H_{40}O_{20}$	647.204	693.2095
27	$C_{30}H_{48}O_2$ and $C_6H_{10}O_4$	$C_{24}H_{40}O_{21}$	663.1989	709.2044
28	O and $C_{18}H_{30}O_{14}$	$C_{42}H_{68}O_{12}$	763.4638	809.4693
29	$C_{30}H_{48}O_3$ and O	$C_{30}H_{50}O_{23}$	777.267	823.2725
30	$C_{18}H_{30}O_{14}$	$C_{42}H_{68}O_{13}$	779.4587	825.4642
31	$C_{30}H_{48}O_3$	$C_{30}H_{50}O_{24}$	793.2619	839.2674
32	$C_{18}H_{30}O_{13}$	$C_{42}H_{68}O_{14}$	795.4536	841.4591
33	$C_{30}H_{48}O_2$	$C_{30}H_{50}O_{25}$	809.2568	855.2623
34	$C_6H_{10}O_6$ and $C_6H_{10}O_5$	$C_{48}H_{78}O_{16}$	909.5217	955.5272
35	O and $C_{12}H_{20}O_9$	$C_{48}H_{78}O_{17}$	925.5166	971.5221
36	$C_{12}H_{20}O_9$	$C_{48}H_{78}O_{18}$	941.5115	987.517
37	$C_{12}H_{20}O_8$	$C_{48}H_{78}O_{19}$	957.5065	1003.5119
38	O and $C_6H_{10}O_6$	$C_{54}H_{88}O_{20}$	1055.5796	1101.5851
39	$C_6H_{10}O_6$	$C_{54}H_{88}O_{21}$	1071.5745	1117.58
40	$C_6H_{10}O_5$	$C_{54}H_{88}O_{22}$	1087.5694	1133.5749
41	$C_6H_{10}O_4$	$C_{54}H_{88}O_{23}$	1103.5644	1149.5698
42	O and O	$C_{60}H_{98}O_{25}$	1217.6324	1263.6379
43	O	$C_{60}H_{98}O_{26}$	1233.6274	1279.6328
44	H_2O	$C_{60}H_{100}O_{26}$	1235.643	1281.6485

Interpretation of the metabolite in rat (M1)

M1 – Loss of C12H20O8+Oxidation [M-H]-

Formula:	C48H78O20
ppm:	7.3
RDB:	10.0

Available Structure Candidates

Rank	Score	Count
1	100.0	24

Applied Metabolite Structure

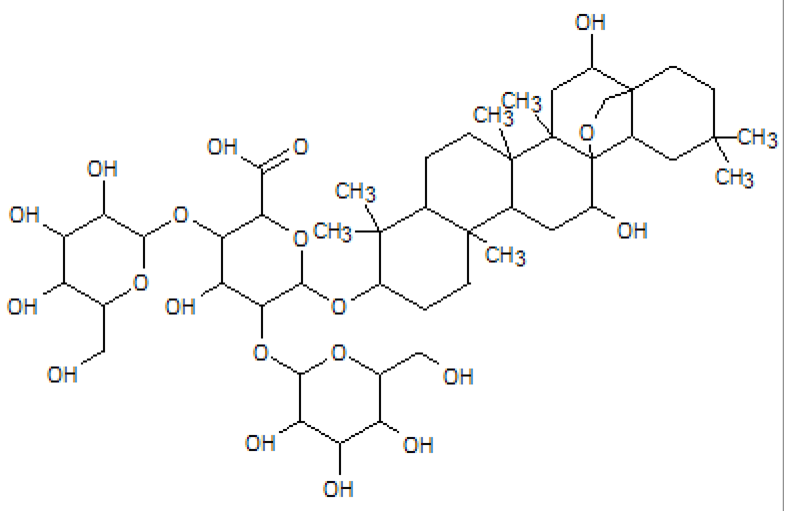

Composition:	C48H78O20
Mass:	974.5086

Metabolite MS/MS

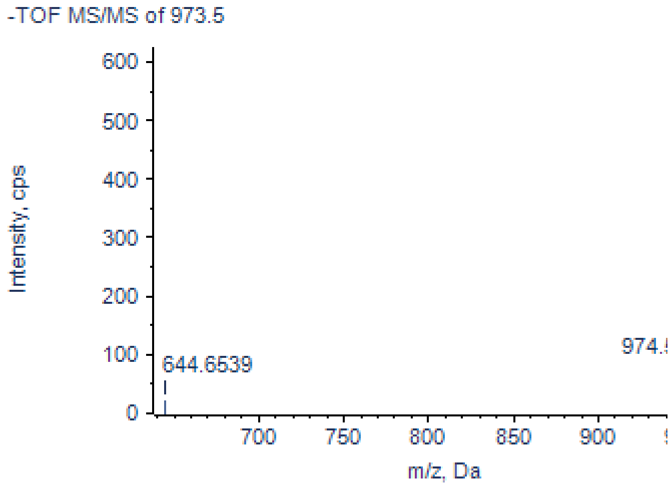

Parent MS/MS

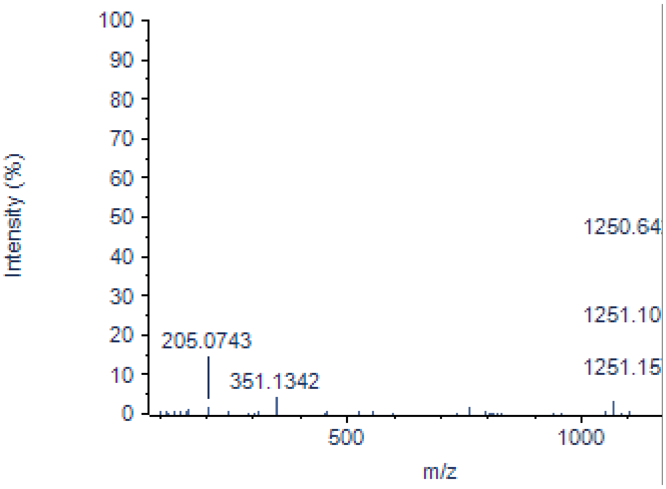

Peaks selected for assignment (m/z): 644.6539, 955.5069, 973.3659, 973.4184, 973.4475, 973.5115, 973.5575

Metabolite Options

Number of fragment peaks selected for assignment:	30
Minimum signal-to-noise ratio:	3
MS/MS m/z tolerance:	15 ppm
Fragmentation Settings	
Break aromatic rings:	True
Maximum number of bonds to break:	4
Maximum number of C-C bonds to break:	4
Label Settings	
Label peaks with:	Ion with ppm Error

Fragment Filters

Fragments with assigned structures	True

Fragment Details	Structure Details
Mass (m/z): 973.5115<br>Ion Formula: C48H77O20<br>Error (ppm): 10.4<br>Intensity (cps): 624.0<br>RDB: 10.0<br>Score: 44.0	

<table>
<tr><th>Fragment Details</th><th>Structure Details</th></tr>
<tr><td></td><td>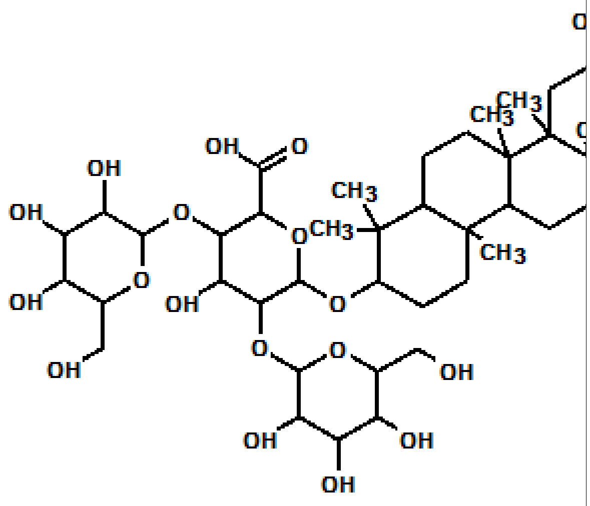
Se-lected: C48H78O20
Mass: 974.5086
Broken Bonds: 0
Delta H: 0
Score: 44.0
Contained Neutral Losses
No contained neutral losses</td></tr>
</table>

Interpretation of the metabolite in rat (M2)

M2 – Isomer of Parent [M-H]-

Formula:	C60H98O27
ppm:	8.4
RDB:	12.0

Available Structure Candidates

Rank	Score	Count
1	100.0	1

Applied Metabolite Structure

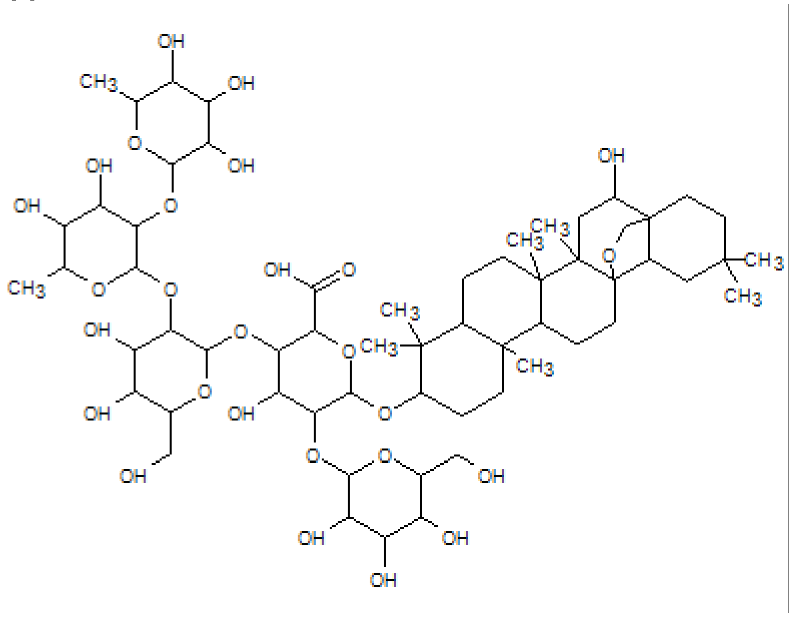

Composition:	C60H98O27
Mass:	1250.6295

Metabolite MS/MS

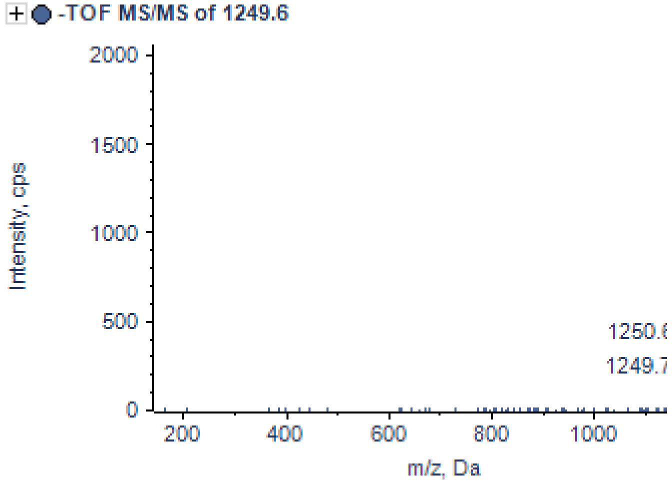

Parent MS/MS

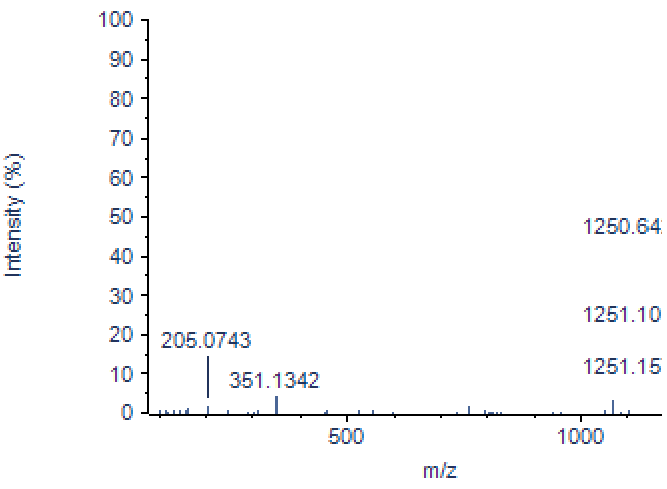

Common product ions (m/z): 1249.6351, 1250.5592, 1250.6393

Peaks selected for assignment (m/z): 163.0619, 205.0735, 365.1182, 365.2372, 384.9487, 398.8678, 425.1299, 445.1563, 481.1906, 620.9673, 624.3121, 624.8094, 645.3482, 658.0536, 671.2028, 671.4936, 678.1662, 728.4814, 875.2764, 923.5005, 937.0390, 1187.6412, 1203.6421, 1211.7120, 1231.6314, 1245.3775, 1245.4514, 1245.5212, 1249.3025, 1249.6351

Metabolite Options

Number of fragment peaks selected for assignment:	30
Minimum signal-to-noise ratio:	3
MS/MS m/z tolerance:	15 ppm
Fragmentation Settings	
Break aromatic rings:	True
Maximum number of bonds to break:	4

Maximum number of C-C bonds to break: 4

Label Settings

Label peaks with: Ion with ppm Error

Fragment Filters

None

Fragment Details	Structure Details
Mass (m/z): 1249.6351<br>Ion Formula: C60H97O27<br>Error (ppm): 10.2<br>Intensity (cps): 2055.9<br>RDB: 12.0<br>Score: 44.0	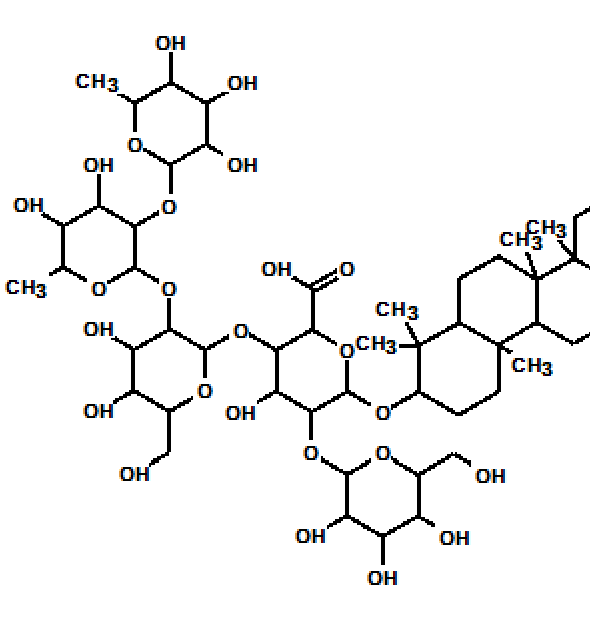<br>Selected: C60H98O27<br>Mass: 1250.6295<br>Broken Bonds: 0<br>Delta H: 0<br>Score: 44.0<br>**Contained Neutral Losses**<br>No contained neutral losses

Interpretation of the metabolite in rat (M3)

M3 – Loss of C12H20O8+Glucose Conjugation [M-H]-

Formula: C54H88O24

ppm: 7.9

RDB: 11.0

Available Structure Candidates

Rank	Score	Count
1	100.0	11

Applied Metabolite Structure

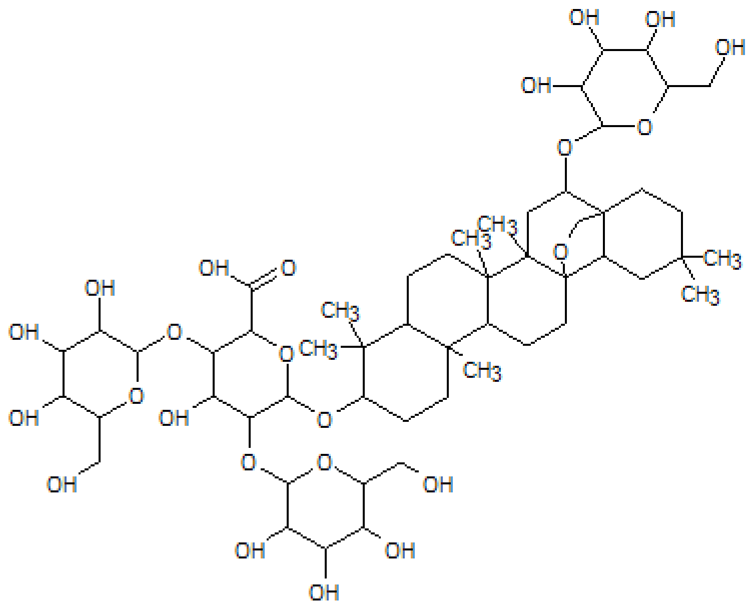

Composition:	C54H88O24
Mass:	1120.5666

Metabolite MS/MS

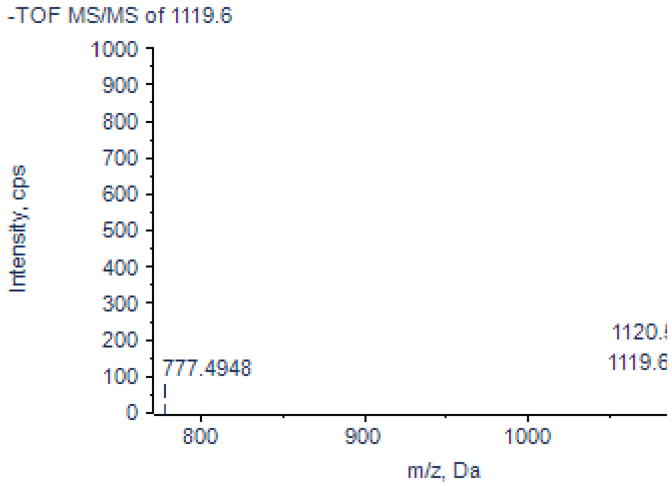

Parent MS/MS

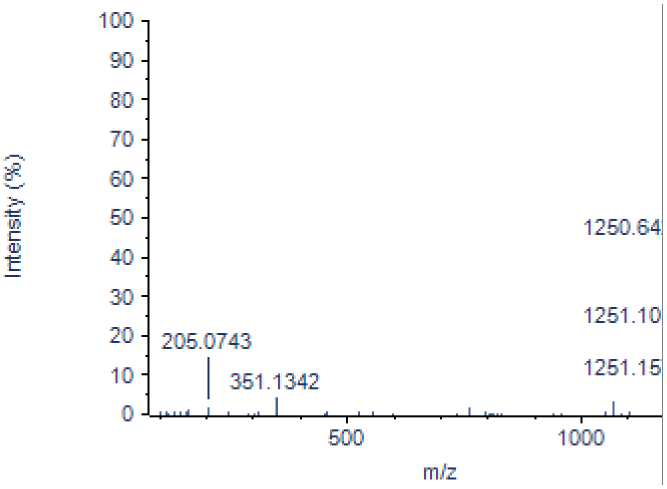

Peaks selected for assignment (m/z): 777.4948, 1119.4503, 1119.4817, 1119.5705, 1119.6264, 1119.6556

Metabolite Options

Number of fragment peaks selected for assignment:	30
Minimum signal-to-noise ratio:	3
MS/MS m/z tolerance:	15 ppm
Fragmentation Settings	
Break aromatic rings:	True
Maximum number of bonds to break:	4
Maximum number of C-C bonds to break:	4
Label Settings	
Label peaks with:	Ion with ppm Error

Fragment Filters

Fragments with assigned structures	True

<table>
<tr><th>Fragment Details</th><th>Structure Details</th></tr>
<tr><td>Mass (m/z): 1119.5705
Ion Formula: C54H87O24
Error (ppm): 10.0
Intensity (cps): 1001.0
RDB: 11.0
Score: 44.0</td><td>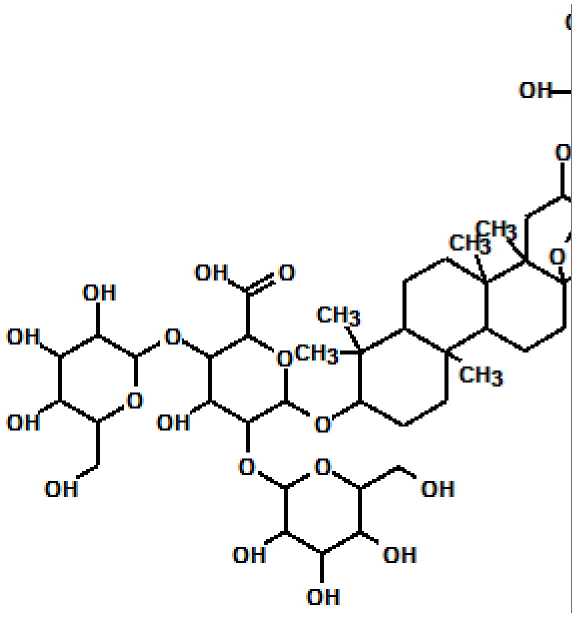
Selected: C54H88O24
Mass: 1120.5666
Broken Bonds: 0
Delta H: 0
Score: 44.0
Contained Neutral Losses
No contained neutral losses</td></tr>
</table>

Interpretation of the metabolite in rat (M4)

M4 – Loss of C18H30O13+Oxidation [M-H]-

Formula:	C42H68O15
ppm:	7.7
RDB:	9.0

Available Structure Candidates

Rank	Score	Count
1	100.0	44

Applied Metabolite Structure

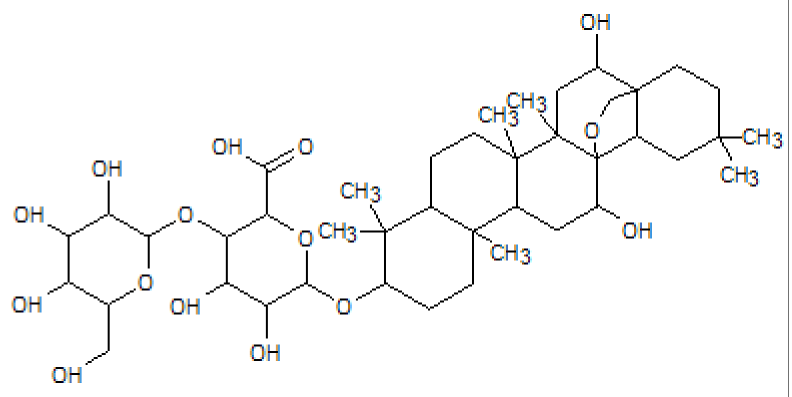

Composi-tion:	C42H68O15
Mass:	812.4558

Metabolite MS/MS

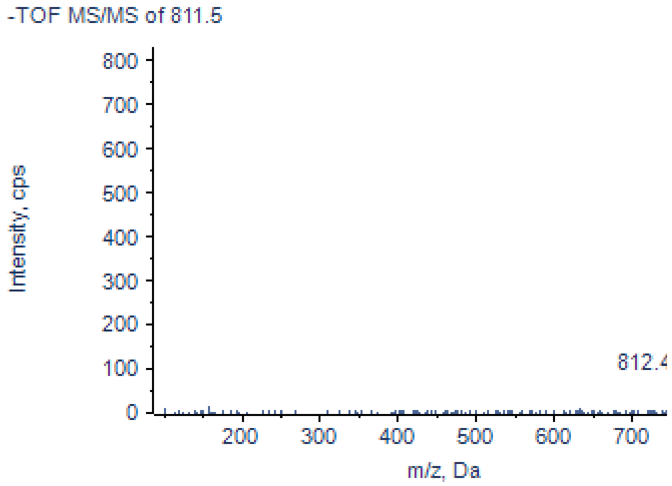

Parent MS/MS

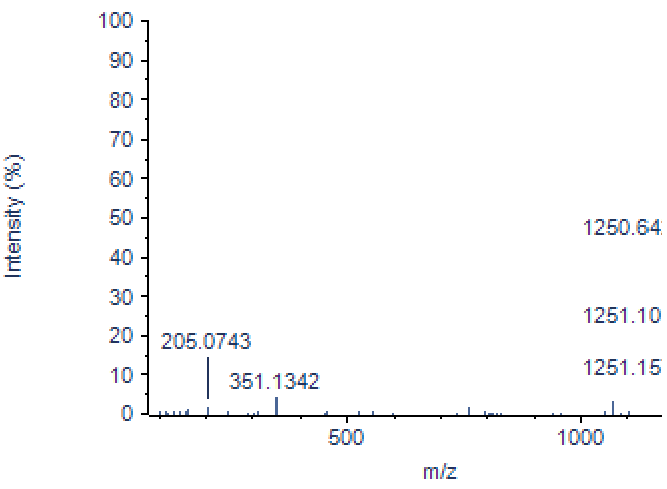

Peaks selected for assignment (m/z): 101.0249, 139.0053, 149.0521, 157.0167, 176.0231, 186.8594, 193.0365, 235.0582, 251.1295, 269.0648, 309.9108, 336.8844, 336.9931, 366.0604, 396.3177, 400.9102, 402.8911, 406.2117, 436.8196, 441.9106, 447.1261, 473.3870, 475.0693, 492.1629, 513.2119, 541.4244, 543.8944, 631.3923, 811.3939, 811.4573

Metabolite Options

Number of fragment peaks selected for assign-ment:	30
Minimum signal-to-noise ratio:	3
MS/MS m/z tolerance:	15 ppm
Fragmentation Settings	
Break aromatic rings:	True
Maximum number of bonds to break:	4
Maximum number of C-C bonds to break:	4

Label Settings

Label peaks with: Ion with ppm Error

Fragment Filters

Fragments with assigned structures True

<table>
<tr><th>Fragment Details</th><th>Structure Details</th></tr>
<tr><td>Mass (m/z): 631.3923
Ion Formula: C36H55O9
Error (ppm): 11.4
Intensity (cps): 12.6
RDB: 9.0
Score: 40.0</td><td>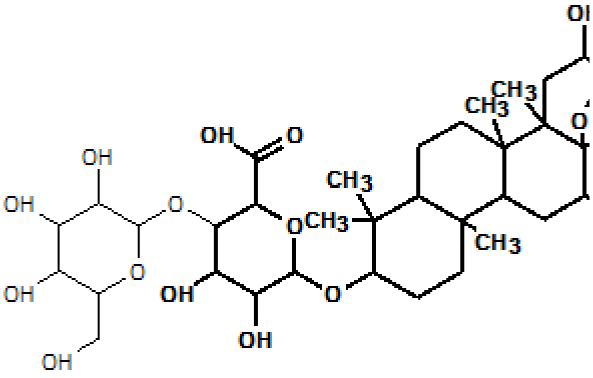

Selected: C36H57O9
Mass: 633.4003
Broken Bonds: 1
Delta H: -2
Score: 40.0

Contained Neutral Losses
No contained neutral losses</td></tr>
<tr><td>Mass (m/z): 811.4573
Ion Formula: C42H67O15
Error (ppm): 10.8
Intensity (cps): 829.4
RDB: 9.0
Score: 44.0</td><td>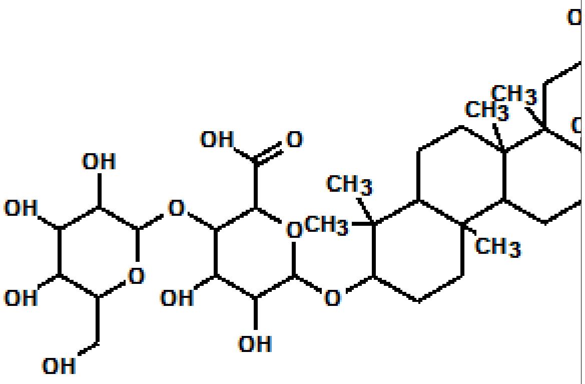

Selected: C42H68O15
Mass: 812.4558
Broken Bonds: 0
Delta H: 0
Score: 44.0</td></tr>
</table>

<table>
<tr><th>Fragment Details</th><th>Structure Details</th></tr>
<tr><td></td><td>Contained Neutral Losses<table><tr><th>Mass</th><th>Formula</th></tr><tr><td>180.0650</td><td>C6H12O6</td></tr></table></td></tr>
</table>

Interpretation of the metabolite in rat (M5)

M5 – Loss of C12H20O8 [M-H]-

Formula:	C48H78O19
ppm:	6.2
RDB:	10.0

Available Structure Candidates

Rank	Score	Count
1	100.0	1

Applied Metabolite Structure

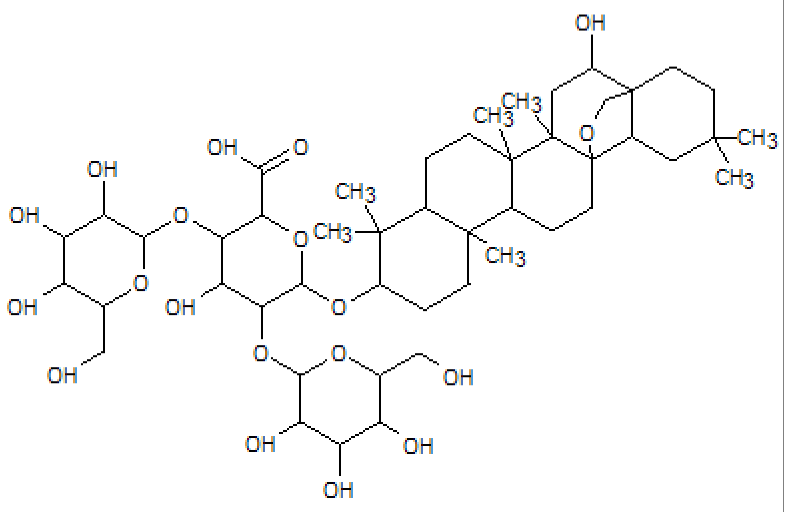

Composition:	C48H78O19
Mass:	958.5137

Metabolite MS/MS

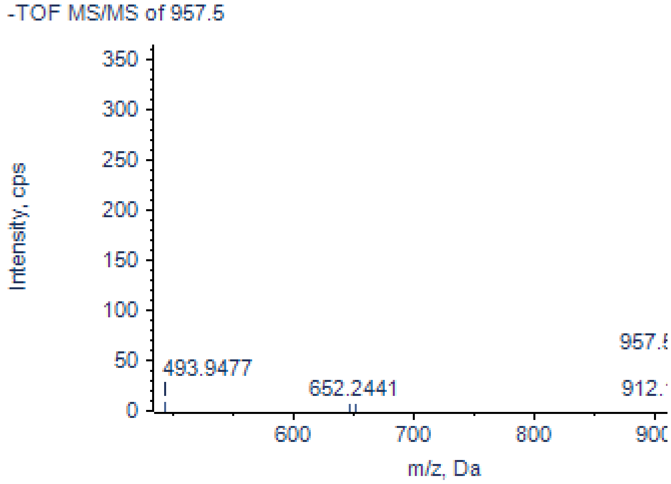

Parent MS/MS

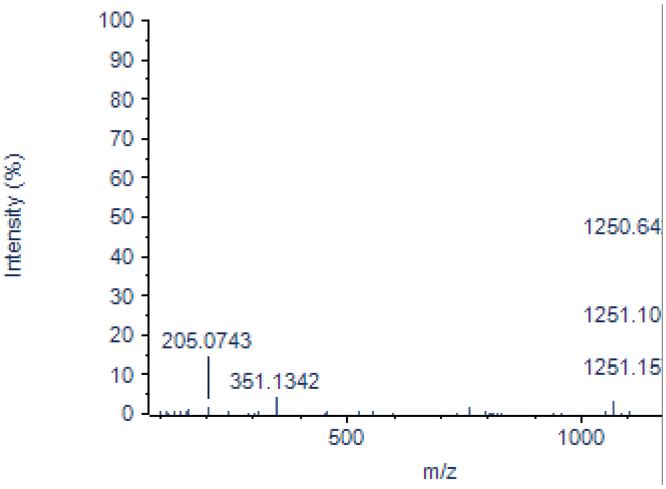

Peaks selected for assignment (m/z): 493.9477, 647.7495, 652.2441, 912.1525, 957.2874, 957.4511, 957.5163

Metabolite Options

Number of fragment peaks selected for assignment:	30
Minimum signal-to-noise ratio:	3
MS/MS m/z tolerance:	15 ppm
Fragmentation Settings	
Break aromatic rings:	True
Maximum number of bonds to break:	4
Maximum number of C-C bonds to break:	4
Label Settings	
Label peaks with:	Ion with ppm Error

Fragment Filters

Fragments with assigned structures	True

Fragment Details		Structure Details
Mass (m/z):	957.5163	
Ion Formula:	C48H77O19	
Error (ppm):	10.3	
Intensity (cps):	363.0	
RDB:	10.0	
Score:	44.0	

<table>
<tr><th>Fragment Details</th><th>Structure Details</th></tr>
<tr><td></td><td>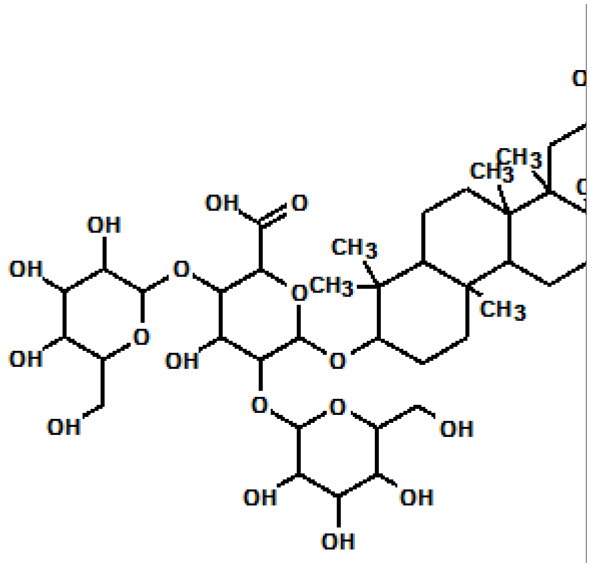
Se-lected: C48H78O19
Mass: 958.5137
Broken Bonds: 0
Delta H: 0
Score: 44.0
Contained Neutral Losses
No contained neutral losses</td></tr>
</table>

Interpretation of the metabolite in rat (M6)

M6 – Loss of O and C12H20O9+Demethylation and Methylene to Ketone [M-H]-

Formula:	C47H74O18
ppm:	7.7
RDB:	11.0

Available Structure Candidates

Rank	Score	Count
1	100.0	1
2	0.0	3

Applied Metabolite Structure

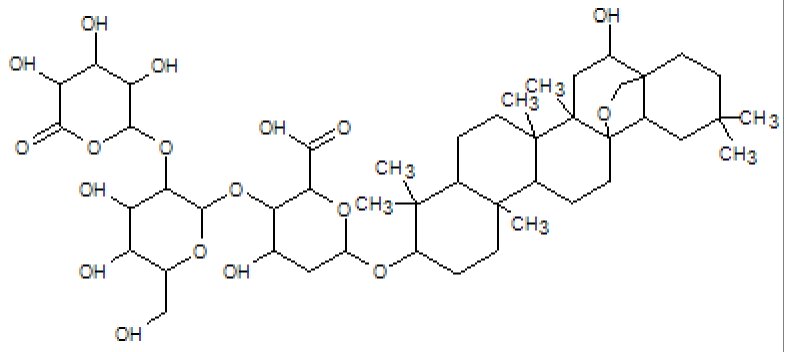

Composi-tion:	C47H74O18
Mass:	926.4875

Metabolite MS/MS

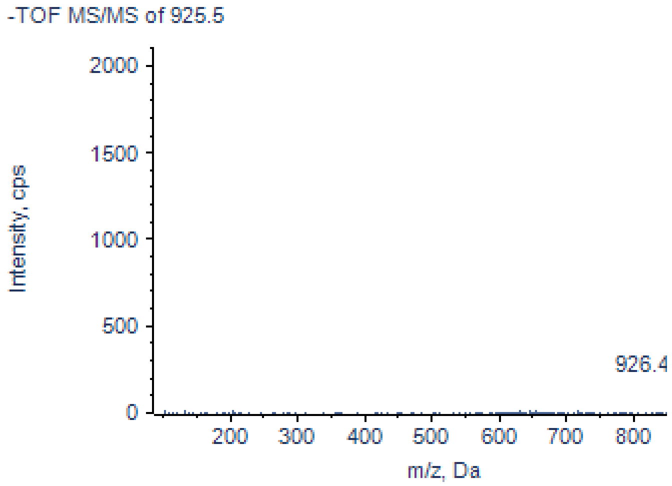

Parent MS/MS

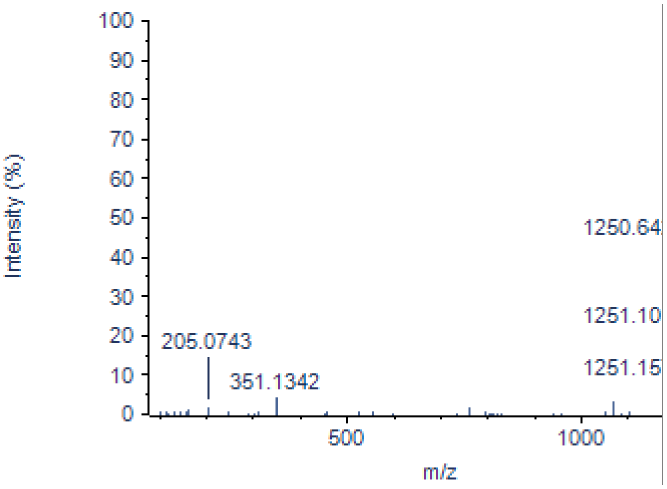

Peaks selected for assignment (m/z): 102.4302, 102.4395, 103.0436, 115.0404, 131.0361, 145.0514, 163.0616, 163.0807, 166.0019, 181.4312, 189.7950, 199.3941, 205.0740, 214.3614, 214.3883, 268.0867, 286.9374, 295.1048, 310.9522, 312.2168, 355.2198, 359.2704, 471.3640, 629.3727, 629.3879, 646.2919, 654.8217, 717.4314, 863.4913, 925.4900

Metabolite Options

Number of fragment peaks selected for assign-ment:	30
Minimum signal-to-noise ratio:	3
MS/MS m/z tolerance:	15 ppm
Fragmentation Settings	
Break aromatic rings:	True
Maximum number of bonds to break:	4
Maximum number of C-C bonds to break:	4

Label Settings

Label peaks with: Ion with ppm Error

Fragment Filters

Fragments with assigned structures True

Fragment Details	Structure Details
Mass (m/z): 205.0740<br>Ion Formula: C8H13O6<br>Error (ppm): 11.1<br>Intensity (cps): 20.1<br>RDB: 2.0<br>Score: 29.5	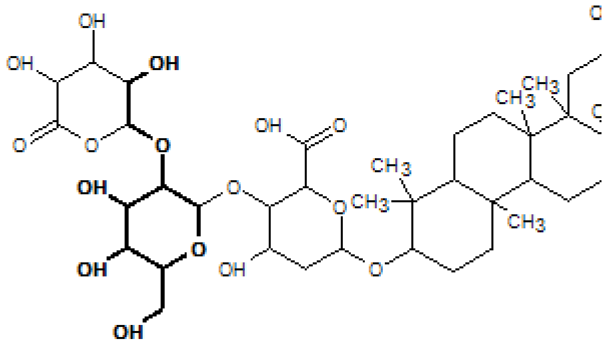<br>Selected: C8H13O6<br>Mass: 205.0712<br>Broken Bonds: 3<br>Delta H: -2<br>Score: 29.5
Mass (m/z): 629.3727<br>Ion Formula: C36H53O9<br>Error (ppm): 5.1<br>Intensity (cps): 14.7<br>RDB: 10.0<br>Score: 25.0	

Contained Neutral Losses

Mass	Formula
42.0125	C2H2O

<table>
<tr><th>Fragment Details</th><th>Structure Details</th></tr>
<tr><td></td><td>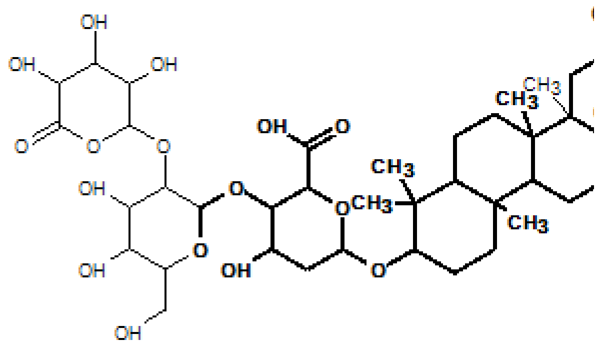
Se-lected: C36H55O9
Mass: 631.3846
Broken Bonds: 3
Delta H: -4
Score: 25.0
Contained Neutral Losses
No contained neutral losses</td></tr>
<tr><td>Mass (m/z): 717.4314
Ion Formula: C40H61O11
Error (ppm): 13.2
Intensity (cps): 9.2
RDB: 10.0
Score: 29.5</td><td>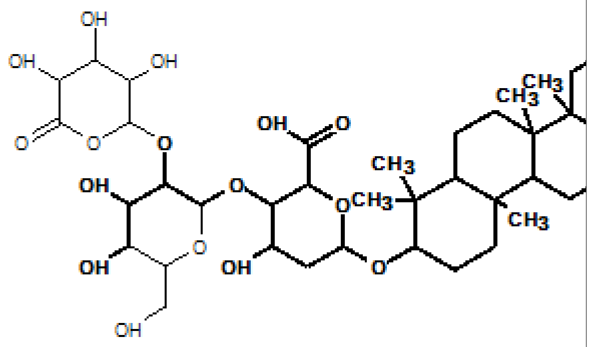
Se-lected: C40H63O11
Mass: 719.4370
Broken Bonds: 3
Delta H: -4
Score: 29.5
Contained Neutral Losses
No contained neutral losses</td></tr>
<tr><td>Mass (m/z): 863.4913
Ion Formula: C46H71O15
Error (ppm): 13.3
Intensity (cps): 13.2</td><td></td></tr>
</table>

Fragment Details	Structure Details
RDB: 11.0<br>Score: 33.5	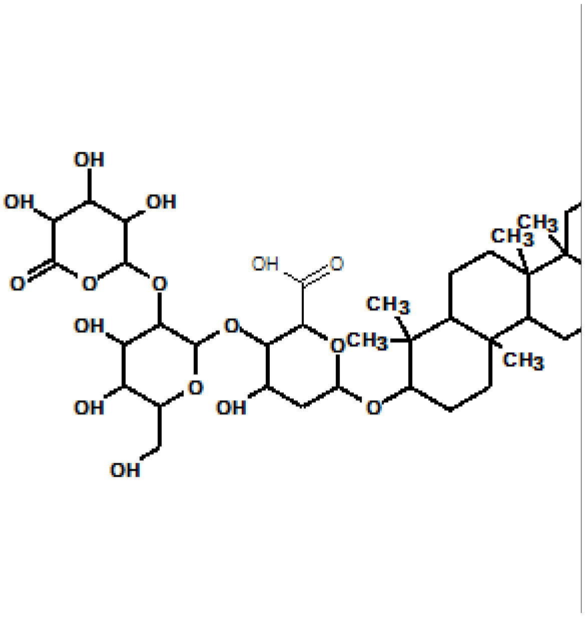<br>Selected: C46H72O15<br>Mass: 864.4871<br>Broken Bonds: 2<br>Delta H: -2<br>Score: 33.5<br>**Contained Neutral Losses**<br>No contained neutral losses
Mass (m/z): 925.4900<br>Ion Formula: C47H73O18<br>Error (ppm): 10.5<br>Intensity (cps): 2102.7<br>RDB: 11.0<br>Score: 44.0	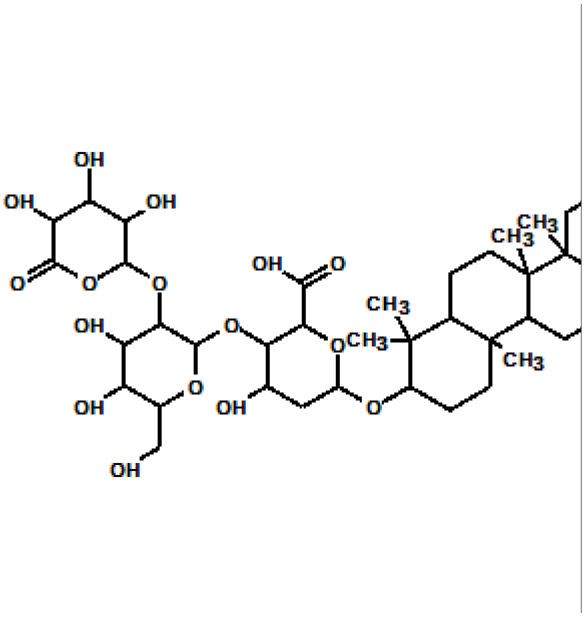<br>Selected: C47H74O18<br>Mass: 926.4875<br>Broken Bonds: 0<br>Delta H: 0<br>Score: 44.0<br>**Contained Neutral Losses**<br>No contained neutral losses

Interpretation of the metabolite in rat (M7)

M7 – Loss of C18H30O14+Demethylation to Carboxylic Acid [M-H]-

Formula:	C42H66O15
ppm:	7.2
RDB:	10.0

Available Structure Candidates

Rank	Score	Count
1	100.0	25
2	0.0	26

Applied Metabolite Structure

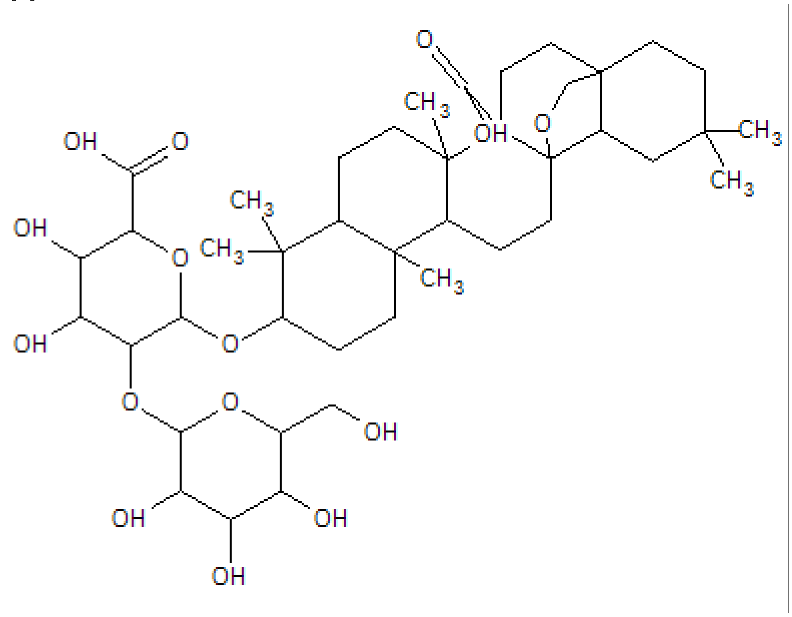

Composi-tion:	C42H66O15
Mass:	810.4402

Metabolite MS/MS

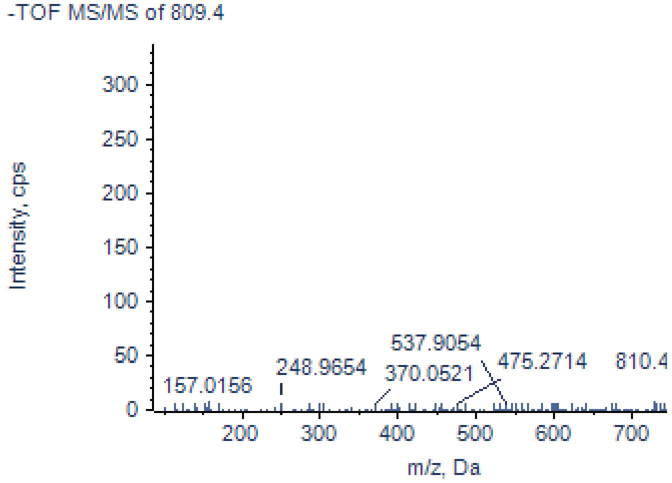

Parent MS/MS

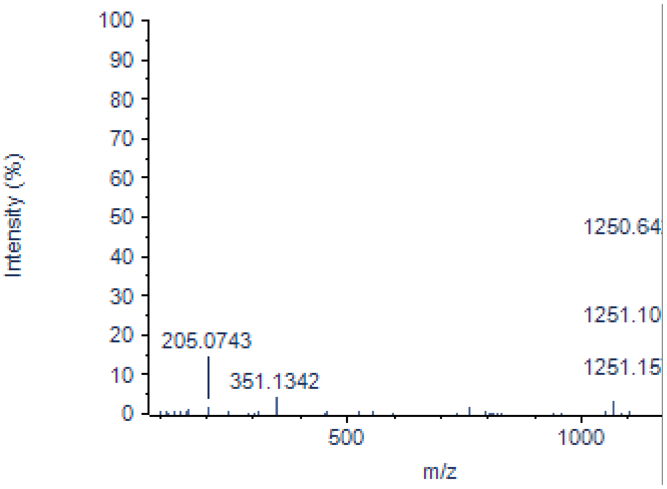

Peaks selected for assignment (m/z): 113.0261, 113.0358, 122.9870, 139.0055, 152.9835, 157.0156, 170.8728, 248.9654, 285.0264, 299.1016, 304.9150, 370.0521, 390.3086, 399.1974, 415.1412, 422.1759, 448.8126, 456.0474, 475.2714, 485.0434, 521.1603, 530.1119, 537.9054, 544.0249, 549.3722, 557.2578, 557.3012, 564.6369, 727.2027, 809.4423

Metabolite Options

Number of fragment peaks selected for assignment:	30
Minimum signal-to-noise ratio:	3
MS/MS m/z tolerance:	15 ppm
Fragmentation Settings	
Break aromatic rings:	True
Maximum number of bonds to break:	4
Maximum number of C-C bonds to break:	4
Label Settings	
Label peaks with:	Ion with ppm Error

Fragment Filters

Fragments with assigned structures	True

Fragment Details	Structure Details
Mass (m/z): 285.0264<br>Ion Formula: C11H9O9<br>Error (ppm): 4.3<br>Intensity (cps): 6.1<br>RDB: 7.0<br>Score: 26.0	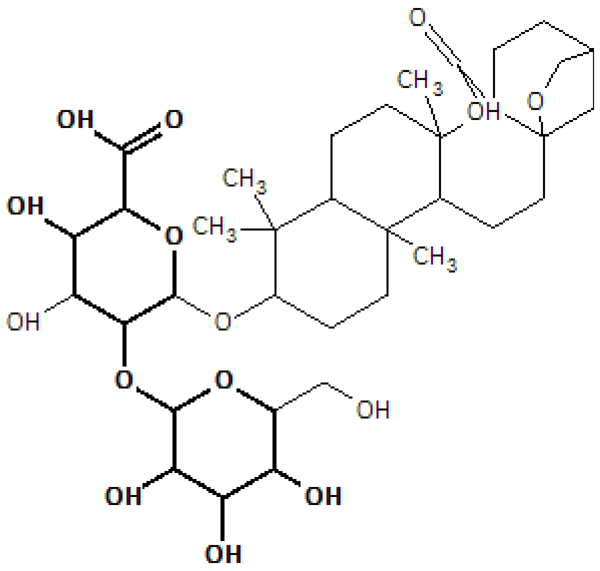<br>Selected: C11H15O9<br>Mass: 291.0716<br>Broken Bonds: 3<br>Delta H: -8<br>Score: 26.0

Contained Neutral Losses

Mass	Formula
146.0210	C5H6O5
132.0429	C5H8O4
128.0109	C5H4O4

Fragment Details	Structure Details
Mass (m/z): 299.1016<br>Ion Formula: C10H19O10<br>Error (ppm): 10.7<br>Intensity (cps): 6.1<br>RDB: 1.0<br>Score: 29.5	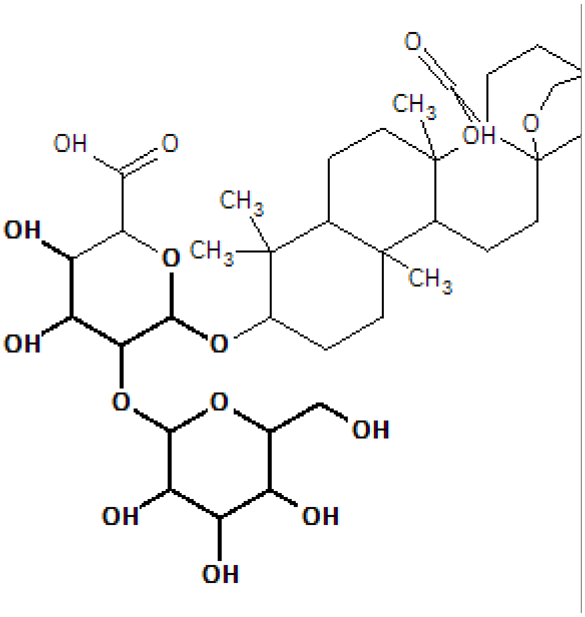<br>Selected: C10H17O10<br>Mass: 297.0822<br>Broken Bonds: 3<br>Delta H: 0<br>Score: 29.5<br>**Contained Neutral Losses**<br>No contained neutral losses
Mass (m/z): 390.3086<br>Ion Formula: C25H42O3<br>Error (ppm): -13.8<br>Intensity (cps): 6.0<br>RDB: 4.5<br>Score: 26.0	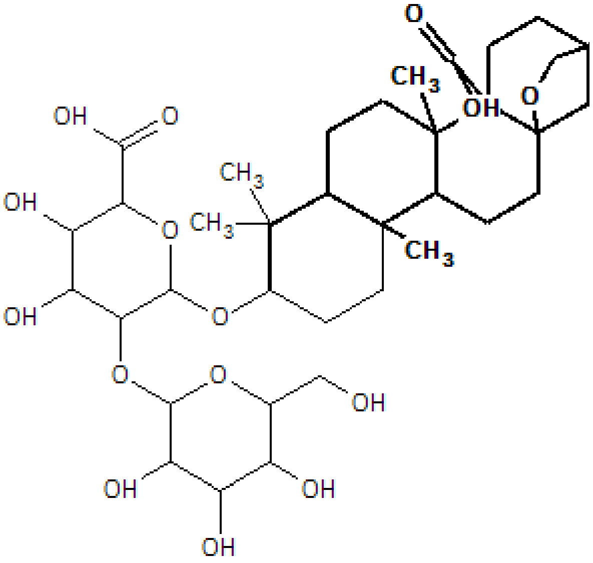<br>Selected: C25H34O3<br>Mass: 382.2508<br>Broken Bonds: 2<br>Delta H: 2<br>Score: 26.0<br>**Contained Neutral Losses**<br>No contained neutral losses
Mass (m/z): 422.1759<br>Ion Formula: C18H30O11	

<table>
<tr><th>Fragment Details</th><th>Structure Details</th></tr>
<tr><td>Error (ppm): -8.1
Intensity (cps): 5.9
RDB: 3.5
Score: 22.0</td><td>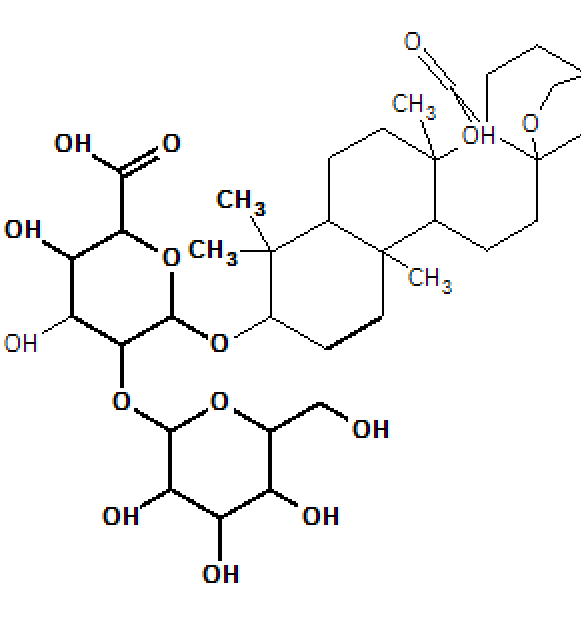
Selected: C18H33O11
Mass: 425.2023
Broken Bonds: 3
Delta H: -2
Score: 22.0
Contained Neutral Losses
No contained neutral losses</td></tr>
<tr><td>Mass (m/z): 549.3722
Ion Formula: C32H53O7
Error (ppm): -13.7
Intensity (cps): 5.8
RDB: 6.0
Score: 25.0</td><td>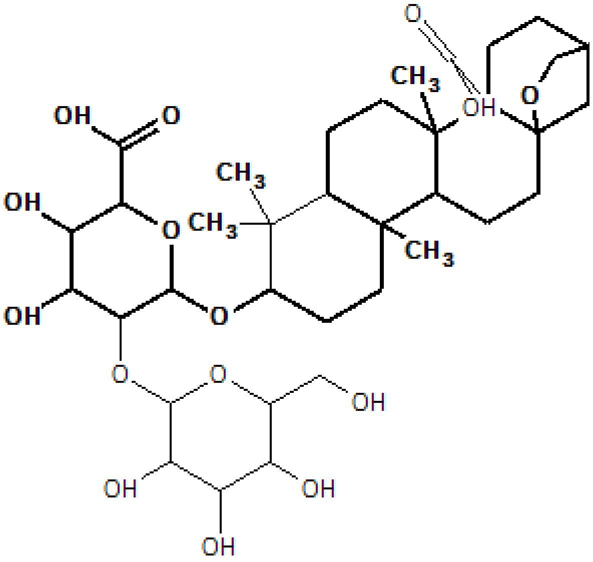
Selected: C32H49O7
Mass: 545.3478
Broken Bonds: 3
Delta H: 2
Score: 25.0
Contained Neutral Losses<table><tr><th>Mass</th><th>Formula</th></tr><tr><td>392.3566</td><td>C26H48O2</td></tr></table></td></tr>
<tr><td>Mass (m/z): 557.3012
Ion Formula: C28H45O11</td><td></td></tr>
</table>

<table>
<tr><th>Fragment Details</th><th>Structure Details</th></tr>
<tr><td>Error (ppm): 8.0
Intensity (cps): 5.8
RDB: 6.0
Score: 25.0</td><td>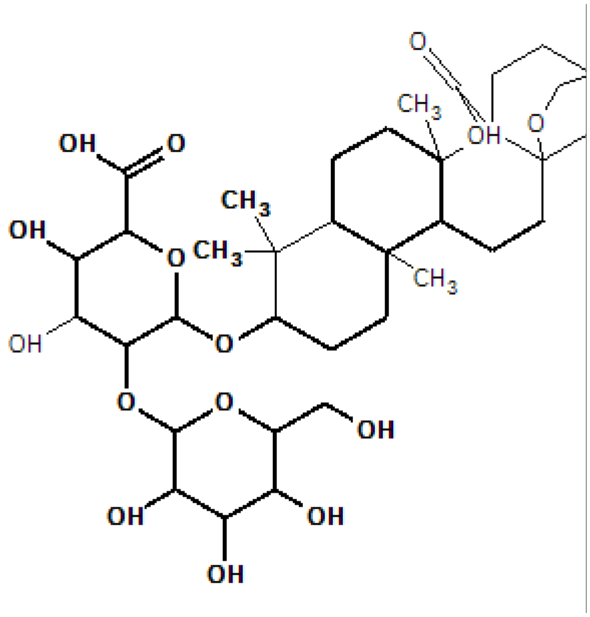
Selected: C28H44O11
Mass: 556.2884
Broken Bonds: 3
Delta H: -2
Score: 25.0

Contained Neutral Losses
<table><tr><th>Mass</th><th>Formula</th></tr><tr><td>404.3176</td><td>C22H44O6</td></tr><tr><td>400.2856</td><td>C22H40O6</td></tr></table></td></tr>
<tr><td>Mass (m/z): 809.4423
Ion Formula: C42H65O15
Error (ppm): 11.7
Intensity (cps): 336.7
RDB: 10.0
Score: 44.0</td><td>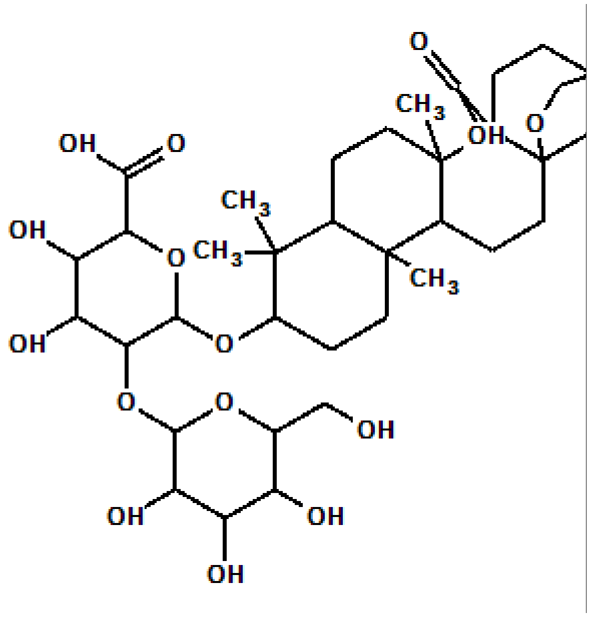
Selected: C42H66O15
Mass: 810.4402
Broken Bonds: 0
Delta H: 0
Score: 44.0

Contained Neutral Losses
No contained neutral losses</td></tr>
</table>

Interpretation of the metabolite in rat (M8)

M8 – Isomer of Parent [M-H]-

Formula:	C60H98O27
ppm:	5.5
RDB:	12.0

Applied Metabolite Structure

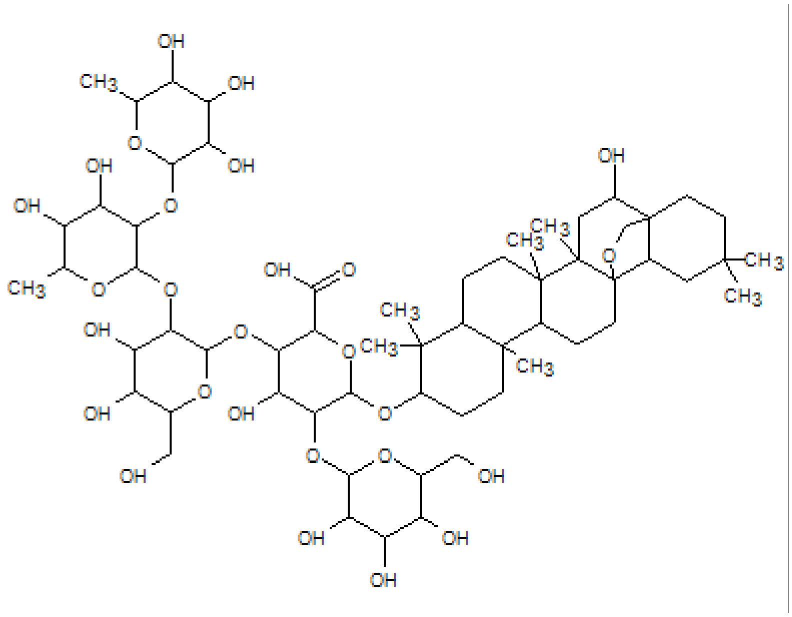

Composition:	C60H98O27
Mass:	1250.6295

Metabolite MS/MS

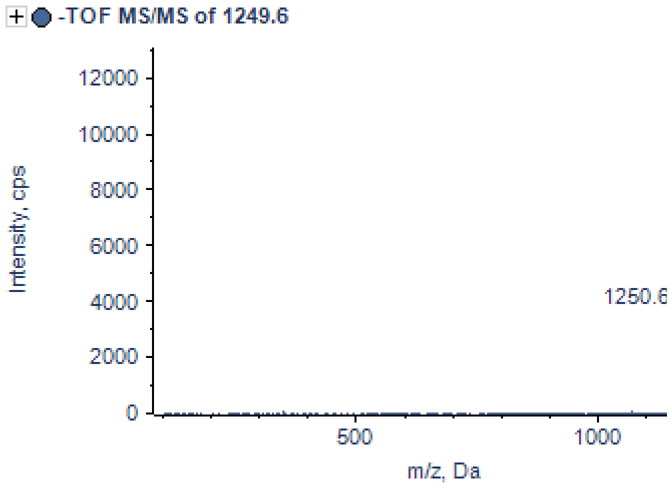

Parent MS/MS

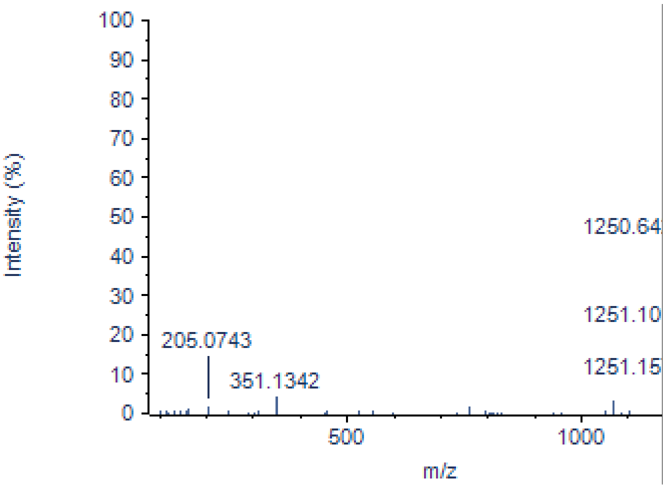

Common product ions (m/z): 1069.5758, 1249.6314, 1250.6390

Peaks selected for assignment (m/z): 115.0408, 131.0361, 143.0367, 145.0538, 157.0152, 163.0630, 205.0742, 247.0823, 291.1097, 301.0635, 309.1251, 351.1341, 455.3531, 457.3736, 582.2263, 759.4383, 1044.8579, 1069.5758, 1070.5468, 1087.5689, 1103.6178, 1199.5322, 1243.1215, 1245.5390, 1246.6600, 1248.1766, 1248.3890, 1248.5706, 1249.6314

Metabolite Options

Number of fragment peaks selected for assignment:	30

Minimum signal-to-noise ratio:	3
MS/MS m/z tolerance:	15 ppm
Fragmentation Settings	
Break aromatic rings:	True
Maximum number of bonds to break:	4
Maximum number of C-C bonds to break:	4
Label Settings	
Label peaks with:	Ion with ppm Error

Fragment Filters

Mass Range (m/z)	300 To 1500
Fragments with assigned structures	True

Fragment Details	Structure Details
Mass (m/z): 351.1341<br>Ion Formula: C14H23O10<br>Error (ppm): 12.7<br>Intensity (cps): 68.7<br>RDB: 3.0	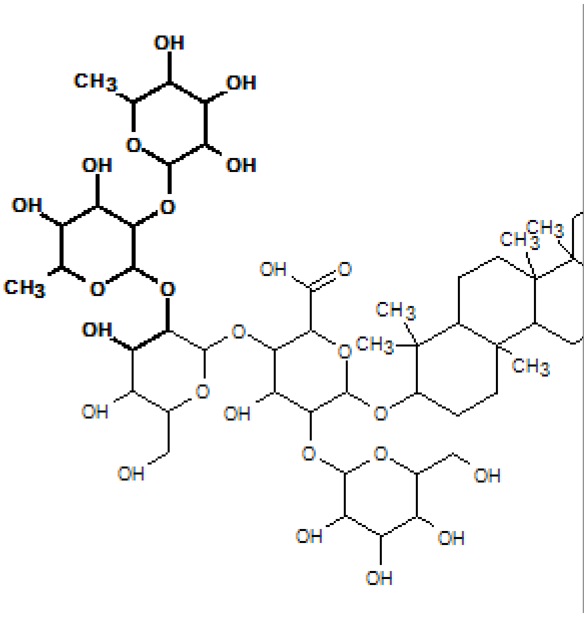<br>Selected: C14H24O10<br>Mass: 352.1369<br>Broken Bonds: 2<br>Delta H: -2

Contained Neutral Losses

Mass	Formula
208.0974	C8H16O6
194.1189	C8H18O5
188.0712	C8H12O5
146.0599	C6H10O4
60.0244	C2H4O2

Fragment Details	Structure Details
Mass (m/z): 455.3531	

Fragment Details	Structure Details
Ion Formula: C30H47O3<br>Error (ppm): 0.0<br>Intensity (cps): 12.0<br>RDB: 7.0	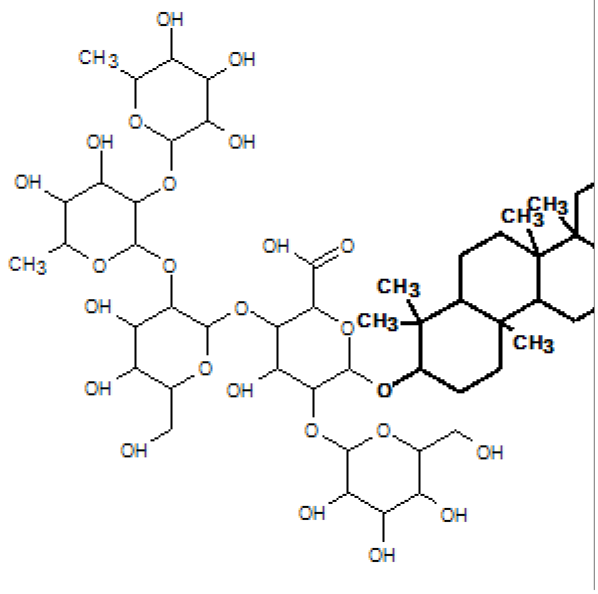<br>Selected: C30H49O3<br>Mass: 457.3682<br>Broken Bonds: 1<br>Delta H: -2<br>**Contained Neutral Losses**<br>No contained neutral losses
Mass (m/z): 457.3736<br>Ion Formula: C30H49O3<br>Error (ppm): 10.6<br>Intensity (cps): 13.3<br>RDB: 6.0	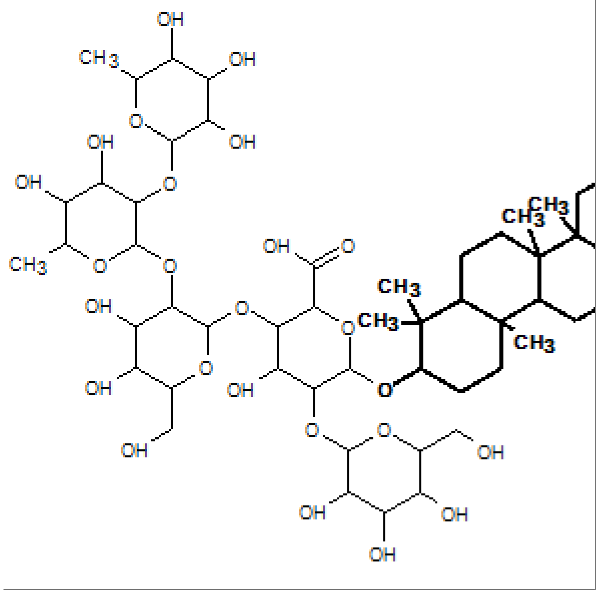<br>Selected: C30H49O3<br>Mass: 457.3682<br>Broken Bonds: 1<br>Delta H: 0<br>**Contained Neutral Losses**<br>No contained neutral losses
Mass (m/z): 759.4383<br>Ion Formula: C42H63O12<br>Error (ppm): 7.6	

Fragment Details	Structure Details
Intensity (cps): 28.0<br>RDB: 11.0	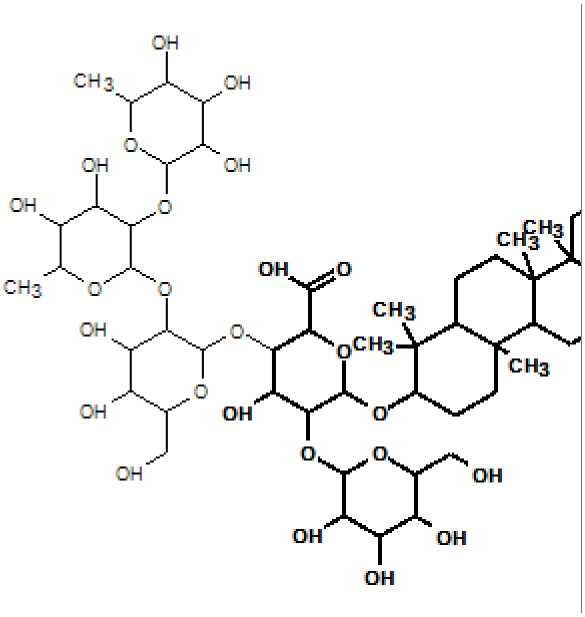<br>Selected: C42H66O12<br>Mass: 762.4554<br>Broken Bonds: 2<br>Delta H: -4

Contained Neutral Losses

Mass	Formula
602.4231	C36H58O7

Fragment Details	Structure Details
Mass (m/z): 1087.5689<br>Ion Formula: C54H87O22<br>Error (ppm): -0.5<br>Intensity (cps): 11.3<br>RDB: 11.0	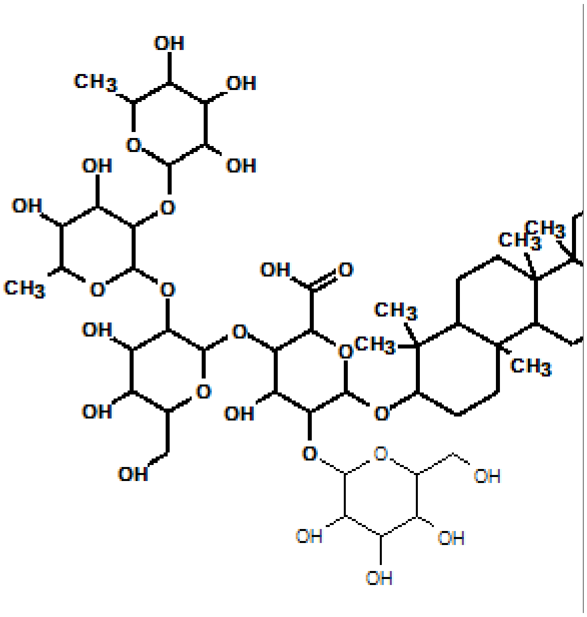<br>Selected: C54H87O22<br>Mass: 1087.5689<br>Broken Bonds: 1<br>Delta H: 0

Contained Neutral Losses

Mass	Formula
944.5321	C48H80O18
924.5059	C48H76O17
796.4591	C42H68O14

Fragment Details	Structure Details

632.2158	C24H40O19
630.1953	C24H38O19
17.9930	H2O

Mass (m/z):	1249.6314
Ion Formula:	C60H97O27
Error (ppm):	7.3
Intensity (cps):	13047.8
RDB:	12.0

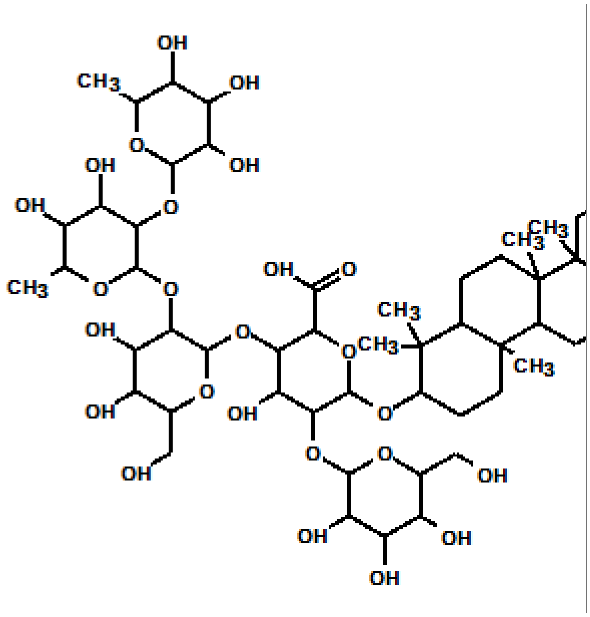

Selected:	C60H98O27
Mass:	1250.6295
Broken Bonds:	0
Delta H:	0

Contained Neutral Losses

Mass	Formula
1106.5947	C54H90O23
1086.5685	C54H86O22
958.5217	C48H78O19
794.2783	C30H50O24
792.2579	C30H48O24
162.0626	C6H10O5

Interpretation of the metabolite in rat (M9)

M9 – Loss of C6H10O4 [M-H]-

Formula:	C54H88O23
ppm:	11.9
RDB:	11.0

Available Structure Candidates

Rank	Score	Count
1	100.0	1

Applied Metabolite Structure

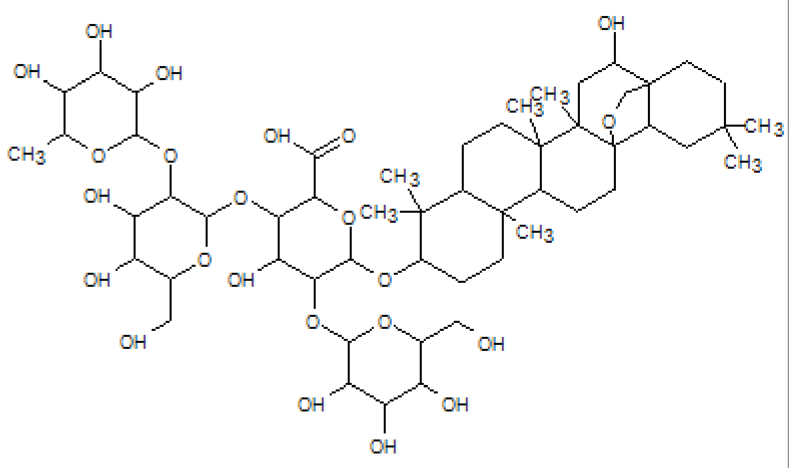

Composition:	C54H88O23
Mass:	1104.5716

Metabolite MS/MS

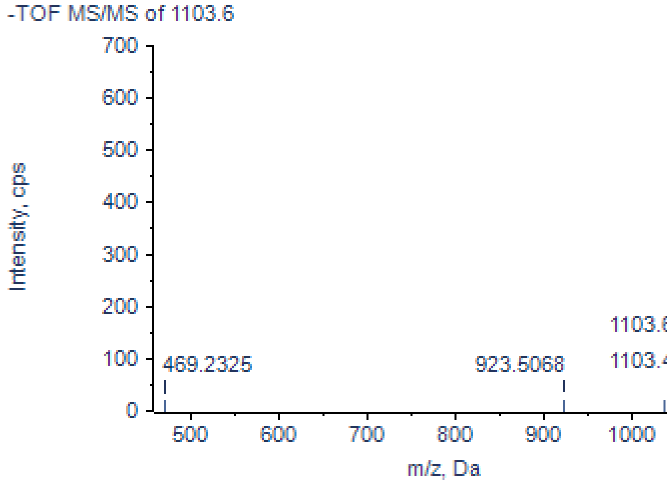

Parent MS/MS

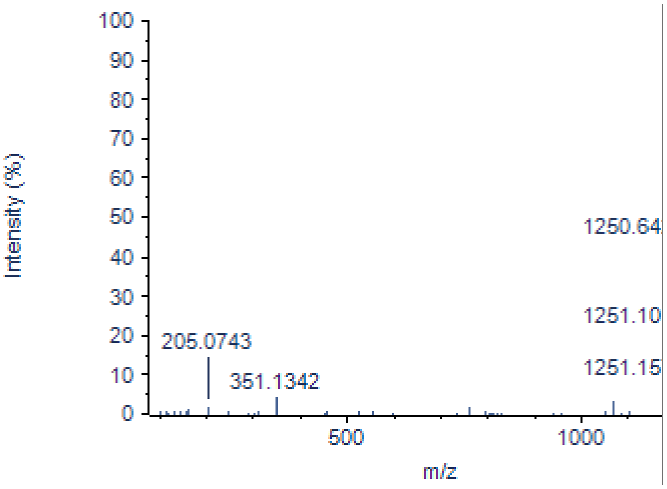

Peaks selected for assignment (m/z): 469.2325, 923.5068, 1035.5142, 1085.5386, 1103.4784, 1103.4948, 1103.5757, 1103.6418, 1103.6697

Metabolite Options

Number of fragment peaks selected for assignment:	30
Minimum signal-to-noise ratio:	3
MS/MS m/z tolerance:	15 ppm
Fragmentation Settings	
Break aromatic rings:	True
Maximum number of bonds to break:	4
Maximum number of C-C bonds to break:	4
Label Settings	
Label peaks with:	Ion with ppm Error

Fragment Filters

Mass Range (m/z)	300 To 1500

Fragment Details	Structure Details
Mass (m/z): 1103.5757<br>Ion Formula: C54H87O23<br>Error (ppm): 10.3<br>Intensity (cps): 700.0<br>RDB: 11.0	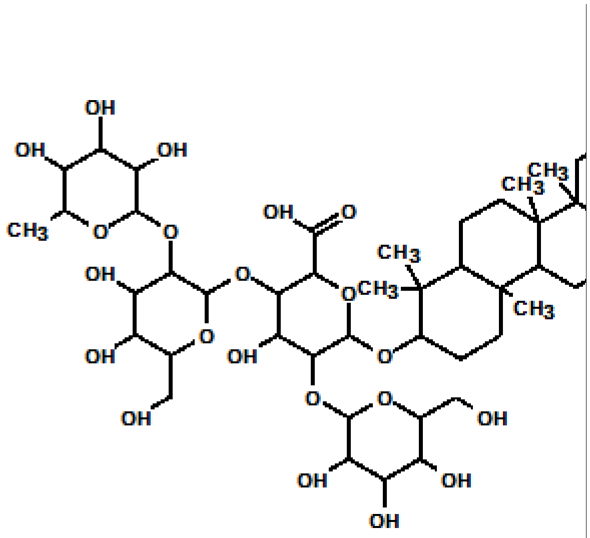<br>Selected: C54H88O23<br>Mass: 1104.5716<br>Broken Bonds: 0<br>Delta H: 0<br>**Contained Neutral Losses**<br>No contained neutral losses

Interpretation of the metabolite in rat (M10)

M10 – Loss of Hydroxymethylene [M-H]-

Formula:	C59H96O26
ppm:	8.6
RDB:	12.0

Available Structure Candidates

Rank	Score	Count
1	NaN	2

Applied Metabolite Structure

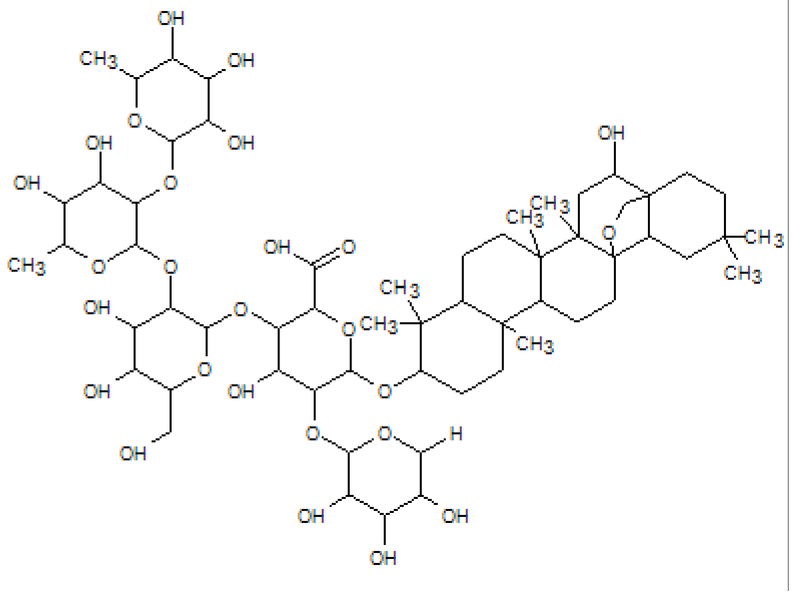

Composition:	C59H96O26
Mass:	1220.6190

Metabolite MS/MS

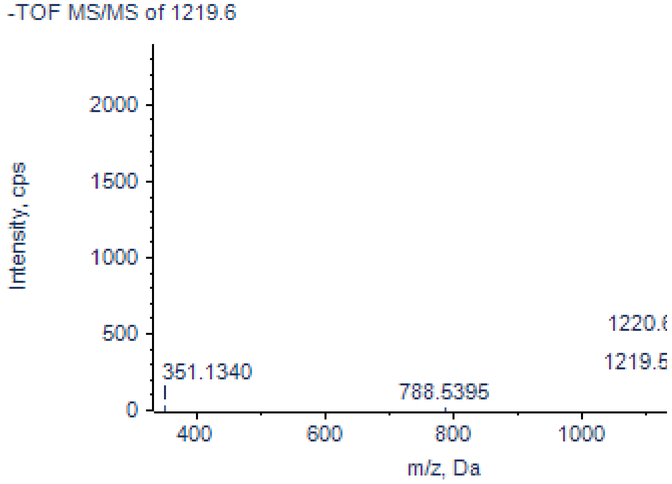

Parent MS/MS

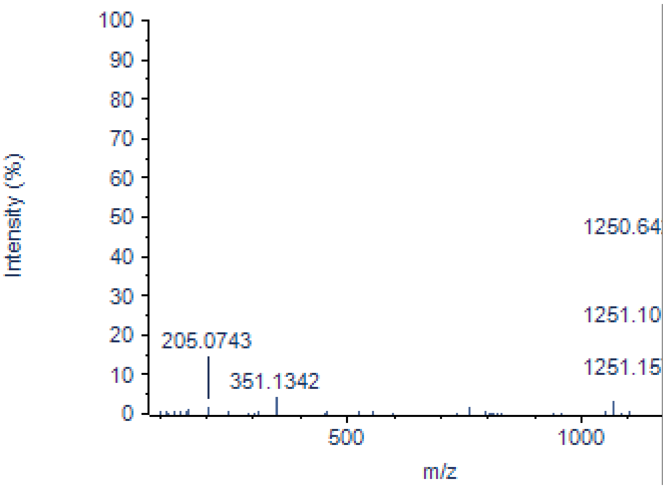

Peaks selected for assignment (m/z): 351.1340, 788.5395, 1219.4378, 1219.6250, 1219.7183

Metabolite Options

Number of fragment peaks selected for assignment:	30
Minimum signal-to-noise ratio:	3
MS/MS m/z tolerance:	15 ppm
Fragmentation Settings	
Break aromatic rings:	True
Maximum number of bonds to break:	4
Maximum number of C-C bonds to break:	4
Label Settings	
Label peaks with:	Ion with ppm Error

Fragment Filters

Mass Range (m/z)	300 To 1500

Fragment Details	Structure Details
Mass (m/z): 1219.6250<br>Ion Formula: C59H95O26<br>Error (ppm): 10.9<br>Intensity (cps): 2393.0<br>RDB: 12.0	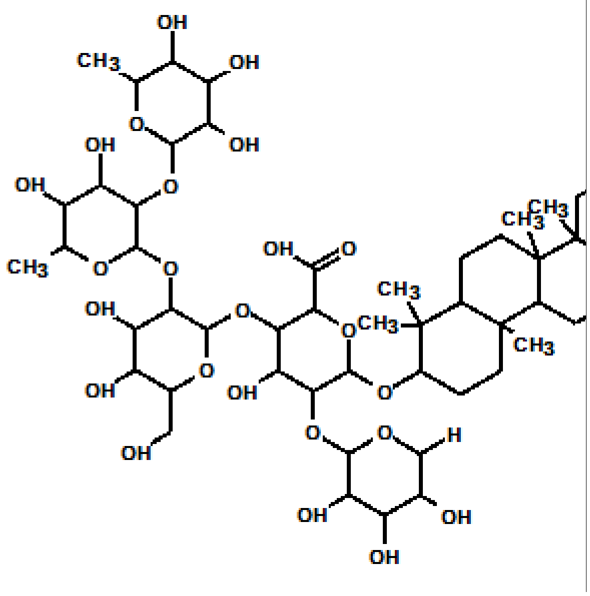<br>Selected: C59H96O26<br>Mass: 1220.6190<br>Broken Bonds: 0<br>Delta H: 0<br>**Contained Neutral Losses**<br>No contained neutral losses

Interpretation of the metabolite in rat (M11)

M11 – Loss of C12H20O8+Decarboxylation [M-H]-

Formula:	C47H78O17
ppm:	8.7
RDB:	9.0

Available Structure Candidates

Rank	Score	Count
1	100.0	1

Applied Metabolite Structure

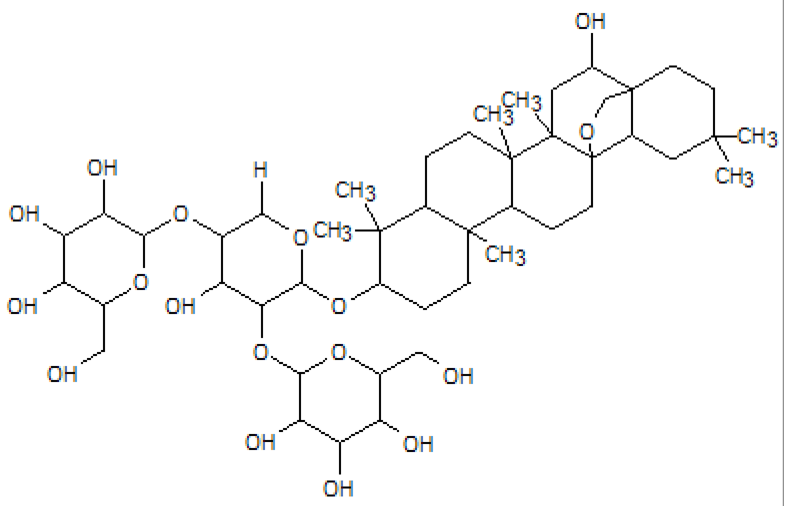

Composition:	C47H78O17
Mass:	914.5239

Metabolite MS/MS

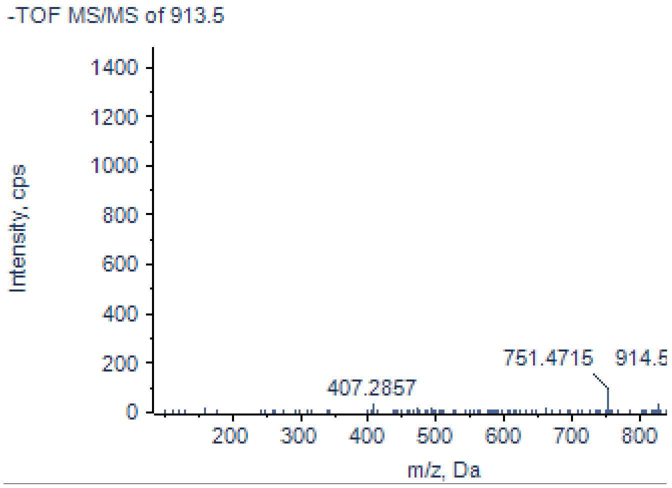

Parent MS/MS

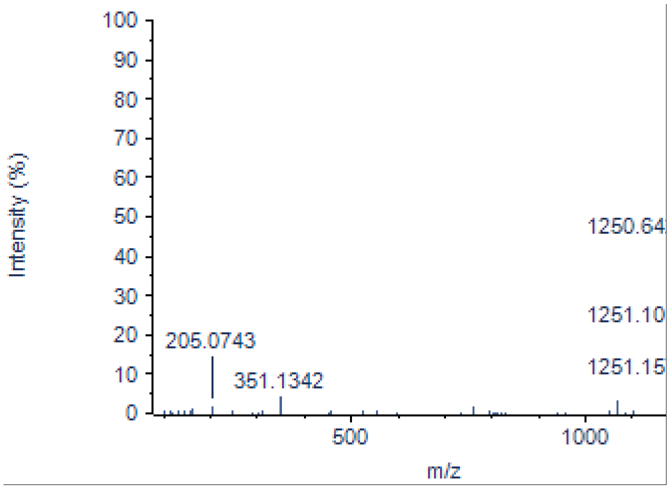

Peaks selected for assignment (m/z): 101.0254, 113.0267, 131.0352, 161.0493, 177.7068, 177.9484, 275.0786, 339.2034, 407.2857, 459.7042, 471.2378, 471.2521, 493.2093, 493.2229, 495.2547, 505.3584, 586.0536, 605.9391, 661.5858, 751.4402, 751.4715, 751.4929, 751.5165, 825.4615, 825.4867, 867.4573, 867.4817, 878.1754, 896.3504, 913.5265

Metabolite Options

Number of fragment peaks selected for assignment:	30
Minimum signal-to-noise ratio:	3
MS/MS m/z tolerance:	15 ppm
Fragmentation Settings	
Break aromatic rings:	True
Maximum number of bonds to break:	4
Maximum number of C-C bonds to break:	4
Label Settings	
Label peaks with:	Ion with ppm Error

Fragment Filters

Mass Range (m/z)	300 To 1500
Fragments with assigned structures	True

<table>
<tr><th>Fragment Details</th><th>Structure Details</th></tr>
<tr><td>Mass (m/z): 751.4715
Ion Formula: C41H67O12
Error (ppm): 10.2
Intensity (cps): 97.6
RDB: 8.0</td><td>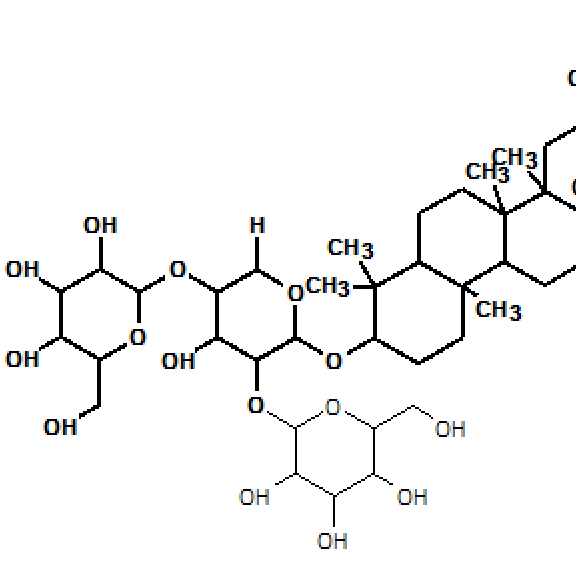
Selected: C41H67O12
Mass: 751.4633
Broken Bonds: 1
Delta H: 0
Contained Neutral Losses
No contained neutral losses</td></tr>
<tr><td>Mass (m/z): 825.4615</td><td></td></tr>
</table>

Fragment Details	Structure Details
Ion Formula: C43H69O15<br>Error (ppm): -3.3<br>Intensity (cps): 30.0<br>RDB: 9.0	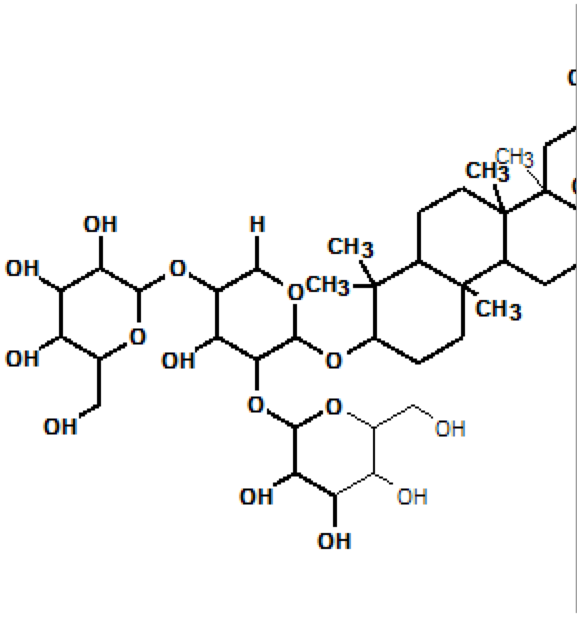<br>Selected: C43H69O15<br>Mass: 825.4636<br>Broken Bonds: 3<br>Delta H: -2<br>**Contained Neutral Losses**<br>No contained neutral losses
Mass (m/z): 867.4817<br>Ion Formula: C45H71O16<br>Error (ppm): 7.9<br>Intensity (cps): 40.0<br>RDB: 10.0	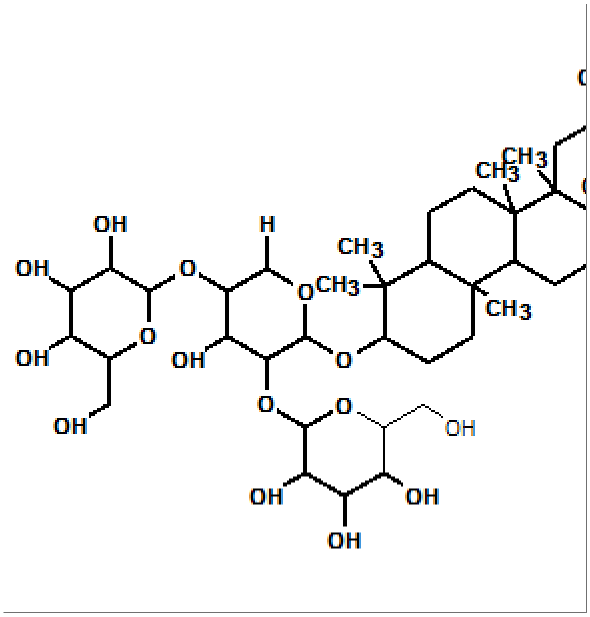<br>Selected: C45H74O16<br>Mass: 870.4977<br>Broken Bonds: 2<br>Delta H: -4<br>**Contained Neutral Losses**<br>Mass: 116.0102 — Formula: C4H4O4
Mass (m/z): 913.5265<br>Ion Formula: C47H77O17	

<table>
<tr><th>Fragment Details</th><th>Structure Details</th></tr>
<tr><td>Error (ppm): 10.8
Intensity (cps): 1478.1
RDB: 9.0</td><td>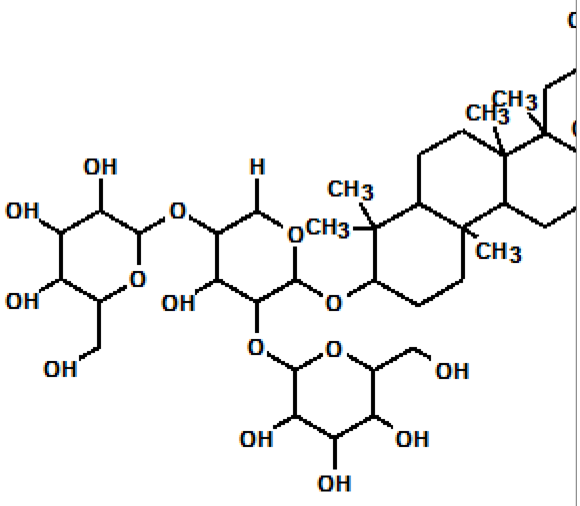
Selected: C47H78O17
Mass: 914.5239
Broken Bonds: 0
Delta H: 0
Score: 44.0

Contained Neutral Losses
Mass: 162.0550 — Formula: C6H10O5</td></tr>
</table>

Interpretation of the metabolite in rat (M12)

M12 – Loss of C12H20O8+Glucose Conjugation [M-H]-

Formula:	C54H88O24
ppm:	8.6
RDB:	11.0

Available Structure Candidates

Rank	Score	Count
1	100.0	11

Applied Metabolite Structure

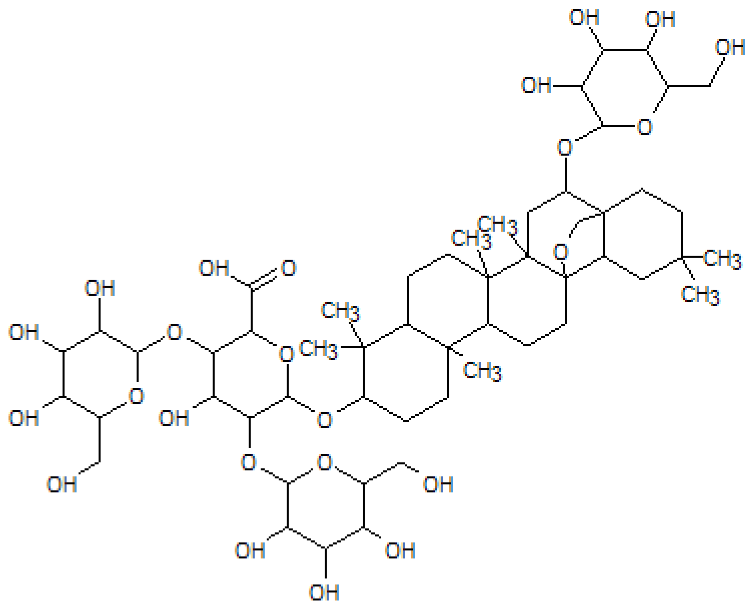

Composition:	C54H88O24
Mass:	1120.5666

Metabolite MS/MS

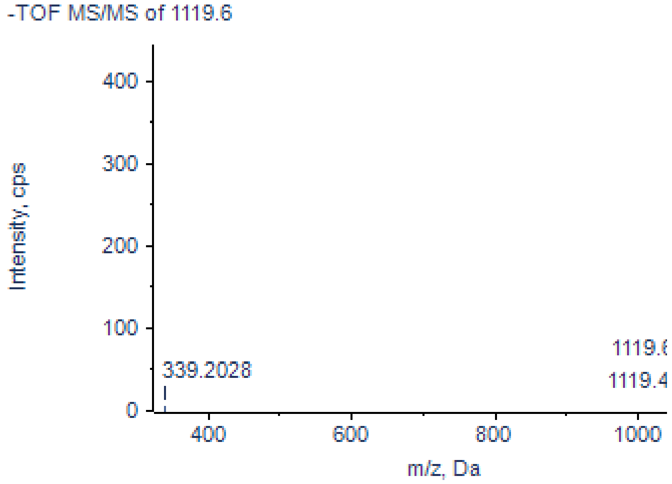

Parent MS/MS

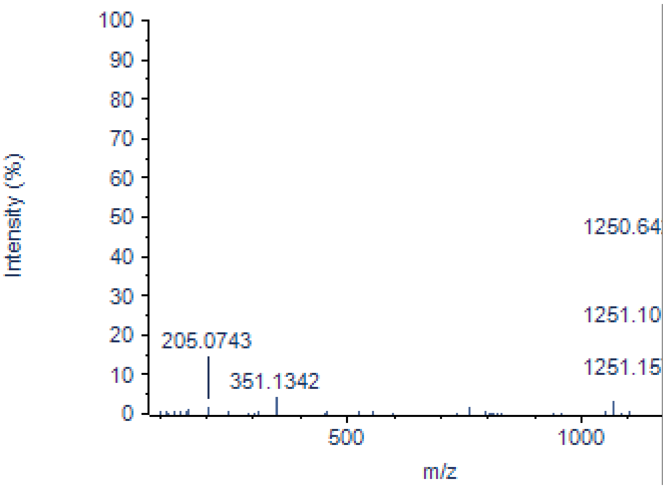

Peaks selected for assignment (m/z): 339.2028, 1073.5454, 1101.5588, 1119.5729, 1119.6397

Metabolite Options

Number of fragment peaks selected for assignment:	30
Minimum signal-to-noise ratio:	3
MS/MS m/z tolerance:	15 ppm
Fragmentation Settings	
Break aromatic rings:	True
Maximum number of bonds to break:	4
Maximum number of C-C bonds to break:	4
Label Settings	
Label peaks with:	Ion with ppm Error

Fragment Filters

Fragments with assigned structures True

Fragment Details	Structure Details
Mass (m/z): 1119.5729<br>Ion Formula: C54H87O24<br>Error (ppm): 12.2<br>Intensity (cps): 443.0<br>RDB: 11.0<br>Score: 44.0	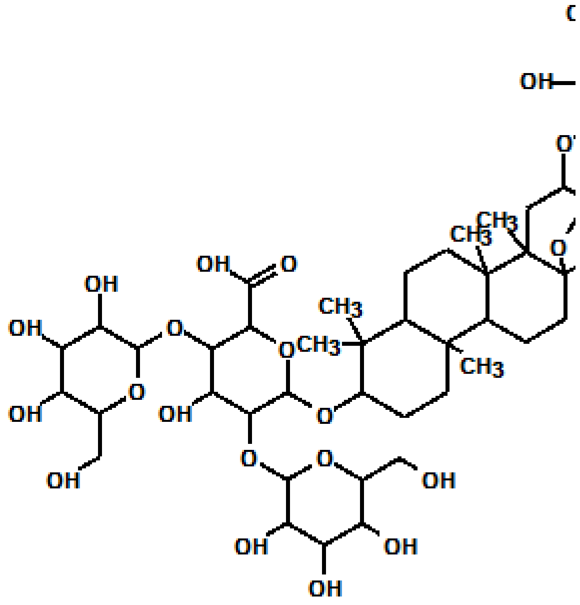<br>Selected: C54H88O24<br>Mass: 1120.5666<br>Broken Bonds: 0<br>Delta H: 0<br>Score: 44.0<br>**Contained Neutral Losses**<br>No contained neutral losses

Interpretation of the metabolite in rat (M13)

M13 – Loss of C12H20O9+Demethylation to Carboxylic Acid [M-H]-

Formula: C48H76O20

ppm: 7.6

RDB: 11.0

Available Structure Candidates

Rank	Score	Count
1	100.0	36
2	0.0	27

Applied Metabolite Structure

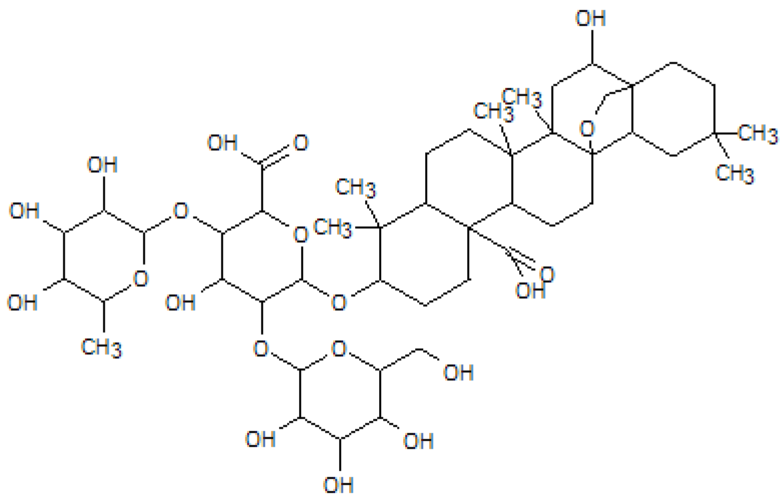

Composition:	C48H76O20
Mass:	972.4930

Metabolite MS/MS

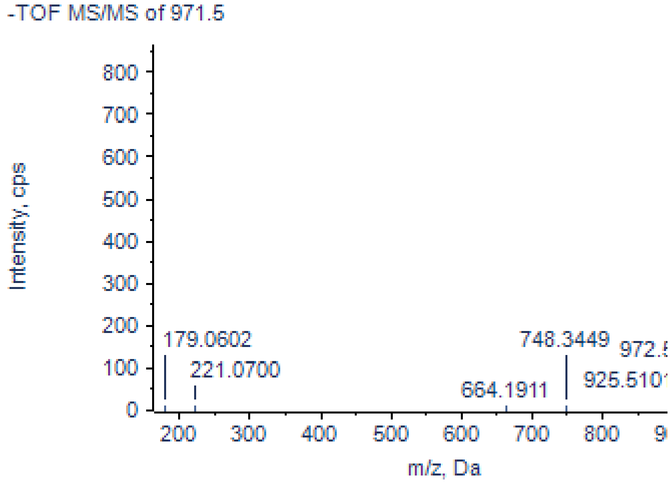

Parent MS/MS

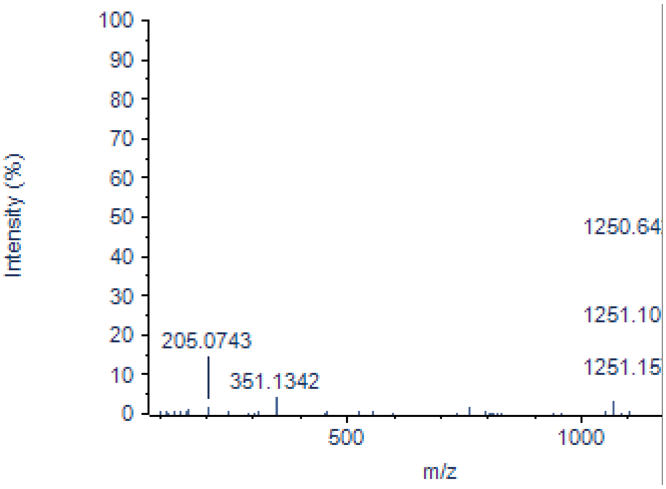

Peaks selected for assignment (m/z): 179.0602, 221.0700, 664.1911, 748.3449, 925.4744, 925.4884, 925.5101, 953.4725, 953.4961, 953.5113, 971.4016, 971.4207, 971.4340, 971.4975, 971.5642, 971.5842

Metabolite Options

Number of fragment peaks selected for assignment:	30
Minimum signal-to-noise ratio:	3
MS/MS m/z tolerance:	15 ppm
Fragmentation Settings	
Break aromatic rings:	True
Maximum number of bonds to break:	4
Maximum number of C-C bonds to break:	4
Label Settings	
Label peaks with:	Ion with ppm Error

Fragment Filters

Fragments with assigned structures	True

Fragment Details	Structure Details
Mass (m/z): 971.4975<br>Ion Formula: C48H75O20<br>Error (ppm): 12.1<br>Intensity (cps): 863.0<br>RDB: 11.0<br>Score: 44.0	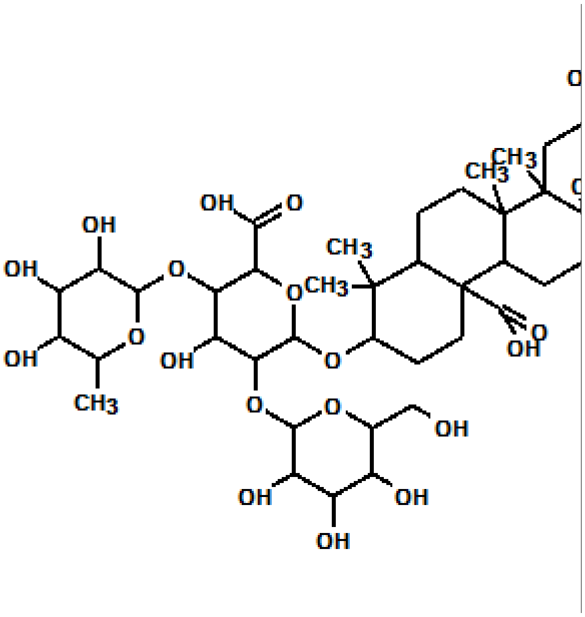<br>Selected: C48H76O20<br>Mass: 972.4930<br>Broken Bonds: 0<br>Delta H: 0<br>Score: 44.0<br>**Contained Neutral Losses**<br>No contained neutral losses

Interpretation of the metabolite in rat (M14)

M14 – Loss of C12H20O9 [M-H]-

Formula:	C48H78O18
ppm:	7.8
RDB:	10.0

Available Structure Candidates

Rank	Score	Count
1	100.0	7
2	0.0	5

Applied Metabolite Structure

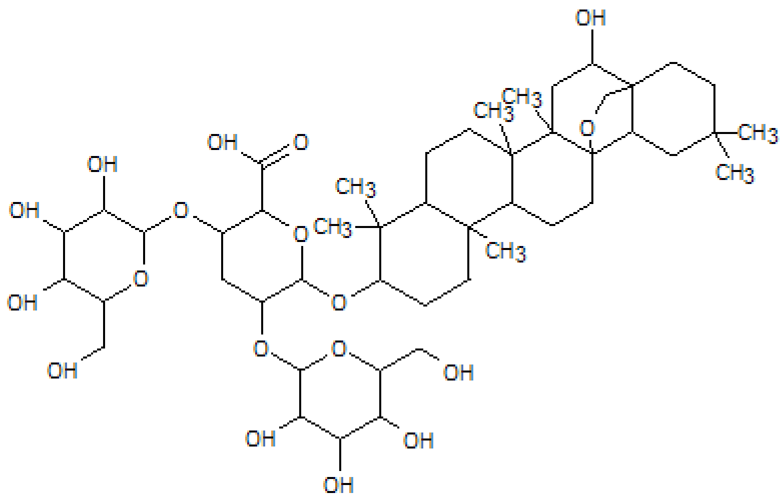

Composition:	C48H78O18
Mass:	942.5188

Metabolite MS/MS

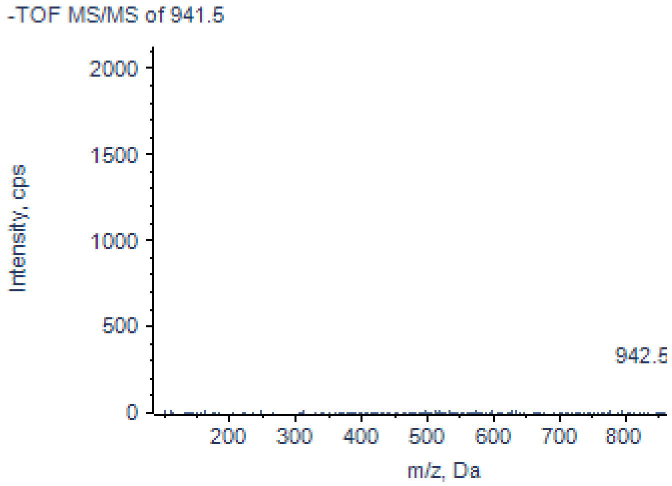

Parent MS/MS

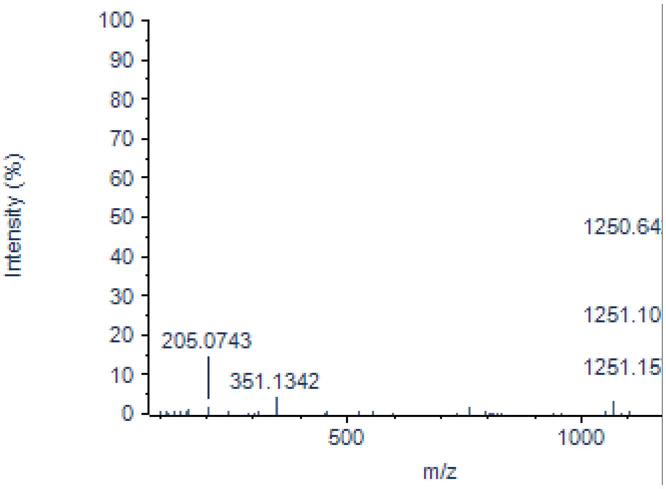

Peaks selected for assignment (m/z): 111.0104, 145.0514, 163.0626, 205.0761, 471.3140, 497.2960, 513.2837, 513.3077, 519.3451, 519.3609, 533.8852, 573.3781, 573.3889, 573.4048, 597.3693, 627.6491, 633.4079, 633.4287, 712.1875, 777.4566, 795.4616, 795.4865, 827.5398, 873.4570, 873.5487, 879.1636, 895.4068, 895.5233, 938.4001, 941.5218

Metabolite Options

Number of fragment peaks selected for assignment:	30
Minimum signal-to-noise ratio:	3
MS/MS m/z tolerance:	15 ppm
Fragmentation Settings	
Break aromatic rings:	True
Maximum number of bonds to break:	4

Maximum number of C-C bonds to break: 4

Label Settings

Label peaks with: Ion with ppm Error

Fragment Filters

Mass Range (m/z) 300 To 1500

Fragment Details	Structure Details
Mass (m/z): 941.5218<br>Ion Formula: C48H77O18<br>Error (ppm): 10.9<br>Intensity (cps): 2121.1<br>RDB: 10.0	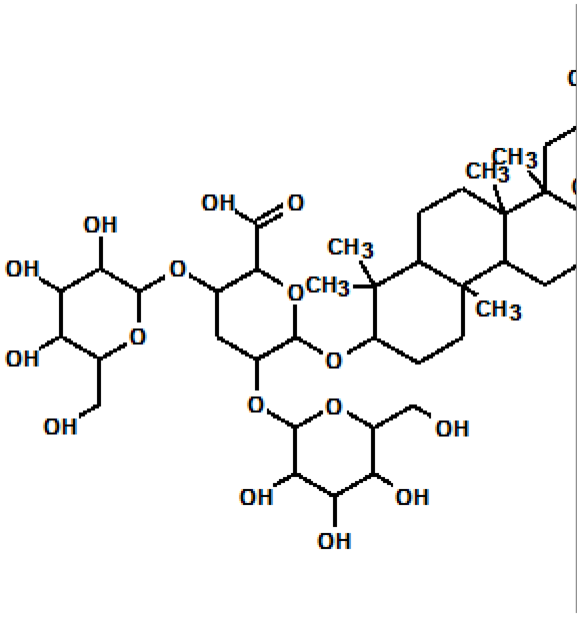<br>Selected: C48H78O18<br>Mass: 942.5188<br>Broken Bonds: 0<br>Delta H: 0<br>**Contained Neutral Losses**<br>No contained neutral losses

Interpretation of the metabolite in rat (M15)

M15 – Loss of C12H20O8 [M-H]-

Formula: C48H78O19

ppm: 6.8

RDB: 10.0

Available Structure Candidates

Rank	Score	Count
1	100.0	1

Applied Metabolite Structure

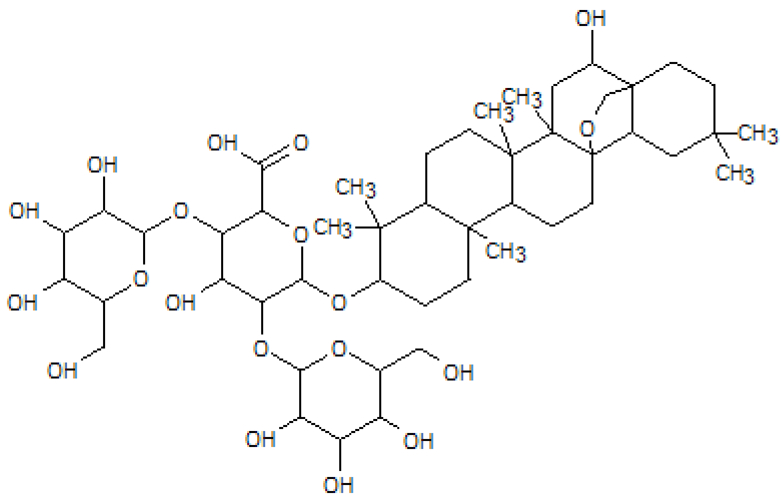

Composition:	C48H78O19
Mass:	958.5137

Metabolite MS/MS

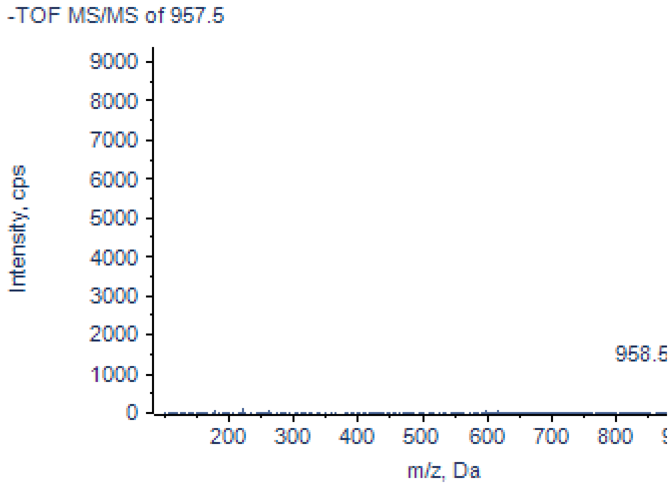

Parent MS/MS

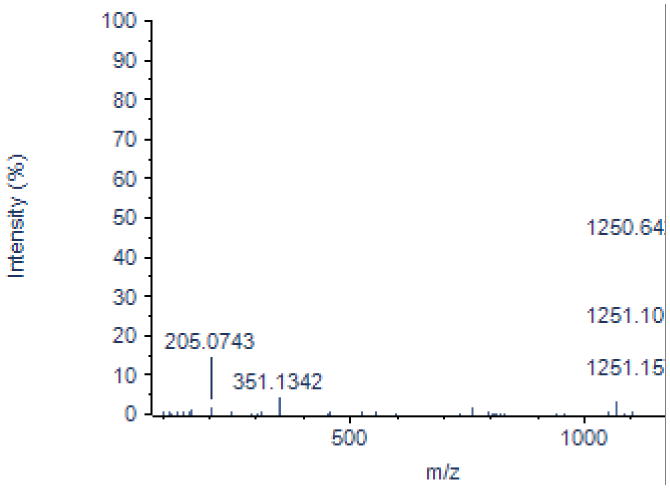

Peaks selected for assignment (m/z): 101.0254, 113.0263, 119.0362, 131.0360, 139.0050, 157.0158, 159.0350, 161.0466, 179.0586, 221.0685, 221.0844, 263.0797, 323.1051, 457.3733, 481.1323, 499.1387, 525.3966, 525.4116, 597.3881, 615.3983, 733.4627, 777.4518, 795.4735, 895.5160, 895.5518, 939.5086, 939.5671, 954.3269, 957.0542, 957.5151

Metabolite Options

Number of fragment peaks selected for assignment:	30
Minimum signal-to-noise ratio:	3
MS/MS m/z tolerance:	15 ppm
Fragmentation Settings	
Break aromatic rings:	True
Maximum number of bonds to break:	4

Maximum number of C-C bonds to break: 4

Label Settings

Label peaks with: Ion with ppm Error

Fragment Filters

Fragments with assigned structures True

Fragment Details	Structure Details
Mass (m/z): 221.0685<br>Ion Formula: C8H13O7<br>Error (ppm): 8.1<br>Intensity (cps): 93.5<br>RDB: 2.0<br>Score: 29.5	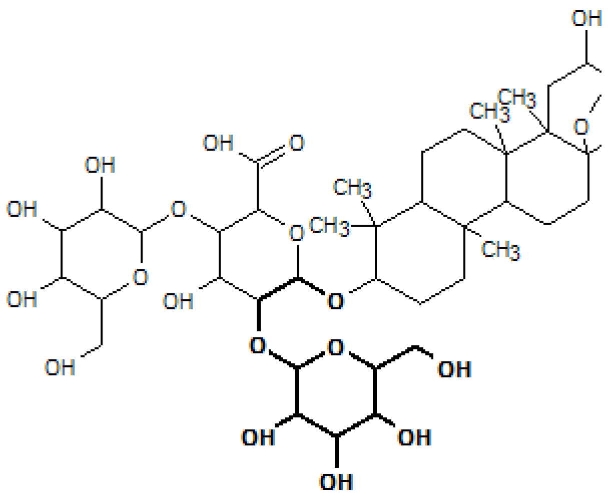<br>Selected: C8H13O7<br>Mass: 221.0661<br>Broken Bonds: 3<br>Delta H: -2<br>Score: 29.5<br><br>**Contained Neutral Losses**
Mass (m/z): 263.0797<br>Ion Formula: C10H15O8<br>Error (ppm): 9.3<br>Intensity (cps): 54.1<br>RDB: 3.0<br>Score: 29.5	

Contained Neutral Losses

Mass	Formula
60.0219	C2H4O2
42.0099	C2H2O

<table>
<tr><th>Fragment Details</th><th>Structure Details</th></tr>
<tr><td></td><td>
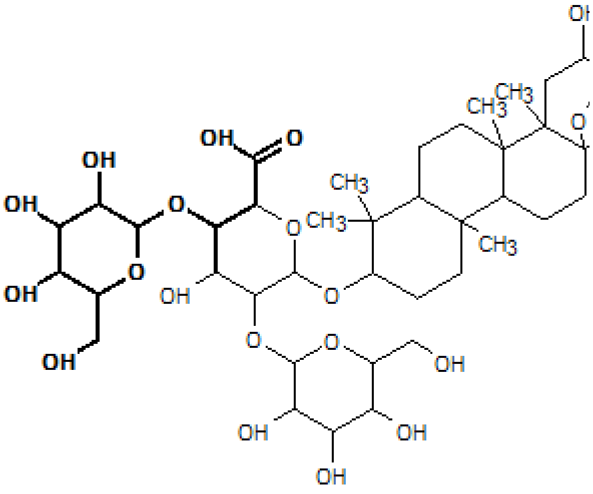

Selected: C10H15O8
Mass: 263.0767
Broken Bonds: 3
Delta H: -2
Score: 29.5

Contained Neutral Losses

<table>
<tr><th>Mass</th><th>Formula</th></tr>
<tr><td>162.0543</td><td>C6H10O5</td></tr>
<tr><td>106.0639</td><td>C4H10O3</td></tr>
<tr><td>102.0331</td><td>C4H6O3</td></tr>
<tr><td>84.0211</td><td>C4H4O2</td></tr>
</table>
</td></tr>
<tr><td>
Mass (m/z): 457.3733
Ion Formula: C30H49O3
Error (ppm): 10.1
Intensity (cps): 14.5
RDB: 6.0
Score: 40.5
</td><td>
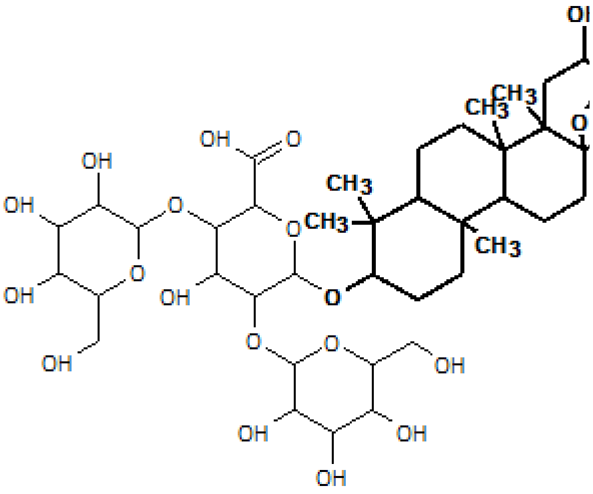

Selected: C30H49O3
Mass: 457.3682
Broken Bonds: 1
Delta H: 0
Score: 40.5

Contained Neutral Losses

No contained neutral losses
</td></tr>
</table>

<table>
<tr><th>Fragment Details</th><th>Structure Details</th></tr>
<tr>
<td>Mass (m/z): 597.3881
Ion Formula: C36H53O7
Error (ppm): 14.2
Intensity (cps): 57.2
RDB: 10.0
Score: 36.0</td>
<td>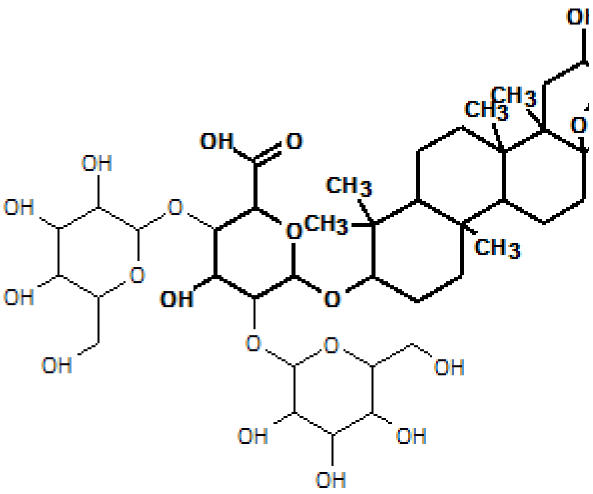
Selected: C36H56O7
Mass: 600.4026
Broken Bonds: 2
Delta H: -4
Score: 36.0

Contained Neutral Losses
<table>
<tr><th>Mass</th><th>Formula</th></tr>
<tr><td>458.3832</td><td>C30H50O3</td></tr>
<tr><td>440.3723</td><td>C30H48O2</td></tr>
<tr><td>140.0148</td><td>C6H4O4</td></tr>
</table></td>
</tr>
<tr>
<td>Mass (m/z): 615.3983
Ion Formula: C36H55O8
Error (ppm): 13.0
Intensity (cps): 45.1
RDB: 9.0
Score: 36.5</td>
<td>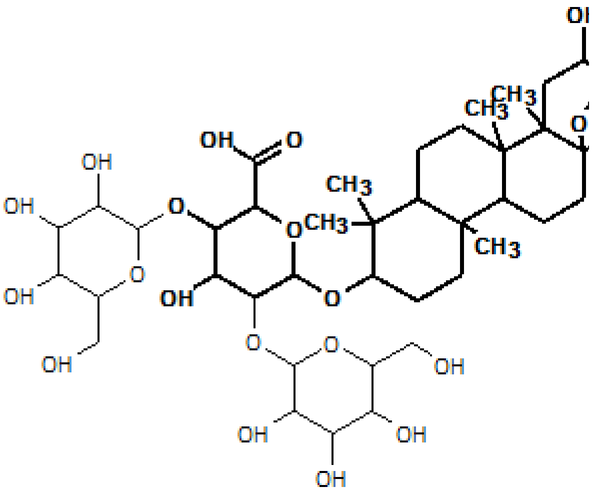
Selected: C36H56O8
Mass: 616.3975
Broken Bonds: 2
Delta H: -2
Score: 36.5

Contained Neutral Losses
<table>
<tr><th>Mass</th><th>Formula</th></tr>
</table></td>
</tr>
</table>

Fragment Details	Structure Details

458.3825	C30H50O3
454.3516	C30H46O3
158.0249	C6H6O5
18.0101	H2O

Mass (m/z):	733.4627
Ion Formula:	C41H65O11
Error (ppm):	12.9
Intensity (cps):	35.0
RDB:	9.0
Score:	33.5

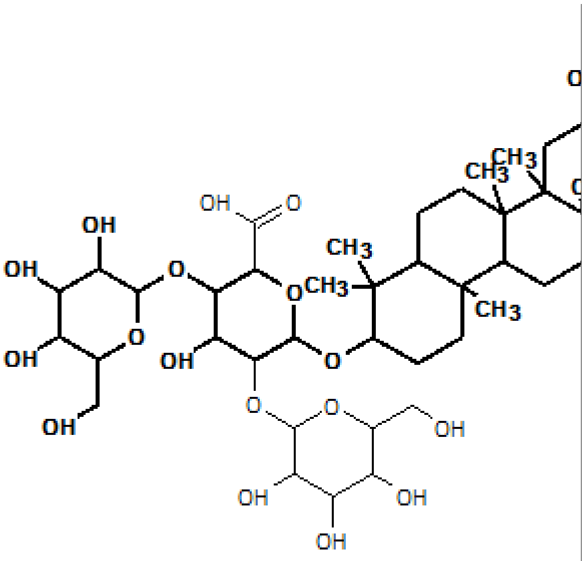

Selected:	C41H66O11
Mass:	734.4605
Broken Bonds:	2
Delta H:	-2
Score:	33.5

Contained Neutral Losses

Mass	Formula
576.4469	C35H60O6
572.4161	C35H56O6
276.0894	C11H16O8

Mass (m/z):	777.4518
Ion Formula:	C42H65O13
Error (ppm):	11.2
Intensity (cps):	8.7
RDB:	10.0
Score:	40.0

Fragment Details	Structure Details

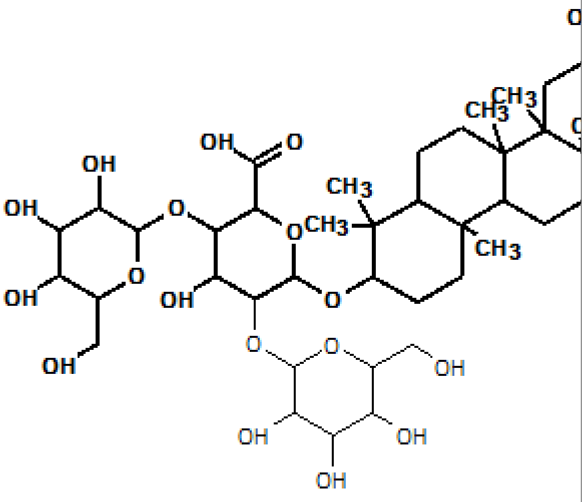

Selected:	C42H67O13
Mass:	779.4582
Broken Bonds:	1
Delta H:	-2
Score:	40.0

Contained Neutral Losses

Mass	Formula
620.4360	C36H60O8
320.0784	C12H16O10
180.0636	C6H12O6
162.0535	C6H10O5
43.9890	CO2

Mass (m/z):	895.5160
Ion Formula:	C47H75O16
Error (ppm):	11.1
Intensity (cps):	23.6
RDB:	10.0
Score:	33.5

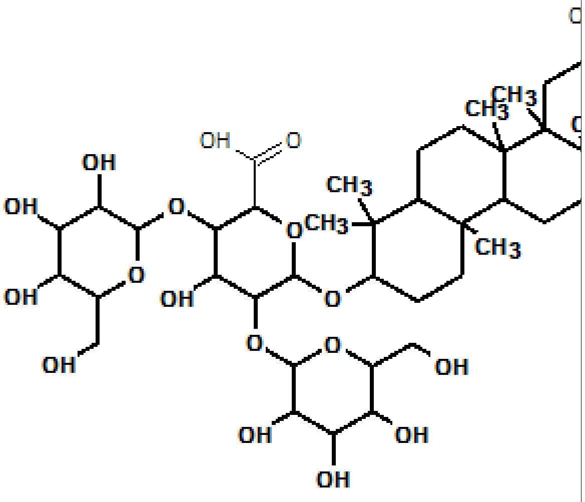

Selected:	C47H76O16
Mass:	896.5133
Broken Bonds:	2
Delta H:	-2
Score:	33.5

Contained Neutral Losses

Fragment Details	Structure Details

Mass	Formula
738.5002	C41H70O11
734.4694	C41H66O11
716.4574	C41H64O10
162.0533	C6H10O5

Mass (m/z):	939.5086
Ion Formula:	C48H75O18
Error (ppm):	13.5
Intensity (cps):	97.9
RDB:	11.0
Score:	40.0

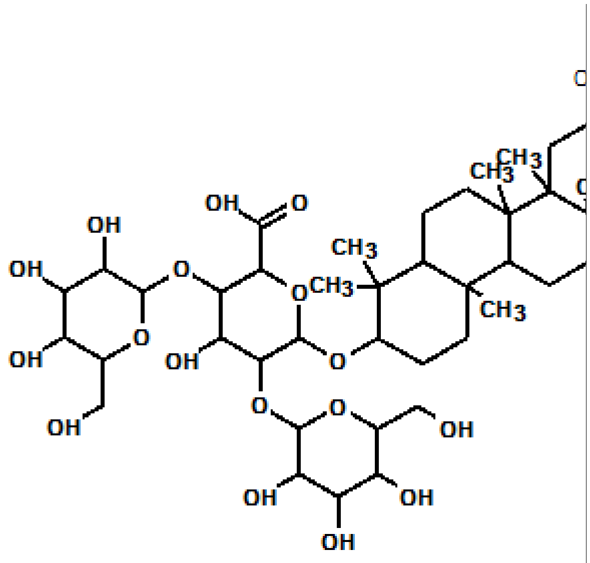

Selected:	C48H77O18
Mass:	941.5110
Broken Bonds:	1
Delta H:	-2
Score:	40.0

Contained Neutral Losses

Mass	Formula
782.4928	C42H70O13
778.4620	C42H66O13
760.4500	C42H64O12
162.0568	C6H10O5
43.9926	CO2

Mass (m/z):	957.5151
Ion Formula:	C48H77O19
Error (ppm):	9.1
Intensity (cps):	9354.2
RDB:	10.0
Score:	44.0

<table>
<tr><th>Fragment Details</th><th>Structure Details</th></tr>
<tr><td></td><td>
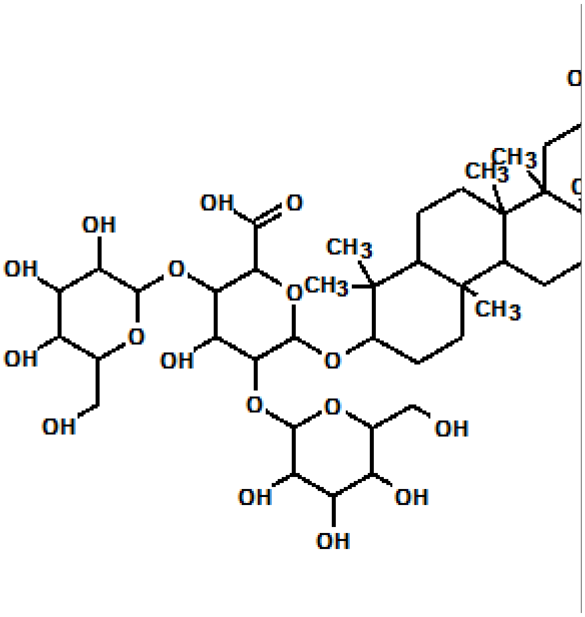

<table>
<tr><td>Selected:</td><td>C48H78O19</td></tr>
<tr><td>Mass:</td><td>958.5137</td></tr>
<tr><td>Broken Bonds:</td><td>0</td></tr>
<tr><td>Delta H:</td><td>0</td></tr>
<tr><td>Score:</td><td>44.0</td></tr>
</table>

Contained Neutral Losses

<table>
<tr><th>Mass</th><th>Formula</th></tr>
<tr><td>796.4685</td><td>C42H68O14</td></tr>
<tr><td>778.4565</td><td>C42H66O13</td></tr>
<tr><td>500.1418</td><td>C18H28O16</td></tr>
<tr><td>180.0634</td><td>C6H12O6</td></tr>
<tr><td>18.0066</td><td>H2O</td></tr>
</table>
</td></tr>
</table>

Interpretation of the metabolite in rat (M16)

M16 – Loss of C12H20O9 [M-H]-

Formula:	C48H78O18
ppm:	5.8
RDB:	10.0

Available Structure Candidates

Rank	Score	Count
1	100.0	7
2	0.0	5

Applied Metabolite Structure

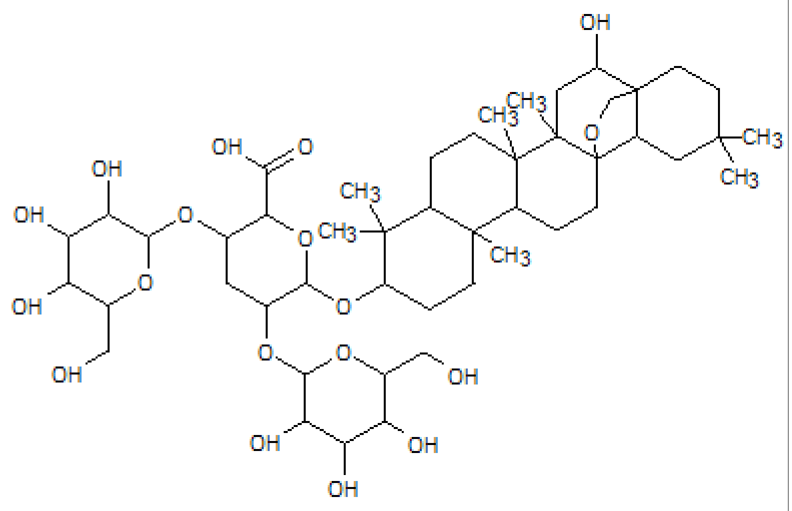

Composition:	C48H78O18
Mass:	942.5188

Metabolite MS/MS

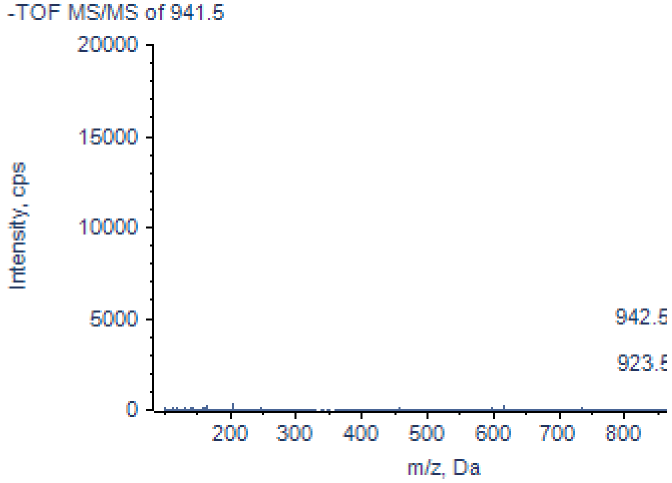

Parent MS/MS

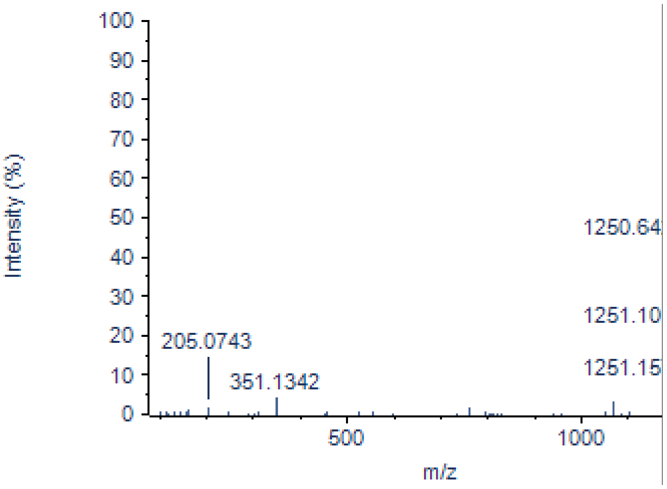

Peaks selected for assignment (m/z): 101.0252, 103.0409, 113.0256, 115.0413, 119.0360, 131.0360, 139.0052, 143.0368, 145.0525, 157.0160, 161.0475, 163.0628, 205.0738, 247.0847, 307.1055, 325.1175, 457.3728, 571.4055, 597.3869, 615.3976, 633.4045, 733.4612, 751.4734, 795.4616, 879.5202, 897.5323, 923.5115, 924.5160, 938.3879, 941.5188

Metabolite Options

Number of fragment peaks selected for assignment:	30
Minimum signal-to-noise ratio:	3
MS/MS m/z tolerance:	15 ppm
Fragmentation Settings	
Break aromatic rings:	True
Maximum number of bonds to break:	4

Maximum number of C-C bonds to break: 4

Label Settings

Label peaks with: Ion with ppm Error

Fragment Filters

Fragments with assigned structures True

<table>
<tr><th>Fragment Details</th><th>Structure Details</th></tr>
<tr>
<td>Mass (m/z): 205.0738
Ion Formula: C8H13O6
Error (ppm): 9.7
Intensity (cps): 322.4
RDB: 2.0
Score: 29.5</td>
<td>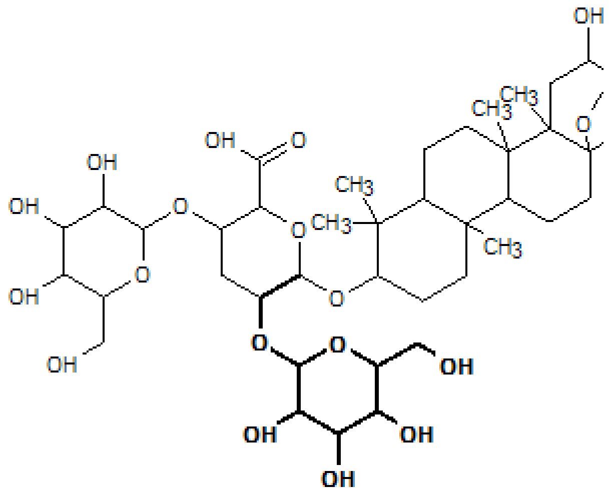

Selected: C8H13O6
Mass: 205.0712
Broken Bonds: 3
Delta H: -2
Score: 29.5
Contained Neutral Losses
<table>
<tr><th>Mass</th><th>Formula</th></tr>
<tr><td>44.0263</td><td>C2H4O</td></tr>
<tr><td>42.0110</td><td>C2H2O</td></tr>
</table>
</td>
</tr>
<tr>
<td>Mass (m/z): 247.0847
Ion Formula: C10H15O7
Error (ppm): 9.6
Intensity (cps): 162.6
RDB: 3.0
Score: 29.5</td>
<td></td>
</tr>
</table>

<table>
<tr><th>Fragment Details</th><th>Structure Details</th></tr>
<tr><td></td><td>

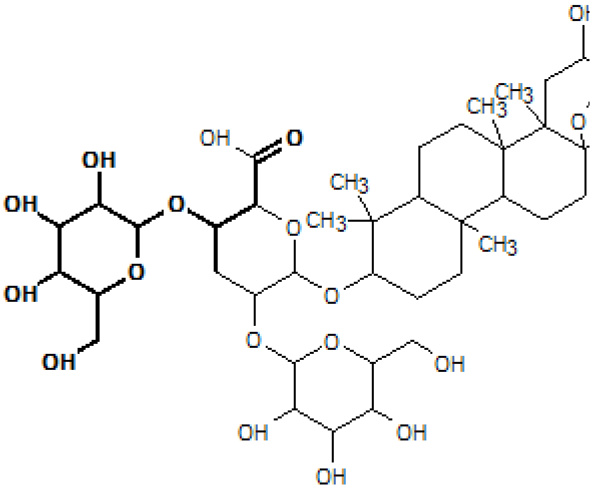

Selected:	C10H15O7
Mass:	247.0818
Broken Bonds:	3
Delta H:	-2
Score:	29.5

Contained Neutral Losses

Mass	Formula
90.0687	C4H10O2
86.0373	C4H6O2
84.0219	C4H4O2
42.0110	C2H2O

</td></tr>
<tr><td>

Mass (m/z):	307.1055
Ion Formula:	C12H19O9
Error (ppm):	6.7
Intensity (cps):	38.6
RDB:	3.0
Score:	36.5

</td><td>

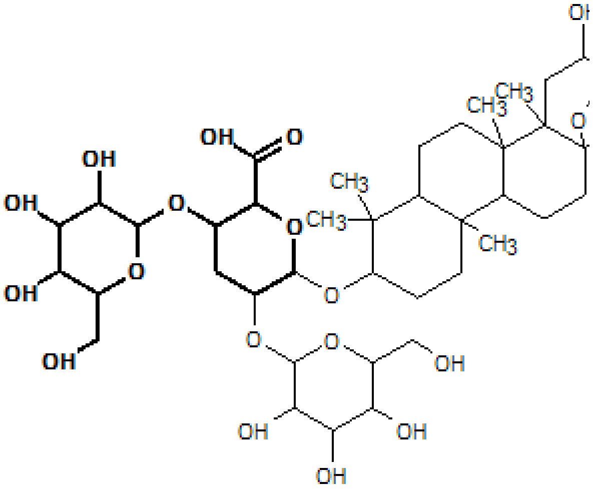

Selected:	C12H18O9
Mass:	306.0951
Broken Bonds:	2
Delta H:	0
Score:	36.5

Contained Neutral Losses

Mass	Formula

</td></tr>
</table>

Fragment Details	Structure Details

164.0687	C6H12O5
162.0530	C6H10O5
146.0581	C6H10O4
144.0427	C6H8O4

Mass (m/z):	325.1175
Ion Formula:	C12H21O10
Error (ppm):	10.8
Intensity (cps):	21.8
RDB:	2.0
Score:	36.5

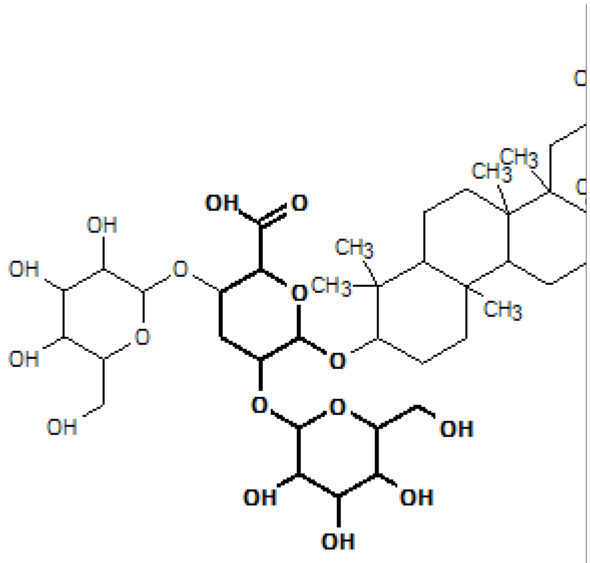

Selected:	C12H18O10
Mass:	322.0900
Broken Bonds:	2
Delta H:	2
Score:	36.5

Contained Neutral Losses

Mass	Formula
182.0807	C6H14O6
180.0650	C6H12O6
164.0701	C6H12O5
162.0547	C6H10O5
18.0120	H2O

Mass (m/z):	457.3728
Ion Formula:	C30H49O3
Error (ppm):	9.0
Intensity (cps):	91.6
RDB:	6.0
Score:	40.5

<table>
<tr><th>Fragment Details</th><th>Structure Details</th></tr>
<tr><td></td><td>
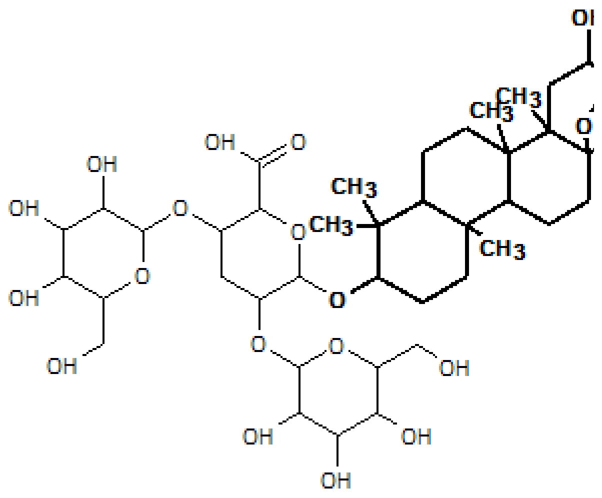
<table>
<tr><td>Selected:</td><td>C30H49O3</td></tr>
<tr><td>Mass:</td><td>457.3682</td></tr>
<tr><td>Broken Bonds:</td><td>1</td></tr>
<tr><td>Delta H:</td><td>0</td></tr>
<tr><td>Score:</td><td>40.5</td></tr>
</table>
Contained Neutral Losses
No contained neutral losses
</td></tr>
<tr><td>
<table>
<tr><td>Mass (m/z):</td><td>571.4055</td></tr>
<tr><td>Ion Formula:</td><td>C35H55O6</td></tr>
<tr><td>Error (ppm):</td><td>8.9</td></tr>
<tr><td>Intensity (cps):</td><td>24.9</td></tr>
<tr><td>RDB:</td><td>8.0</td></tr>
<tr><td>Score:</td><td>29.5</td></tr>
</table>
</td><td>
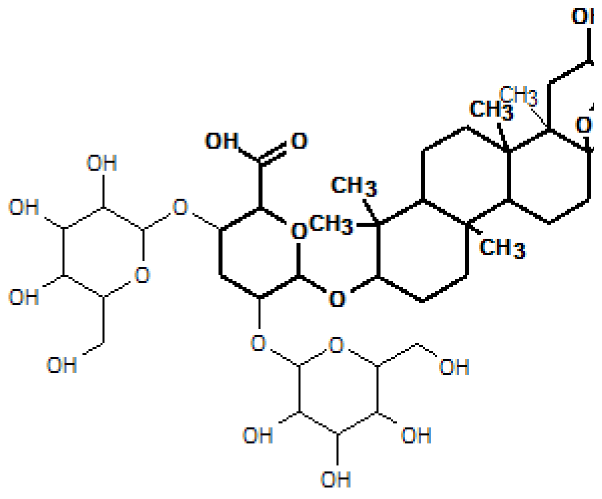
<table>
<tr><td>Selected:</td><td>C35H53O6</td></tr>
<tr><td>Mass:</td><td>569.3842</td></tr>
<tr><td>Broken Bonds:</td><td>3</td></tr>
<tr><td>Delta H:</td><td>0</td></tr>
<tr><td>Score:</td><td>29.5</td></tr>
</table>
Contained Neutral Losses
<table>
<tr><th>Mass</th><th>Formula</th></tr>
<tr><td>428.3687</td><td>C29H48O2</td></tr>
<tr><td>426.3530</td><td>C29H46O2</td></tr>
</table>
</td></tr>
<tr><td>
<table>
<tr><td>Mass (m/z):</td><td>597.3869</td></tr>
</table>
</td><td></td></tr>
</table>

Fragment Details	Structure Details
Ion Formula: C36H53O7<br>Error (ppm): 12.1<br>Intensity (cps): 91.8<br>RDB: 10.0<br>Score: 36.0	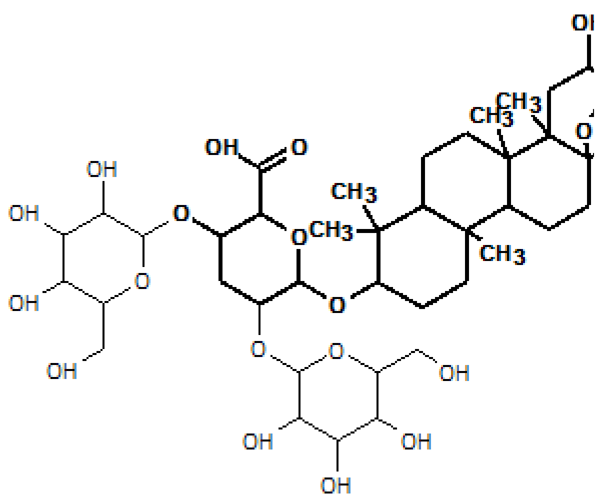<br>Selected: C36H56O7<br>Mass: 600.4026<br>Broken Bonds: 2<br>Delta H: -4<br>Score: 36.0

Contained Neutral Losses

Mass	Formula
458.3817	C30H50O3
454.3501	C30H46O3
440.3709	C30H48O2
140.0141	C6H4O4

Fragment Details	Structure Details
Mass (m/z): 615.3976<br>Ion Formula: C36H55O8<br>Error (ppm): 11.9<br>Intensity (cps): 289.7<br>RDB: 9.0<br>Score: 36.5	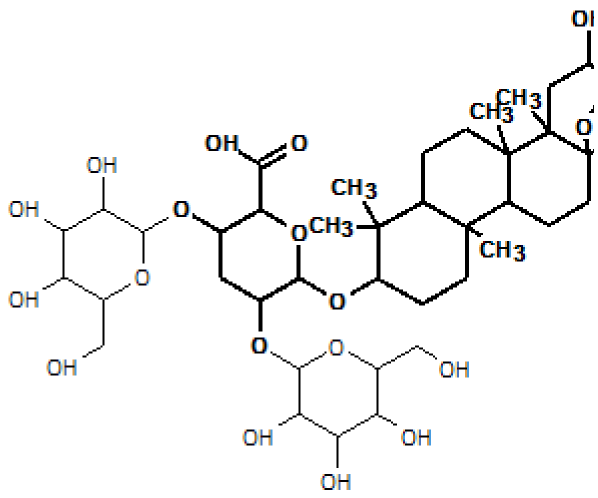<br>Selected: C36H56O8<br>Mass: 616.3975<br>Broken Bonds: 2<br>Delta H: -2<br>Score: 36.5

Contained Neutral Losses

Mass	Formula

Fragment Details	Structure Details

458.3816	C30H50O3
454.3501	C30H46O3
158.0248	C6H6O5
43.9921	CO2
18.0107	H2O

Mass (m/z):	733.4612
Ion Formula:	C41H65O11
Error (ppm):	10.8
Intensity (cps):	144.8
RDB:	9.0
Score:	33.5

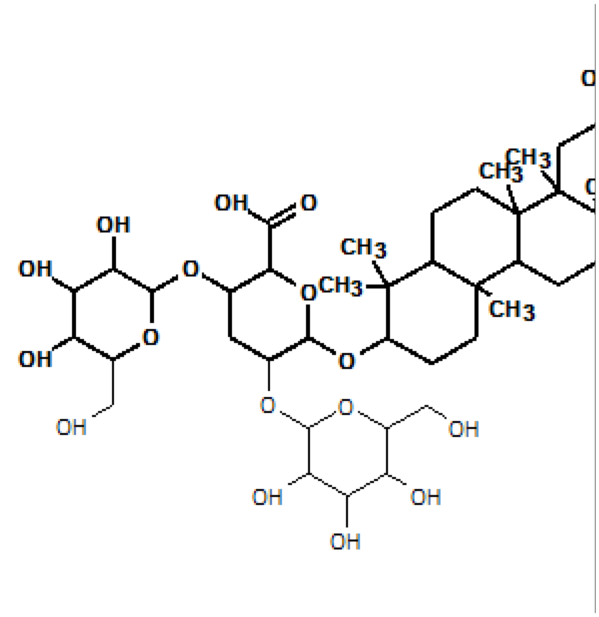

Selected:	C41H64O11
Mass:	732.4449
Broken Bonds:	2
Delta H:	0
Score:	33.5

Contained Neutral Losses

Mass	Formula
602.4252	C36H58O7
276.0884	C11H16O8
136.0743	C5H12O4

Mass (m/z):	751.4734
Ion Formula:	C41H67O12
Error (ppm):	12.8
Intensity (cps):	37.8
RDB:	8.0
Score:	33.5

Fragment Details	Structure Details

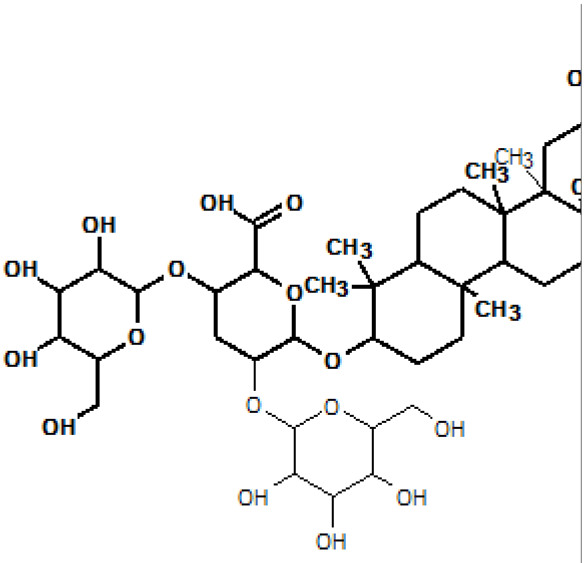

Selected:	C41H64O12
Mass:	748.4398
Broken Bonds:	2
Delta H:	2
Score:	33.5

Contained Neutral Losses

Mass	Formula
590.4260	C35H58O7
588.4107	C35H56O7
444.3679	C29H48O3
426.3559	C29H46O2
180.0679	C6H12O6
18.0123	H2O

Mass (m/z):	795.4616
Ion Formula:	C42H67O14
Error (ppm):	10.0
Intensity (cps):	53.4
RDB:	9.0
Score:	25.0

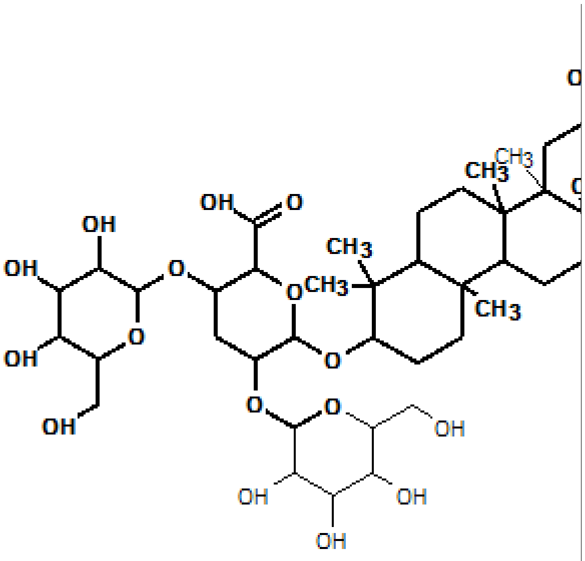

Selected:	C42H65O14
Mass:	793.4374
Broken Bonds:	3
Delta H:	0
Score:	25.0

Fragment Details	Structure Details

Contained Neutral Losses

Mass	Formula
634.4142	C36H58O9
632.3988	C36H56O9
180.0640	C6H12O6
162.0571	C6H10O5
43.9882	CO2

Mass (m/z):	879.5202
Ion Formula:	C47H75O15
Error (ppm):	10.3
Intensity (cps):	274.6
RDB:	10.0
Score:	33.5

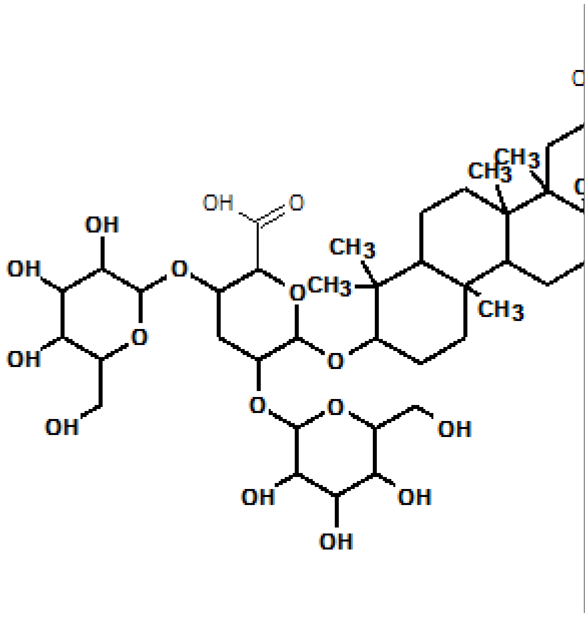

Selected:	C47H76O15
Mass:	880.5184
Broken Bonds:	2
Delta H:	-2
Score:	33.5

Contained Neutral Losses

Mass	Formula
722.5042	C41H70O10
718.4728	C41H66O10
716.4574	C41H64O10

Mass (m/z):	897.5323
Ion Formula:	C47H77O16
Error (ppm):	11.8
Intensity (cps):	22.0
RDB:	9.0
Score:	37.5

Fragment Details	Structure Details

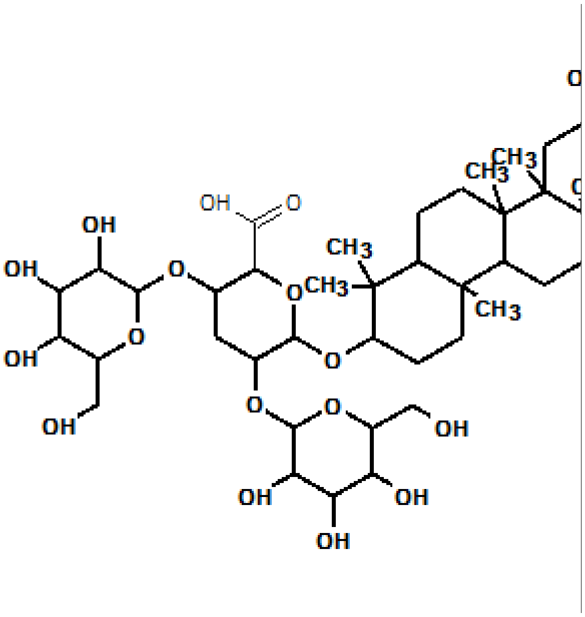

Se-lected:	C47H77O16
Mass:	897.5212
Broken Bonds:	1
Delta H:	0
Score:	37.5

Contained Neutral Losses

Mass	Formula
736.4848	C41H68O11
734.4695	C41H66O11
440.1595	C17H28O13
164.0711	C6H12O5
18.0121	H2O

Mass (m/z):	923.5115
Ion Formula:	C48H75O17
Error (ppm):	11.4
Intensity (cps):	593.9
RDB:	11.0
Score:	40.0

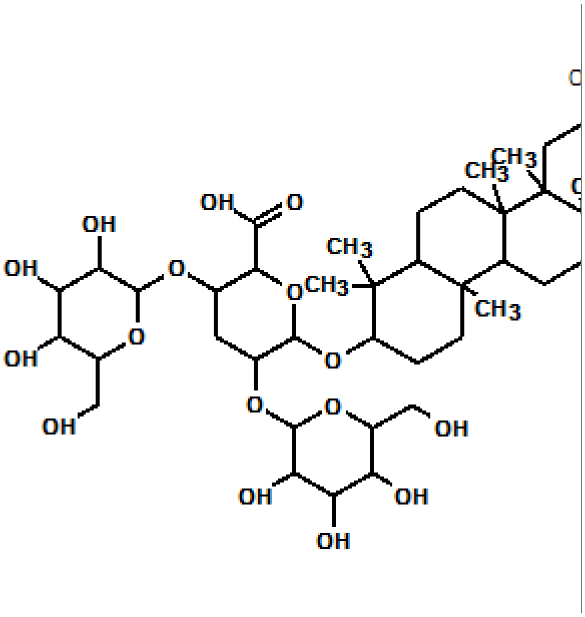

Se-lected:	C48H77O17
Mass:	925.5161
Broken Bonds:	1
Delta H:	-2
Score:	40.0

Contained Neutral Losses

Fragment Details	Structure Details

Mass	Formula
766.4955	C42H70O12
762.4641	C42H66O12
760.4488	C42H64O12
43.9913	CO2

Mass (m/z):	924.5160
Ion Formula:	C48H76O17
Error (ppm):	7.8
Intensity (cps):	54.0
RDB:	10.5
Score:	37.0

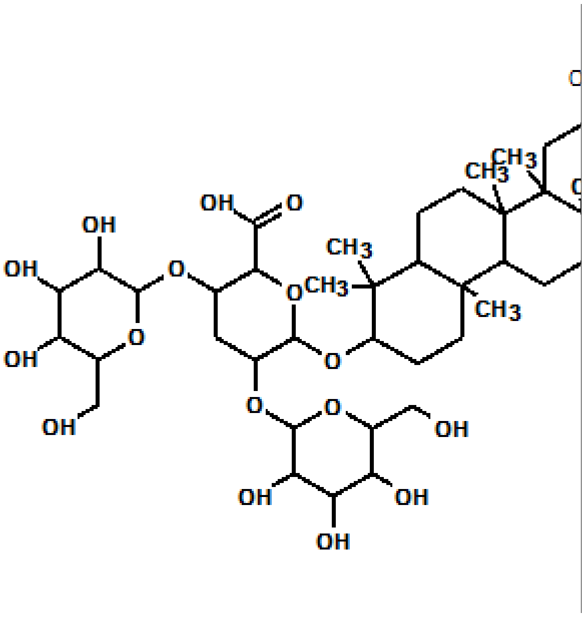

Selected:	C48H77O17
Mass:	925.5161
Broken Bonds:	1
Delta H:	-2
Score:	37.0

Contained Neutral Losses

No contained neutral losses

Mass (m/z):	941.5188
Ion Formula:	C48H77O18
Error (ppm):	7.7
Intensity (cps):	20002.9
RDB:	10.0
Score:	44.0

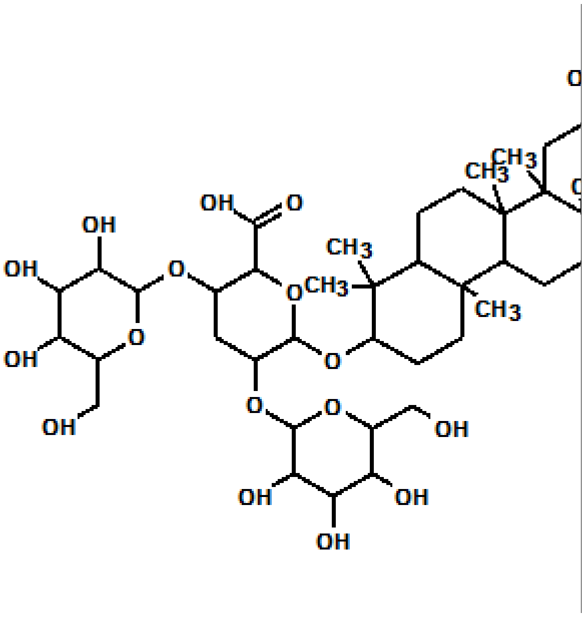

Selected:	C48H78O18
Mass:	942.5188
Broken Bonds:	0
Delta H:	0
Score:	44.0

Fragment Details	Structure Details
	**Contained Neutral Losses**<br><table><tr><th>Mass</th><th>Formula</th></tr><tr><td>780.4713</td><td>C42H68O13</td></tr><tr><td>778.4560</td><td>C42H66O13</td></tr><tr><td>484.1460</td><td>C18H28O15</td></tr><tr><td>43.9865</td><td>CO2</td></tr><tr><td>18.0073</td><td>H2O</td></tr></table>

Interpretation of the metabolite in rat (M17)

M17 – Oxidation [M-H]-

Formula:	C60H98O28
ppm:	6.3
RDB:	12.0

Available Structure Candidates

Rank	Score	Count
1	NaN	28

Applied Metabolite Structure

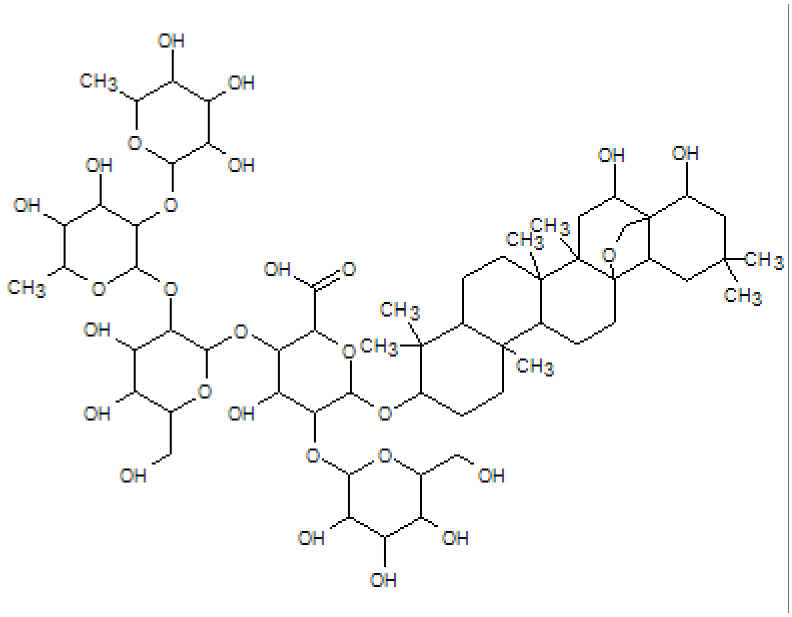

Composition:	C60H98O28
Mass:	1266.6245

Metabolite MS/MS

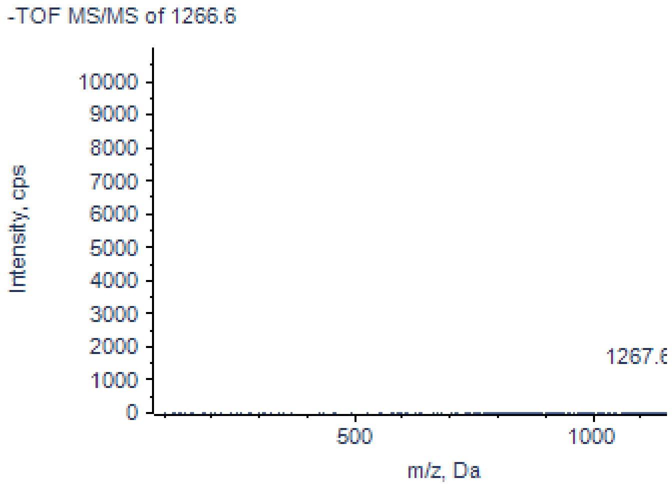

Parent MS/MS

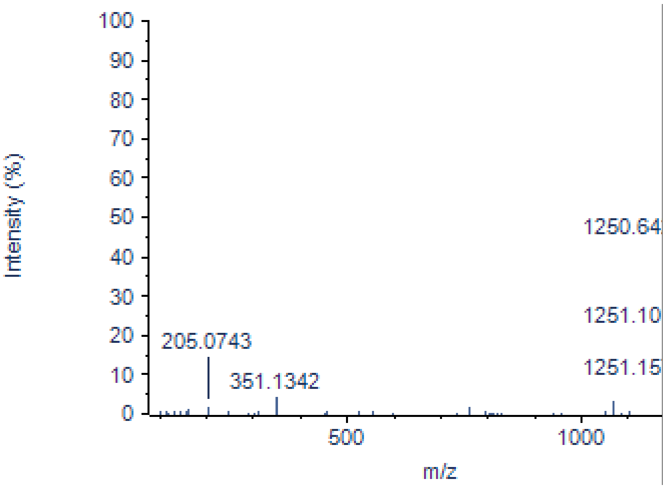

Peaks selected for assignment (m/z): 101.0236, 143.0348, 179.0551, 205.0739, 301.0585, 367.1291, 367.1533, 457.3617, 759.4238, 822.5806, 847.3978, 972.2022, 1085.5661, 1085.5941, 1086.5492, 1152.9982, 1248.5979, 1262.3174, 1262.4371, 1262.5572, 1263.7996, 1265.1758, 1265.2758, 1265.3316, 1265.6344, 1266.1416, 1266.2376, 1266.3621, 1266.5042, 1266.6303

Assigned: 3 of 30 peaks, score for 3 proposed assignments: 110.5

Metabolite Options

Number of fragment peaks selected for assignment:	30
Minimum signal-to-noise ratio:	3
MS/MS m/z tolerance:	15 ppm
Fragmentation Settings	
Break aromatic rings:	True
Maximum number of bonds to break:	4
Maximum number of C-C bonds to break:	4
Label Settings	
Label peaks with:	Ion with ppm Error

Fragment Filters

Mass Range (m/z)	300 To 1500

Fragment Details	Structure Details
Mass (m/z): 367.1291<br>Ion Formula: C14H23O11<br>Error (ppm): 12.4	

Fragment Details	Structure Details
Intensity (cps): 38.5<br>RDB: 3.0	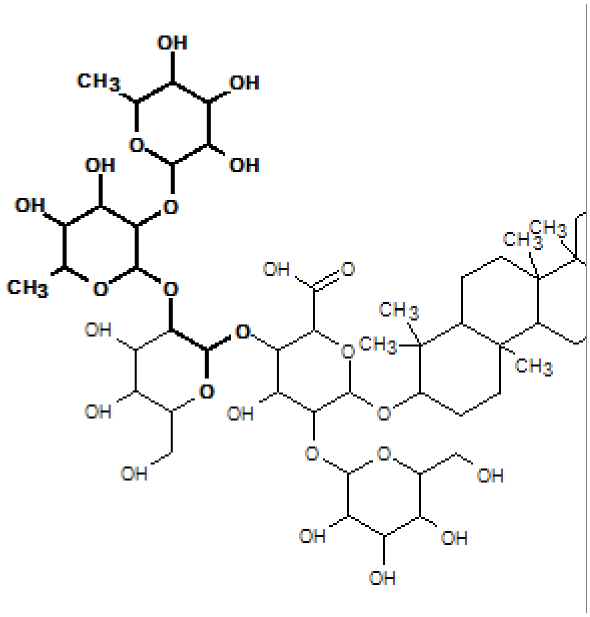<br>Selected: C14H23O11<br>Mass: 367.1240<br>Broken Bonds: 3<br>Delta H: -2<br>**Contained Neutral Losses**<br>No contained neutral losses<br>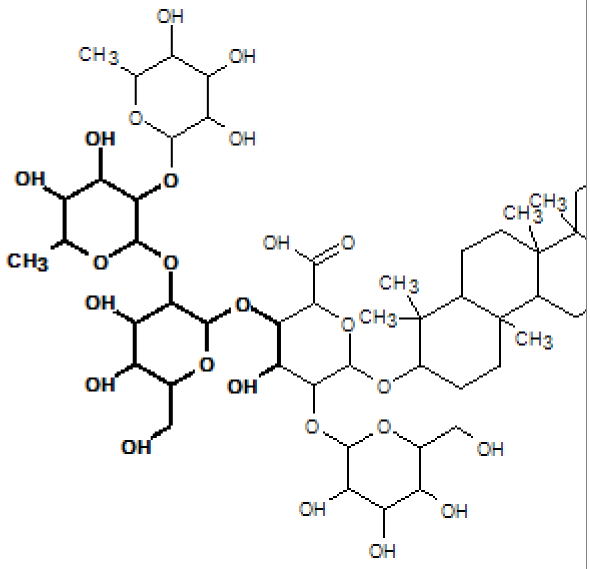<br>Selected: C14H23O11<br>Mass: 367.1240<br>Broken Bonds: 3<br>Delta H: -2<br>**Contained Neutral Losses**<br>No contained neutral losses
Mass (m/z): 1248.5979<br>Ion Formula: C60H96O27<br>Error (ppm): -13.3<br>Intensity (cps): 28.6	

Fragment Details	Structure Details
RDB: 12.5	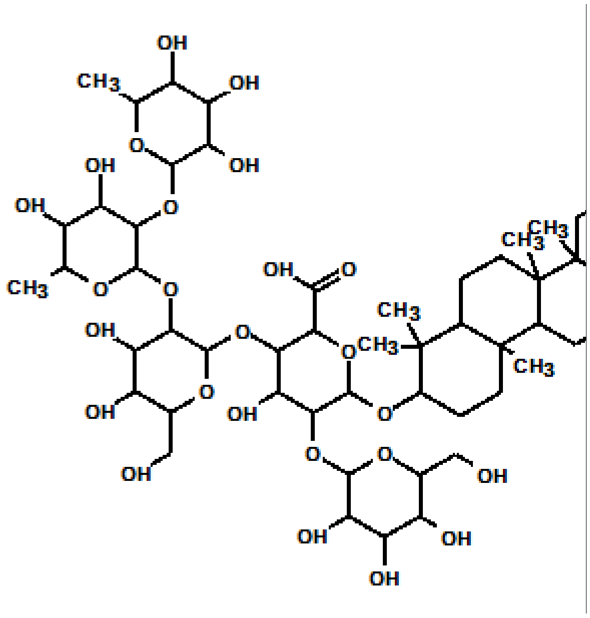<br>Selected: C60H97O27<br>Mass: 1249.6217<br>Broken Bonds: 1<br>Delta H: -2<br>**Contained Neutral Losses**<br>No contained neutral losses
Mass (m/z): 1265.6344<br>Ion Formula: C60H97O28<br>Error (ppm): 13.6<br>Intensity (cps): 460.7<br>RDB: 12.0	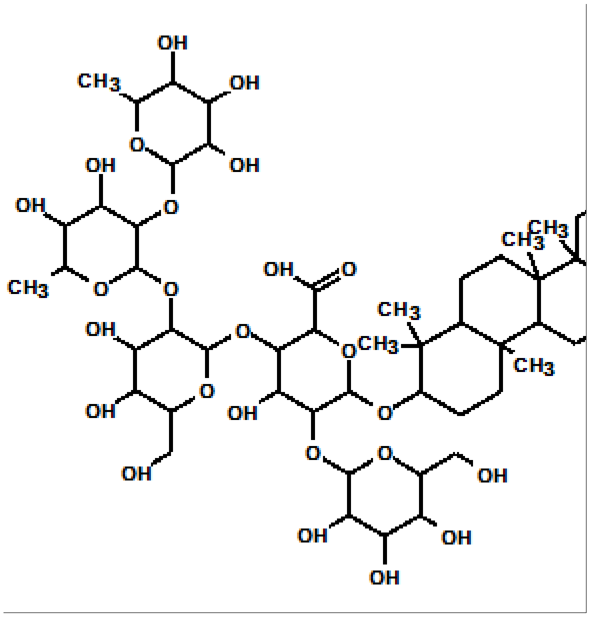<br>Selected: C60H98O28<br>Mass: 1266.6245<br>Broken Bonds: 0<br>Delta H: 0<br>**Contained Neutral Losses**<br>No contained neutral losses

Interpretation of the metabolite in rat (M0)

M0– Parent [M-H]-

Formula:	C60H98O27
ppm:	3.0
RDB:	12.0

Available Structure Candidates

Rank	Score	Count
1	100.0	1

Applied Metabolite Structure

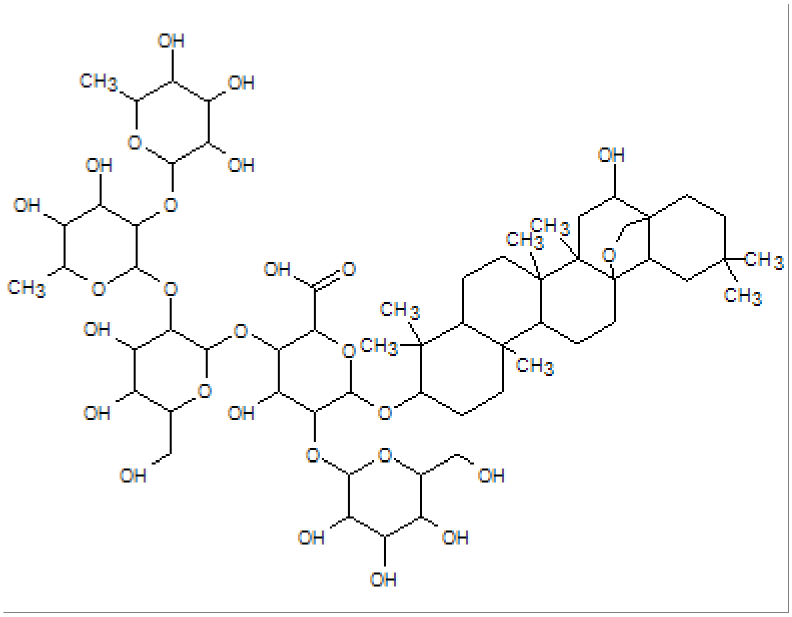

Composition:	C60H98O27
Mass:	1250.6295

Metabolite MS/MS

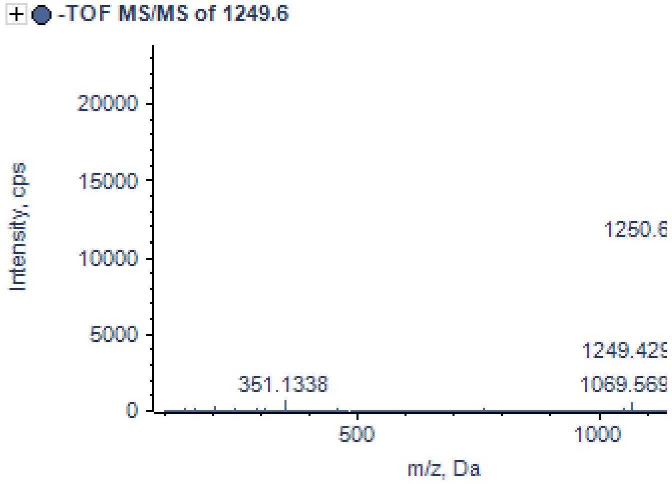

Parent MS/MS

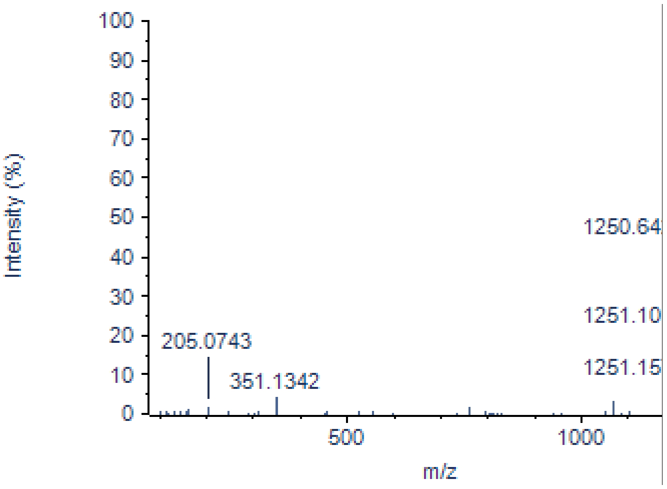

Common product ions (m/z): 1069.5696, 1249.6404, 1250.6386

Peaks selected for assignment (m/z): 101.0256, 113.0264, 115.0418, 119.0363, 143.0371, 163.0632, 205.0735, 247.0849, 291.1112, 301.0589, 309.1224, 351.1338, 455.3567, 457.3741, 553.3947, 733.4568, 759.4418, 1051.5615, 1069.5696, 1103.5783, 1231.6261, 1245.4698, 1245.6570, 1246.3711, 1247.0064, 1247.2636, 1247.5178, 1247.5620, 1247.6078, 1249.6404

Assigned: 21 of 30 peaks, score for 21 proposed assignments: 771.0

Metabolite Options

Number of fragment peaks selected for assignment:	30
Minimum signal-to-noise ratio:	3
MS/MS m/z tolerance:	15 ppm
Fragmentation Settings	
Break aromatic rings:	True
Maximum number of bonds to break:	4
Maximum number of C-C bonds to break:	4
Label Settings	
Label peaks with:	Ion with ppm Error

Fragment Filters

Mass Range (m/z)	300 To 1500
Fragments with assigned structures	True

Fragment Details	Structure Details
Mass (m/z): 301.0589<br>Ion Formula: C12H13O9<br>Error (ppm): 7.9<br>Intensity (cps): 64.1<br>RDB: 6.0	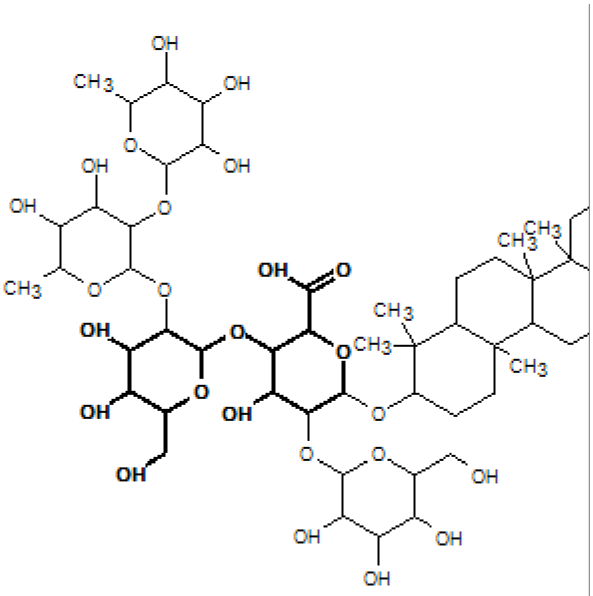<br>Selected: C12H17O9<br>Mass: 305.0873<br>Broken Bonds: 3<br>Delta H: -6<br>**Contained Neutral Losses**

Fragment Details	Structure Details

Mass	Formula
158.0218	C6H6O5
137.9957	C6H2O4

Mass (m/z):	309.1224
Ion Formula:	C12H21O9
Error (ppm):	10.8
Intensity (cps):	143.6
RDB:	2.0

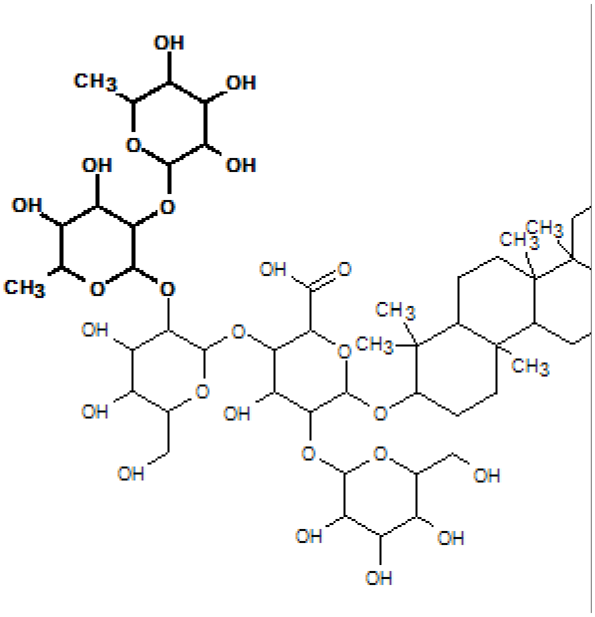

Selected:	C12H21O9
Mass:	309.1186
Broken Bonds:	1
Delta H:	0

Contained Neutral Losses

Mass	Formula
166.0854	C6H14O5
146.0593	C6H10O4
18.0112	H2O

Mass (m/z):	351.1338
Ion Formula:	C14H23O10
Error (ppm):	11.9
Intensity (cps):	677.7
RDB:	3.0

Fragment Details	Structure Details

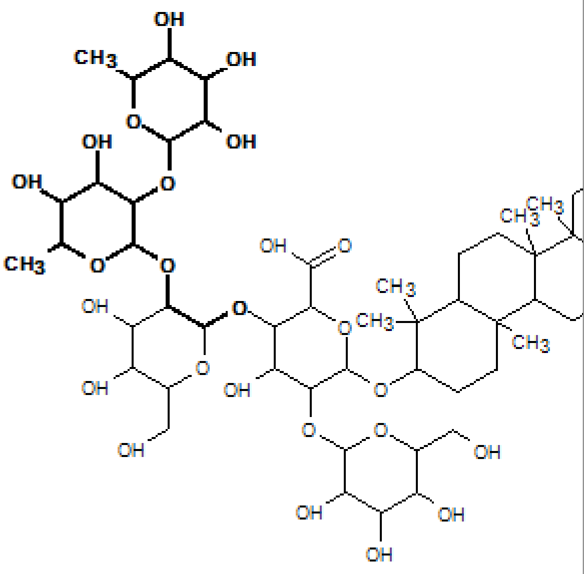

Selected:	C14H23O10
Mass:	351.1291
Broken Bonds:	3
Delta H:	-2

Contained Neutral Losses

Mass	Formula
208.0968	C8H16O6
188.0707	C8H12O5
146.0603	C6H10O4
60.0226	C2H4O2
42.0114	C2H2O

Mass (m/z):	455.3567
Ion Formula:	C30H47O3
Error (ppm):	8.0
Intensity (cps):	85.0
RDB:	7.0

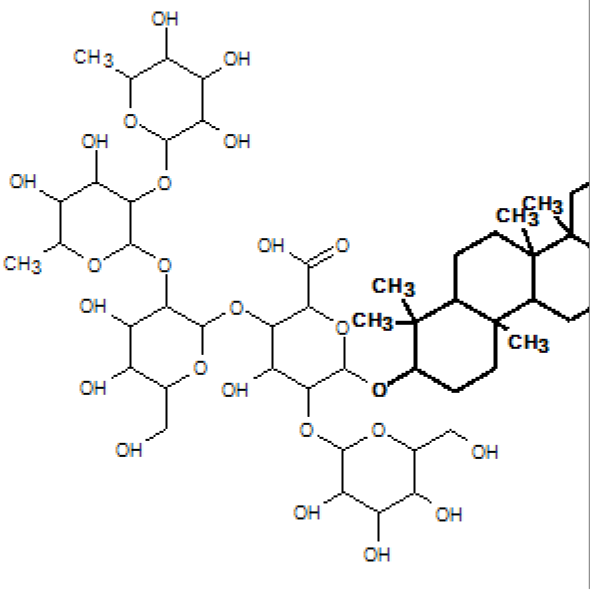

Selected:	C30H49O3
Mass:	457.3682
Broken Bonds:	1
Delta H:	-2

Contained Neutral Losses

Fragment Details	Structure Details
	No contained neutral losses
Mass (m/z): 457.3741<br>Ion Formula: C30H49O3<br>Error (ppm): 11.8<br>Intensity (cps): 126.0<br>RDB: 6.0	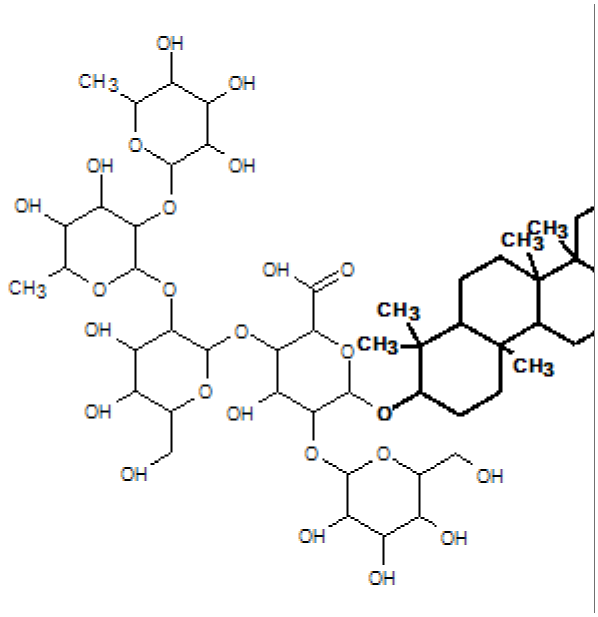<br>Selected: C30H49O3<br>Mass: 457.3682<br>Broken Bonds: 1<br>Delta H: 0<br>**Contained Neutral Losses**<br>No contained neutral losses
Mass (m/z): 553.3947<br>Ion Formula: C35H53O5<br>Error (ppm): 8.9<br>Intensity (cps): 54.8<br>RDB: 9.0	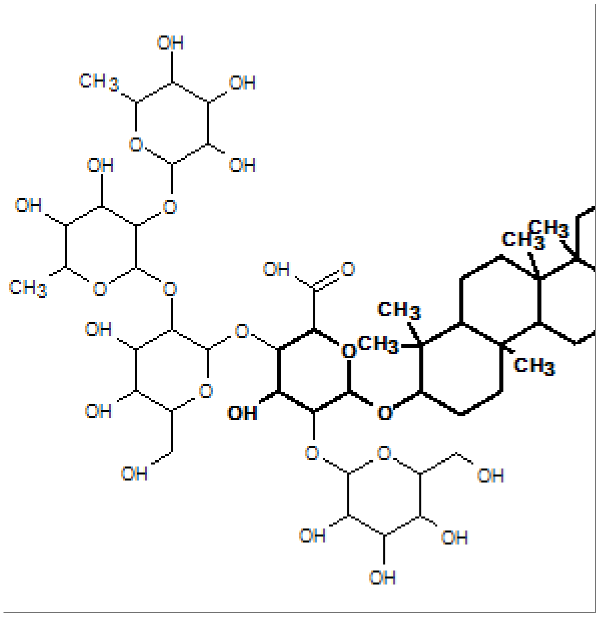<br>Selected: C35H55O5<br>Mass: 555.4050<br>Broken Bonds: 3<br>Delta H: -4<br>**Contained Neutral Losses**<br><table><tr><th>Mass</th><th>Formula</th></tr><tr><td>98.0380</td><td>C5H6O2</td></tr></table>

Fragment Details	Structure Details
	96.0206 | C5H4O2
Mass (m/z): 733.4568<br>Ion Formula: C41H65O11<br>Error (ppm): 4.8<br>Intensity (cps): 36.3<br>RDB: 9.0	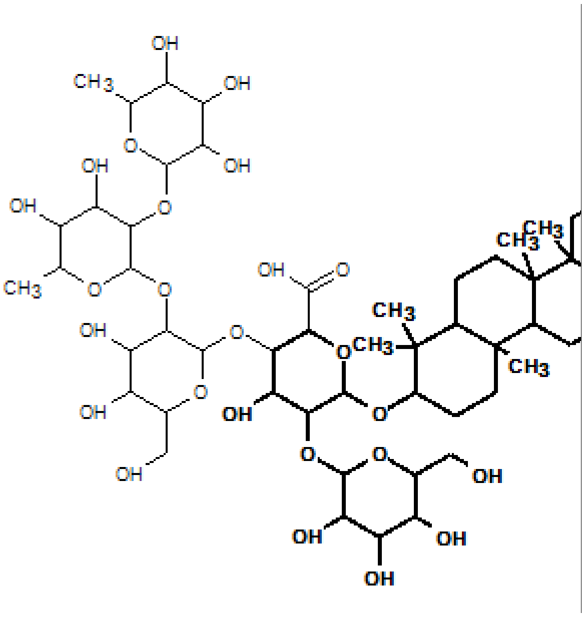<br>Selected: C41H66O11<br>Mass: 734.4605<br>Broken Bonds: 2<br>Delta H: -2
Mass (m/z): 759.4418<br>Ion Formula: C42H63O12<br>Error (ppm): 12.3<br>Intensity (cps): 197.5<br>RDB: 11.0	

Contained Neutral Losses

Mass	Formula
570.3936	C35H54O6
278.1001	C11H18O8
276.0827	C11H16O8
180.0620	C6H12O6

<table>
<tr><th>Fragment Details</th><th>Structure Details</th></tr>
<tr>
<td></td>
<td>
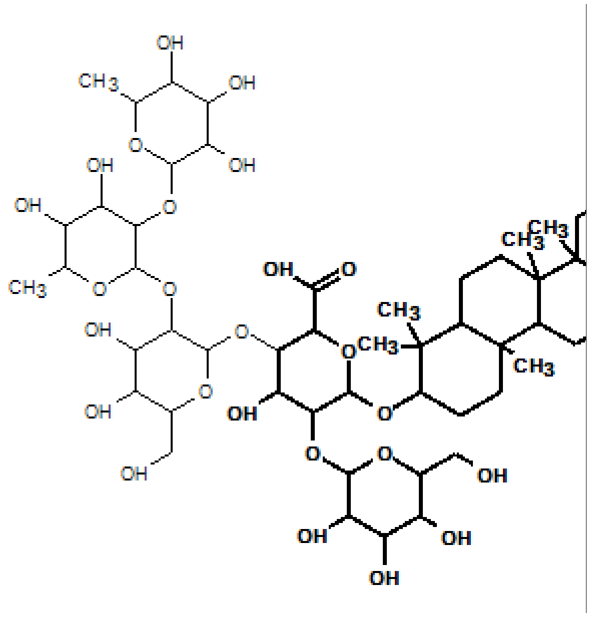

Selected: C42H66O12
Mass: 762.4554
Broken Bonds: 2
Delta H: -4

Contained Neutral Losses

No contained neutral losses
</td>
</tr>
<tr>
<td>
Mass (m/z): 1051.5615
Ion Formula: C54H83O20
Error (ppm): 12.6
Intensity (cps): 75.7
RDB: 13.0
</td>
<td>
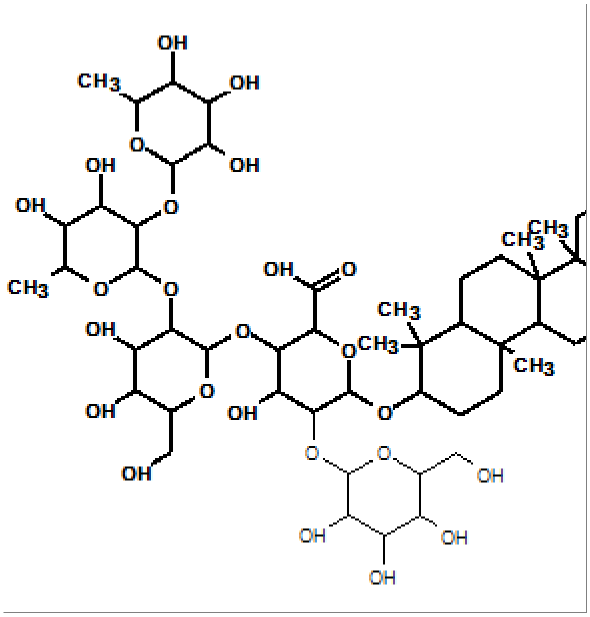

Selected: C54H86O20
Mass: 1054.5712
Broken Bonds: 2
Delta H: -4

Contained Neutral Losses

<table>
<tr><th>Mass</th><th>Formula</th></tr>
<tr><td>908.5245</td><td>C48H76O16</td></tr>
<tr><td>888.4984</td><td>C48H72O15</td></tr>
<tr><td>760.4504</td><td>C42H64O12</td></tr>
<tr><td>750.5027</td><td>C42H70O11</td></tr>
</table>
</td>
</tr>
</table>

Fragment Details	Structure Details
	742.4391 — C42H62O11<br>292.1197 — C12H20O8
Mass (m/z): 1069.5696<br>Ion Formula: C54H85O21<br>Error (ppm): 10.0<br>Intensity (cps): 601.4<br>RDB: 12.0	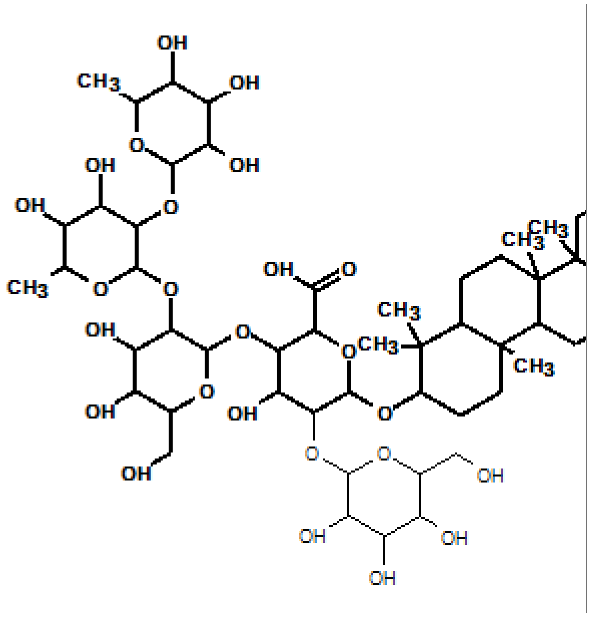<br>Selected: C54H87O21<br>Mass: 1071.5740<br>Broken Bonds: 1<br>Delta H: -2
Mass (m/z): 1103.5783<br>Ion Formula: C54H87O23<br>Error (ppm): 12.7<br>Intensity (cps): 53.0<br>RDB: 11.0	

Contained Neutral Losses

Mass	Formula
768.5107	C42H72O12
614.2129	C24H38O18
612.1955	C24H36O18
310.1278	C12H22O9
18.0080	H2O

Fragment Details	Structure Details
	(see below)

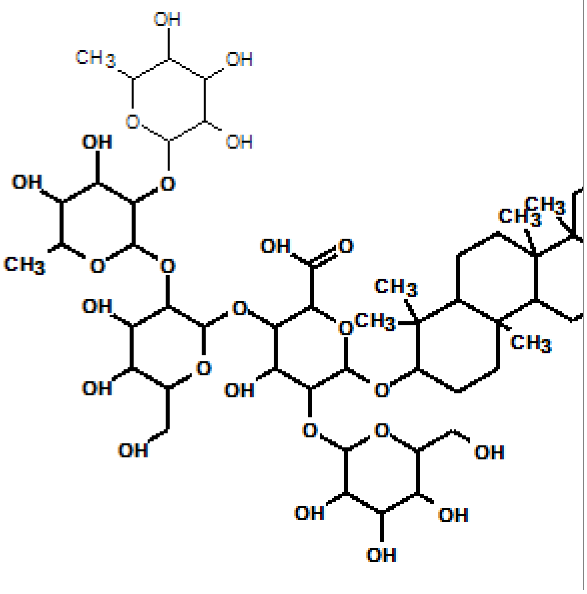

Selected:	C54H87O23
Mass:	1103.5638
Broken Bonds:	1
Delta H:	0

Contained Neutral Losses

Mass	Formula
960.5413	C48H80O19
794.4559	C42H66O14
648.2216	C24H40O20
646.2042	C24H38O20

Mass (m/z):	1231.6261
Ion Formula:	C60H95O26
Error (ppm):	11.7
Intensity (cps):	197.9
RDB:	13.0

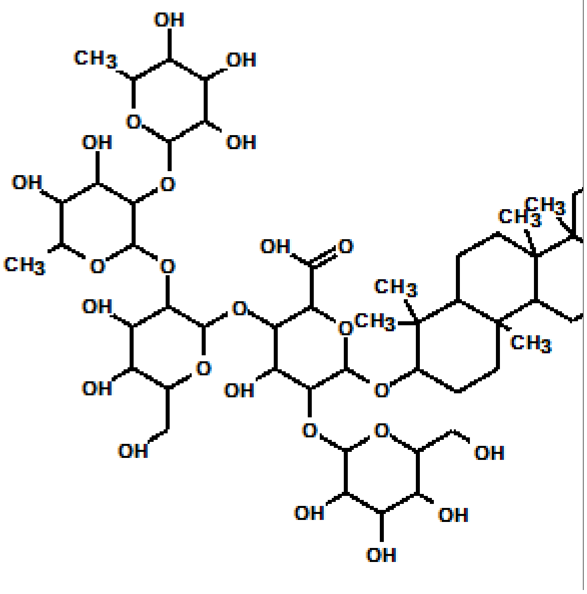

Selected:	C60H97O26
Mass:	1233.6268
Broken Bonds:	1
Delta H:	-2

Contained Neutral Losses

Mass	Formula

Fragment Details	Structure Details

1088.5891	C54H88O22
1068.5629	C54H84O21
940.5149	C48H76O18
930.5672	C48H82O17
922.5037	C48H74O17
472.1843	C18H32O14
180.0646	C6H12O6
162.0565	C6H10O5

Mass (m/z):	1247.6078
Ion Formula:	C60H95O27
Error (ppm):	0.9
Intensity (cps):	42.5
RDB:	13.0

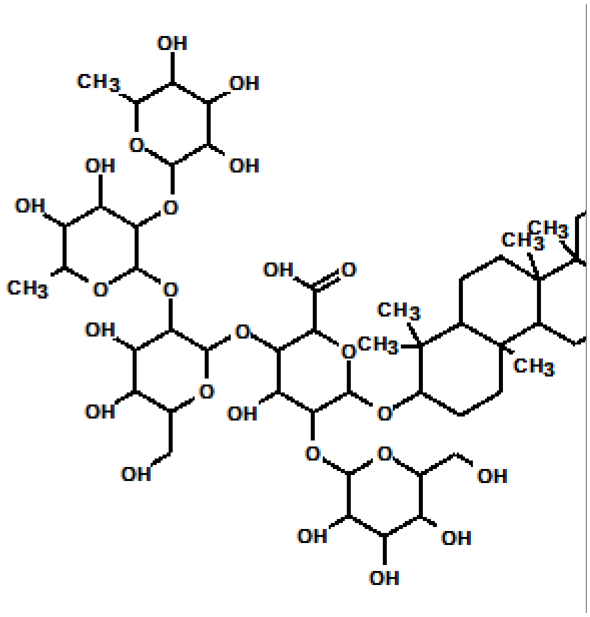

Selected:	C60H98O27
Mass:	1250.6295
Broken Bonds:	0
Delta H:	-2

Contained Neutral Losses

Mass	Formula
1104.5707	C54H88O23
1084.5446	C54H84O22
956.4966	C48H76O19
946.5489	C48H82O18
938.4854	C48H74O18
792.2511	C30H48O24
790.2337	C30H46O24
144.0295	C6H8O4

Mass (m/z):	1249.6404

Fragment Details	
Ion Formula:	C60H97O27
Error (ppm):	14.5
Intensity (cps):	23795.1
RDB:	12.0

Structure Details

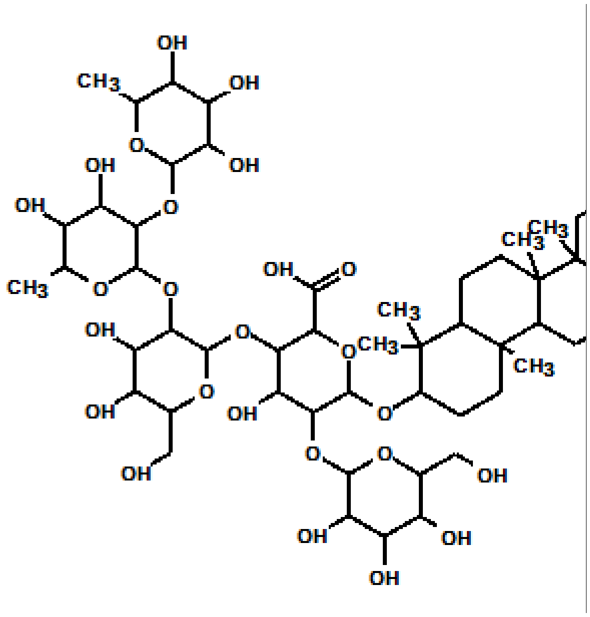

Selected:	C60H98O27
Mass:	1250.6295
Broken Bonds:	0
Delta H:	0

Contained Neutral Losses

Mass	Formula
1106.6034	C54H90O23
1086.5772	C54H86O22
958.5292	C48H78O19
940.5180	C48H76O18
794.2837	C30H50O24
792.2663	C30H48O24
180.0708	C6H12O6
146.0621	C6H10O4
18.0143	H2O

Interpretation of the metabolite in rat (M18)

M18 – Desaturation [M-H]-

Formula:	C60H96O27
ppm:	6.2
RDB:	13.0

Available Structure Candidates

Rank	Score	Count
1	NaN	14

Applied Metabolite Structure

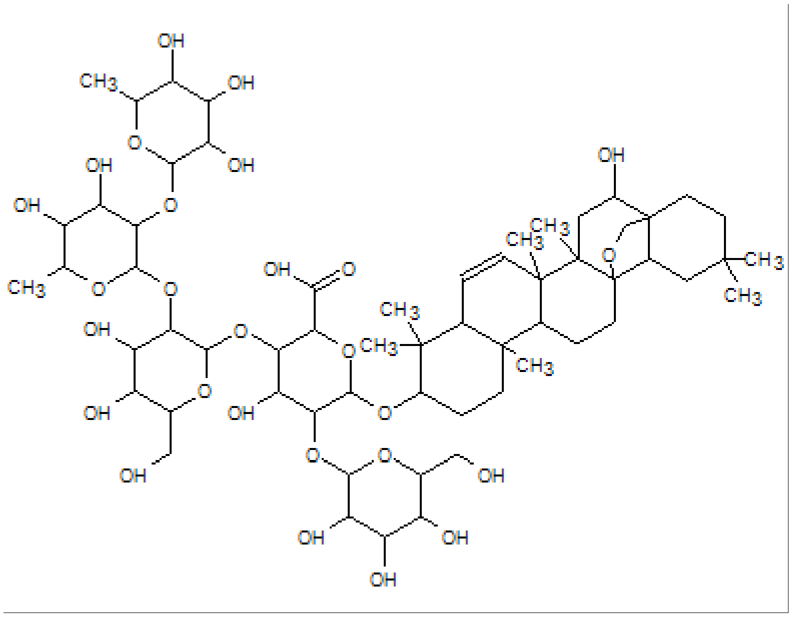

Composition:	C60H96O27
Mass:	1248.6139

Metabolite MS/MS

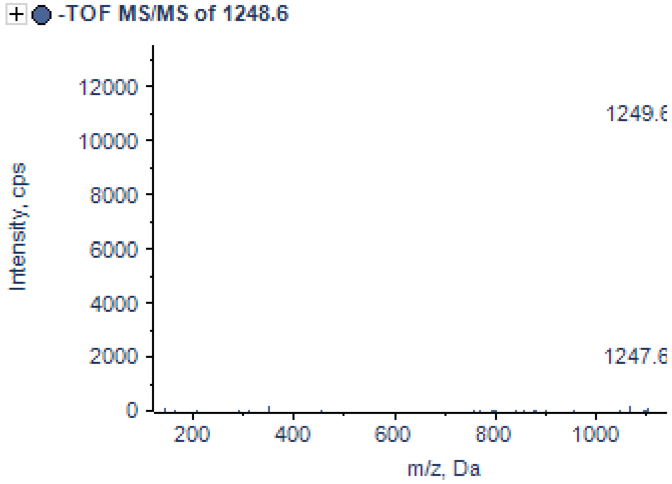

Parent MS/MS

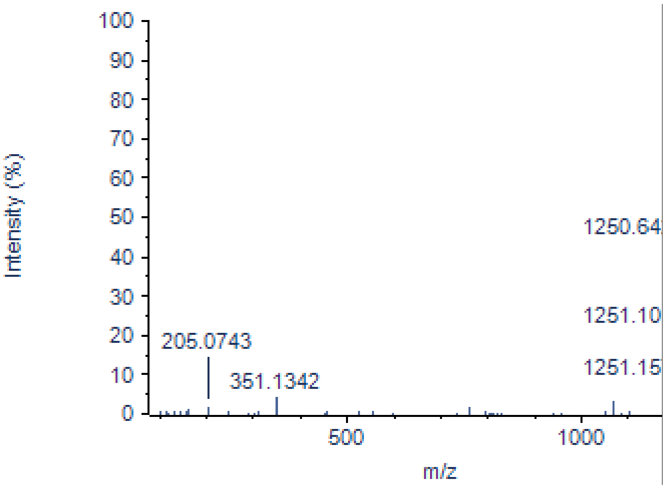

Common product ions (m/z): 1249.6319

Peaks selected for assignment (m/z): 143.0358, 163.0633, 205.0724, 351.1348, 352.1272, 757.4277, 758.4191, 840.0619, 859.1286, 876.2994, 1067.5239, 1068.5653, 1104.5990, 1230.6192, 1230.6424, 1244.3843, 1244.4438, 1244.4806, 1244.5174, 1244.5978, 1247.0877, 1247.6213, 1247.8547, 1248.0084, 1248.2864, 1248.4130, 1248.4695, 1248.6209

Metabolite Options

Number of fragment peaks selected for assignment:	30
Minimum signal-to-noise ratio:	3
MS/MS m/z tolerance:	15 ppm
Fragmentation Settings	
Break aromatic rings:	True
Maximum number of bonds to break:	4
Maximum number of C-C bonds to break:	4
Label Settings	
Label peaks with:	Ion with ppm Error

Fragment Filters

Mass Range (m/z)	300 To 1500

Fragment Details	Structure Details
Mass (m/z): 351.1348<br>Ion Formula: C14H23O10<br>Error (ppm): 14.5<br>Intensity (cps): 140.0<br>RDB: 3.0	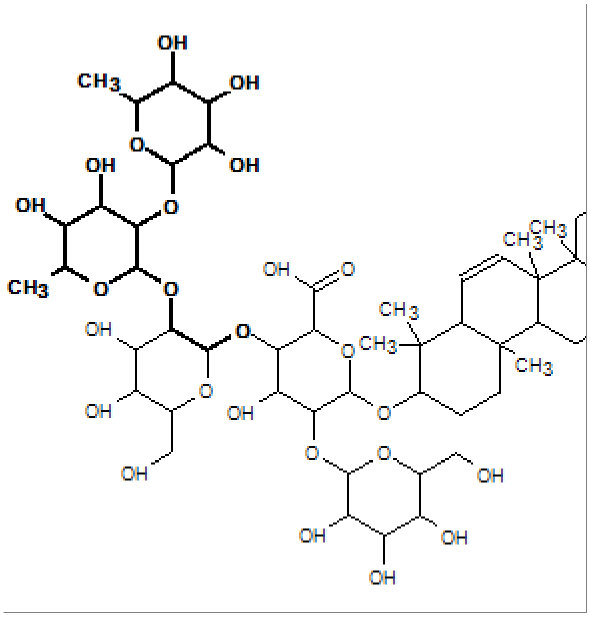<br>Selected: C14H23O10<br>Mass: 351.1291<br>Broken Bonds: 3<br>Delta H: -2<br>**Contained Neutral Losses**<br>Mass: 208.0990 — Formula: C8H16O6
Mass (m/z): 1068.5653	

Fragment Details	Structure Details
Ion Formula: C54H84O21<br>Error (ppm): 13.3<br>Intensity (cps): 140.0<br>RDB: 12.5	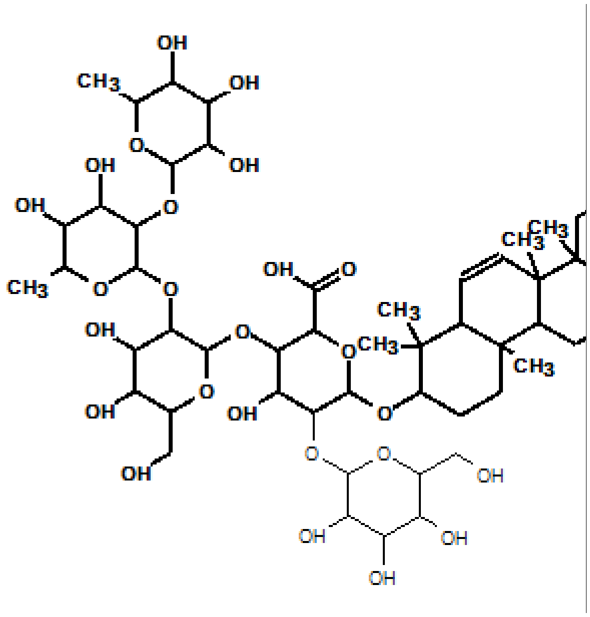<br>Selected: C54H85O21<br>Mass: 1069.5583<br>Broken Bonds: 1<br>Delta H: -2<br>**Contained Neutral Losses**<br>No contained neutral losses
Mass (m/z): 1104.5990<br>Ion Formula: C55H92O22<br>Error (ppm): -8.6<br>Intensity (cps): 60.0<br>RDB: 9.5	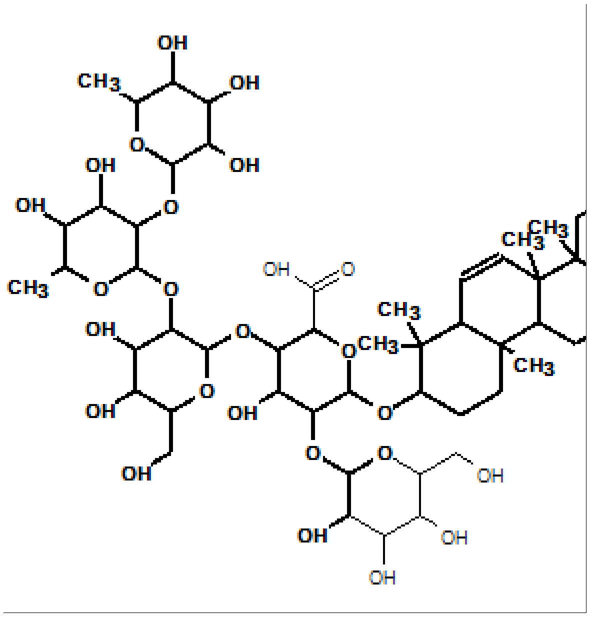<br>Selected: C55H87O22<br>Mass: 1099.5689<br>Broken Bonds: 3<br>Delta H: 2<br>**Contained Neutral Losses**<br>No contained neutral losses
Mass (m/z): 1230.6192<br>Ion Formula: C60H94O26<br>Error (ppm): 12.5	

Fragment Details	Structure Details
Intensity (cps): 80.0<br>RDB: 13.5	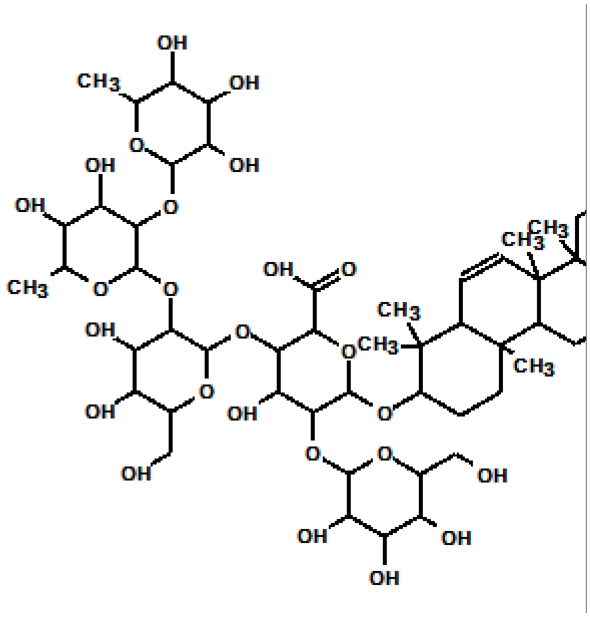<br>Selected: C60H95O26<br>Mass: 1231.6112<br>Broken Bonds: 1<br>Delta H: -2<br>**Contained Neutral Losses**<br>Mass: 162.0539 — Formula: C6H10O5
Mass (m/z): 1247.6213<br>Ion Formula: C60H95O27<br>Error (ppm): 11.8<br>Intensity (cps): 857.0<br>RDB: 13.0	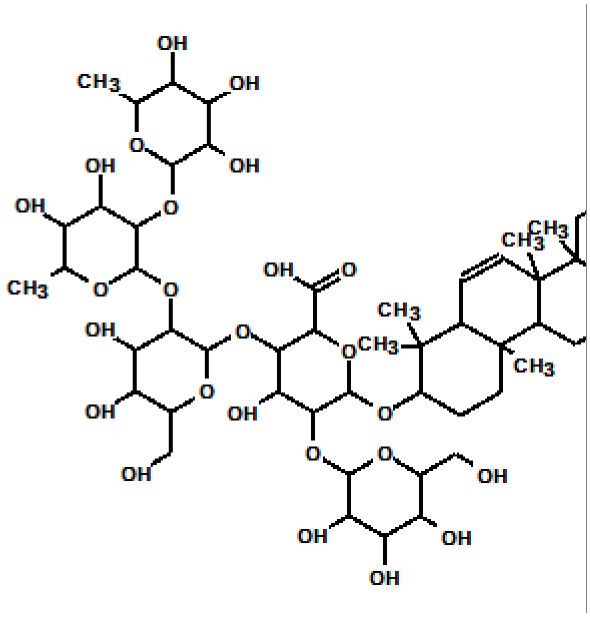<br>Selected: C60H96O27<br>Mass: 1248.6139<br>Broken Bonds: 0<br>Delta H: 0<br>Score: 44.0<br>**Contained Neutral Losses**<br>Mass: 1104.5855 — Formula: C54H88O23

Interpretation of the metabolite in rat (M19)

M19 – Loss of C6H10O4 [M-H]-

Formula: C54H88O23

ppm: 6.6

RDB: 11.0

Available Structure Candidates

Rank	Score	Count
1	100.0	1

Applied Metabolite Structure

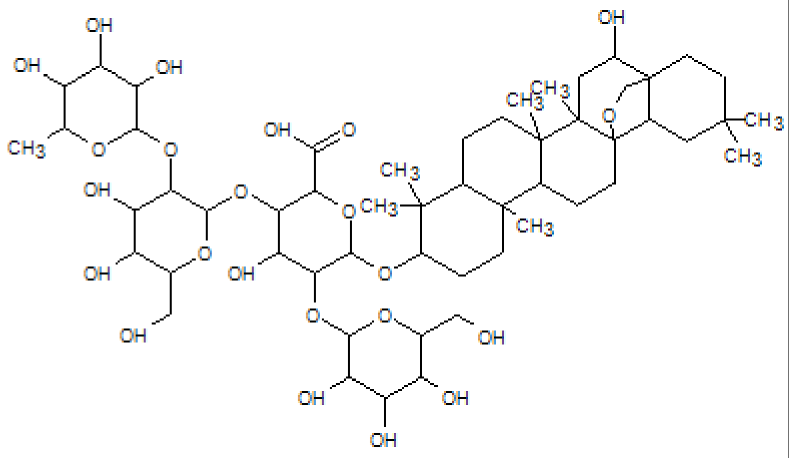

Composition: C54H88O23

Mass: 1104.5716

Metabolite MS/MS

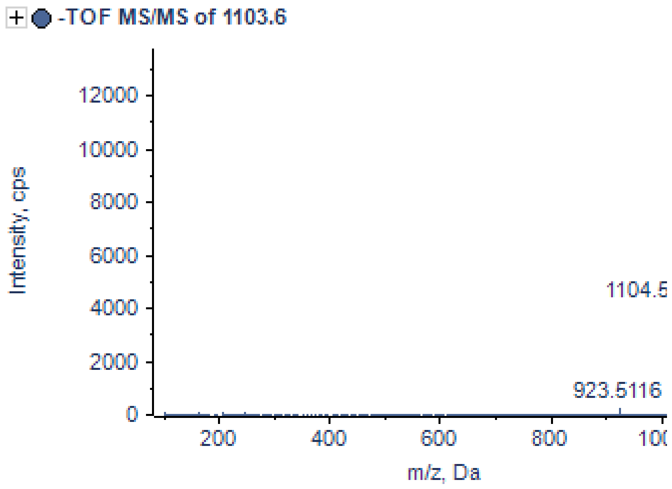

Parent MS/MS

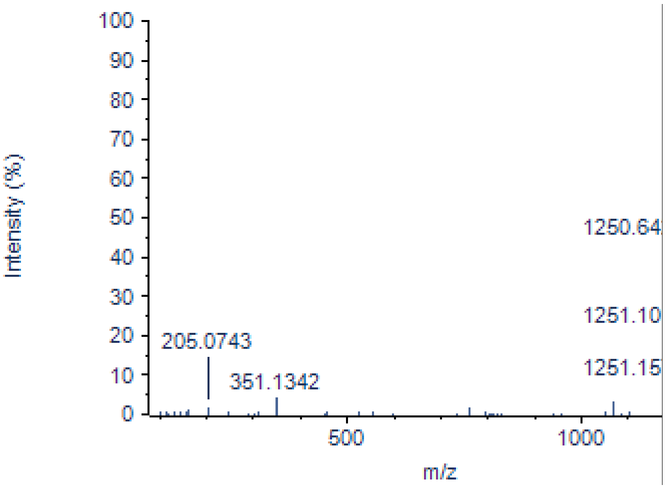

Peaks selected for assignment (m/z): 101.0259, 103.0417, 113.0257, 115.0387, 119.0375, 143.0379, 163.0633, 179.0586, 205.0754, 247.0847, 293.1826, 301.0612, 455.3568, 457.3737, 553.3931, 759.4420, 777.4732, 795.4589, 905.5056, 923.5116, 924.5210, 939.4972, 941.5058, 941.5247, 941.5408, 957.5135, 1099.8516, 1099.9372, 1103.4407, 1103.5732

Metabolite Options

Number of fragment peaks selected for assignment:	30
Minimum signal-to-noise ratio:	3
MS/MS m/z tolerance:	15 ppm
Fragmentation Settings	
Break aromatic rings:	True
Maximum number of bonds to break:	4
Maximum number of C-C bonds to break:	4
Label Settings	
Label peaks with:	Ion with ppm Error

Fragment Filters

Mass Range (m/z)	300 To 1500
Fragments with assigned structures	True

<table>
<tr><th>Fragment Details</th><th>Structure Details</th></tr>
<tr><td>Mass (m/z): 455.3568
Ion Formula: C30H47O3
Error (ppm): 8.1
Intensity (cps): 20.0
RDB: 7.0</td><td>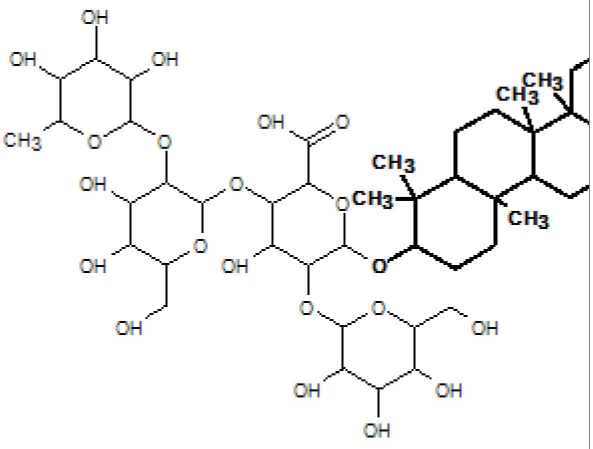
Selected: C30H49O3
Mass: 457.3682
Broken Bonds: 1
Delta H: -2

Contained Neutral Losses
No contained neutral losses</td></tr>
<tr><td>Mass (m/z): 457.3737</td><td></td></tr>
</table>

Fragment Details	Structure Details

Ion Formula: C30H49O3

Error (ppm): 10.8

Intensity (cps): 43.3

RDB: 6.0

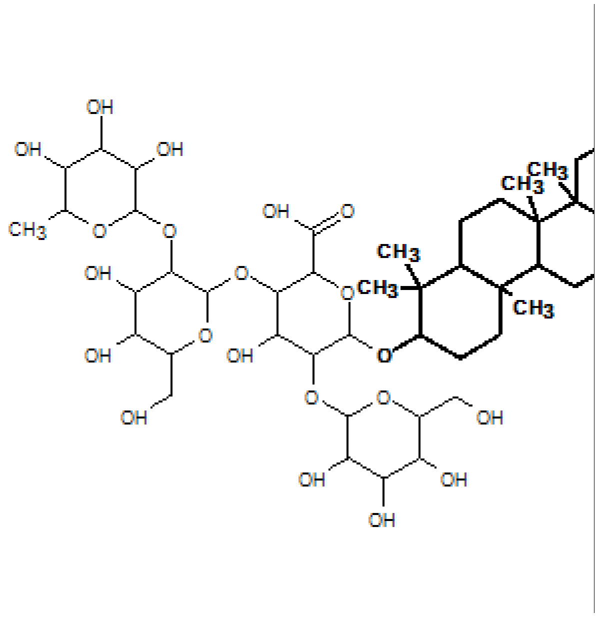

Selected: C30H49O3

Mass: 457.3682

Broken Bonds: 1

Delta H: 0

Contained Neutral Losses

No contained neutral losses

Mass (m/z): 553.3931

Ion Formula: C35H53O5

Error (ppm): 5.9

Intensity (cps): 34.9

RDB: 9.0

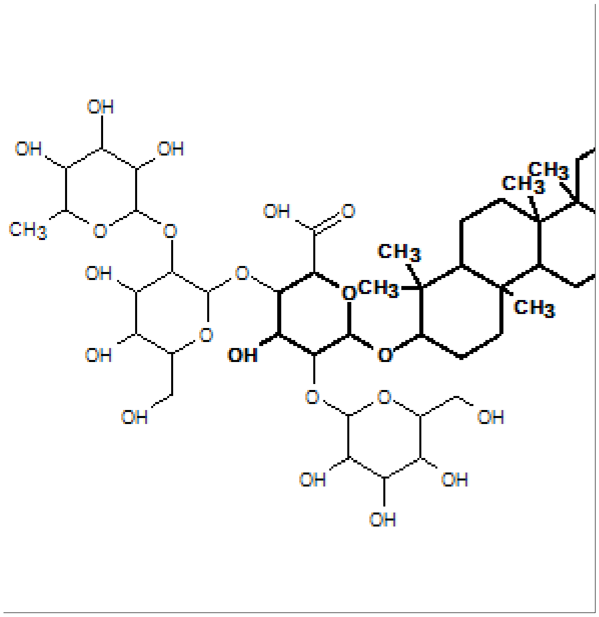

Selected: C35H55O5

Mass: 555.4050

Broken Bonds: 3

Delta H: -4

Contained Neutral Losses

Mass	Formula
440.3674	C30H48O2
438.3544	C30H46O2
98.0364	C5H6O2
96.0195	C5H4O2

<table>
<tr><th>Fragment Details</th><th>Structure Details</th></tr>
<tr><td>Mass (m/z): 759.4420
Ion Formula: C42H63O12
Error (ppm): 12.5
Intensity (cps): 34.3
RDB: 11.0</td><td>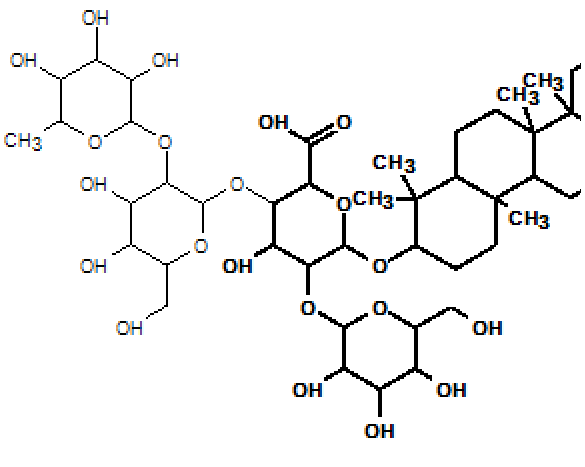
Selected: C42H66O12
Mass: 762.4554
Broken Bonds: 2
Delta H: -4

Contained Neutral Losses
No contained neutral losses</td></tr>
<tr><td>Mass (m/z): 795.4589
Ion Formula: C42H67O14
Error (ppm): 6.6
Intensity (cps): 15.2
RDB: 9.0</td><td>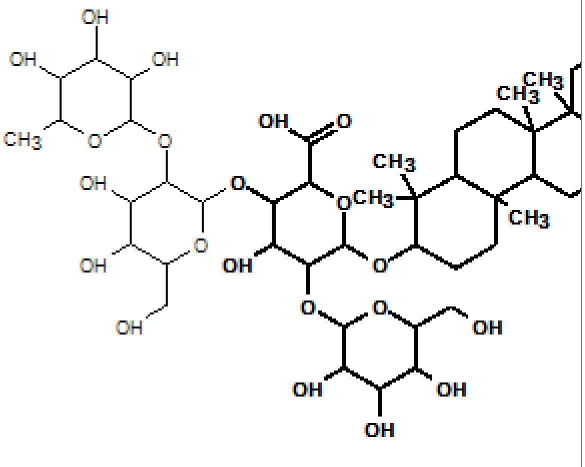
Selected: C42H67O14
Mass: 795.4531
Broken Bonds: 1
Delta H: 0

Contained Neutral Losses
<table><tr><th>Mass</th><th>Formula</th></tr><tr><td>632.3956</td><td>C36H56O9</td></tr><tr><td>616.4002</td><td>C36H56O8</td></tr><tr><td>340.1021</td><td>C12H20O11</td></tr></table></td></tr>
</table>

Fragment Details	Structure Details

338.0852	C12H18O11

Mass (m/z):	923.5116
Ion Formula:	C48H75O17
Error (ppm):	11.5
Intensity (cps):	269.5
RDB:	11.0

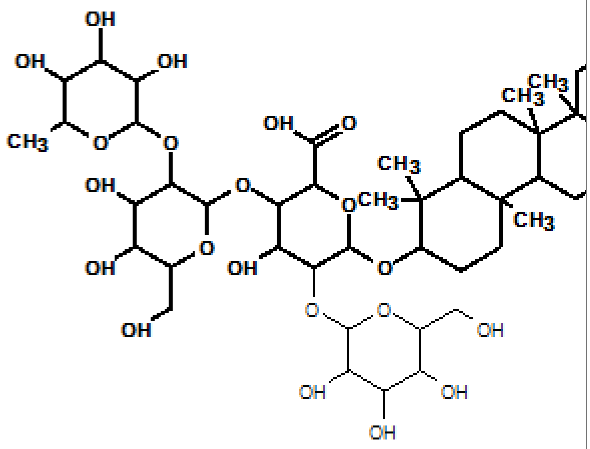

Selected:	C48H77O17
Mass:	925.5161
Broken Bonds:	1
Delta H:	-2

Contained Neutral Losses

Mass	Formula
468.1549	C18H28O14
466.1380	C18H26O14
164.0696	C6H12O5
18.0061	H2O

Mass (m/z):	924.5210
Ion Formula:	C48H76O17
Error (ppm):	13.2
Intensity (cps):	29.0
RDB:	10.5

<table>
<tr><th>Fragment Details</th><th>Structure Details</th></tr>
<tr><td></td><td>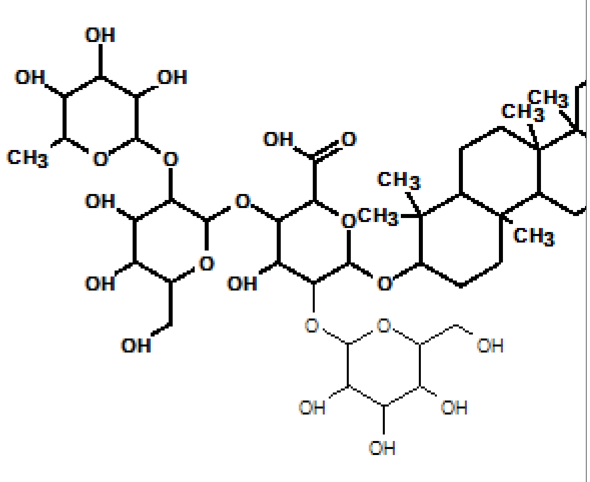
Selected: C48H77O17
Mass: 925.5161
Broken Bonds: 1
Delta H: -2

Contained Neutral Losses
No contained neutral losses</td></tr>
<tr><td>Mass (m/z): 939.4972
Ion Formula: C48H75O18
Error (ppm): 1.4
Intensity (cps): 14.7
RDB: 11.0</td><td>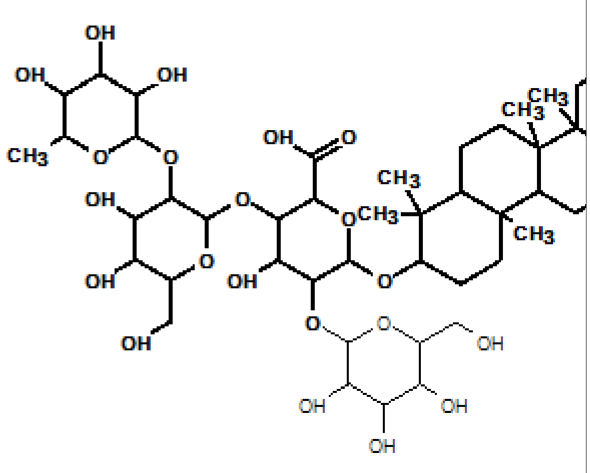
Selected: C48H77O18
Mass: 941.5110
Broken Bonds: 1
Delta H: -2

Contained Neutral Losses
<table><tr><th>Mass</th><th>Formula</th></tr><tr><td>776.4339</td><td>C42H64O13</td></tr><tr><td>484.1405</td><td>C18H28O15</td></tr><tr><td>482.1236</td><td>C18H26O15</td></tr><tr><td>144.0384</td><td>C6H8O4</td></tr></table></td></tr>
</table>

Fragment Details	Structure Details

Mass (m/z): 941.5408
Ion Formula: C49H81O17
Error (ppm): -7.6
Intensity (cps): 15.2
RDB: 9.0

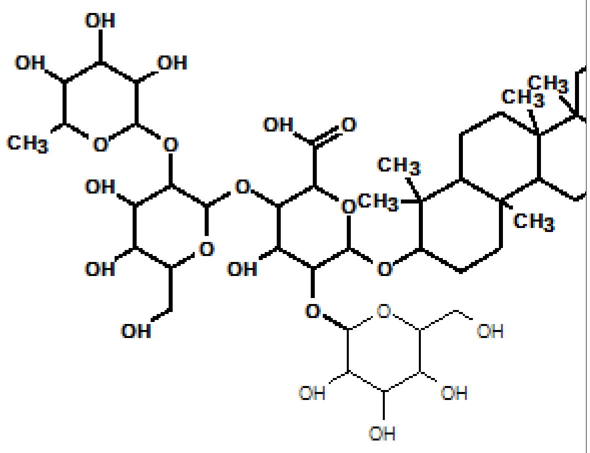

Selected: C49H77O17
Mass: 937.5161
Broken Bonds: 3
Delta H: 2

Contained Neutral Losses

Mass	Formula
778.4775	C43H70O12
164.0676	C6H12O5
18.0291	H2O

Mass (m/z): 957.5135
Ion Formula: C48H77O19
Error (ppm): 7.4
Intensity (cps): 28.6
RDB: 10.0

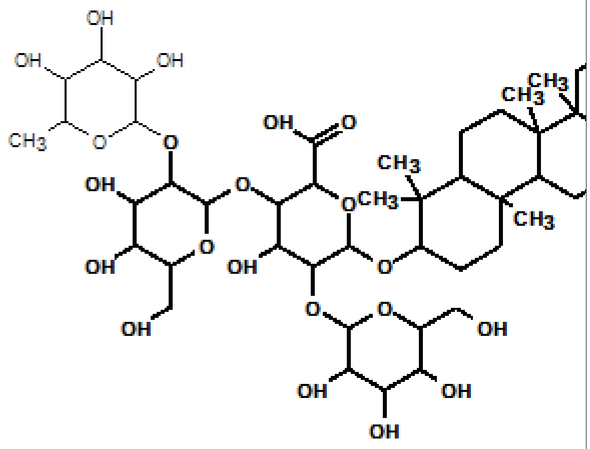

Selected: C48H77O19
Mass: 957.5059
Broken Bonds: 1
Delta H: 0

Contained Neutral Losses

Fragment Details	Structure Details

Mass	Formula
794.4502	C42H66O14
778.4549	C42H66O13
502.1567	C18H30O16
500.1398	C18H28O16
162.0547	C6H10O5
18.0163	H2O

Mass (m/z):	1103.5732
Ion Formula:	C54H87O23
Error (ppm):	8.0
Intensity (cps):	13738.0
RDB:	11.0

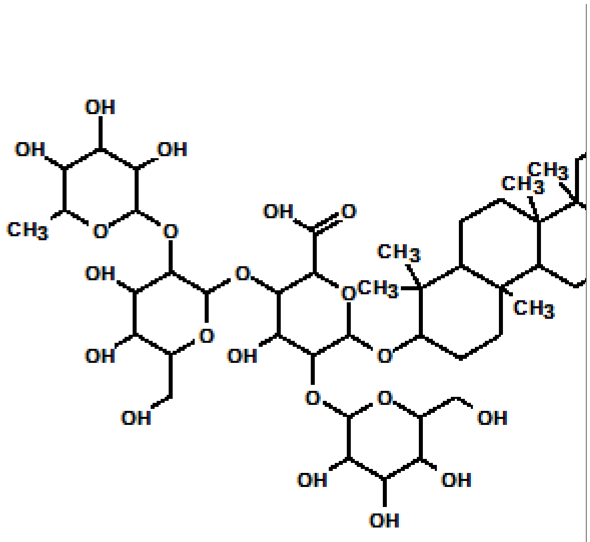

Selected:	C54H88O23
Mass:	1104.5716
Broken Bonds:	0
Delta H:	0

Contained Neutral Losses

Mass	Formula
940.5099	C48H76O18
924.5145	C48H76O17
648.2164	C24H40O20
646.1995	C24H38O20
308.1143	C12H20O9
180.0616	C6H12O6
164.0760	C6H12O5
146.0597	C6H10O4

Interpretation of the metabolite in rat (M20)

M20 – Loss of C12H20O9+Loss of Hydroxymethylene [M-H]-

Formula:	C47H76O17
ppm:	6.3
RDB:	10.0

Available Structure Candidates

Rank	Score	Count
1	100.0	13
2	0.0	8

Applied Metabolite Structure

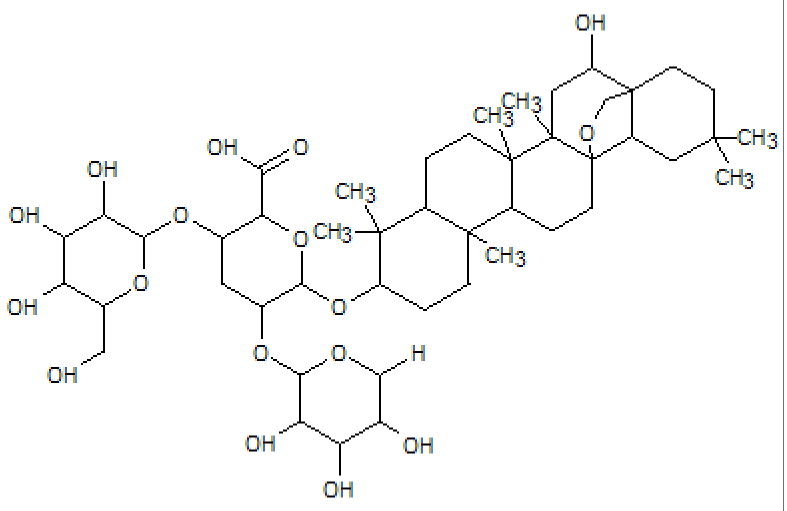

Composition:	C47H76O17
Mass:	912.5083

Metabolite MS/MS

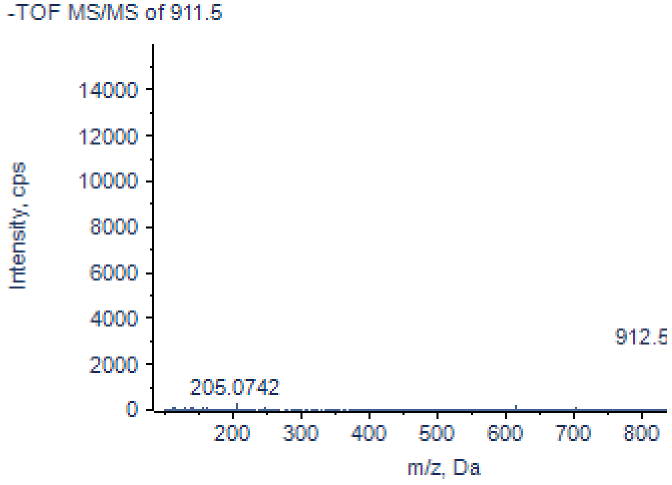

Parent MS/MS

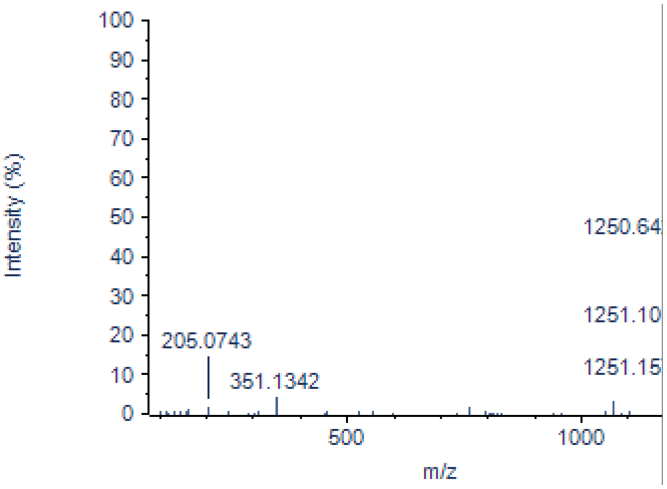

Peaks selected for assignment (m/z): 101.0257, 103.0407, 113.0257, 115.0411, 119.0366, 131.0365, 139.0045, 143.0371, 145.0525, 149.0467, 157.0157, 163.0630, 205.0742, 247.0847, 277.0962, 295.1065, 457.3738, 571.4067, 597.3862, 615.3628, 615.3965, 703.4509, 721.4603, 721.4886, 765.4521, 849.5109, 867.5199, 893.5009, 908.4814, 911.5077

Metabolite Options

Number of fragment peaks selected for assignment:	30
Minimum signal-to-noise ratio:	3
MS/MS m/z tolerance:	15 ppm
Fragmentation Settings	
Break aromatic rings:	True
Maximum number of bonds to break:	4
Maximum number of C-C bonds to break:	4
Label Settings	
Label peaks with:	Ion with ppm Error

Fragment Filters

Fragments with assigned structures	True

Fragment Details	Structure Details
Mass (m/z): 205.0742<br>Ion Formula: C8H13O6<br>Error (ppm): 11.8<br>Intensity (cps): 272.1<br>RDB: 2.0<br>Score: 29.5	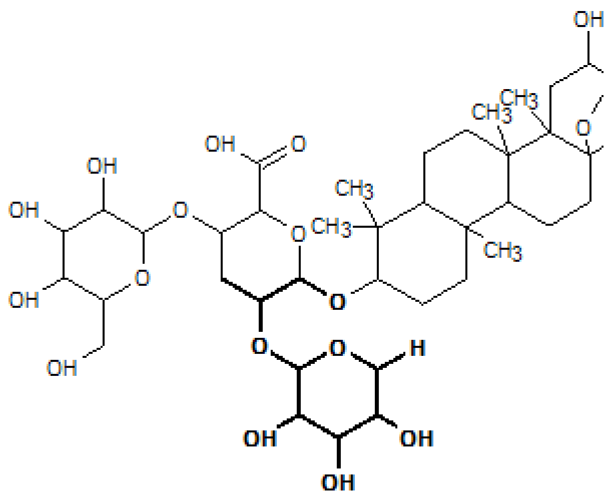<br>Selected: C8H13O6<br>Mass: 205.0712<br>Broken Bonds: 3<br>Delta H: -2<br>Score: 29.5

Contained Neutral Losses

Mass	Formula
74.0377	C3H6O2
56.0274	C3H4O

Fragment Details	Structure Details

Mass (m/z): 247.0847
Ion Formula: C10H15O7
Error (ppm): 9.5
Intensity (cps): 82.0
RDB: 3.0
Score: 29.5

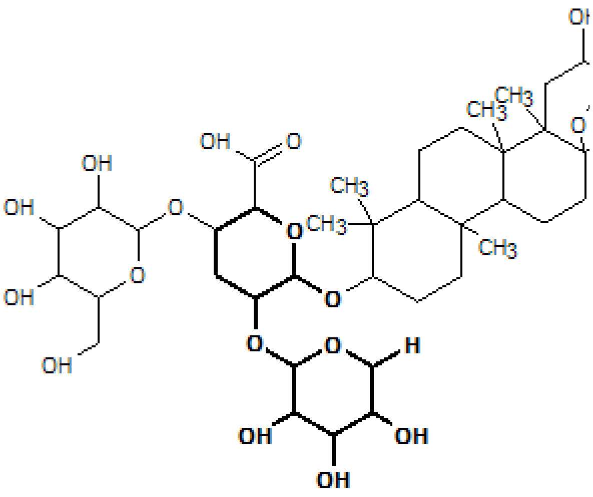

Selected:	C10H15O7
Mass:	247.0818
Broken Bonds:	3
Delta H:	-2
Score:	29.5

Contained Neutral Losses

Mass	Formula
134.0590	C5H10O4
132.0436	C5H8O4
116.0482	C5H8O3
98.0379	C5H6O2

Mass (m/z): 277.0962
Ion Formula: C11H17O8
Error (ppm): 12.1
Intensity (cps): 30.4
RDB: 3.0
Score: 36.5

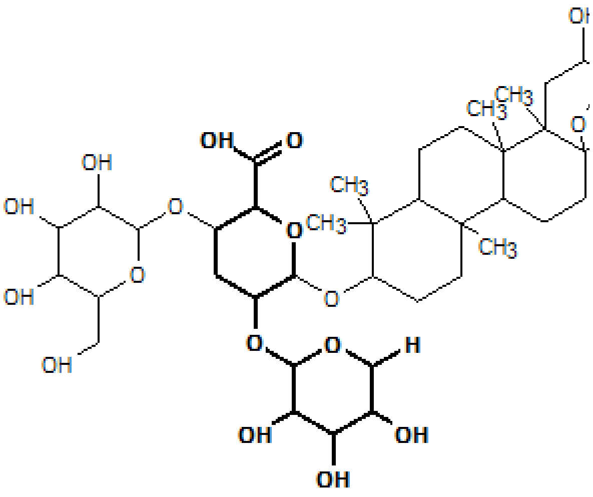

Selected:	C11H16O8
Mass:	276.0845
Broken Bonds:	2
Delta H:	0
Score:	36.5

Contained Neutral Losses

Fragment Details	Structure Details

Mass	Formula
146.0597	C6H10O4
134.0591	C5H10O4
132.0437	C5H8O4
128.0495	C6H8O3

Mass (m/z):	295.1065
Ion Formula:	C11H19O9
Error (ppm):	10.5
Intensity (cps):	16.9
RDB:	2.0
Score:	36.5

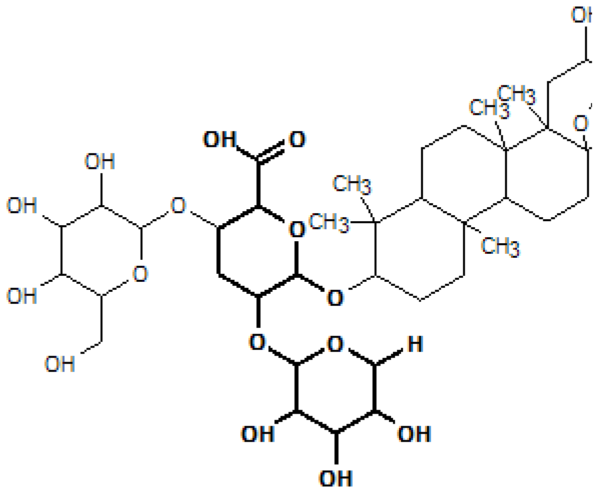

Selected:	C11H16O9
Mass:	292.0794
Broken Bonds:	2
Delta H:	2
Score:	36.5

Contained Neutral Losses

Mass	Formula
164.0700	C6H12O5
152.0694	C5H12O5
150.0540	C5H10O5
146.0598	C6H10O4
132.0436	C5H8O4
48.0219	CH4O2
18.0103	H2O

Mass (m/z):	457.3738
Ion Formula:	C30H49O3
Error (ppm):	11.0
Intensity (cps):	50.9
RDB:	6.0
Score:	40.5

<table>
<tr><th>Fragment Details</th><th>Structure Details</th></tr>
<tr><td></td><td>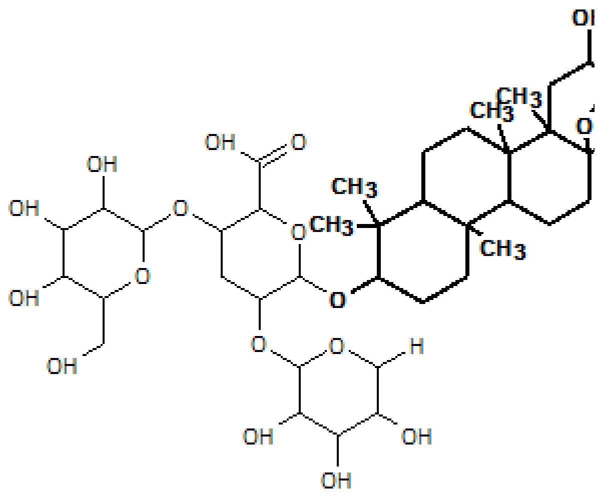
Selected: C30H49O3
Mass: 457.3682
Broken Bonds: 1
Delta H: 0
Score: 40.5
Contained Neutral Losses
No contained neutral losses</td></tr>
<tr><td>Mass (m/z): 571.4067
Ion Formula: C35H55O6
Error (ppm): 11.0
Intensity (cps): 31.0
RDB: 8.0
Score: 29.5</td><td>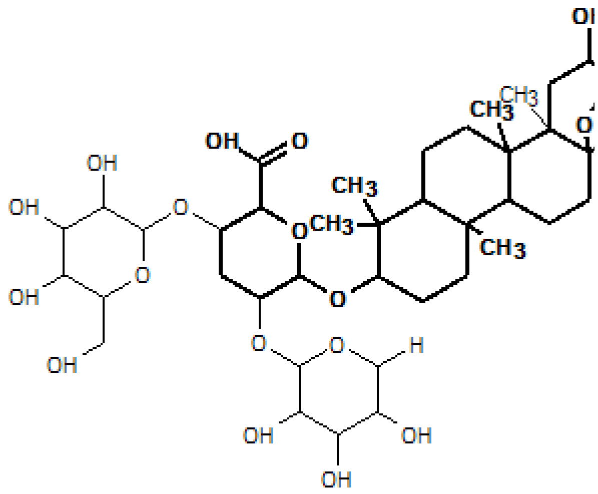
Selected: C35H53O6
Mass: 569.3842
Broken Bonds: 3
Delta H: 0
Score: 29.5
Contained Neutral Losses
<table><tr><th>Mass</th><th>Formula</th></tr><tr><td>428.3696</td><td>C29H48O2</td></tr><tr><td>426.3542</td><td>C29H46O2</td></tr></table></td></tr>
<tr><td>Mass (m/z): 597.3862</td><td></td></tr>
</table>

Fragment Details	Structure Details

Ion Formula:	C36H53O7
Error (ppm):	10.9
Intensity (cps):	42.3
RDB:	10.0
Score:	36.0

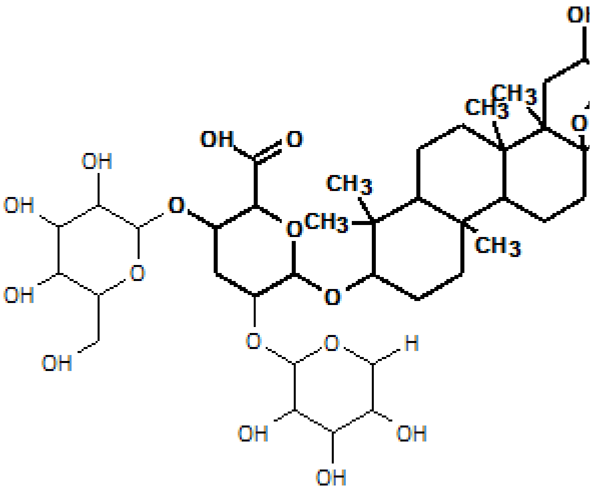

Selected:	C36H56O7
Mass:	600.4026
Broken Bonds:	2
Delta H:	-4
Score:	36.0

Contained Neutral Losses

Mass	Formula
458.3817	C30H50O3
454.3491	C30H46O3
440.3705	C30H48O2
140.0124	C6H4O4

Mass (m/z):	615.3965
Ion Formula:	C36H55O8
Error (ppm):	10.2
Intensity (cps):	193.2
RDB:	9.0
Score:	36.5

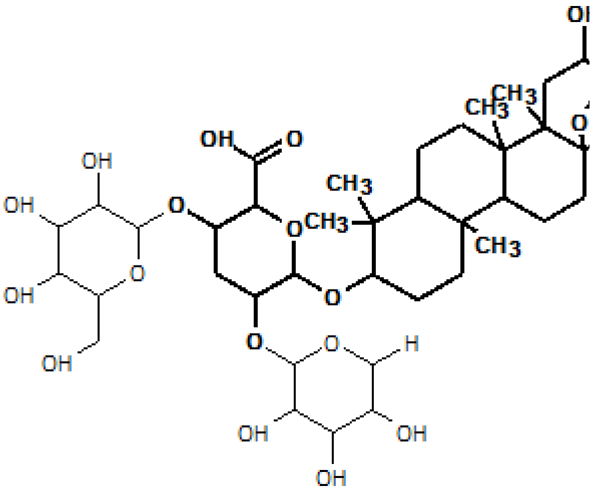

Selected:	C36H56O8
Mass:	616.3975
Broken Bonds:	2
Delta H:	-2
Score:	36.5

Contained Neutral Losses

Mass	Formula

<table>
<tr><th>Fragment Details</th><th>Structure Details</th></tr>
<tr><td></td><td>
<table>
<tr><td>458.3808</td><td>C30H50O3</td></tr>
<tr><td>158.0228</td><td>C6H6O5</td></tr>
<tr><td>43.9898</td><td>CO2</td></tr>
<tr><td>18.0103</td><td>H2O</td></tr>
</table>
</td></tr>
<tr><td>
<table>
<tr><td>Mass (m/z):</td><td>703.4509</td></tr>
<tr><td>Ion Formula:</td><td>C40H63O10</td></tr>
<tr><td>Error (ppm):</td><td>11.6</td></tr>
<tr><td>Intensity (cps):</td><td>58.1</td></tr>
<tr><td>RDB:</td><td>9.0</td></tr>
<tr><td>Score:</td><td>33.5</td></tr>
</table>
</td><td>
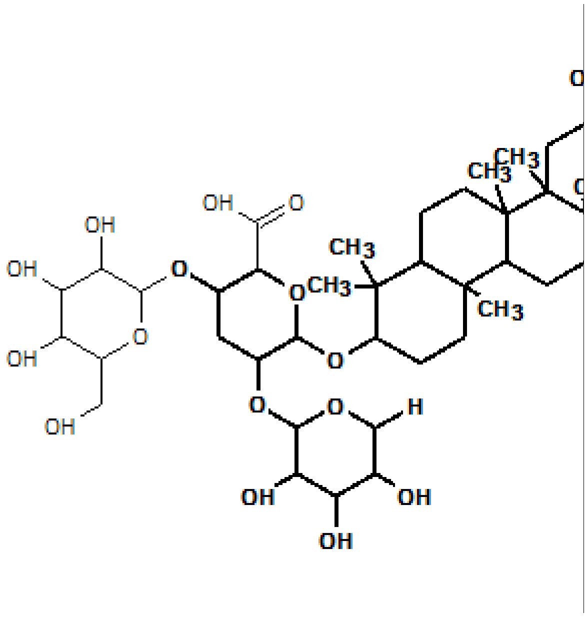

<table>
<tr><td>Selected:</td><td>C40H64O10</td></tr>
<tr><td>Mass:</td><td>704.4499</td></tr>
<tr><td>Broken Bonds:</td><td>2</td></tr>
<tr><td>Delta H:</td><td>-2</td></tr>
<tr><td>Score:</td><td>33.5</td></tr>
</table>
Contained Neutral Losses
<table>
<tr><th>Mass</th><th>Formula</th></tr>
<tr><td>572.4143</td><td>C35H56O6</td></tr>
<tr><td>456.3662</td><td>C30H48O3</td></tr>
<tr><td>246.0771</td><td>C10H14O7</td></tr>
<tr><td>132.0442</td><td>C5H8O4</td></tr>
</table>
</td></tr>
<tr><td>
<table>
<tr><td>Mass (m/z):</td><td>721.4603</td></tr>
<tr><td>Ion Formula:</td><td>C40H65O11</td></tr>
<tr><td>Error (ppm):</td><td>9.8</td></tr>
<tr><td>Intensity (cps):</td><td>20.8</td></tr>
<tr><td>RDB:</td><td>8.0</td></tr>
<tr><td>Score:</td><td>33.5</td></tr>
</table>
</td><td></td></tr>
</table>

Fragment Details	Structure Details

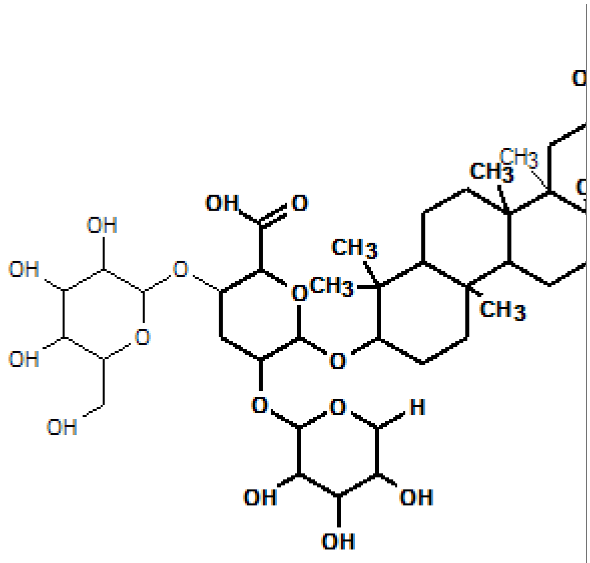

Selected:	C40H62O11
Mass:	718.4292
Broken Bonds:	2
Delta H:	2
Score:	33.5

Contained Neutral Losses

Mass	Formula
590.4238	C35H58O7
572.4136	C35H56O6
444.3641	C29H48O3
426.3538	C29H46O2
150.0536	C5H10O5
18.0095	H2O

Mass (m/z):	721.4886
Ion Formula:	C41H69O10
Error (ppm):	-1.4
Intensity (cps):	15.5
RDB:	7.0
Score:	38.5

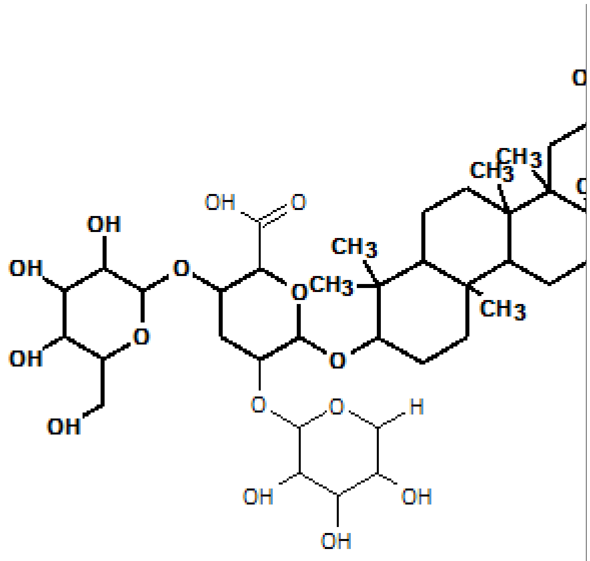

Selected:	C41H66O10
Mass:	718.4656
Broken Bonds:	2
Delta H:	2
Score:	38.5

<table>
<tr><th>Fragment Details</th><th>Structure Details</th></tr>
<tr><td></td><td>

Contained Neutral Losses

<table>
<tr><th>Mass</th><th>Formula</th></tr>
<tr><td>558.4257</td><td>C35H58O5</td></tr>
<tr><td>444.3924</td><td>C30H52O2</td></tr>
<tr><td>264.1149</td><td>C11H20O7</td></tr>
</table>

</td></tr>
<tr><td>

<table>
<tr><td>Mass (m/z):</td><td>765.4521</td></tr>
<tr><td>Ion Formula:</td><td>C41H65O13</td></tr>
<tr><td>Error (ppm):</td><td>11.8</td></tr>
<tr><td>Intensity (cps):</td><td>23.7</td></tr>
<tr><td>RDB:</td><td>9.0</td></tr>
<tr><td>Score:</td><td>33.5</td></tr>
</table>

</td><td>

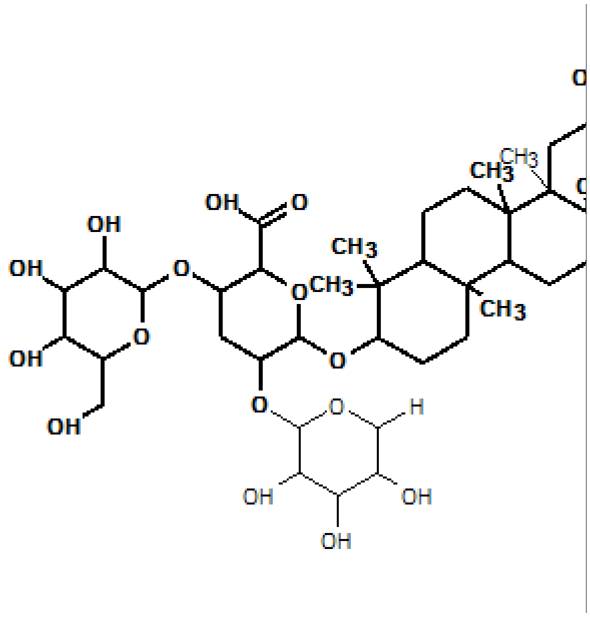

<table>
<tr><td>Selected:</td><td>C41H64O13</td></tr>
<tr><td>Mass:</td><td>764.4347</td></tr>
<tr><td>Broken Bonds:</td><td>2</td></tr>
<tr><td>Delta H:</td><td>0</td></tr>
<tr><td>Score:</td><td>33.5</td></tr>
</table>

Contained Neutral Losses

<table>
<tr><th>Mass</th><th>Formula</th></tr>
<tr><td>602.3891</td><td>C35H54O8</td></tr>
<tr><td>43.9918</td><td>CO2</td></tr>
</table>

</td></tr>
<tr><td>

<table>
<tr><td>Mass (m/z):</td><td>849.5109</td></tr>
<tr><td>Ion Formula:</td><td>C46H73O14</td></tr>
<tr><td>Error (ppm):</td><td>12.1</td></tr>
<tr><td>Intensity (cps):</td><td>103.3</td></tr>
<tr><td>RDB:</td><td>10.0</td></tr>
<tr><td>Score:</td><td>33.5</td></tr>
</table>

</td><td></td></tr>
</table>

<table>
<tr><th>Fragment Details</th><th>Structure Details</th></tr>
<tr><td></td><td>
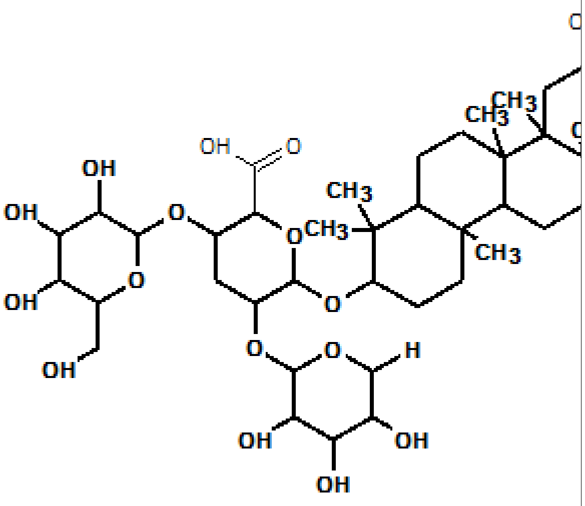

Selected: C46H74O14
Mass: 850.5079
Broken Bonds: 2
Delta H: -2
Score: 33.5

Contained Neutral Losses

<table>
<tr><th>Mass</th><th>Formula</th></tr>
<tr><td>718.4743</td><td>C41H66O10</td></tr>
<tr><td>700.4641</td><td>C41H64O9</td></tr>
<tr><td>692.4952</td><td>C40H68O9</td></tr>
<tr><td>686.4479</td><td>C40H62O9</td></tr>
</table>
</td></tr>
<tr><td>
Mass (m/z): 867.5199
Ion Formula: C46H75O15
Error (ppm): 10.1
Intensity (cps): 24.4
RDB: 9.0
Score: 37.5
</td><td>
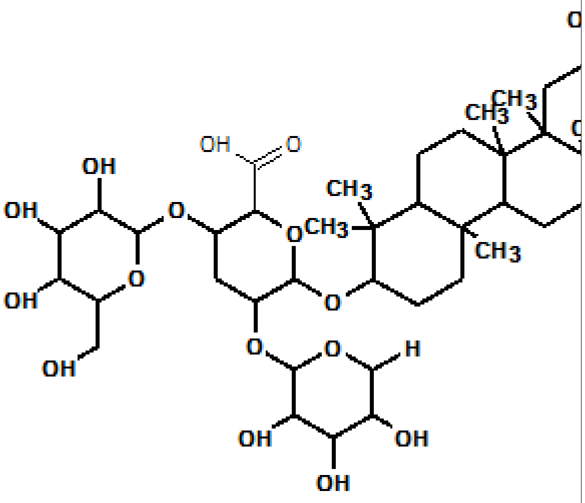

Selected: C46H75O15
Mass: 867.5106
Broken Bonds: 1
Delta H: 0
Score: 37.5

Contained Neutral Losses

<table>
<tr><th>Mass</th><th>Formula</th></tr>
</table>
</td></tr>
</table>

Fragment Details	Structure Details

736.4834	C41H68O11
718.4732	C41H66O10
704.4569	C40H64O10
410.1462	C16H26O12
164.0691	C6H12O5
18.0091	H2O

Mass (m/z):	893.5009
Ion Formula:	C47H73O16
Error (ppm):	11.8
Intensity (cps):	194.5
RDB:	11.0
Score:	40.0

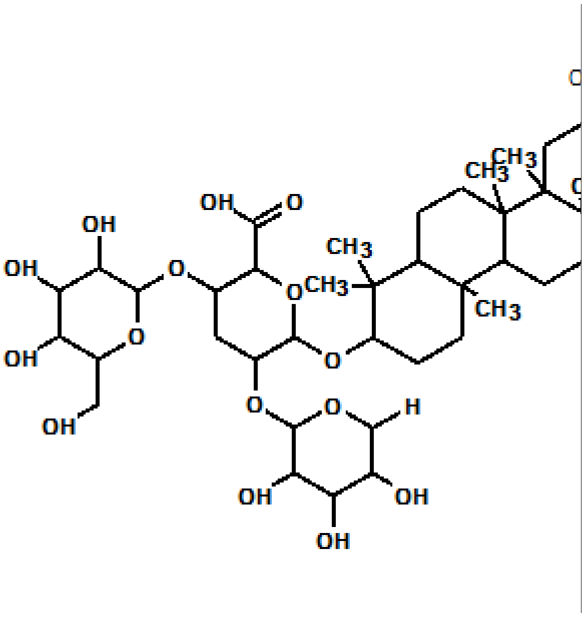

Selected:	C47H75O16
Mass:	895.5055
Broken Bonds:	1
Delta H:	-2
Score:	40.0

Contained Neutral Losses

Mass	Formula
762.4644	C42H66O12
744.4542	C42H64O11
736.4852	C41H68O11
730.4379	C41H62O11
43.9901	CO2

Mass (m/z):	911.5077
Ion Formula:	C47H75O17
Error (ppm):	7.3
Intensity (cps):	15973.0
RDB:	10.0
Score:	44.0

Fragment Details	Structure Details

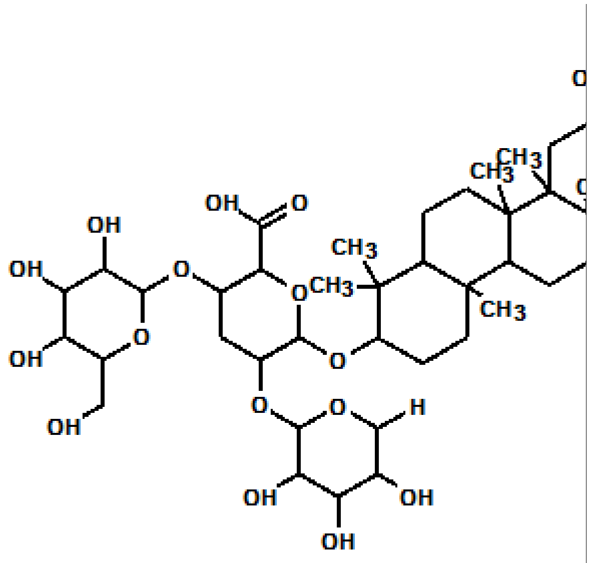

Selected:	C47H76O17
Mass:	912.5083
Broken Bonds:	0
Delta H:	0
Score:	44.0

Contained Neutral Losses

Mass	Formula
780.4711	C42H68O13
762.4609	C42H66O12
748.4447	C41H64O12
454.1339	C17H26O14
43.9877	CO2
18.0067	H2O

Interpretation of the metabolite in rat (M21)

M21 – Loss of C12H20O8 [M-H]-

Formula:	C48H78O19
ppm:	8.8
RDB:	10.0

Available Structure Candidates

Rank	Score	Count
1	100.0	1

Applied Metabolite Structure

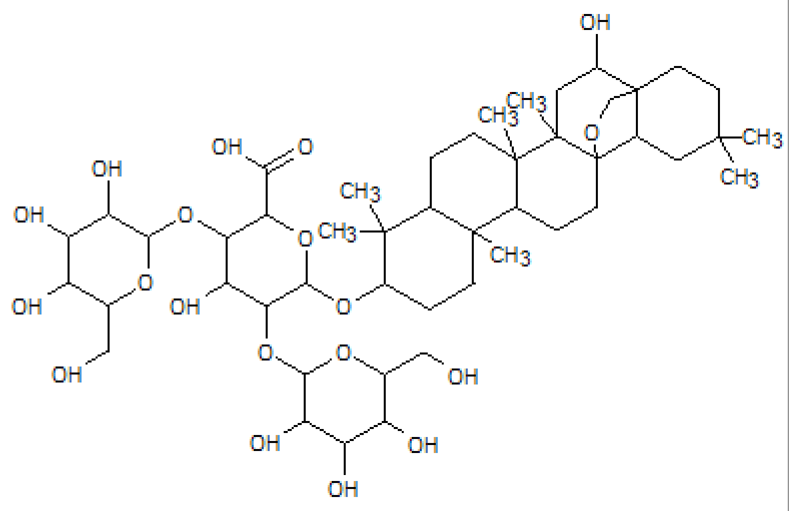

Composition:	C48H78O19
Mass:	958.5137

Metabolite MS/MS

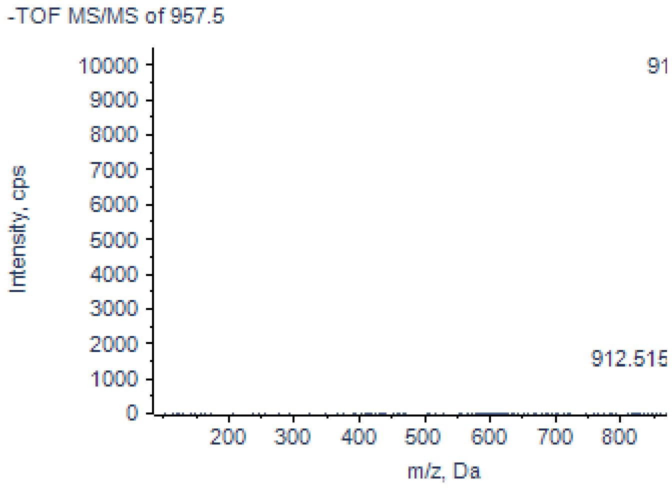

Parent MS/MS

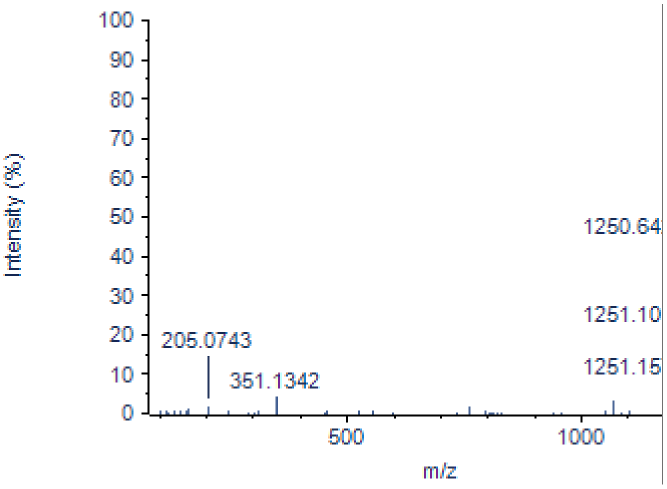

Peaks selected for assignment (m/z): 113.0240, 115.0421, 131.0366, 143.0355, 205.0734, 247.0841, 590.9887, 615.3929, 703.4552, 849.5078, 893.4914, 893.5137, 908.4195, 908.4401, 908.4827, 908.4933, 911.1536, 911.3822, 911.4195, 911.5088, 911.9381, 911.9849, 912.1365, 912.2608, 912.5159, 912.8159, 957.4613, 957.4912, 957.5508, 957.5982

Metabolite Options

Number of fragment peaks selected for assignment:	30
Minimum signal-to-noise ratio:	3
MS/MS m/z tolerance:	15 ppm
Fragmentation Settings	
Break aromatic rings:	True
Maximum number of bonds to break:	4
Maximum number of C-C bonds to break:	4

Label Settings

Label peaks with: Ion with ppm Error

Fragment Filters

Fragments with assigned structures True

<table>
<tr><th>Fragment Details</th><th>Structure Details</th></tr>
<tr><td>Mass (m/z): 205.0734
Ion Formula: C8H13O6
Error (ppm): 7.8
Intensity (cps): 33.0
RDB: 2.0
Score: 29.5</td><td>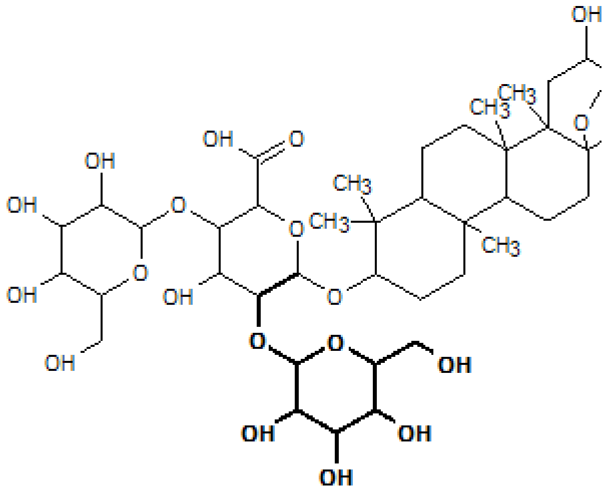
Selected: C8H13O6
Mass: 205.0712
Broken Bonds: 3
Delta H: -2
Score: 29.5

Contained Neutral Losses
No contained neutral losses</td></tr>
<tr><td>Mass (m/z): 893.4914
Ion Formula: C47H73O16
Error (ppm): 1.1
Intensity (cps): 45.9
RDB: 11.0
Score: 38.0</td><td>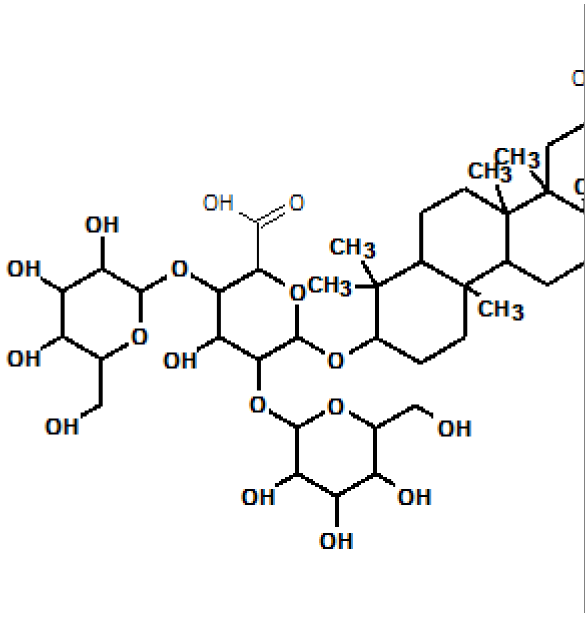
Selected: C47H76O16
Mass: 896.5133
Broken Bonds: 2
Delta H: -4
Score: 38.0</td></tr>
</table>

Fragment Details	Structure Details
	**Contained Neutral Losses**<br>No contained neutral losses
Mass (m/z): 908.4401<br>Ion Formula: C46H68O18<br>Error (ppm): -1.2<br>Intensity (cps): 30.0<br>RDB: 12.5<br>Score: 27.5	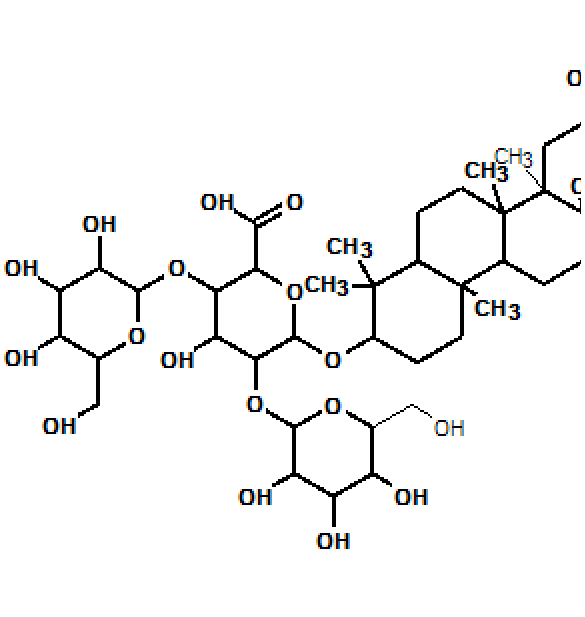<br>Selected: C46H72O18<br>Mass: 912.4719<br>Broken Bonds: 2<br>Delta H: -6<br>Score: 27.5<br>**Contained Neutral Losses**<br>No contained neutral losses
Mass (m/z): 908.4827<br>Ion Formula: C47H72O17<br>Error (ppm): 5.7<br>Intensity (cps): 25.8<br>RDB: 11.5<br>Score: 30.0	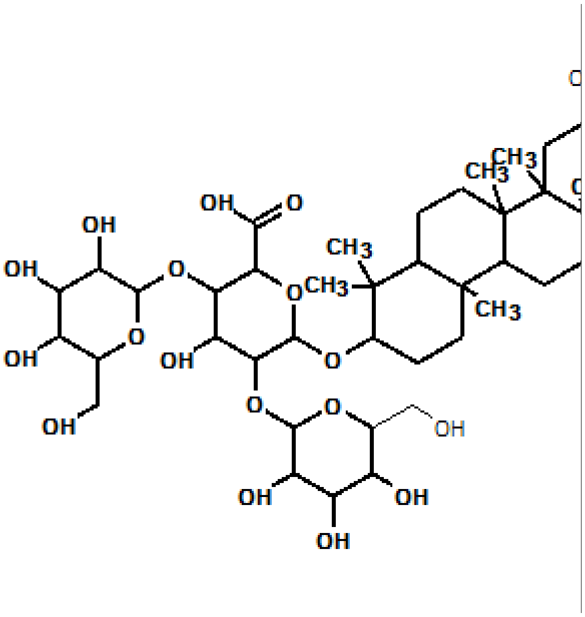<br>Selected: C47H74O17<br>Mass: 910.4926<br>Broken Bonds: 2<br>Delta H: -4<br>Score: 30.0<br>**Contained Neutral Losses**<br>No contained neutral losses

Fragment Details	Structure Details
Mass (m/z): 911.5088<br>Ion Formula: C47H75O17<br>Error (ppm): 8.5<br>Intensity (cps): 10478.4<br>RDB: 10.0<br>Score: 37.0	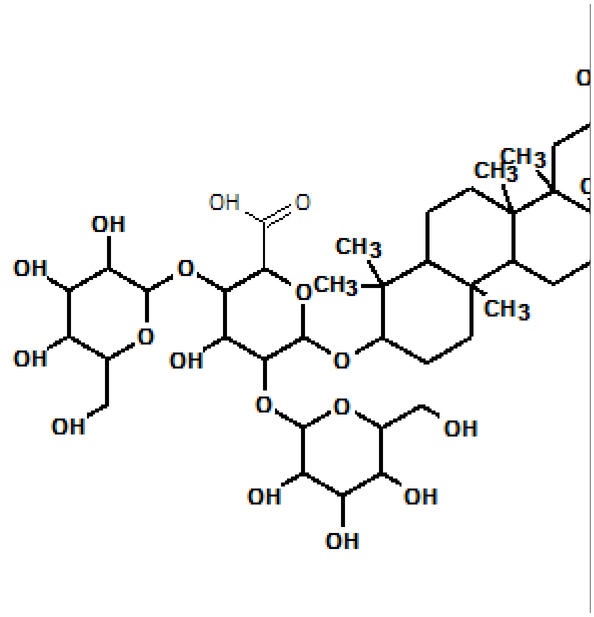<br>Selected: C47H77O17<br>Mass: 913.5161<br>Broken Bonds: 1<br>Delta H: -2<br>Score: 37.0

Contained Neutral Losses

Mass	Formula
18.0173	H2O

Fragment Details	Structure Details
Mass (m/z): 912.5159<br>Ion Formula: C47H76O17<br>Error (ppm): 7.8<br>Intensity (cps): 685.5<br>RDB: 9.5<br>Score: 34.0	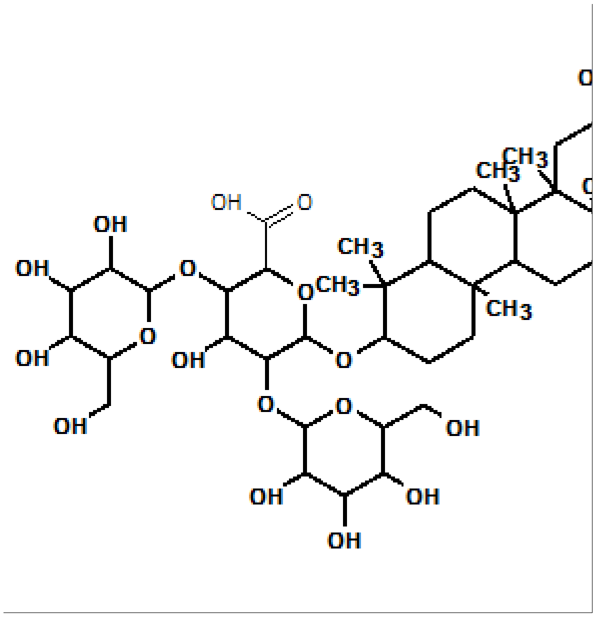<br>Selected: C47H77O17<br>Mass: 913.5161<br>Broken Bonds: 1<br>Delta H: -2<br>Score: 34.0

Contained Neutral Losses

No contained neutral losses

Interpretation of the metabolite in rat (M22)

M22 – Loss of C18H30O13 [M-H]-

Formula:	C42H68O14
ppm:	8.4
RDB:	9.0

Available Structure Candidates

Rank	Score	Count
1	100.0	2

Applied Metabolite Structure

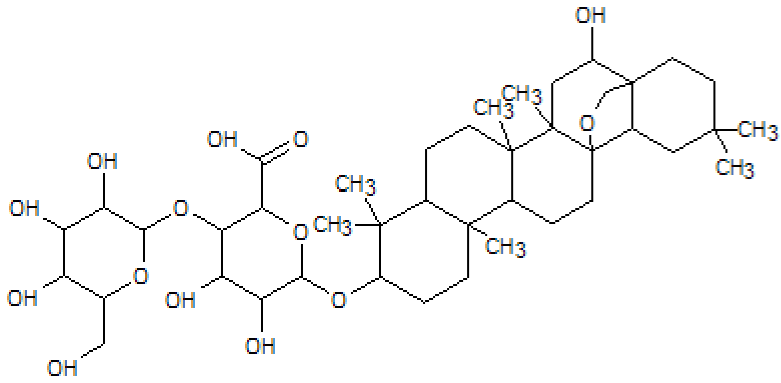

Composition:	C42H68O14
Mass:	796.4609

Metabolite MS/MS

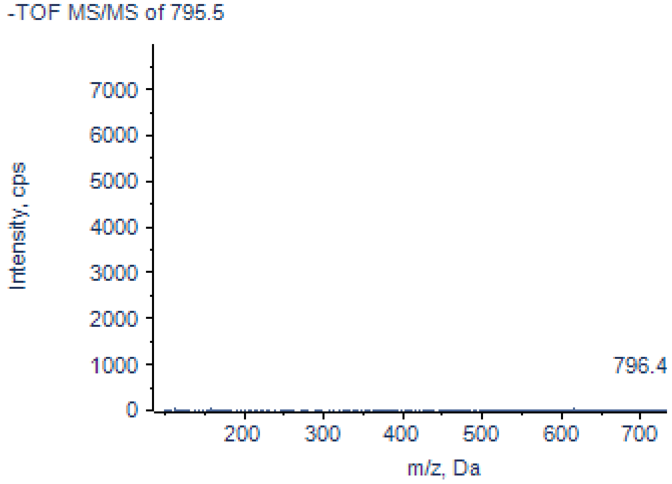

Parent MS/MS

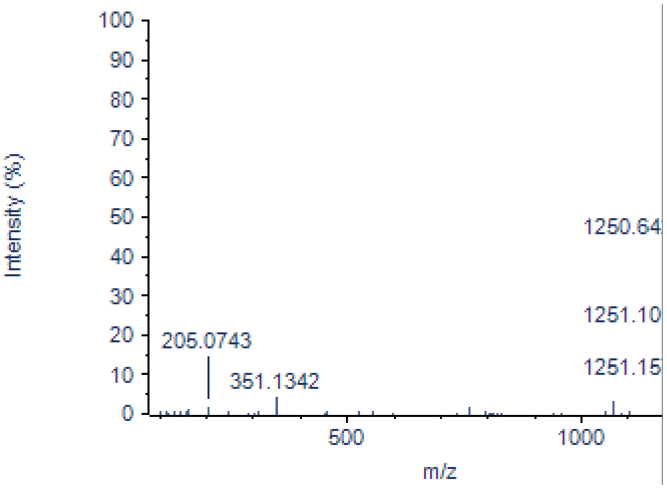

Peaks selected for assignment (m/z): 101.0257, 113.0250, 119.0363, 125.0259, 131.0378, 139.0057, 149.0474, 157.0156, 157.0520, 161.0472, 179.0588, 248.9635, 249.9632, 255.2338, 257.0700, 279.0742, 407.3407, 437.3434, 457.3737, 615.3962, 633.4034, 727.3869, 733.4586, 751.4742, 792.8201, 795.0735, 795.0989, 795.2206, 795.3702, 795.4602

Metabolite Options

Number of fragment peaks selected for assignment:	30
Minimum signal-to-noise ratio:	3
MS/MS m/z tolerance:	10 mDa
Fragmentation Settings	
Break aromatic rings:	True
Maximum number of bonds to break:	4
Maximum number of C-C bonds to break:	4
Label Settings	
Label peaks with:	Ion

Fragment Filters

None

Fragment Details	Structure Details
Mass (m/z): 248.9635<br>Ion Formula: C10HO8<br>Error (ppm): -4.2<br>Intensity (cps): 6.6<br>RDB: 10.0<br>Score: 3.0	
Mass (m/z): 255.2338<br>Ion Formula: C16H31O2<br>Error (ppm): 0.9<br>Intensity (cps): 8.5<br>RDB: 1.0<br>Score: 3.0	
Mass (m/z): 257.0700<br>Ion Formula: C11H13O7<br>Error (ppm): 3.3<br>Intensity (cps): 11.3<br>RDB: 5.0<br>Score: 24.5	

Fragment Details	Structure Details

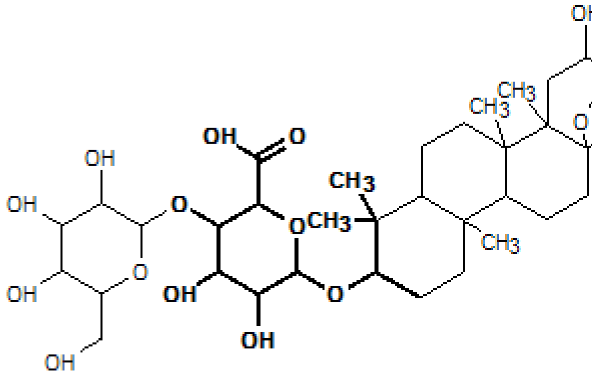

Selected:	C11H17O7
Mass:	261.0974
Broken Bonds:	3
Delta H:	-6
Score:	24.5

Contained Neutral Losses

No contained neutral losses

Mass (m/z):	279.0742
Ion Formula:	C10H15O9
Error (ppm):	2.1
Intensity (cps):	5.4
RDB:	3.0
Score:	33.5

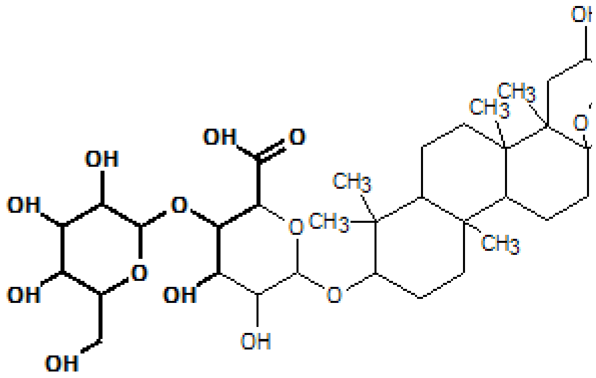

Selected:	C10H16O9
Mass:	280.0794
Broken Bonds:	2
Delta H:	-2
Score:	33.5

Contained Neutral Losses

Mass	Formula
178.0486	C6H10O6
160.0379	C6H8O5
122.0586	C4H10O4
118.0270	C4H6O4

Fragment Details	Structure Details
	100.0154 | C4H4O3
Mass (m/z): 407.3407<br>Ion Formula: C29H43O<br>Error (ppm): 8.7<br>Intensity (cps): 5.2<br>RDB: 8.0<br>Score: 29.5	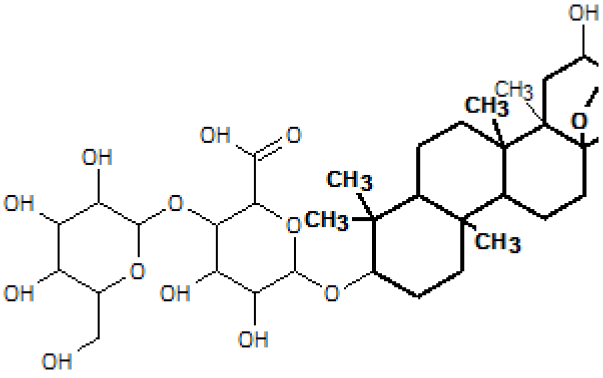<br>Selected: C29H45O<br>Mass: 409.3470<br>Broken Bonds: 3<br>Delta H: -4<br>Score: 29.5<br>**Contained Neutral Losses**<br>No contained neutral losses
Mass (m/z): 437.3434<br>Ion Formula: C30H45O2<br>Error (ppm): 0.9<br>Intensity (cps): 5.6<br>RDB: 8.0<br>Score: 42.0	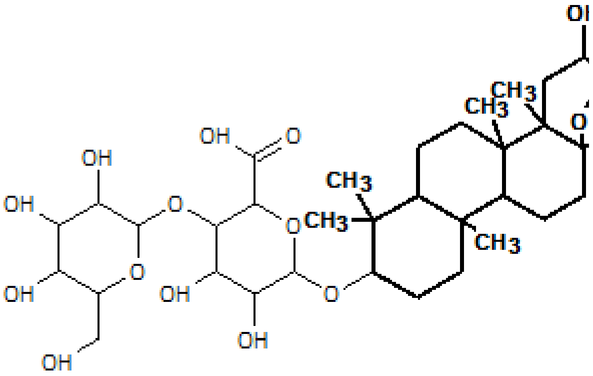<br>Selected: C30H49O2<br>Mass: 441.3733<br>Broken Bonds: 1<br>Delta H: -4<br>Score: 42.0<br>**Contained Neutral Losses**<br>No contained neutral losses
Mass (m/z): 457.3737	

Fragment Details	Structure Details
Ion Formula: C30H49O3<br>Error (ppm): 5.0<br>Intensity (cps): 12.6<br>RDB: 6.0<br>Score: 40.5	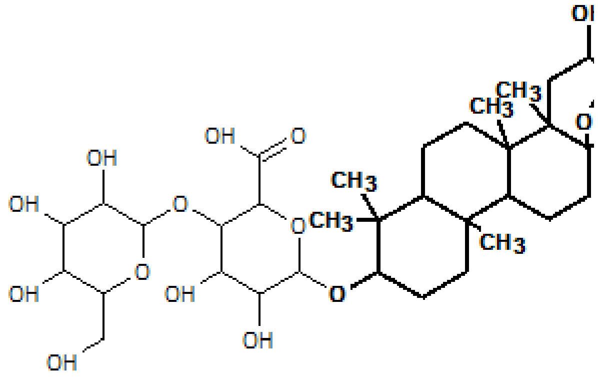<br>Selected: C30H49O3<br>Mass: 457.3682<br>Broken Bonds: 1<br>Delta H: 0<br>Score: 40.5<br>**Contained Neutral Losses**<br>No contained neutral losses
Mass (m/z): 615.3962<br>Ion Formula: C36H55O8<br>Error (ppm): 5.9<br>Intensity (cps): 37.1<br>RDB: 9.0<br>Score: 40.0	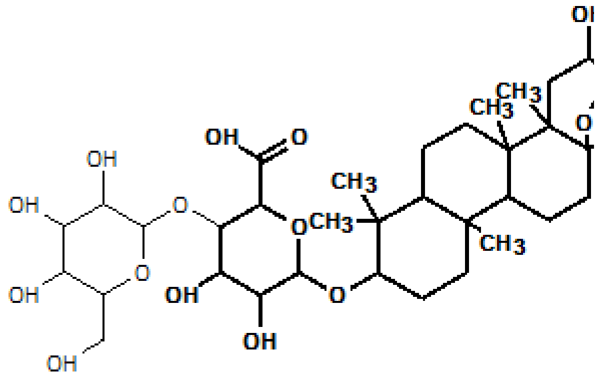<br>Selected: C36H57O8<br>Mass: 617.4053<br>Broken Bonds: 1<br>Delta H: -2<br>Score: 40.0<br>**Contained Neutral Losses**

Mass	Formula
458.3806	C30H50O3
454.3489	C30H46O3
178.0527	C6H10O6
158.0224	C6H6O5

Fragment Details	Structure Details
Mass (m/z): 633.4034<br>Ion Formula: C36H57O9<br>Error (ppm): 2.6<br>Intensity (cps): 8.1<br>RDB: 8.0<br>Score: 40.5	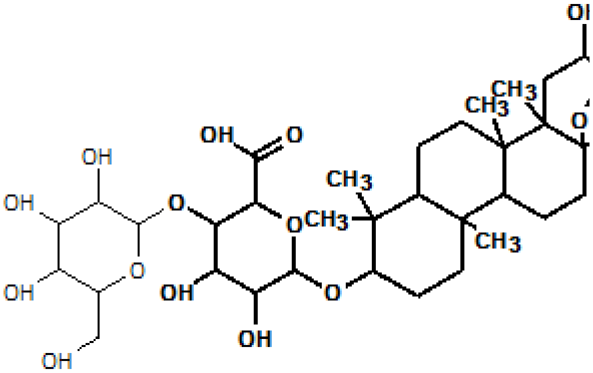 Selected: C36H57O9<br>Mass: 633.4003<br>Broken Bonds: 1<br>Delta H: 0<br>Score: 40.5

Contained Neutral Losses

Mass	Formula
196.0600	C6H12O7
176.0297	C6H8O6
18.0073	H2O

Fragment Details	Structure Details
Mass (m/z): 727.3869<br>Ion Formula: C37H59O14<br>Error (ppm): -4.1<br>Intensity (cps): 8.6<br>RDB: 8.0<br>Score: 29.0	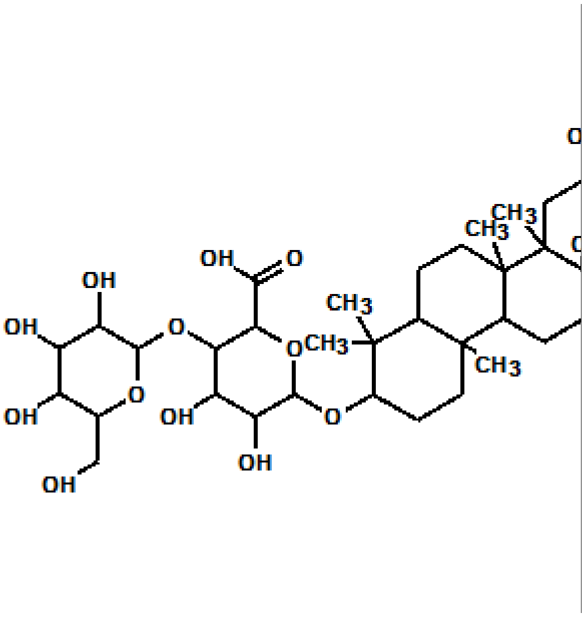 Selected: C37H58O14<br>Mass: 726.3827<br>Broken Bonds: 2<br>Delta H: 0<br>Score: 29.0

Contained Neutral Losses

Mass	Formula

Fragment Details	Structure Details

566.3397	$C_{31}H_{50}O_9$
548.3281	$C_{31}H_{48}O_8$

Mass (m/z):	733.4586
Ion Formula:	$C_{41}H_{65}O_{11}$
Error (ppm):	5.4
Intensity (cps):	18.8
RDB:	9.0
Score:	33.5

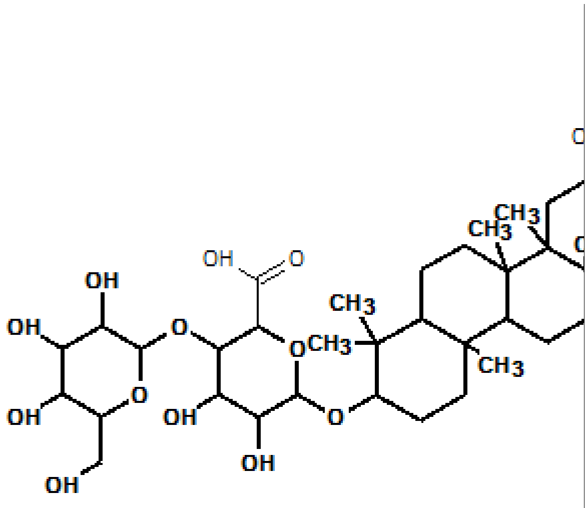

Selected:	$C_{41}H_{66}O_{11}$
Mass:	734.4605
Broken Bonds:	2
Delta H:	-2
Score:	33.5

Contained Neutral Losses

Mass	Formula
576.4430	$C_{35}H_{60}O_6$
572.4114	$C_{35}H_{56}O_6$
554.3998	$C_{35}H_{54}O_5$
296.1152	$C_{11}H_{20}O_9$

Mass (m/z):	795.4602
Ion Formula:	$C_{42}H_{67}O_{14}$
Error (ppm):	6.6
Intensity (cps):	7968.8
RDB:	9.0
Score:	44.0

Fragment Details	Structure Details
	(see below)

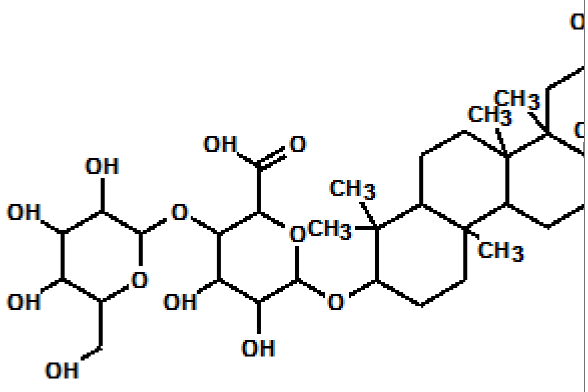

Selected:	C42H68O14
Mass:	796.4609
Broken Bonds:	0
Delta H:	0
Score:	44.0

Contained Neutral Losses

Mass	Formula
634.4130	C36H58O9
358.1168	C12H22O12
338.0865	C12H18O11
180.0641	C6H12O6
162.0568	C6H10O5

Interpretation of the metabolite in rat (M23)

M23 – Loss of Hydroxymethylene [M-H]-

Formula:	C59H96O26
ppm:	5.5
RDB:	12.0

Available Structure Candidates

Rank	Score	Count
1	NaN	2

Applied Metabolite Structure

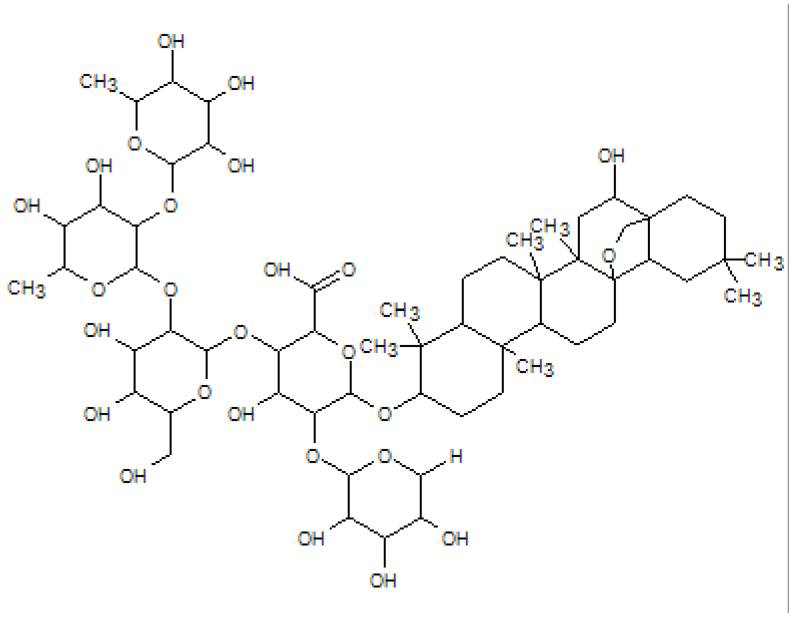

Composition:	C59H96O26
Mass:	1220.6190

Metabolite MS/MS

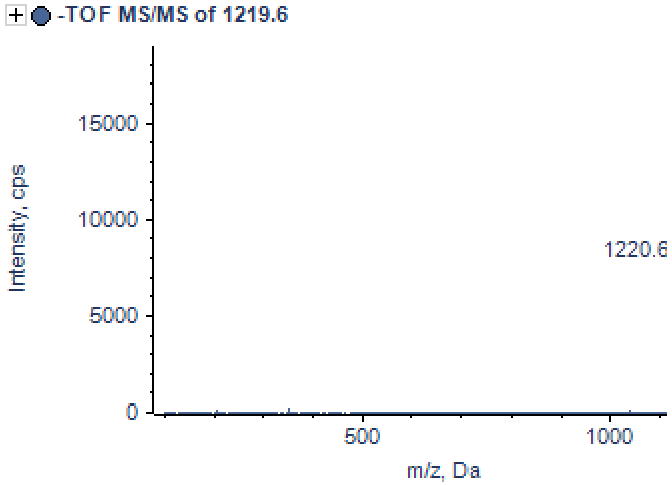

Parent MS/MS

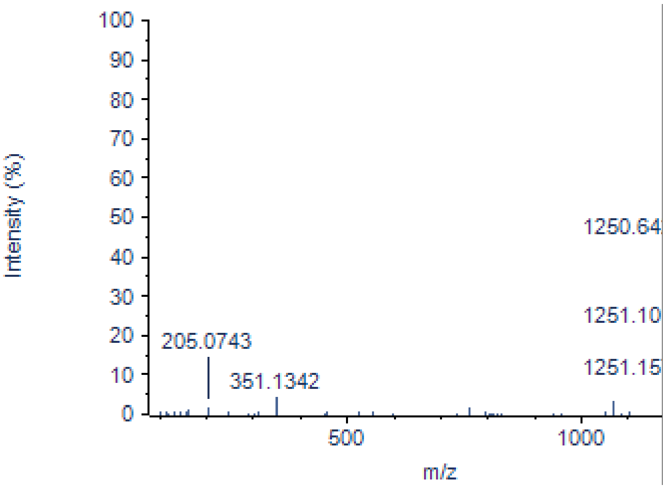

Peaks selected for assignment (m/z): 113.0261, 115.0420, 131.0370, 143.0368, 157.0150, 161.0468, 163.0624, 205.0744, 291.1127, 301.0634, 309.1206, 351.1328, 423.1534, 455.3583, 457.3729, 525.4056, 553.3957, 759.4414, 805.5719, 1011.5580, 1039.5614, 1201.6138, 1215.5637, 1217.7247, 1217.7893, 1218.0594, 1218.1263, 1218.1526, 1218.8378, 1219.6215

Metabolite Options

Number of fragment peaks selected for assignment:	30
Minimum signal-to-noise ratio:	3
MS/MS m/z tolerance:	15 ppm
Fragmentation Settings	
Break aromatic rings:	True

Maximum number of bonds to break:	4
Maximum number of C-C bonds to break:	4

Label Settings

Label peaks with:	Ion with ppm Error

Fragment Filters

Mass Range (m/z)	200 To 1200
Fragments with assigned structures	True

Fragment Details / Structure Details

Fragment Details

Mass (m/z):	291.1127
Ion Formula:	C12H19O8
Error (ppm):	14.4
Intensity (cps):	26.2
RDB:	3.0

Structure Details

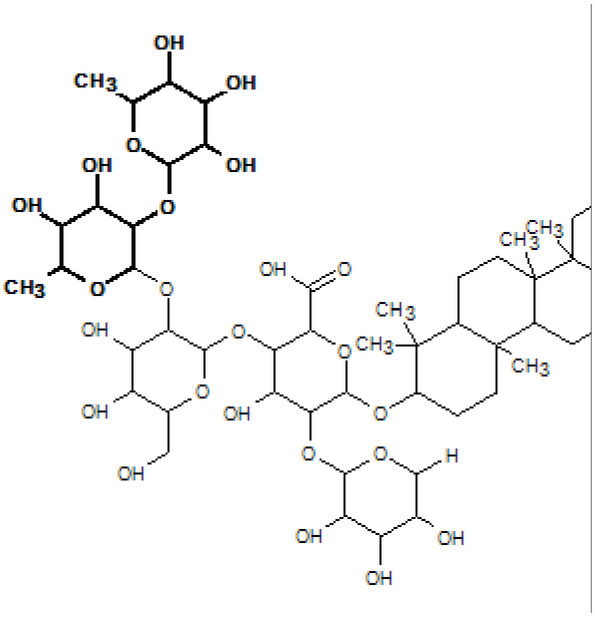

Selected:	C12H21O8
Mass:	293.1236
Broken Bonds:	1
Delta H:	-2

Contained Neutral Losses

Mass	Formula
148.0759	C6H12O4
134.0977	C6H14O3
130.0659	C6H10O3
128.0503	C6H8O3

Fragment Details

Mass (m/z):	309.1206
Ion Formula:	C12H21O9
Error (ppm):	4.9
Intensity (cps):	48.2
RDB:	2.0

Fragment Details	Structure Details

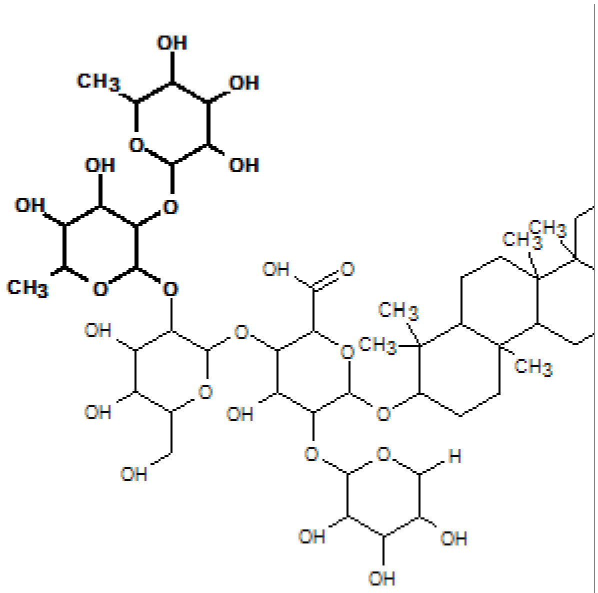

Selected:	C12H21O9
Mass:	309.1186
Broken Bonds:	1
Delta H:	0

Contained Neutral Losses

Mass	Formula
166.0838	C6H14O5
148.0738	C6H12O4
146.0582	C6H10O4
18.0079	H2O

Mass (m/z):	351.1328
Ion Formula:	C14H23O10
Error (ppm):	8.8
Intensity (cps):	203.3
RDB:	3.0

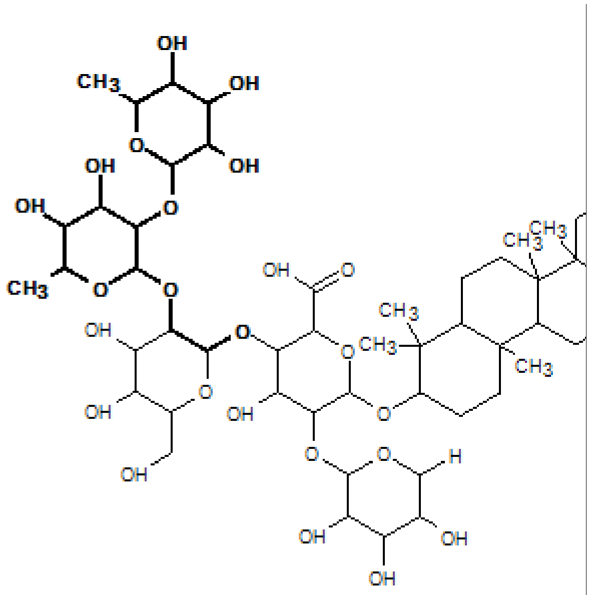

Selected:	C14H23O10
Mass:	351.1291
Broken Bonds:	3
Delta H:	-2

Contained Neutral Losses

Mass	Formula

Fragment Details	Structure Details

208.0959	C8H16O6
194.1177	C8H18O5
190.0860	C8H14O5
188.0704	C8H12O5
146.0584	C6H10O4
60.0200	C2H4O2
42.0122	C2H2O

Mass (m/z):	423.1534
Ion Formula:	C17H27O12
Error (ppm):	6.0
Intensity (cps):	18.8
RDB:	4.0

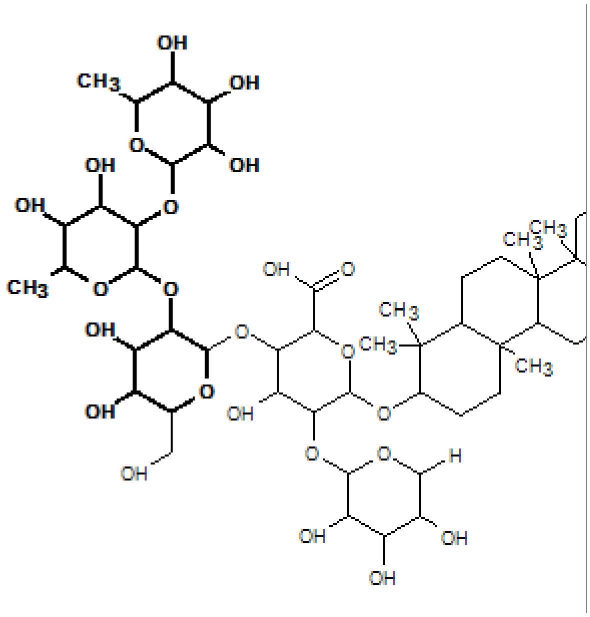

Selected:	C17H28O12
Mass:	424.1581
Broken Bonds:	2
Delta H:	-2

Contained Neutral Losses

Mass	Formula
310.1273	C12H22O9
280.1165	C11H20O8
266.1383	C11H22O7
262.1065	C11H18O7
260.0909	C11H16O7
132.0406	C5H8O4
114.0327	C5H6O3

Mass (m/z):	455.3583
Ion Formula:	C30H47O3
Error (ppm):	11.5

<table>
<tr><th>Fragment Details</th><th>Structure Details</th></tr>
<tr><td>Intensity (cps): 42.0
RDB: 7.0</td><td>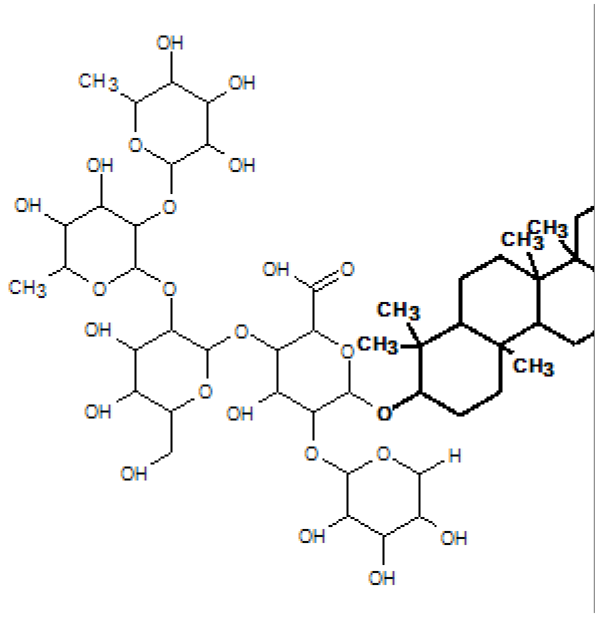

Selected: C30H49O3
Mass: 457.3682
Broken Bonds: 1
Delta H: -2
Contained Neutral Losses
No contained neutral losses</td></tr>
<tr><td>Mass (m/z): 457.3729
Ion Formula: C30H49O3
Error (ppm): 9.1
Intensity (cps): 57.4
RDB: 6.0</td><td>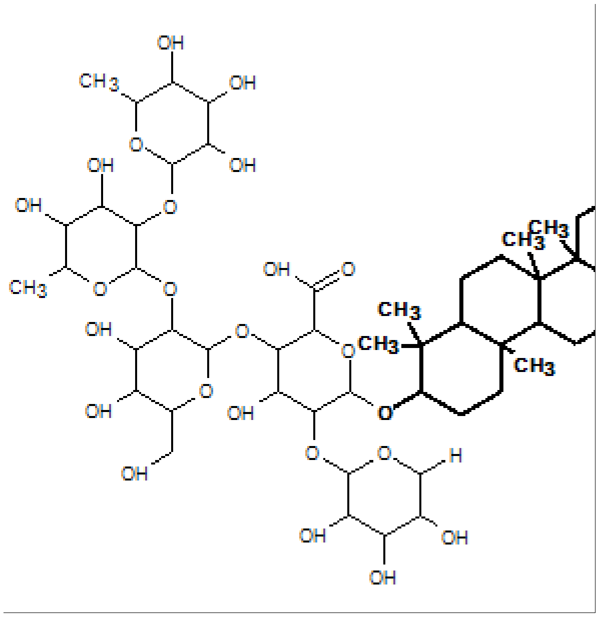

Selected: C30H49O3
Mass: 457.3682
Broken Bonds: 1
Delta H: 0
Contained Neutral Losses
No contained neutral losses</td></tr>
<tr><td>Mass (m/z): 553.3957
Ion Formula: C35H53O5
Error (ppm): 10.6</td><td></td></tr>
</table>

Fragment Details	Structure Details
Intensity (cps): 33.5<br>RDB: 9.0	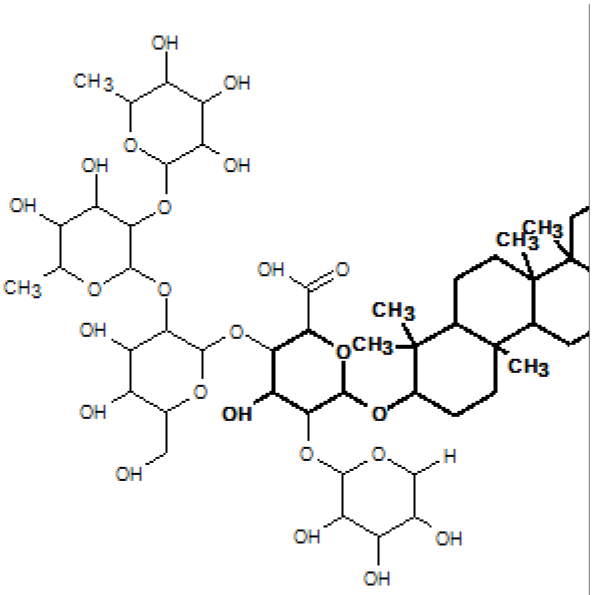<br>Selected: C35H55O5<br>Mass: 555.4050<br>Broken Bonds: 3<br>Delta H: -4

Contained Neutral Losses

Mass	Formula
440.3697	C30H48O2
98.0374	C5H6O2
96.0228	C5H4O2

Fragment Details	Structure Details
Mass (m/z): 759.4414<br>Ion Formula: C42H63O12<br>Error (ppm): 11.8<br>Intensity (cps): 59.1<br>RDB: 11.0	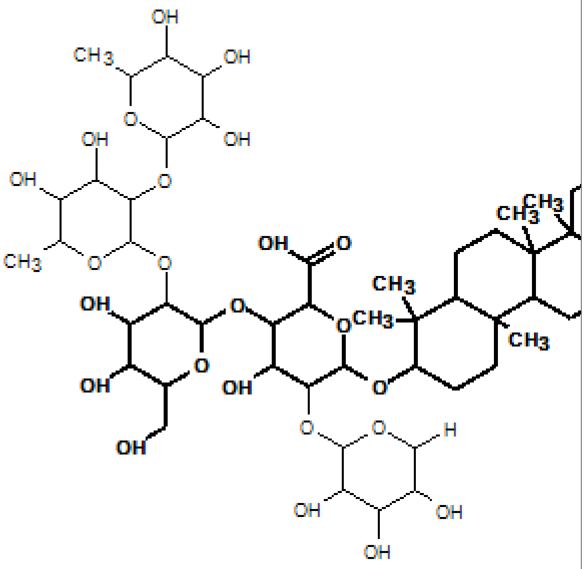<br>Selected: C42H66O12<br>Mass: 762.4554<br>Broken Bonds: 2<br>Delta H: -4

Contained Neutral Losses

Mass	Formula
602.4264	C36H58O7

Fragment Details	Structure Details

304.0831	C12H16O9
302.0685	C12H14O9

Mass (m/z):	1011.5580
Ion Formula:	C52H83O19
Error (ppm):	4.6
Intensity (cps):	19.0
RDB:	11.0

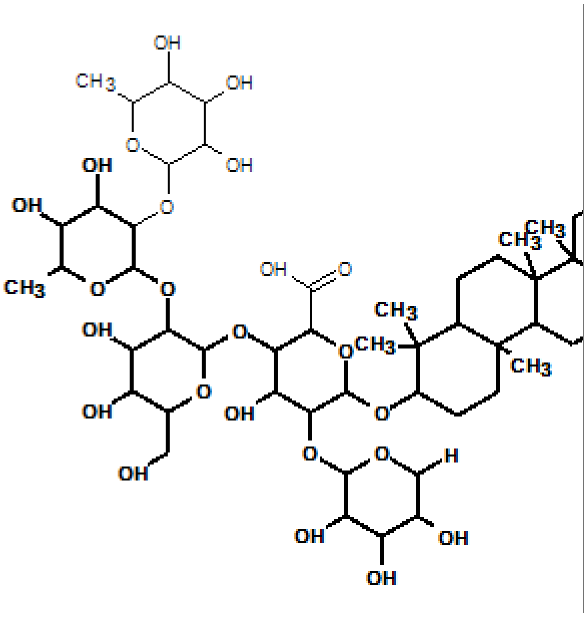

Selected:	C52H84O19
Mass:	1012.5607
Broken Bonds:	2
Delta H:	-2

Contained Neutral Losses

Mass	Formula
868.5212	C46H76O15
720.4453	C40H64O11
556.1997	C22H36O16
554.1851	C22H34O16

Mass (m/z):	1039.5614
Ion Formula:	C53H83O20
Error (ppm):	12.5
Intensity (cps):	151.8
RDB:	12.0

<table>
<tr><th>Fragment Details</th><th>Structure Details</th></tr>
<tr><td></td><td>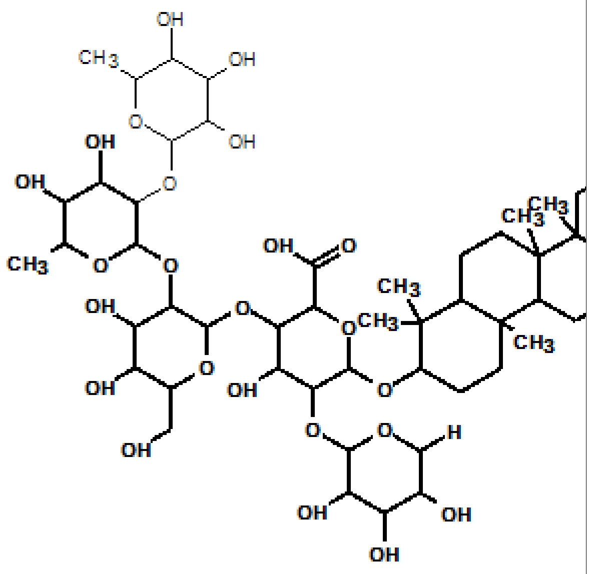

<table>
<tr><td>Selected:</td><td>C53H84O20</td></tr>
<tr><td>Mass:</td><td>1040.5556</td></tr>
<tr><td>Broken Bonds:</td><td>2</td></tr>
<tr><td>Delta H:</td><td>-2</td></tr>
</table>

Contained Neutral Losses

<table>
<tr><th>Mass</th><th>Formula</th></tr>
<tr><td>896.5245</td><td>C47H76O16</td></tr>
<tr><td>730.4407</td><td>C41H62O11</td></tr>
<tr><td>28.0033</td><td>CO</td></tr>
</table>
</td></tr>
</table>

Interpretation of the metabolite in rat (M24)

M24 – Loss of O and C12H20O9 [M-H]-

Formula:	C48H78O17
ppm:	7.8
RDB:	10.0

Available Structure Candidates

Rank	Score	Count
1	100.0	4
2	0.0	8

Applied Metabolite Structure

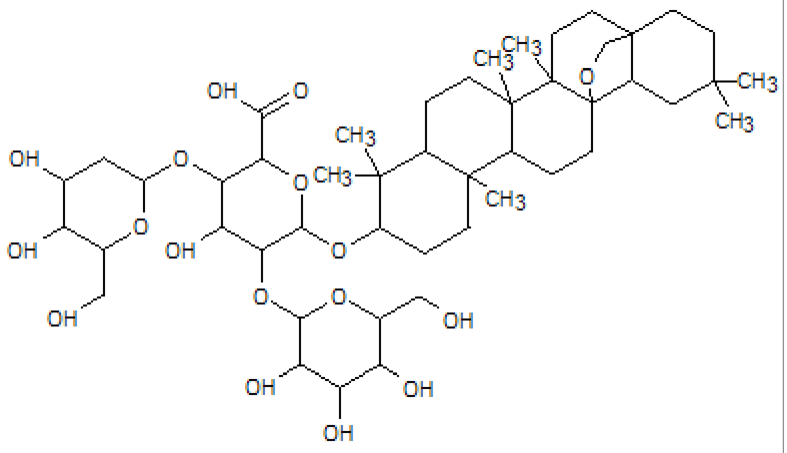

Composition:	C48H78O17
Mass:	926.5239

Metabolite MS/MS

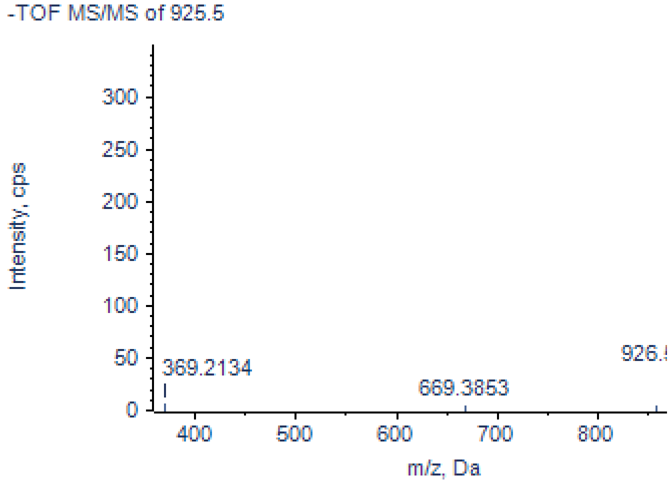

Parent MS/MS

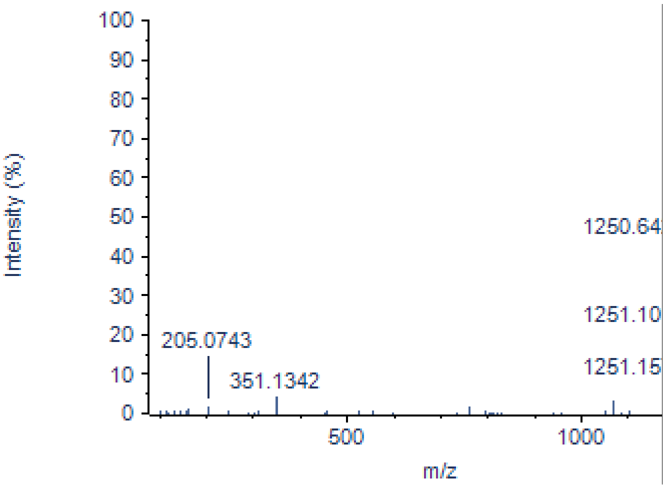

Peaks selected for assignment (m/z): 369.2134, 669.3263, 669.3765, 669.3853, 857.6846, 907.5060, 907.5301, 907.6115, 922.5002, 925.4361, 925.5270, 925.6039

Metabolite Options

Number of fragment peaks selected for assignment:	30
Minimum signal-to-noise ratio:	3
MS/MS m/z tolerance:	15 ppm
Fragmentation Settings	
Break aromatic rings:	True
Maximum number of bonds to break:	4
Maximum number of C-C bonds to break:	4
Label Settings	

Label peaks with: Ion with ppm Error

Fragment Filters

None

<table>
<tr><th>Fragment Details</th><th>Structure Details</th></tr>
<tr><td>Mass (m/z): 925.5270
Ion Formula: C48H77O17
Error (ppm): 11.2
Intensity (cps): 349.0
RDB: 10.0
Score: 44.0</td><td>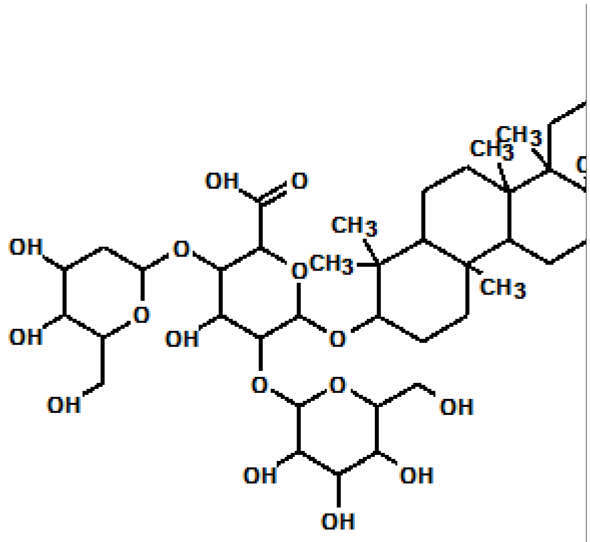
Se-lected: C48H78O17
Mass: 926.5239
Bro-ken Bonds: 0
Delta H: 0
Score: 44.0

Contained Neutral Losses
No contained neutral losses</td></tr>
</table>

Interpretation of the metabolite in rat (M25)

M25 – Loss of C6H10O6+Sulfate Conjugation [M-H]-

Formula:	C54H88O24S
ppm:	4.0
RDB:	11.0

Available Structure Candidates

Rank	Score	Count
1	100.0	11
2	0.0	253

Applied Metabolite Structure

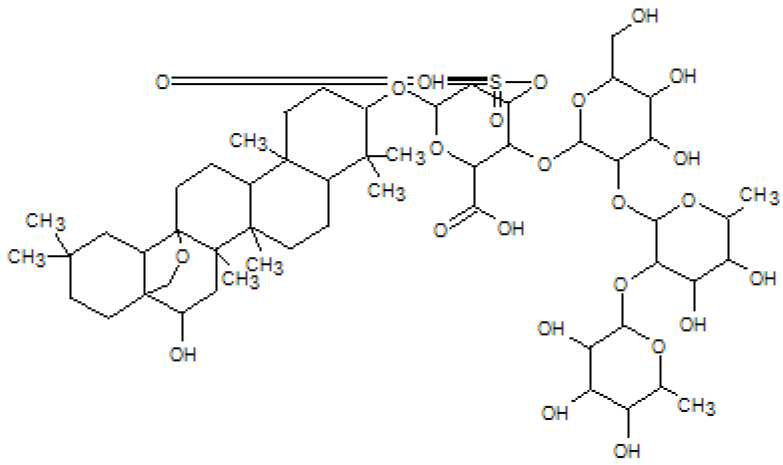

Composition:	C54H88O24S
Mass:	1152.5386

Metabolite MS/MS

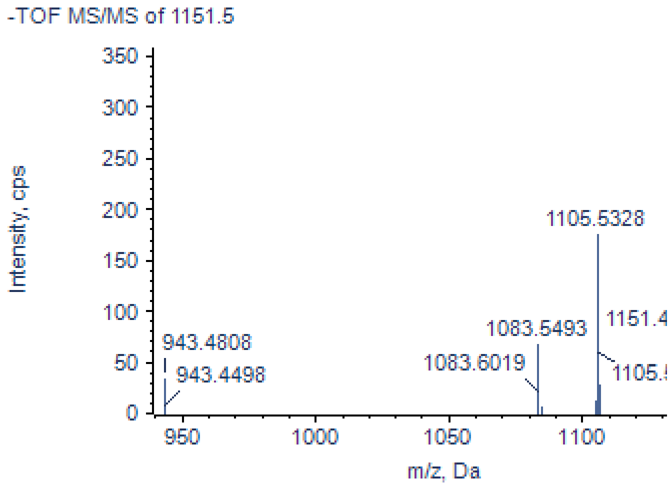

Parent MS/MS

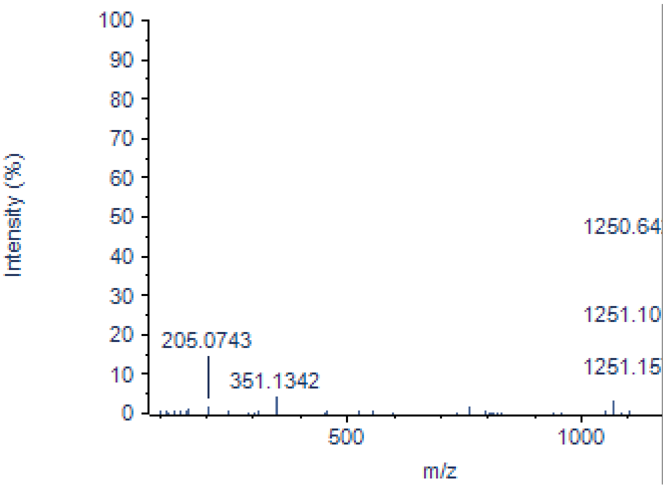

Peaks selected for assignment (m/z): 943.4498, 943.4808, 1083.4801, 1083.5057, 1083.5493, 1083.5770, 1083.6019, 1105.4432, 1105.4749, 1105.5328, 1105.5722, 1105.6083, 1105.6383, 1106.7801, 1151.4019, 1151.4264, 1151.4769, 1151.5386, 1151.6222

Metabolite Options

Number of fragment peaks selected for assignment:	30
Minimum signal-to-noise ratio:	3
MS/MS m/z tolerance:	15 ppm
Fragmentation Settings	
Break aromatic rings:	True
Maximum number of bonds to break:	4
Maximum number of C-C bonds to break:	4

Label Settings

Label peaks with: Ion with ppm Error

Fragment Filters

None

Fragment Details	Structure Details
Mass (m/z): 943.4808<br>Ion Formula: C47H75O17S<br>Error (ppm): 8.2<br>Intensity (cps): 33.0<br>RDB: 10.0<br>Score: 33.5	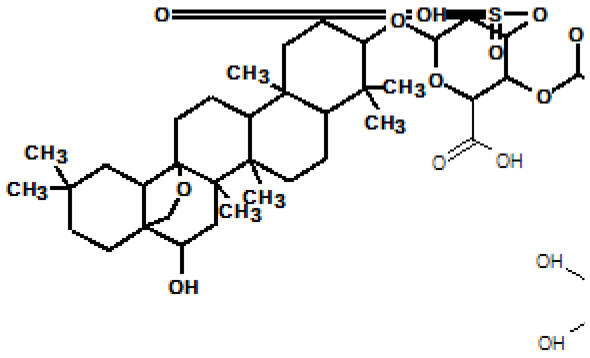<br>Selected: C47H76O17S<br>Mass: 944.4803<br>Broken Bonds: 2<br>Delta H: -2<br>Score: 33.5<br>**Contained Neutral Losses**<br>No contained neutral losses
Mass (m/z): 1083.5493<br>Ion Formula: C51H87O22S<br>Error (ppm): 7.2<br>Intensity (cps): 67.0<br>RDB: 8.0<br>Score: 25.0	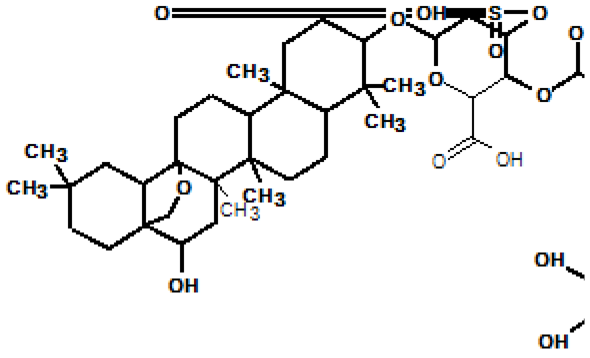<br>Selected: C51H83O22S<br>Mass: 1079.5097<br>Broken Bonds: 3<br>Delta H: 2<br>Score: 25.0

Fragment Details	Structure Details
	**Contained Neutral Losses**<br>No contained neutral losses
Mass (m/z): 1105.5328<br>Ion Formula: C53H85O22S<br>Error (ppm): 6.3<br>Intensity (cps): 175.0<br>RDB: 11.0<br>Score: 37.0	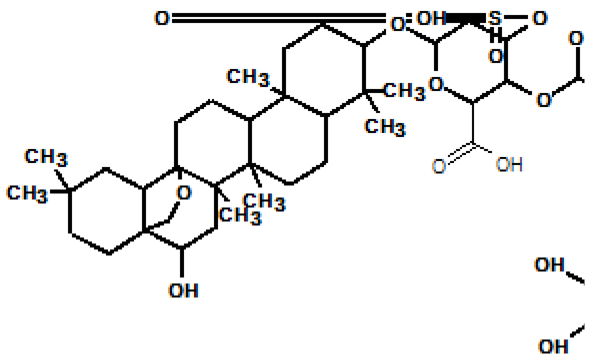<br>Selected: C53H87O22S<br>Mass: 1107.5410<br>Broken Bonds: 1<br>Delta H: -2<br>Score: 37.0<br>**Contained Neutral Losses**

Mass	Formula
162.0520	C6H10O5

Fragment Details	Structure Details
Mass (m/z): 1151.5386<br>Ion Formula: C54H87O24S<br>Error (ppm): 6.3<br>Intensity (cps): 358.0<br>RDB: 11.0<br>Score: 44.0	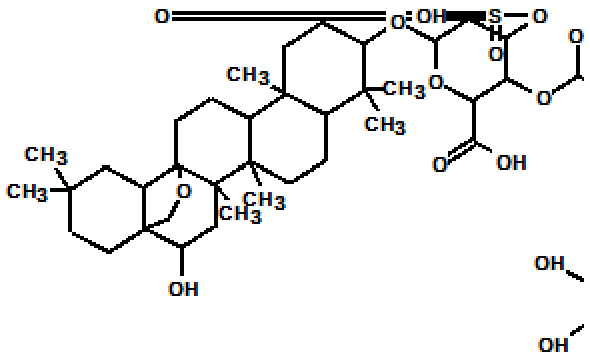<br>Selected: C54H88O24S<br>Mass: 1152.5386<br>Broken Bonds: 0<br>Delta H: 0<br>Score: 44.0<br>**Contained Neutral Losses**

Mass	Formula

Fragment Details	Structure Details	
	46.0058	CH_2O_2

Interpretation of the metabolite in rat (M26)

M26 – Loss of $C_6H_{10}O_4$+Demethylation and Methylene to Ketone [M-H]-

Formula:	$C_{53}H_{84}O_{24}$
ppm:	5.9
RDB:	12.0

Available Structure Candidates

Rank	Score	Count
1	100.0	1

Applied Metabolite Structure

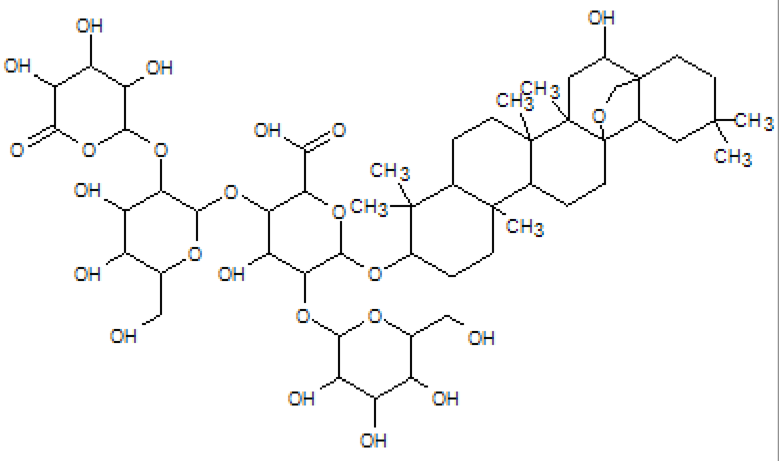

Composition:	$C_{53}H_{84}O_{24}$
Mass:	1104.5353

Metabolite MS/MS

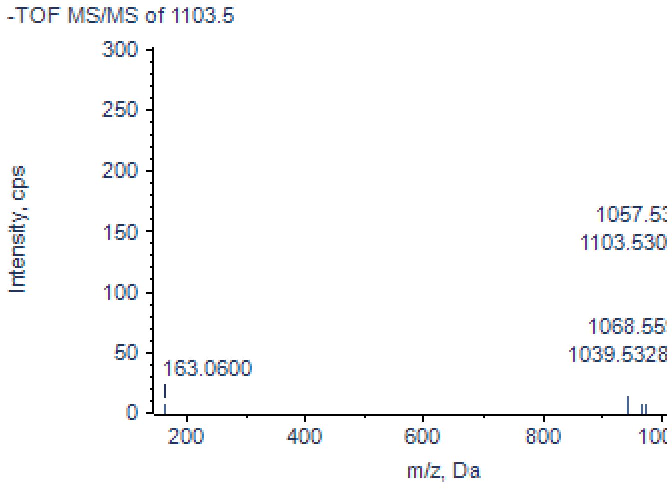

Parent MS/MS

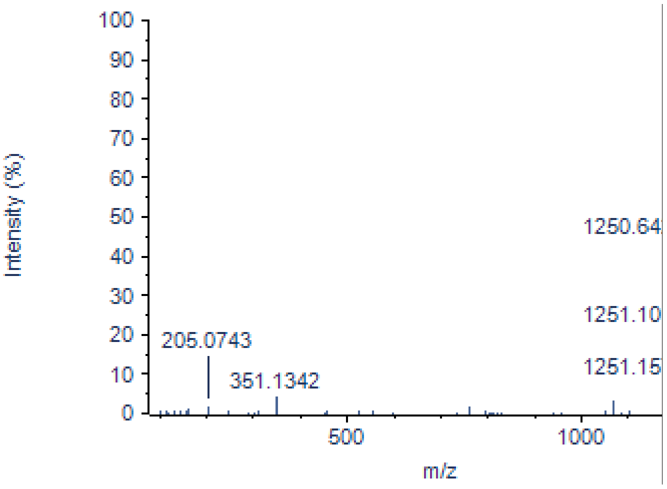

Peaks selected for assignment (m/z): 163.0600, 942.5484, 967.3164, 971.5122, 1039.5077, 1039.5328, 1049.5785, 1057.3690, 1057.4570, 1057.4829, 1057.5366, 1057.6153, 1067.4511, 1067.4806, 1067.5602, 1067.6211, 1067.6839, 1080.5168, 1085.5482, 1085.5931, 1103.3745, 1103.4427, 1103.4767, 1103.5303, 1103.5849

Metabolite Options

Number of fragment peaks selected for assignment:	30
Minimum signal-to-noise ratio:	3
MS/MS m/z tolerance:	15 ppm
Fragmentation Settings	
Break aromatic rings:	True
Maximum number of bonds to break:	4
Maximum number of C-C bonds to break:	4
Label Settings	
Label peaks with:	Ion with ppm Error

Fragment Filters

None

Fragment Details		Structure Details
Mass (m/z):	1039.5328	
Ion Formula:	C49H83O23	
Error (ppm):	-0.3	
Intensity (cps):	27.0	
RDB:	8.0	
Score:	8.0	

Fragment Details	Structure Details
Mass (m/z): 1057.5366<br>Ion Formula: C52H81O22<br>Error (ppm): 13.4<br>Intensity (cps): 134.0<br>RDB: 12.0<br>Score: 37.0	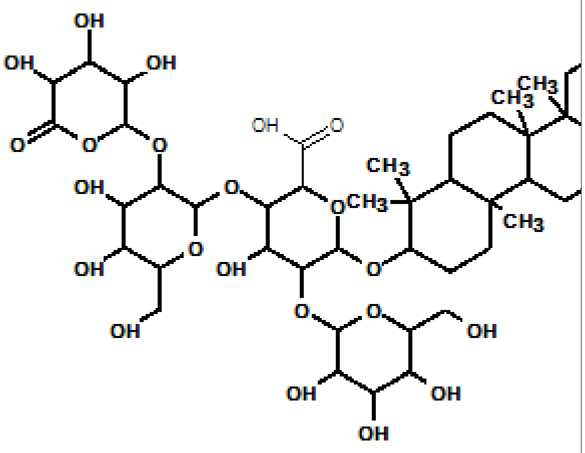<br>Selected: C52H83O22<br>Mass: 1059.5376<br>Broken Bonds: 1<br>Delta H: -2<br>Score: 37.0<br>**Contained Neutral Losses**<br>No contained neutral losses
Mass (m/z): 1103.5303<br>Ion Formula: C53H83O24<br>Error (ppm): 2.1<br>Intensity (cps): 107.0<br>RDB: 12.0<br>Score: 49.0	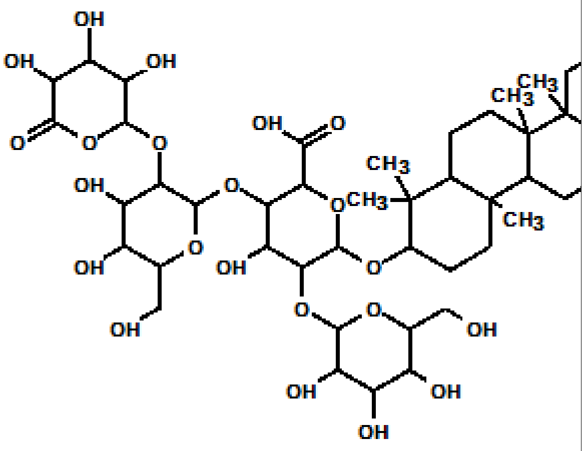<br>Selected: C53H84O24<br>Mass: 1104.5353<br>Broken Bonds: 0<br>Delta H: 0<br>Score: 49.0<br>**Contained Neutral Losses**

Mass	Formula
45.9937	CH2O2

Interpretation of the metabolite in rat (M27)

M27 – Loss of O and C6H10O6+Sulfate Conjugation [M-H]-

Formula:	C54H88O23S
ppm:	1.6
RDB:	11.0

Available Structure Candidates

Rank	Score	Count
1	100.0	10
2	0.0	100

Applied Metabolite Structure

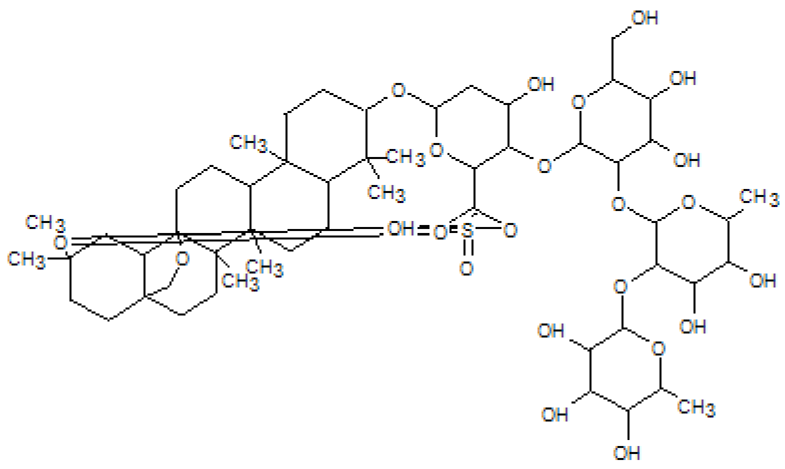

Composition:	C54H88O23S
Mass:	1136.5437

Metabolite MS/MS

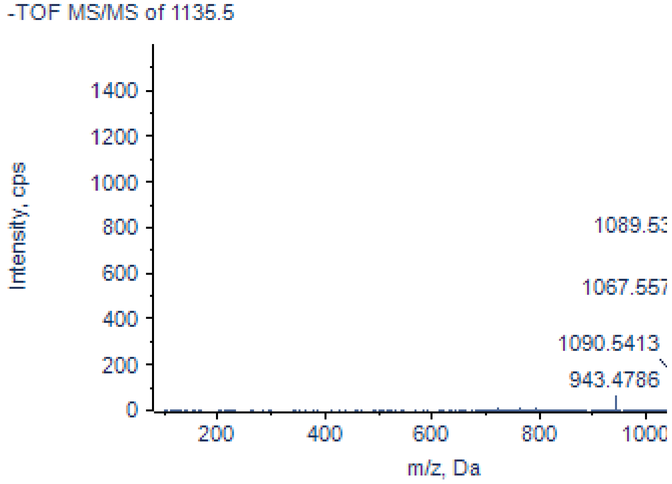

Parent MS/MS

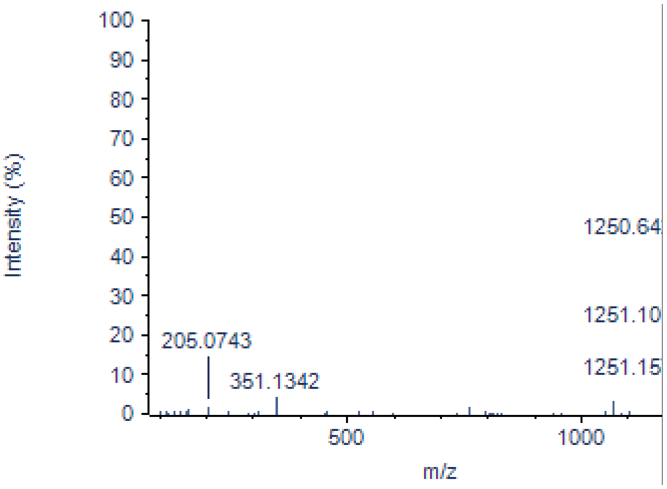

Peaks selected for assignment (m/z): 101.0247, 112.9874, 119.0446, 126.2826, 163.0653, 722.5663, 734.7942, 740.5025, 763.4120, 789.3445, 793.2904, 817.4260, 925.4514, 941.5129, 943.4786, 944.5002, 963.5201, 1005.5630, 1049.5385, 1049.5658, 1067.4891, 1067.5577, 1067.7919, 1085.9071, 1089.5387, 1090.5413, 1091.2523, 1134.8531, 1135.0875, 1135.5446

Metabolite Options

Number of fragment peaks selected for assignment:	30
Minimum signal-to-noise ratio:	3
MS/MS m/z tolerance:	15 ppm
Fragmentation Settings	
Break aromatic rings:	True
Maximum number of bonds to break:	4
Maximum number of C-C bonds to break:	4
Label Settings	
Label peaks with:	Ion with ppm Error

Fragment Filters

None

<table>
<tr><th>Fragment Details</th><th>Structure Details</th></tr>
<tr><td>Mass (m/z): 763.4120
Ion Formula: C41H63O11S
Error (ppm): 3.1
Intensity (cps): 7.4
RDB: 10.0
Score: 29.5</td><td>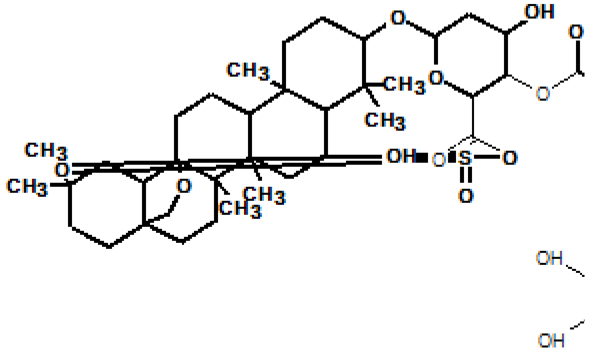
Selected: C41H66O11S
Mass: 766.4326
Broken Bonds: 3
Delta H: -4
Score: 29.5
Contained Neutral Losses
No contained neutral losses</td></tr>
<tr><td>Mass (m/z): 793.2904</td><td></td></tr>
</table>

Fragment Details	Structure Details
Ion Formula: C38H49O18<br>Error (ppm): -2.5<br>Intensity (cps): 5.6<br>RDB: 14.0<br>Score: 8.0	
Mass (m/z): 925.4514<br>Ion Formula: C47H73O16S<br>Error (ppm): -12.0<br>Intensity (cps): 5.4<br>RDB: 11.0<br>Score: 33.0	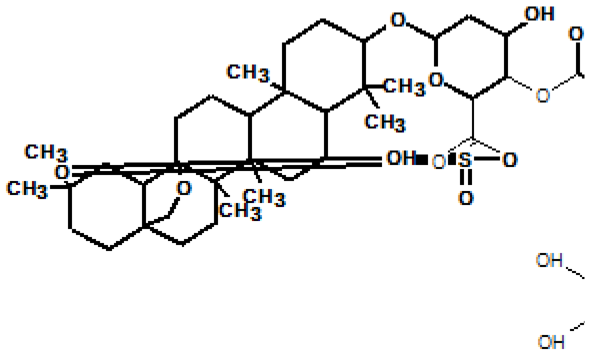<br>Selected: C47H77O16S<br>Mass: 929.4932<br>Broken Bonds: 2<br>Delta H: -4<br>Score: 33.0<br>**Contained Neutral Losses**<br>No contained neutral losses
Mass (m/z): 941.5129<br>Ion Formula: C48H77O18<br>Error (ppm): 1.5<br>Intensity (cps): 5.8<br>RDB: 10.0<br>Score: 8.0	
Mass (m/z): 943.4786<br>Ion Formula: C47H75O17S<br>Error (ppm): 5.8<br>Intensity (cps): 63.5<br>RDB: 10.0<br>Score: 33.5	

Fragment Details	Structure Details
	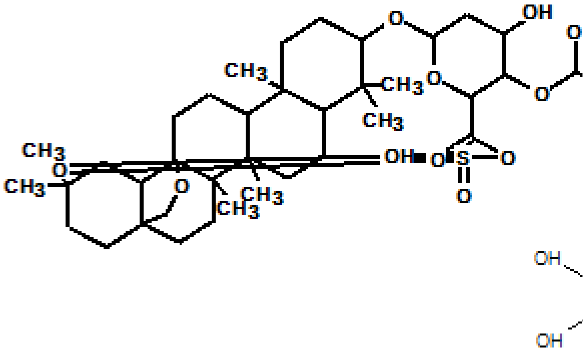<br>Selected: C47H74O17S<br>Mass: 942.4647<br>Broken Bonds: 2<br>Delta H: 0<br>Score: 33.5<br>**Contained Neutral Losses**<br>Mass: 18.0271 — Formula: H2O
Mass (m/z): 944.5002<br>Ion Formula: C51H76O16<br>Error (ppm): -14.5<br>Intensity (cps): 5.7<br>RDB: 13.5<br>Score: 23.0	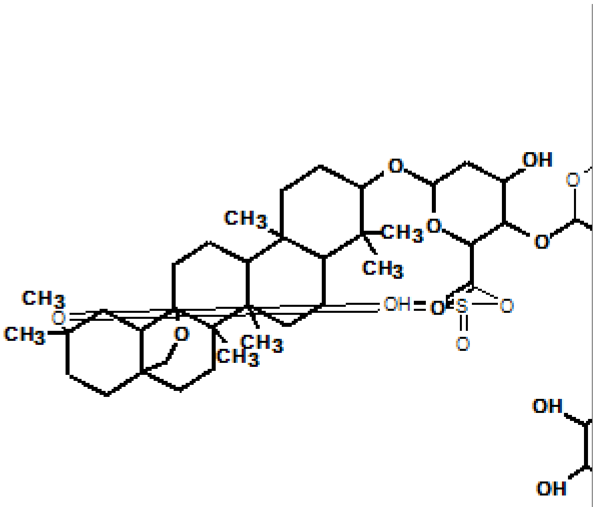<br>Selected: C51H81O16<br>Mass: 949.5525<br>Broken Bonds: 3<br>Delta H: -8<br>Score: 23.0<br>**Contained Neutral Losses**<br>No contained neutral losses
Mass (m/z): 1005.5630<br>Ion Formula: C50H85O20	

<table>
<tr><th>Fragment Details</th><th>Structure Details</th></tr>
<tr><td>Error (ppm): -0.9
Intensity (cps): 5.9
RDB: 8.0
Score: 8.0</td><td></td></tr>
<tr><td>Mass (m/z): 1049.5658
Ion Formula: C51H85O22
Error (ppm): 11.5
Intensity (cps): 7.3
RDB: 9.0
Score: 3.0</td><td></td></tr>
<tr><td>Mass (m/z): 1067.5577
Ion Formula: C51H87O21S
Error (ppm): 10.4
Intensity (cps): 380.7
RDB: 8.0
Score: 25.0</td><td>

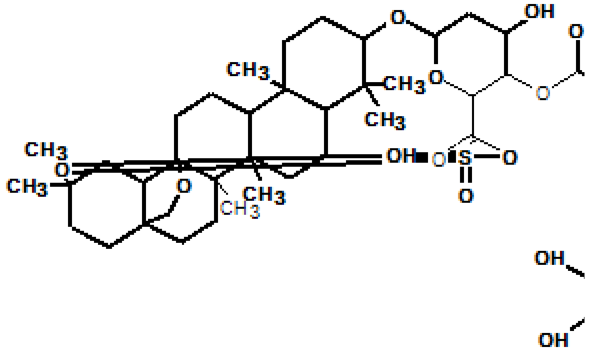

Selected: C51H84O21S
Mass: 1064.5226
Broken Bonds: 3
Delta H: 2
Score: 25.0

Contained Neutral Losses

Mass	Formula
18.0192	H2O

</td></tr>
<tr><td>Mass (m/z): 1089.5387
Ion Formula: C53H85O21S
Error (ppm): 7.2
Intensity (cps): 654.9
RDB: 11.0
Score: 37.0</td><td></td></tr>
</table>

<table>
<tr><th>Fragment Details</th><th>Structure Details</th></tr>
<tr><td></td><td>
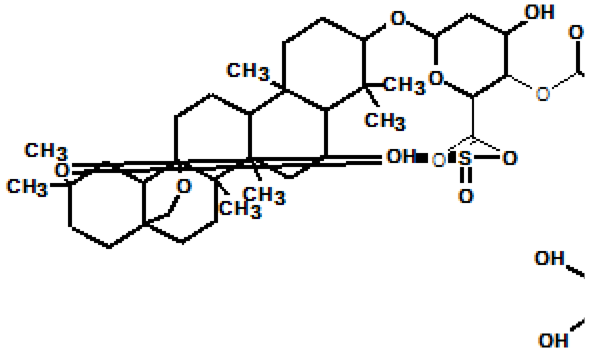

Selected: C53H88O21S
Mass: 1092.5539
Broken Bonds: 1
Delta H: -2
Score: 37.0

Contained Neutral Losses

<table>
<tr><th>Mass</th><th>Formula</th></tr>
<tr><td>146.0602</td><td>C6H10O4</td></tr>
</table>
</td></tr>
<tr><td>
Mass (m/z): 1135.5446
Ion Formula: C54H87O23S
Error (ppm): 7.2
Intensity (cps): 1598.7
RDB: 11.0
Score: 44.0
</td><td>
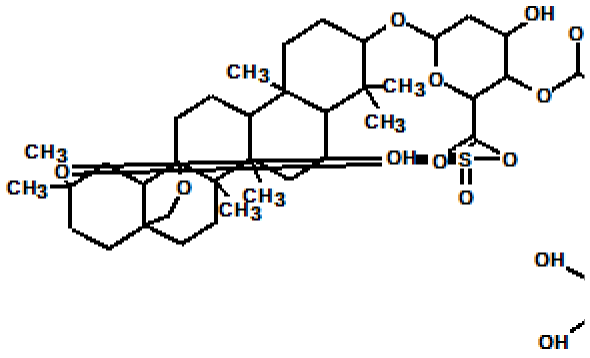

Selected: C54H88O23S
Mass: 1136.5437
Broken Bonds: 0
Delta H: 0
Score: 44.0

Contained Neutral Losses

<table>
<tr><th>Mass</th><th>Formula</th></tr>
<tr><td>46.0059</td><td>CH2O2</td></tr>
</table>
</td></tr>
</table>

Interpretation of the metabolite in rat (M28)

M28 – Loss of C30H48O24+Glucose Conjugation [M-H]-

Formula:	C36H60O8
ppm:	8.3
RDB:	7.0

Available Structure Candidates

Rank	Score	Count
1	100.0	2

Applied Metabolite Structure

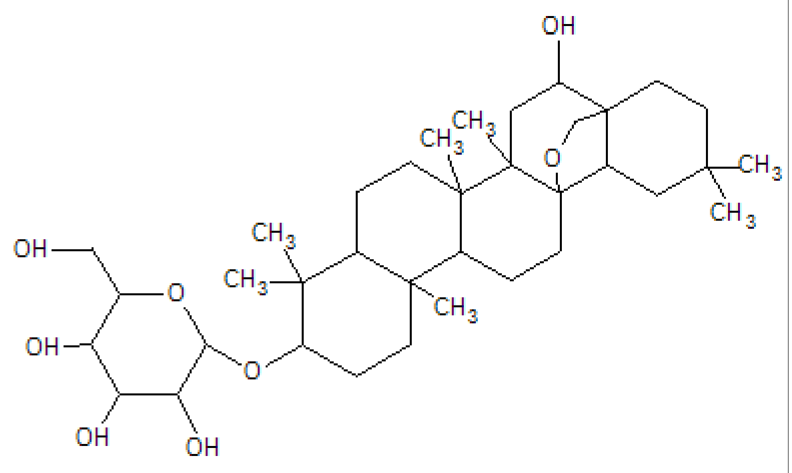

Composition:	C36H60O8
Mass:	620.4288

Metabolite MS/MS

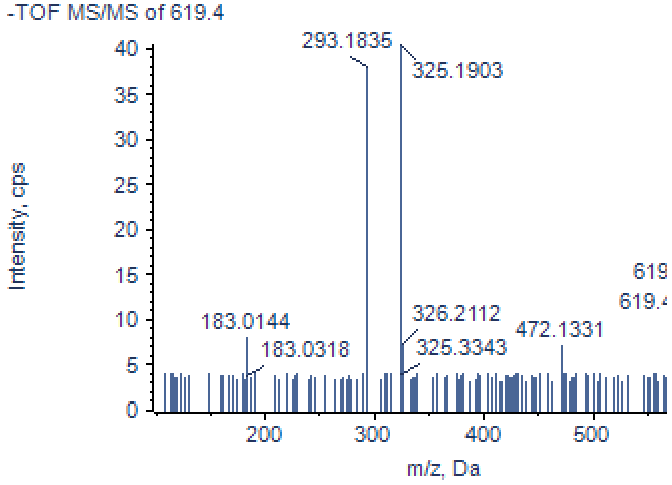

Parent MS/MS

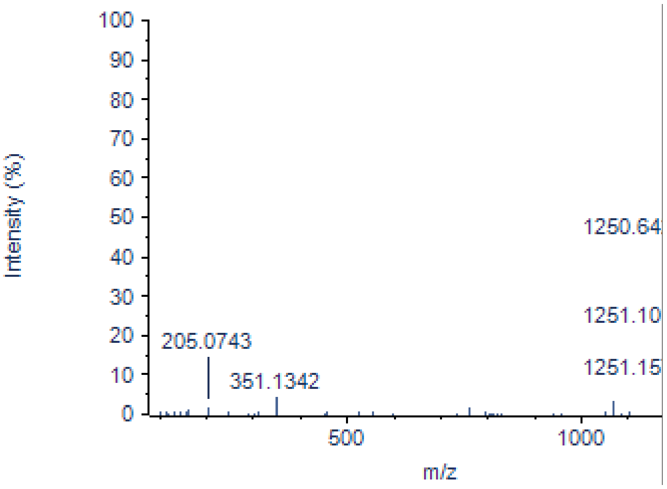

Peaks selected for assignment (m/z): 108.0472, 113.0264, 123.0606, 148.9623, 183.0144, 190.9803, 219.9820, 289.2268, 293.1835, 315.0284, 325.1903, 325.3343, 326.2112, 357.1568, 376.2242, 459.0020, 472.1331, 501.3310, 573.4261, 583.3969, 601.4234, 619.2320, 619.3423, 619.3676, 619.3751, 619.4047, 619.4237, 619.4430, 619.4560, 619.4847

Metabolite Options

Number of fragment peaks selected for assignment:	30
Minimum signal-to-noise ratio:	3
MS/MS m/z tolerance:	10 mDa
Fragmentation Settings	
Break aromatic rings:	True
Maximum number of bonds to break:	4
Maximum number of C-C bonds to break:	4
Label Settings	
Label peaks with:	Ion

Fragment Filters

None

Fragment Details	Structure Details
Mass (m/z): 293.1835<br>Ion Formula: C21H25O<br>Error (ppm): -7.6<br>Intensity (cps): 38.0<br>RDB: 9.0<br>Score: 3.0	
Mass (m/z): 325.1903<br>Ion Formula: C14H29O8<br>Error (ppm): 3.5<br>Intensity (cps): 40.4<br>RDB: 0.0<br>Score: 3.0	
Mass (m/z): 619.4237<br>Ion Formula: C36H59O8<br>Error (ppm): 2.2<br>Intensity (cps): 11.2<br>RDB: 7.0<br>Score: 44.0	

Fragment Details	Structure Details
	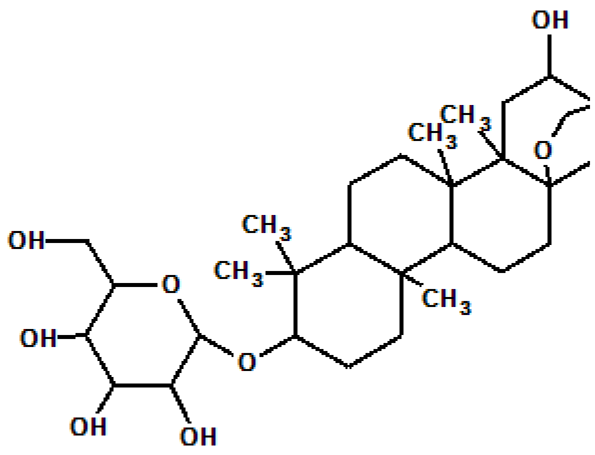 Selected: C36H60O8; Mass: 620.4288; Broken Bonds: 0; Delta H: 0; Score: 44.0. **Contained Neutral Losses** No contained neutral losses

Interpretation of the metabolite in rat (M29)

M29 – Loss of C18H30O14+Loss of Hydroxymethylene [M-H]-

Formula: C41H66O12

ppm: -6.8

RDB: 9.0

Available Structure Candidates

Rank	Score	Count
1	100.0	5
2	0.0	4

Applied Metabolite Structure

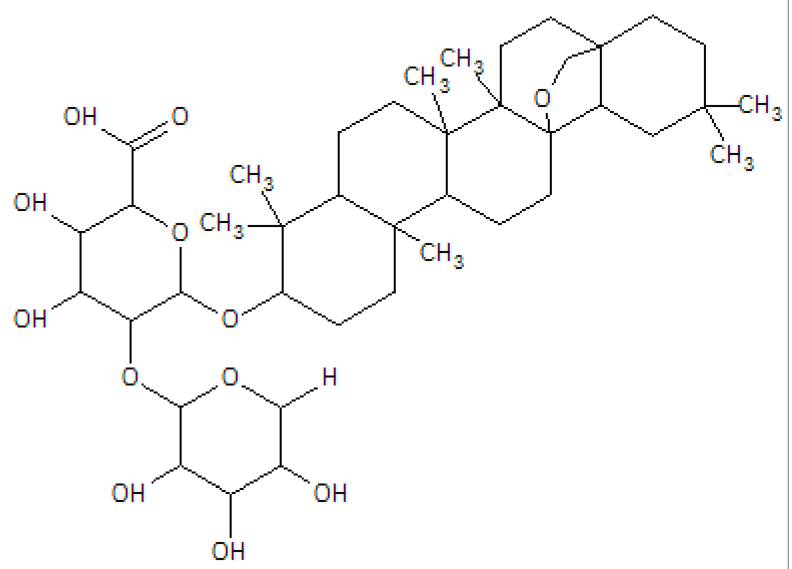

Composition:	C41H66O12
Mass:	750.4554

Metabolite MS/MS

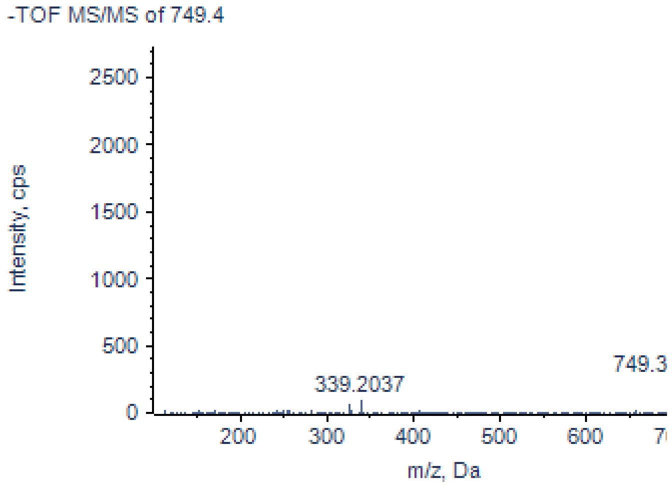

Parent MS/MS

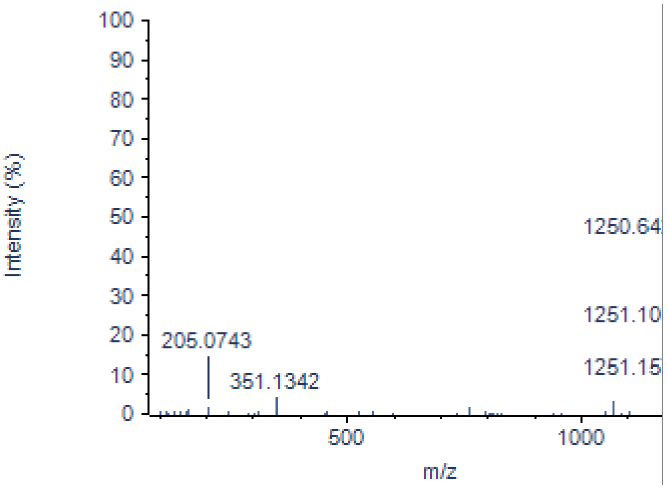

Peaks selected for assignment (m/z): 112.9857, 152.9976, 171.0078, 242.2209, 248.9596, 255.2358, 281.2517, 297.1690, 311.1677, 325.1875, 327.1871, 339.2037, 339.2221, 341.2030, 371.1717, 387.1460, 407.1887, 657.2507, 665.3751, 703.3264, 703.3567, 703.4102, 703.4522, 703.4860, 703.5297, 705.4759, 731.4928, 749.3501, 749.4433, 749.5335

Metabolite Options

Number of fragment peaks selected for assignment:	30
Minimum signal-to-noise ratio:	3
MS/MS m/z tolerance:	15 ppm

Fragmentation Settings

Break aromatic rings:	True

Maximum number of bonds to break: 4

Maximum number of C-C bonds to break: 4

Label Settings

Label peaks with: Ion

Fragment Filters

Fragments with assigned structures True

<table>
<tr><th>Fragment Details</th><th>Structure Details</th></tr>
<tr><td>Mass (m/z): 703.4102
Ion Formula: C39H59O11
Error (ppm): 5.6
Intensity (cps): 19.1
RDB: 10.0</td><td>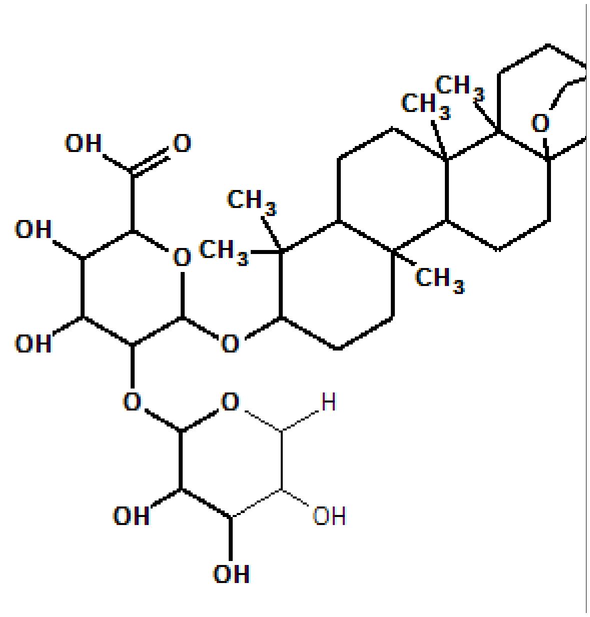

Selected: C39H62O11
Mass: 706.4292
Broken Bonds: 2
Delta H: -4
Contained Neutral Losses
No contained neutral losses</td></tr>
<tr><td>Mass (m/z): 749.4433
Ion Formula: C41H65O12
Error (ppm): -6.4
Intensity (cps): 2725.9
RDB: 9.0</td><td>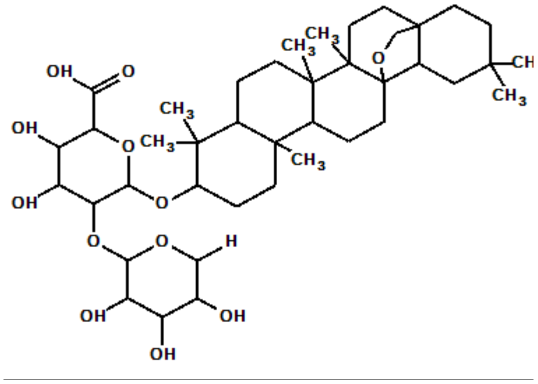

Selected: C41H66O12
Mass: 750.4554
Broken Bonds: 0
Delta H: 0
Contained Neutral Losses</td></tr>
</table>

Fragment Details	Structure Details
	No contained neutral losses

Interpretation of the metabolite in rat (M30)

M30 – Loss of C18H30O14+Methylation [M-H]-

Formula: C43H70O13

ppm: -9.0

RDB: 9.0

Available Structure Candidates

Rank	Score	Count
1	100.0	35
2	0.0	35

Applied Metabolite Structure

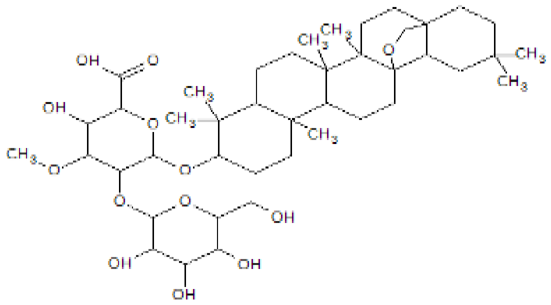

Composition: C43H70O13
Mass: 794.4816

Metabolite MS/MS

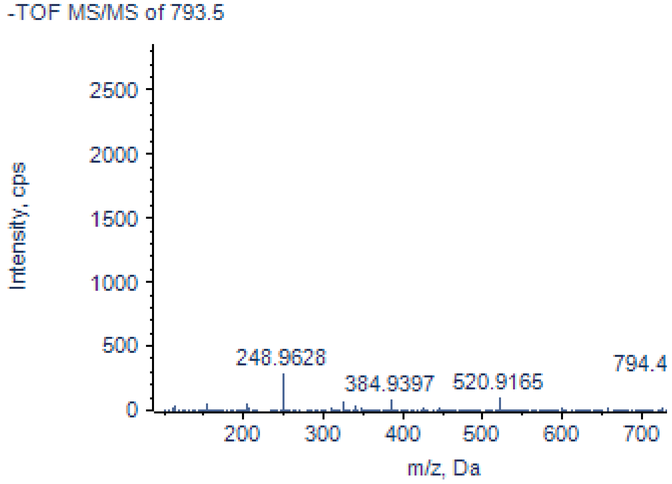

Parent MS/MS

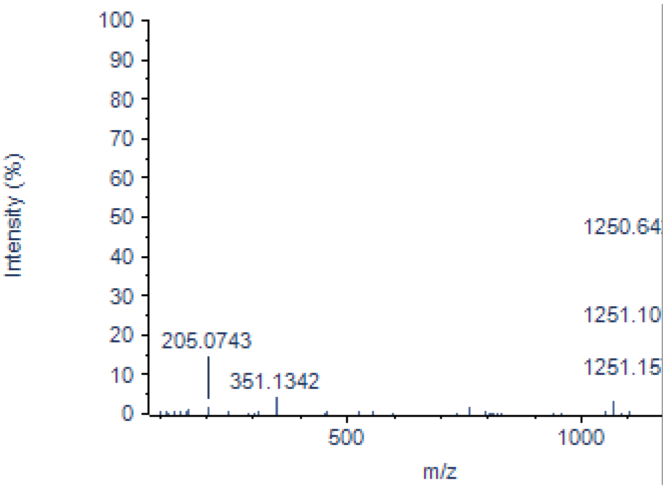

Peaks selected for assignment (m/z): 112.9869, 152.9797, 154.9760, 183.0112, 196.9696, 204.9724, 248.9628, 249.9844, 311.1699, 314.1032, 325.1872, 339.2049, 348.0914, 384.9397, 385.9776, 426.9271, 445.2952, 467.2791, 520.9165, 521.9520, 521.9746, 551.2357, 599.3213, 656.8920, 701.4508, 725.3688, 747.4626, 747.4990, 756.4026, 793.4691

Metabolite Options

Number of fragment peaks selected for assignment:	30
Minimum signal-to-noise ratio:	3
MS/MS m/z tolerance:	15 ppm
Fragmentation Settings	
Break aromatic rings:	True
Maximum number of bonds to break:	4
Maximum number of C-C bonds to break:	4
Label Settings	
Label peaks with:	Ion with ppm Error

Fragment Filters

Fragments with assigned structures	True

Fragment Details
